# The immune-modulating pregnancy-specific glycoproteins evolve rapidly and their presence correlates with hemochorial placentation in primates

**DOI:** 10.1186/s12864-021-07413-8

**Published:** 2021-02-18

**Authors:** Wolfgang Zimmermann, Robert Kammerer

**Affiliations:** 1 Tumor Immunology Laboratory, LIFE Center, Department of Urology, University Hospital, LMU Munich, Germany; 2 Institute of Immunology, Friedrich-Loeffler-Institut, Federal Research Institute for Animal Health, Greifswald, Insel Riems Germany

**Keywords:** Pregnancy-specific glycoprotein (PSG), Carcinoembryonic antigen-related cell-cell adhesion molecule (CEACAM), Immunoglobulin superfamily, Positive selection, Primates, Trophoblast, Hemochorial placenta

## Abstract

**Background:**

*Pregnancy-specific glycoprotein* (*PSG*) genes belong to the *carcinoembryonic antigen* (*CEA*) gene family, within the immunoglobulin gene superfamily. In humans, 10 *PSG* genes encode closely related secreted glycoproteins. They are exclusively expressed in fetal syncytiotrophoblast cells and represent the most abundant fetal proteins in the maternal blood. In recent years, a role in modulation of the maternal immune system possibly to avoid rejection of the semiallogeneic fetus and to facilitate access of trophoblast cells to maternal resources via the blood system has been suggested. Alternatively, they could serve as soluble pathogen decoy receptors like other members of the CEA family. Despite their clearly different domain organization, similar functional properties have also been observed for murine and bat PSG. As these species share a hemochorial type of placentation and a seemingly convergent formation of *PSG* genes during evolution, we hypothesized that hemochorial placentae support the evolution of *PSG* gene families.

**Results:**

To strengthen this hypothesis, we have analyzed *PSG* genes in 57 primate species which exhibit hemochorial or epitheliochorial placentation. In nearly all analyzed apes some 10 *PSG* genes each could be retrieved from genomic databases, while 6 to 24 *PSG* genes were found in Old World monkey genomes. Surprisingly, only 1 to 7 *PSG* genes could be identified in New World monkeys. Interestingly, no PSG genes were found in more distantly related primates with epitheliochorial placentae like lemurs and lorises. The exons encoding the putative receptor-binding domains exhibit strong selection for diversification in most primate *PSG* as revealed by rapid loss of orthologous relationship during evolution and high ratios of nonsynonymous and synonymous mutations.

**Conclusion:**

The distribution of trophoblast-specific PSGs in primates and their pattern of selection supports the hypothesis that PSG are still evolving to optimize fetal-maternal or putative pathogen interactions in mammals with intimate contact of fetal cells with the immune system of the mother like in hemochorial placentation.

**Supplementary Information:**

The online version contains supplementary material available at 10.1186/s12864-021-07413-8.

## Background

In placental mammals, the fetus develops in a protected environment inside the uterus of the mother. There, the placenta provides the growing fetus with nutrients, allows removal of waste products and serves as an immunological barrier to protect the fetus from the maternal immune system and infectious agents. Numerous placental variants exist. However, three major types can be discerned differing in the number and type of cell layers which separate the maternal and fetal blood systems: epitheliochorial, endotheliochorial and hemochorial placentae. The most intimate contact is found in mammals with hemochorial placentation where maternal blood is in direct contact with fetal trophoblast cells of the chorionic villi. This facilitates efficient nutritional supply of the fetus but is more demanding to maintain gestational tolerance of the maternal immune system towards semiallogeneic fetal cells. Little is known about molecular factors which specifically support hemochorial placentation.

Pregnancy-specific glycoproteins (PSG) may represent such molecules. PSGs are secreted proteins and are nearly exclusively expressed in trophoblast cells in human as well as rodent (mouse and rat) placentae both being of the hemochorial type [1, 2]. PSG were also described in a subgroup of bats and in the horse [3, 4]. While bats with PSGs possess also hemochorial placentae, the horse has an epitheliochorial placenta. However, in the horse unique trophoblast cells exist that invade the endometrium and are recognized by the maternal immune system, thus these trophoblast cells have a similar intimate contact with the maternal immune system as in hemochorial placentae [5]. PSG belong to the CEA family which is a member of the immunoglobulin superfamily. In humans and mice they are encoded by 10 and 17 closely linked genes, respectively [6]. In the horse, 8 PSG-like genes were described and 4 of them were shown to be expressed by trophoblast cells and in some microbat species up to 50 PSGs were identified [3, 4]. PSGs differ significantly in their domain organization: while human PSGs consist of one N-terminal immunoglobulin (Ig) variable-like (IgV-like) or N domain and 2–3 Ig constant-like (IgC-like) domains, murine PSGs contain multiple IgV-like N domains (commonly 3) and one carboxy-terminal IgC-like domain, bat PSGs consist of a single N domain or one N domain and one IgC-like domain and in horse PSGs are built from a single N domain [3, 4, 6, 7]. These facts and the non-syntenic location of the human and murine PSG loci strongly suggest independent generation of these genes by convergent evolution [8].

Despite the marked differences in their overall domain organization, similar functional properties have been observed for human and murine PSG. Individual PSG family members have been shown to exhibit immunoregulatory, pro-angiogenic and a possible antithrombotic function [6, 9, 10]. The immune regulatory function involves release of anti-inflammatory cytokines from monocytes and macrophages [11]. Some if not all of the tolerogenic and pro-angiogenic effects appear to be mediated through the transforming growth factor β1 (TGFβ1) signaling pathway [12–15]. In vitro experiments suggest that a dual function attributed to different PSG domains exists. A region of the N domain of PSG1 around the lysine-tyrosine-histidine-tyrosine (LYHY) tetra-peptide motif appears to be responsible for the release of activated TGFβ1 from macrophages and other immune cells while the C-terminal IgC-like B2 domain of human PSGs and the N domain of murine and equine PSGs are responsible for activation of so called latent TGFβ1 [16–18]. Furthermore, in vitro platelet-fibrinogen interaction involving αIIbβ3 integrin is compromised by recombinant human PSG1 which binds to αIIbβ3 [10]. An arginine-glycine-aspartic acid-like (RGD-like) tri-peptide motif present in integrin-interacting proteins like fibrinogen and disintegrins is found in a loop of the N domain of most human and rodent PSGs was expected to target the platelet integrin [19]. Mutational analysis revealed, however, that this is not the case. Therefore, the mechanism how PSGs potentially prevent platelet aggregation in the prothrombotic maternal environment during pregnancy is still unclear.

PSGs exist only in a minority of mammals [8]. Species with a less invasive placenta type (e.g. endotheliochorial and epitheliochorial) like dogs and cattle were found to have no *PSG* genes. Thus we hypothesized that the presence of a hemochorial placenta or otherwise highly invasive trophoblast cell populations in direct contact with the maternal immune system drive the formation of *PSG* gene families [3, 8]. To strengthen this hypothesis, we have analyzed 57 primate and four closely related species (1 flying lemur, 3 tree shrew species) with hemochorial or epitheliochorial placentae for the presence of *PSG* genes. Indeed, all analyzed lemur and a loris species which exhibit epitheliochorial placentation lacked *PSG* genes. On the other hand, the genomes of haplorhine apes, Old World monkeys (OWM) and New World monkeys (NWM) with hemochorial placentation contained highly variable numbers of *PSG* genes. Only in tarsius a distantly related haplorhine primate with a hemochorial placenta no *PSG* genes could be identified.

## Results

### Differential expansion of *PSG* genes in primates at syntenic loci

**Fig. 1:**
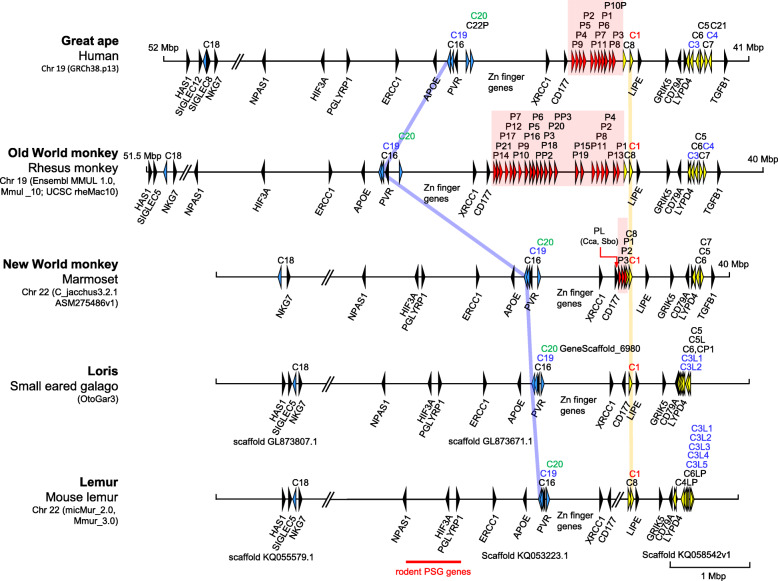
**CEACAM loci in primates.** The chromosomal arrangement of CEACAM genes in selected primates is depicted. Arrowheads represent genes with their transcriptional orientation. The PSG genes are shown in red, CEACAM1-related CEACAM genes in yellow, orthologous CEACAM genes in blue, selected flanking genes in black. The PSG clusters are marked with red boxes. The nonsyntenic localization of the PSG cluster in rodents is indicated by a red line at the bottom. The CEACAM gene loci were aligned along the position of CEACAM1 (yellow line), the CEACAM16 loci are connected with a blue line. Names of CEACAM1-like genes with ITIM/ITSM-encoding exons are shown in red and with ITAM and ITAM-like motif-encoding exons in green and blue, respectively. The nucleotide numbering of the chromosomes starts at the telomere of the short arms which point to the right. The chromosomal or scaffold location, databases and their versions used are indicated below the species name. The place corresponding to the location of the PSG-like gene (not found in marmoset) in capucin (Cca) and Bolivian squirrel monkeys (Sbo) is indicated by a red arrow. Of note: The primate genomes (except the human genome) are not completely refined yet. Therefore, not all CEACAM genes identified in WGS databases have been found in the published assembled genomes. C, CEACAM; Chr, chromosome; CP, CEACAM pseudogene; C3L(P), C4L(P), C5L, C6L(P), CEACAM3, 4, 5, 6-like (pseudo)gene; Mbp, million base pairs; P, pregnancy-specific glycoprotein (PSG) genes; PL, PSG-like; P10P, PSG10 pseudogene; PP2, PP3, PSG pseudogene 2, 3.

Functionally related PSG have been formed independently in humans, horse and rodents. Independent evolution is supported by both structural differences (i.e. number and type Ig domains) and localization of mouse and human *PSG* gene clusters at non-syntenic regions within the otherwise conserved *CEA* gene cluster (Fig. 1) [7, 8]. Presently, it is unknown, however, whether primate *PSG* loci have evolved at syntenic locations from a common ancestral *PSG*. To this end we compared the *CEA* loci of three haplorhine primates human (*Homo sapiens*), rhesus monkey (*Macaca mulatta*) and marmoset (*Callithrix jacchus*) representing great apes, Old World monkeys (OWM) and New world monkeys (NWM), respectively, as well as of the strepsirrhine primate species small-eared galago (*Otolemur garnettii*) and the gray mouse lemur (*Microcebus murinus*), using the corresponding annotated genomes available at the *Ensembl* and the *UCSC Genome Browsers*. In all three Haplorhini primates *PSG* gene clusters encoding secreted glycoproteins could be identified between *CD177* and the *CEACAM* genes *CEACAM8/CEACAM1* (Fig. 1). Their number of genes found in these databases varied between 23 in *Macaca mulatta,* 11 in *Homo sapiens* and three in *Callithrixs jacchus*. Despite a similar organization of the *CEA* gene locus and flanking non-*CEACAM* genes, no *PSG*-related genes were found in the galago and in the mouse lemur (Fig. 1).

### *PSG* genes are present in haplorhine but not in strepsirrhine primates

**Fig. 2:**
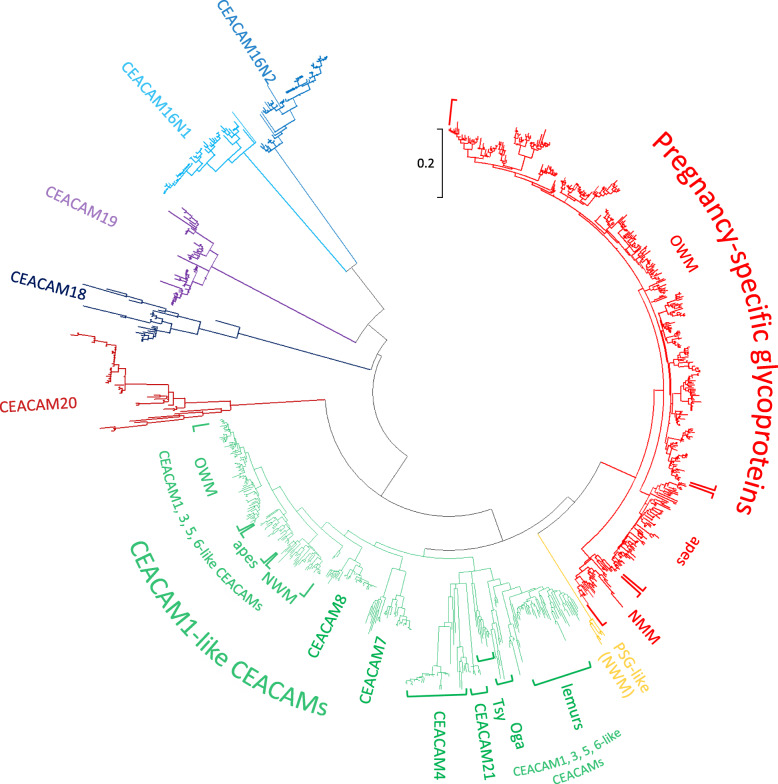
**Orthologous and paralogous relationship of CEACAM genes in primates.** Phylogenetic trees were constructed based on IgV-like N domain exon nucleotide sequences of CEACAM genes from 57 primate species using the Maximum Likelihood method (MEGA6 software). The tree with the highest log likelihood is shown. The CEACAM1-like genes are marked in green, the PSG paralogs in red. CEACAM1, CEACAM3, CEACAM5 and CEACAM6 represent paralogs and, CEACAM4, CEACAM7, CEACAM8 and CEACAM21 exhibit an orthologous relationship. Note that CEACAM21 and PSG-like genes are only present in apes and NWM, respectively. The scale next to the dendrogram shows the number of substitutions per site. CEACAM, CEA-related cell-cell adhesion molecule; NWM, New World monkeys; OWM, Old World monkeys; Oga, *Otolemur garnettii*, bushbaby; PSG, pregnancy-specific glycoprotein; Tsy, *Tarsius syrichta*, tarsier

**Fig. 3:**
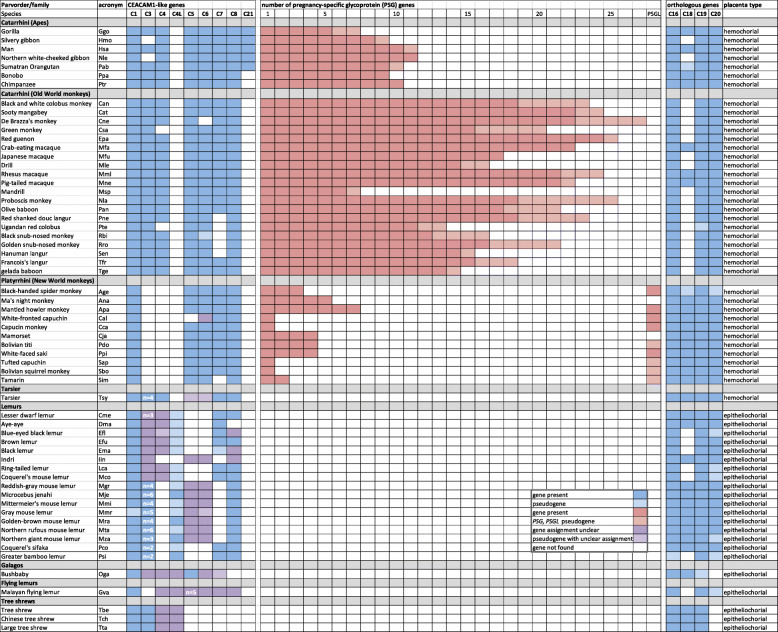
**PSG genes exist only in primates with hemochorial placentation.** Genomic databases of 57 primate species and, for comparison, of four closely related non-primate species (flying lemur, three treeshrew species) were screened for CEACAM genes based on the presence of IgV-like domain (N) exons as described in the Methods section (for genomic data source see Supplementary Table 1). Genes for which no N exons could be identified are shown as white boxes. Genes were registered as pseudogenes when the N domain exons contained stop codon(s) or noncanonical splice acceptor or donor sequences. They are shown in in lighter color. Genes which could not be clearly assigned to orthologous CEACAM1-like genes are shown in purple. If more than one CEACAM3-related gene was found the number of paralogs is shown in the corresponding boxes. The number of GPI-linked CEACAM paralogs in the flying lemur is also indicated. The Latin names from which the species’ acronyms were derived are listed in Supplementary Table 1. The species’ placenta type is indicated at the right

**Fig. 4:**
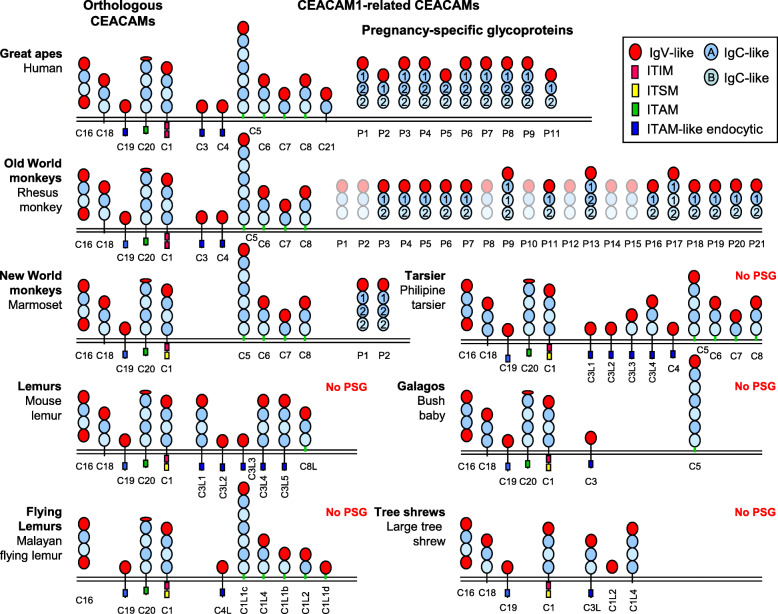
**Domain organization of CEACAM proteins in primates.** The domain organization of CEACAM family members from selected primates was predicted by gene analysis. Human and rhesus monkey PSG domain organizations were confirmed by EST sequences. Domains are shown in light colors for rhesus monkey PSGs not found in the EST database. Their domain composition is deduced from genomic analyses. If more than one splice variant exists, the longest is shown. The orthologous CEACAM family members are conserved and counterparts can be assigned between primate species. CEACAM1-related members represent paralogs and are much more variable between primate species. IgV-like domains are shown as red, IgC-like domains as blue ovals with subtypes A and B shown in darker and lighter coloration, respectively. Numbers in the IgC-like domains identify their origin from the first or second A, B exon pair in the PSG gene. Note that in human PSGs B1 exons are never spliced-in due to a splice acceptor defect in most PSG genes except in PSG11. In rhesus monkey such splice events are observed (PSG9, PSG17). The predicted signaling motifs in the cytoplasmic domains are schematically shown as green (ITAM), blue (ITAM-like motif), red (ITIM) and yellow boxes (ITSM). Transmembrane domains and GPI anchors are indicated by black and green lines, respectively. Note the highly variable number of PSG in the different primate species (between 0 and 21). Identical PSG numbering does not imply orthologous relationship. Also note the lack of CEACAMs with ITAM-like motifs in NWM and their expansion in tarsius and mouse lemur. For the bush baby the domain organization could not be delineated for all CEACAM1-like proteins due to low quality of the genome assembly. C, CEACAM; P, PSG

In order to substantiate the correlation of the presence and absence of *PSG* in Haplorhini and Strepsirrhini primates, respectively, we analyzed the *PSG* gene content in 39 haplorhine and 18 strepsirrhine primates (Supplementary Fig. 1). As a first step, N domain exons from human *PSG* genes were used to screen primate nucleotide databases. *PSG* candidate sequences of individual primate species were used for comprehensive in depth screening of whole genome shot-gun (WGS) sequence data bases. In all analyzed haplorhine primates except for the NWM species, *Cebus capuchinus* and *Saimiri boliviensis*, and *Tarsius syrichta* (see below) *PSG* genes could be identified using this strategy. Alignments of *CEACAM1*-like *CEACAM* and *PSG* as well as the more distantly related *CEACAM16-CEACAM20* N domain exon sequences from all analyzed primate species revealed that *PSG* genes can clearly be differentiated from other *CEACAM* genes in haplorhine primates. *PSG* exhibit a paralogous relationship like *CEACAM1, CEACAM3, CEACAM5* and *CEACAM6* N domain exon sequences in being often more closely related among each other within a given species than between species (Fig. 2). In contrast, *CEACAM4*, *CEACAM7*, *CEACAM8*, *CEACAM16, CEACAM18, CEACAM19, CEACAM20* and *CEACAM21* N domain exon nucleotide sequences were found to be most closely related with their counterparts in haplorhine species thus exhibiting an orthologous relationship (Fig. 2). The number of non-pseudogene *PSG* genes (for definition see Method section) varied widely between primate families: in apes from 5 *PSG* (gorilla) to 11 (Northern white cheeked gibbon), in OWM from 6 (mandrill) to 24 (red guenon) and 1–7 in NWM (Supplementary Table 1; Fig. 3; Supplementary Fig. 1). On average 3- and 6-fold more *PSG* genes were found in ape (mean ± standard error of the mean (SEM): 8.9 ± 0.7) and OWM subgroups (16.8 ± 1.0), respectively in comparison to NWM (2.9 ± 0.6). In the tarsier *Tarsius syrichta* the most distantly related haplorhine primate analyzed, a total of 15 *CEACAM1-related* N exon-containing genes could be identified. Four of the *CEACAM1*-like genes contain a nonsense mutation in their N exons. Five (including one pseudogene) encode GPI signal sequences, another five immunoreceptor tyrosine-based activation-related motifs (ITAM-like) and one an immunoreceptor tyrosine-based switch motif (ITSM) (Supplementary Table 1; Fig. 3; Fig. 4). The one unassigned gene apparently lacks exons encoding IgC-like domains (data not shown) which makes a *PSG* assignment unlikely. No *PSG* genes could be detected in 16 lemurs and 1 loris (Fig. 1; Fig. 2; Fig. 3; Supplementary Fig. 1; Supplementary Table 1).

In summary, *PSG* genes were detected in 38 out of 39 Haplorhini primates (with *Tarsius syrichta* being the single exception) but not in 17 Strepsirrhini primates.

### Differential conservation of putative functional motifs in primate PSGs

Two putative functional amino acid sequence motifs have been identified in the N domain of human PSGs: the leucine-tyrosine-histidine-tyrosine (LYHY) motif which is needed for the induction of latent TGFβ1 secretion by macrophages [16] and the disintegrin-like motif lysine/arginine-glycine-aspartic acid/glutamic acid (K/RGD/E) which could be involved in disruption of platelet-fibrinogen interaction by binding to platelet αIIbβ3 integrin by PSGs [10]. Interestingly, the LYHY motif is conserved on average in more than 80% of ape PSGs. However, it is either not found at all in NWM or on average in only 6% of OWM PSGs (Supplementary Fig. 2A, B). Of note, LYHY-containing OWM PSGs could only be identified in the *Colobinae* subfamily (Supplementary Fig. 1). Taken to gether, this suggests functional diversification during primate evolution or relaxed motif requirements.

**Fig. 5:**
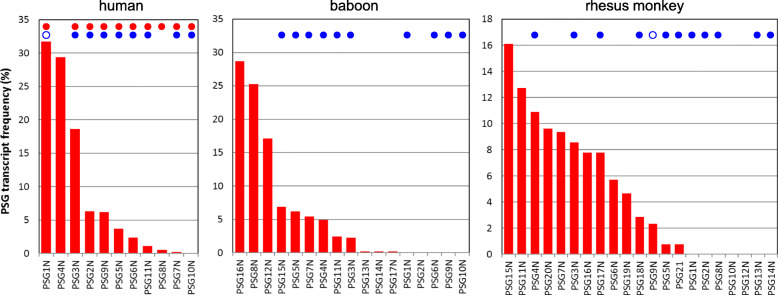
**The most highly expressed human and OWM PSG genes lack the disintegrin-like RGD motif.** The relative expression frequencies of PSG were estimated by counting the clones matching N exon nucleotide sequences of the different PSG genes present in human, baboon and rhesus monkey placental EST libraries. Only hits with E val = 0.0 were assigned to a given gene. A total number of 2302, 404 and 345 PSG clones, were identified in human, baboon and rhesus monkey, respectively. The presence of the LYHY motif needed for latent TGFβ1 secretion and the disintegrin-like RGD motif are shown as red and blue filled-in circles, respectively. The presence of RGD motifs with conservative amino acid changes (KGD, RGE) are indicated by blue open circles. Of note: The PSG genes were numbered arbitrarily. Therefore, no orthology can be inferred for genes with the same numbers between different species

In contrast, the K/RGD/E motif is found on average in 80 and 71% of apes and OWM PSGs, respectively, but rarely in PSGs of NWM species (14%) (Supplementary Fig. 2B). It is interesting to note that human PSG4, olive baboon PSG8, PSG12 and PSG16 as well as rhesus monkey PSG11 and PSG15 which lack these motifs are among the most highly expressed PSGs as estimated by their cDNA frequency in placental expressed sequence tag (EST) libraries (Fig. 5). This could indicate that highly expressed PSG might differ in function from low or moderately expressed PSGs.

The N-terminal IgV- and the C-terminal IgC-related domain of subtype B in human PSG1 appear to play a role in TGFβ1 release and activation, respectively [16]. Additional IgC-related domains of unknown function are regularly found in between. Primate *PSG* genes contain a duplicated set of IgC-related A and B domain exons named A1, B1 and A2, B2. In humans, the B1 exon splice acceptor is corrupted in most *PSG* genes except for *PSG11* (Supplementary Fig. 3). OWM *PSG* genes exhibit the same exon organization as human *PSG* genes, but their B1 exon splice acceptor sites appear to be functional. Analysis of EST data revealed, however, that this exon is rarely spliced in. In these rare cases, no PSG with 4 IgC-related domains are observed (Fig. 4). Possibly only a certain distance between the two functional domains of PSGs (N, B2) created by 1 or 2 IgC-related domains is permissible for PSG function.

### Selection for diversification in primate PSG

Phylogenetic analyses of *PSG* genes in great apes revealed rapid divergence of N domain exon sequences as demonstrated by the lack of an orthologous relationship of the majority of orangutan and all gibbon *PSG* genes with ape *PSG* genes evident from intraspecies clusters (Supplementary Fig. 4A). Two of the orangutan *PSG* N domain sequences, however, cluster with the corresponding sequences of human, bonobo, chimpanzee and gorilla (*PSG4, PSG5*; note that only 5 non-pseudogene *PSG* could be identified in gorilla) and thus probably represent orthologs (Supplementary Fig. 4A). In addition, no orthologs could be identified in NWM (with the exception of the single *PSG* gene found in the closely related capucin species) and between *PSG* genes of the OWM *Colobinae* and *Cercopithecinae* subfamilies (Supplementary Fig. 4B, C). This indicates that although all primates inherited probably the same number of *PSG* genes from a common ancestor, rapid divergence led to an extended expansion or contraction of the PSG family as well as loss of an orthologous relationship. This is in contrast to *CEACAM7* and *CEACAM8,* other primate CEACAM1-related members, for which an orthologous relationship can be observed with the corresponding N domain exons of primates as distantly related as NWM (Fig. 2). NWM diverged almost 2.5 times earlier from humans than orangutan (33 versus 13 million years ago [20]).

**Fig. 6:**
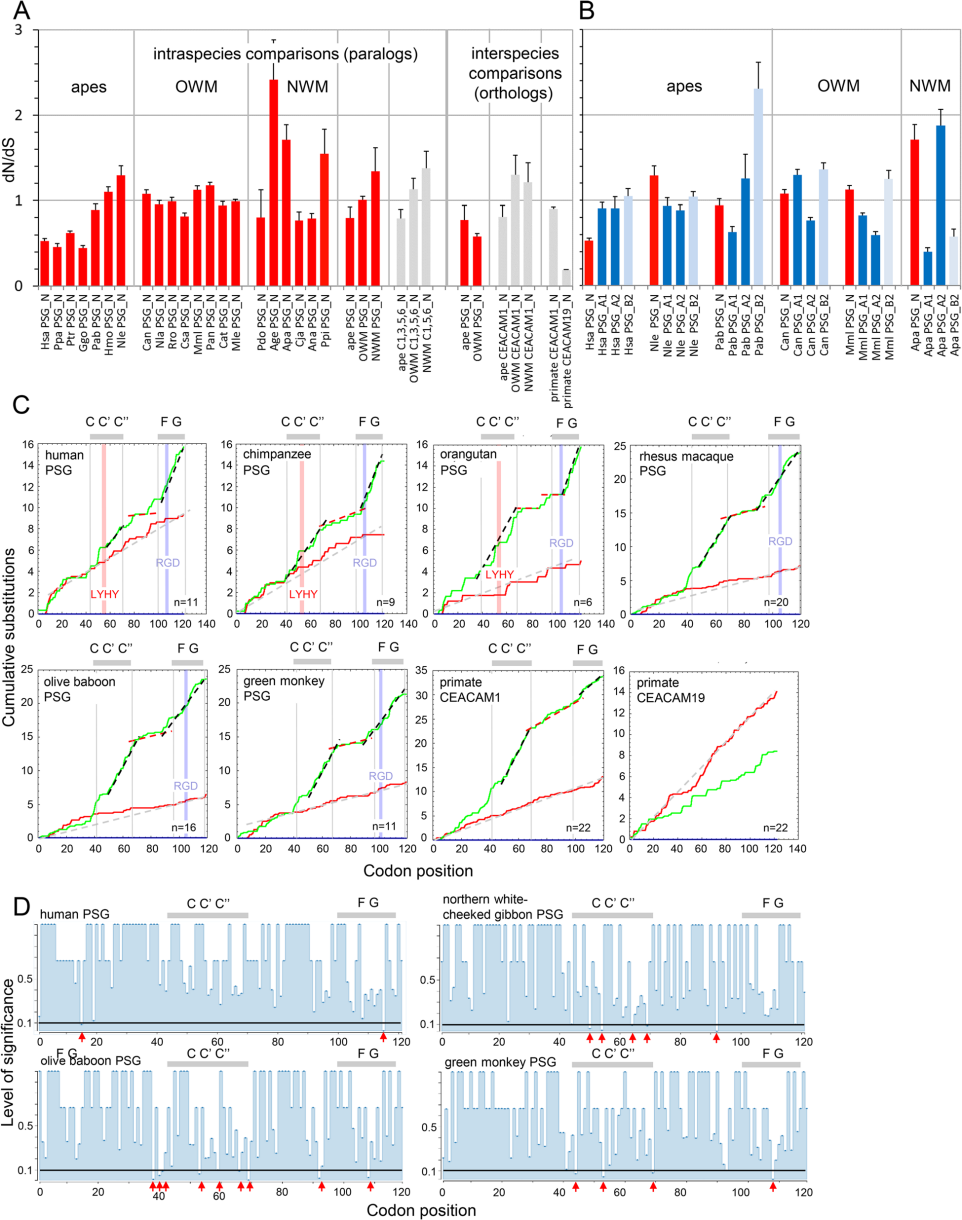
**Selection for diversification in PSG N and B2 domains. a** Nucleotide sequences of N domain exons of PSG paralogs from ape and NWM species with ≥3 PSG as well as a subset of OWM species were compared pair-wise in all combinations for each indicated species and the ratio of the rate of nonsynonymous (dN) and synonymous mutations (dS) was calculated and the mean ratios (± SEM) were plotted. In addition, the average of dN/dS means (± SEM) for the analyzed species within the ape, OWM and NWM subgroups were calculated (forth panel). For comparison, the same calculations were performed for CEACAM1-like paralogs (CEACAM1, 3, 5, 6) of the same primate subgroups (fifth panel). The means (± SEM) of N exon dN/dS ratios were also calculated for PSG ortholog pairs which could be reliably identified for closely related ape (Ggo, Hsa, Ppa, Ptr; Hmo, Nle) and OWM species (Cat, Mfa, Mle, Mml, Mne, Pan) but not for NWM by phylogenetic analyses and for comparison for CEACAM1 orthologs of the same species or all NWM species. The means of all pairwise orthologous PSG gene N exon comparisons for the above indicated species within ape (35 comparisons) and OWM subgroups (117 comparisons) are shown. In addition, the dN/dS ratios for the N domain exons of CEACAM1 and CEACAM19 orthologs of 22 primate species including lemurs and lories were calculated. **b** dN/dS calculations for selected apes, OWM and NWM using all retrievable IgC-like domain exon nucleotide sequences with open reading frames were performed as in **a** and compared to dN/dS values for the corresponding N exon sequences (mean ± SEM). **c** The cumulative frequencies of nonsynonymous (green curves) and synonymous substitutions (red curves) along the N exons of paralogous PSG from selected great apes as well as primate CEACAM1 and CEACAM19 orthologs were determined. Note the rapid accumulation of nonsynonymous mutations in the CC’C″FG β-strand regions (black broken lines) which indicates selection for diversification. This contrasts with conserved regions between CC’C″ and FG β-strands (red broken lines). The location of CC’C″ and FG β-strand regions determined by 3D modeling are indicated by gray boxes above the graphs. Note the relative steady accumulation of synonymous substitutions indicated by gray broken lines. The location of LYHY and RGD motifs are shown by red and blue lines, respectively. The number of analyzed genes is indicated in lower right corner. A1, A2, B2, IgC-like domain exons; N, N domain exon; NWM, New World monkey; OWM, Old World monkey. For assignment of acronyms to common species names refer to Supplementary Table 1. **d** Regions of positive selection within PSG N domains differ between species. Sites within N domain exons (x-axis) with episodic diversifying selection as detected by MEME (red arrows) were plotted against the *p*-value (level of significance; y-axis). The species from which the PSGs were analyzed are indicated. Note that in different species sites under positive selection differ in number and location

Assuming that the paralogous *PSG* genes were derived from a single *PSG* by repeated tandem duplication [21] in a common primate ancestor, determination of the ratio of the number of nonsynonymous substitutions per non-synonymous site (dN) and the number of synonymous substitutions per synonymous site (dS) in the N domain exons of paralogous *PSG* genes allows estimation whether overall pressure for conservation (dN/dS < 1) or selection for diversification (dN/dS > 1) exists within primate species. Gene conversion events which are common within families of closely related genes like the *PSG* genes [22] and might influence the dN/dS ratios were not considered. The three highest ratios, averaged from all pairwise PSG N exon comparisons within a species, were observed for NWM (mean ± SEM: 2.4 ± 0.5, 1.7 ± 0.2, 1.5 ± 0.3). Gibbons and 3 out of 8 analyzed OWM exhibited dN/dS ratios > 1 (between 1.1–1.3) (Fig. 6a). Human, bonobo, chimpanzee and gorilla *PSG* paralogs showed the lowest N exon dN/dS ratios (between 0.45 and 0.6). In comparison, the N exon dN/dS ratios of orthologous *CEACAM1* genes from 22 primate species is close to 1 (0.90 ± 0.02). The *CEACAM1* N exon dS/dN ratio indicates selection for diversification taking into account that some of the amino acids of the IgV-like domain have to be invariant in order to maintain the β-sheet structure. In contrast, the N exons of *CEACAM19* from the same set of primates not known to be under diversification pressure shows a 4.7-fold lower dN/dS ratio (0.19 ± 0.01 SEM; Fig. 5a). Taken together, these findings suggest that N domain exons from paralogous *PSG* from most primates, with the exception of human, chimpanzee and gorilla *PSG* N exons, are undergoing selection for diversification. Interestingly, the means of ape, OWM, NWM species paralogous *PSG* N exon dN/dS ratios are very similar to that of *CEACAM1, CEACAM3, CEACAM5* and *CEACAM6* paralogs the diversification of which is known or thought to be driven by pathogen usage of these members as entry or decoy receptors (Fig. 6a) [23, 24].

Are orthologues PSG in different species also selected for diversification? Due to rapid divergence identification of pairs of orthologous *PSG* genes is restricted to closely related great apes (human, gorilla, chimpanzee and bonobo; Northern white cheeked gibbon and silvery gibbon) and OWM (colobus monkey, macaca species, mandrill, baboon). In total, 35 and 117 orthologous *PSG* pairs could be reliably identified in 6 great ape and 6 OWM species, respectively. No orthologous *PSG* gene pairs among NWM species could be identified. For both great ape and OWM *PSG* N exons average dN/dS ratios clearly < 1 (0.78 ± 0.17 SEM and 0.58 ± 0.04 SEM, respectively) were found. N exons of *CEACAM1* genes of the same ape and OWM species exhibited similar or larger dN/dS ratios (0.74 ± 0.13 SEM and 1.3 ± 0.23 SEM, respectively) (Fig. 6a).

Generally, high dN/dS ratios were also observed for exons encoding IgC-like domains of *PSG* paralogs with the exception of A1 exons in some primates. Interestingly, in all analyzed primate species besides the mantled howler monkey, a NWM, the highest ratios were found for the exons encoding the B2 domains which have been demonstrated to be involved in TGFβ1 activation (Fig. 6b) [16].

Selection for diversification in PSGs is expected to be not evenly distributed across the N domain. Amino acids positions which are needed for the generation of the immunoglobulin fold are anticipated to be under purifying selection while regions involved in ligand binding might exhibit selection for diversification. Indeed, the regions known in other CEACAM members such as CEACAM1 (but not CEACAM19) to interact with ligands and bacterial adhesins, the CC’C″FG face of the immunoglobulin fold, with the exception of the LYHY and RGD motifs, apparently accumulate nonsynonymous substitutions with a high rate (black stippled lines) while other regions e.g. the D and E immunoglobulin β-strand regions do not (red stippled lines; Fig. 6c). In addition, we used the MEME software to confirm that individual sites of the N domains are under positive selection. Exemplarily, we analyzed PSGs from humans, gibbon, baboon and green monkey and detected 2, 5, 9, and 4 sites under episodic positive selection at a significance level of 0.1 (default and the most stringent setting of the software), respectively. In particular in the non-human primates, these sites were located in or near the CC’C″FG face which is the main ligand-binding region of CEACAMs (Fig. 6d).

## Discussion

We hypothesized that direct exposure of fetal trophoblast cells to maternal immune cells is a requirement for the presence of *PSG* genes. This hypothesis has been substantiated through this study which demonstrated the presence of *PSG* genes in 38 out of 39 primates with hemochorial placentation, tarsius the most distantly related haplorhine primate investigated being the sole exception and the absence of *PSG* genes from primates with epitheliochorial placentae lacking immune cell-trophoblast cell contact (one loris and 16 lemurs). Interestingly, PSG-like genes are only found in bats of the suborder Yangochiroptera with hemochorial placentation but not in the Yinpterochiroptera suborder despite the presence of species with hemochorial placentae in the latter [4]. Thus it appears that the presence of highly invasive trophoblast cells like in hemochorial placentae or in equine endometrial cups is necessary but not sufficient for the presence of PSG genes.

Genomic analyses reported here and elsewhere suggest that *PSG* genes were derived from the same single *PSG* gene or set of ancestral *PSG* genes present in the last common ancestor of apes, OWM and NWM which lived some 40 million years ago [20, 25]. During evolution of the *Catarrhini* (apes and OWM) and *Platyrrhini* parvorders (NWM) the *PSG* loci expanded or contracted quite differently or stayed the same leading to a single copy in capucin and Bolivian squirrel monkeys and a total of 27 copies (including *PSG* pseudogenes) in the De Brazza’s OWM. The average *PSG* family sizes (excluding pseudogenes) vary vastly: 3 PSG genes in NWM, 9 in apes and 17 in OWM. Presently it is unclear whether expansion of *PSG* gene families is driven by the need for large quantities of PSG protein with similar function for example to block large amounts of fibrinogen in the maternal blood to attenuate coagulation during pregnancy or by the requirement for PSGs with diversified functions [10]. The former seems to be supported by the fact that several PSGs in one species exhibit the same function (PSG1, PSG9 inhibit platelet-fibrinogen interaction in humans; PSG17, PSG22, PSG23 bind to heparin sulfate in mouse) [10, 26]. Copy number variations are common in the human *PSG* locus leading to 11–30 PSG members in normal individuals [25]. Thus higher *PSG* dosages might not only be tolerated but could confer an adaptive advantage through elevated PSG levels.

However, there are also indications that various PSG members within a species can differ in their function. For example, both murine PSG17 and PSG19 but not PSG23 bind to the tetraspanin receptor CD9 [27]. Further support for a different function of individual PSGs is derived from the observation that in primates PSG mRNA levels differ tremendously. For example, human *PSG1* transcripts are nearly 200-fold more frequently found in placental EST libraries than *PSG7* transcripts. Furthermore, the tetra-peptide sequence motif LYHY which is required for the induction of latent TGFβ1 secretion is absent from the highly expressed human PSG4 and from most or all PSGs of OWM and NWM. In addition, the putative disintegrin-like RGD motif although more common than the LYHY motif is missing from highly expressed PSGs. However, relaxed sequence motif requirements i.e. tolerance of conservative amino acid changes in the motifs have to be kept in mind before final conclusions can be drawn. *PSG11* gene copy-number loss and increased PSG9 levels are associated with a higher risk for preeclampsia, a serious pregnancy complication characterized by high blood pressure and proteinuria, also indicate different roles of PSGs in the establishment and maintenance of successful pregnancies [28–30]. However, these data have to be substantiated in more patient samples.

PSGs which have independently originated in distantly related mammals share multiple features possibly due to convergent evolution in primates, rodents, horse and by inference in bats (where formal proof of placental expression is still lacking [4]). In this line TGFβ1 activation as well as inhibition of platelet aggregation through fibrinogen by human, murine and equine PSGs are common functions of PSGs [3, 6, 17]. This hints towards evolutionary pressure for generation of molecules with similar functions in mammals with hemochorial placentation and/or with invasive trophoblast cells [5]. Such multifunctional genes tend to undergo subfunctionalization after gene duplication [31]. Thus some paralogs may be optimized for one of the functions of the ancestral gene. Such optimization processes may lead to positive selection and diversification of paralogs followed by purifying selection once a new function has been established. The latter is most evident in orthologous *PSG* in OWM.

However, a second functional layer seems to exist in PSGs. As pointed out before, strong selection for diversification as indicated by an excess of nonsynonymous substitutions in PSG genes [25] and an dN/dS ratio of greater than 1 in N exons of PSG in a number of primates has been shown here. This has also been observed for CEACAM1 in humans and mice and it has been suggested to serve as bacterial, fungal and viral pathogen receptor also in other vertebrate species [8, 32–37]. Interestingly, a similar selection for diversification has been noted for proven (human CEACAM3) and suspected decoy pathogen receptors (human CEACAM5 and CEACAM6), which function as phagocytic receptors in granulocytes and as a possible pathogen sink in the intestine, respectively [23, 24, 38]. However, despite the pathogen-host arms race leading to rapid divergence of host pathogen receptors, decoy receptors have to be kept similar e.g. by gene conversion as found for CEACAM1/CEACAM3 [22]. Therefore, we speculate that PSG might act, in addition to their conserved functions, as decoy receptors for pathogens which imperil pregnancies. Interaction of PSGs with pathogens is also suggested by the rapid accumulation of nonsynonymous substitutions encoding the CC’C″FG β-sheet of the N domain which is targeted by pathogen adhesins in CEACAM pathogen receptors [39]. Different degrees of positive selection for diversification (low in human, bonobo, chimpanzee and gorilla; high in gibbons, some OWM and NWM) and highly variant numbers of *PSG* in various primate species could indicate episodic challenges by pathogens. Pronounced gene family size differences in closely related mouse species have also been observed for a group of eosinophil-associated RNases suspected to exhibit bacterial membrane disruptive properties [40]. When pathogen pressure ceases, contraction of gene numbers and homogenization of sequences could occur e.g. by gene conversion as noted for the human *PSG* locus [22].

## Conclusions

The presence of trophoblast-specific immune-modulating PSGs in all but one primates with hemochorial but not in primates with epitheliochorial placentae supports the notion that close contact of fetal cells with the maternal immune system as seen in hemochorial placentation favor the evolution and expansion of PSGs. Furthermore, phylogenetic analyses revealed selection for sequence diversity of functional domains but also conservation of orthologous PSGs mainly in OWM indicating ongoing functional diversification and stabilization of newly acquired functions. The large number of PSG sequences provided here might serve as a basis for the identification of functional PSG subgroups and delineation of functional sequence motifs in the future.

## Methods

### Identification and nomenclature of genes

Nucleotide sequence searches were performed using the NCBI BLAST/BLAT tools (http://www.ncbi.nlm.nih.gov/BLAST) and the Ensembl database, the UCSC Genome Browser (http://www.ensembl.org/Multi/Tools/Blast?db=core; https://genome.ucsc.edu/cgi-bin/hgGateway) as well as WGS contig databases using default parameters. The databases used are listed in Supplementary Table 1. For identification of *PSG* genes regions syntenic to human *PSG* loci were analyzed for the presence of CEACAM Ig domain-encoding exons. Primate genomes were reprobed with exon sequences from newly identified *PSG* genes. Although most of the genomes had been sequenced in great depth (see genome coverage in Supplementary Table 1) the number of PSG genes might increase with further genome refinement. PSG genes were numbered arbitrarily except for ape *PSG* genes for which assignment to orthologous human genes was possible. Genes that contained stop codons within their N domain exons or lacked appropriate splice acceptor and donor sites in these exons were considered to represent pseudogenes. Nucleotide sequences from the N domain exons can be used as gene identifiers (Supplementary File 1). The same strategy was employed to identify other genes of the CEACAM families. CEACAM/PSG genes, the N exons of which exhibited > 99% nucleotide sequence identity, were considered to represent alleles.

### Quantification of *PSG* expression by EST frequency determination

The relative expression frequencies of PSG were estimated by counting the clones matching N exon nucleotide sequences of the different PSG genes present in human, baboon (*Pan anubis*) and rhesus monkey (*Macaca mulatta*) placental EST libraries using the NCBI BLAST program as above. Only hits with E val = 0.0 were assigned to a given gene.

### Sequence motif identification and 3D modeling

The presence of immunoreceptor tyrosine-based activation motifs (ITAM), ITAM-like, and immunoreceptor tyrosine-based inhibition motifs (ITIM) and immunoreceptor tyrosine-based switch motifs (ITSM) were confirmed using the amino acid sequence pattern search program ELM (http://elm.eu.org/). Transmembrane regions, glycosylphosphatidylinositol (GPI) signal domains and leader peptide sequences were identified using the TMHMM (http://www.cbs.dtu.dk/services/TMHMM-2.0/), the big-PI predictor (http://mendel.imp.ac.at/sat/gpi/gpi_server.html), GPI-SOM (http://gpi.unibe.ch/) and the SignalP 4.1 programs (http://www.cbs.dtu.dk/services/SignalP/), respectively [41]. The three-dimensional structure of IgV-like domains for the localization of β-strands was modeled using the I-TASSER server (https://zhanglab.ccmb.med.umich.edu/I-TASSER/) with default settings [42].

### Phylogenetic analyses and determination of positive selection

Phylogenetic analyses based on nucleotide sequences were conducted using MEGA7 [43]. Sequence alignments were performed using muscle (https://www.ebi.ac.uk/Tools/msa/muscle/). Phylogenetic trees were constructed using the unweighted pair group method with arithmetic mean (UPGMA) or the maximum likelihood (ML) method with bootstrap testing (500 replicates). Multiple nucleotide sequence alignments were performed with ClustalW programs (http://npsa-pbil.ibcp.fr/cgi-bin/npsa_automat.pl?page=/NPSA/npsa_clustalw.html; http://www.genome.jp/tools/clustalw/). In order to determine the selective pressure on the maintenance of the nucleotide sequences, the number of nonsynonymous nucleotide substitution per nonsynonymous site (dN) and the number of synonymous nucleotide substitutions per synonymous site (dS) were determined for *PSG* and *CEACAM* N domain and IgC-like exons. The dN/dS ratios between pairs of *PSG* orthologs and paralogs and orthologous *CEACAM* genes as well as the cumulative synonymous and nonsynonymous substitutions along coding regions of N domain exons from paralogous PSG and orthologous CEACAM genes were calculated after manual editing of sequence gaps or insertions guided by the amino acid sequences using the SNAP program (Synonymous Nonsynonymous Analysis Program; http://www.hiv.lanl.gov/content/sequence/SNAP/SNAP.html) [44]. For the detection of individual sites under positive selection we used the mixed effects model of evolution software (MEME) [45].

## Supplementary Information

**Additional file 1: Supplementary Figure 1. Evolutionary relationship of the primate species of this study.** The Maximum Likelihood method (MEGA6 software) was used to construct the phylogenetic trees based on concatenated nucleotide sequences of exons coding for extracellular domains of conserved CEACAMs i.e. CEACAM16 N1 and N2, and CEACAM19 N from 56 primate and three non-primate species (two shrews, one flying lemur). The Northern tree shrew and the Ugandan red colobus were not included due to incompleteness of their retrieved CEACAM16N1 and CEACAM19 N exon sequences, respectively. The tree with the highest log likelihood is shown. The percentage of trees in which the nucleotide sequences clustered together is shown next to the branches. The branching point which leads to PSG-positive primates is indicated. The number of PSG genes with N exon open reading frames is indicated in red. Primate suborders (Haplorhini, Strepsirrhini) and OWM subfamilies (Colobinae, Cercopithecinae) and type of placentation is indicated in the right margin. The scale below the dendrogram shows the number of substitutions per site. NWM, New World monkeys; OWM, Old World monkeys; PSG, pregnancy-specific glycoprotein.

**Additional file 2: Supplementary Figure 2. Latent TGFβ1 secretion and putative disintegrin motifs in primate PSGs.** (A) Amino acid sequences (one letter code) of mature N domains from ape, OWM and NWM species were aligned. Amino acids conserved in all PSG in a given species are shown in red, positions with conserved amino acid changes are shown in green, less conserved positions in blue. Non-conservative changes are shown in black. The LYHY motif shown to be responsible for latent TGFβ1 secretion is highlighted with filled-in red boxes, the putative disintegrin motifs with filled-in blue boxes. Disintegrin-like motifs with conservative amino acid changes are marked by blue open boxes. For the long form of the abbreviated Latin species names see Supplementary Table 1. (B) The fraction of PSGs with latent TGFβ1 secretion and disintegrin-like R/KGD/E motifs was calculated for each primate species and the means (± SEM) were plotted for apes, OWM and NWM. NWM, New World monkeys; OWM, Old World monkeys; PSG, pregnancy-specific glycoprotein; TGFβ1, tumor growth factor β1.

**Additional file 3: Supplementary Figure 3. IgC-type exon inclusion in human, rhesus and howler monkey PSG mRNAs.** (A) a schematic exon organization of human, rhesus monkey and howler monkey PSG genes is shown. Two pairs of exons encoding IgC-like A- and B-type domains are present in primate PSG genes. Most of A1 and B2 exons contain intact consensus splice sites and open reading frames in transcribed human and rhesus monkey PSG genes. Due to the lack of PSG transcription information in howler monkey all exons of the Apa_PSG genes were analyzed. In contrast to A1 and B2 exons, only 1 out of 10, 7 out of 20, 2 out of 8 B1 exons in human, rhesus monkey and howler monkey PSG genes, respectively, exhibit both intact consensus splice sites and open reading frames. In rhesus monkey, only 4 out of 20 PSG contain intact A2 exons. However, these exons are not (B1 exons in human PSG) or rarely spliced-in (B1, in 2 out of 13, A2 in 4 out of 13 transcribed rhesus monkey PSG). (B) All human and rhesus monkey PSG transcripts encode functionally important N and B2 domains, conveying TGFβ1 secretion and TGFβ1 activation, respectively, while variably 1 or 2 but never 3 IgC-like domains seem to serve as “spacers” indicated by brackets. For howler monkey the domain organization of the expected largest PSG is shown. Apa, *Alouatta palliata*, howler monkey; Hsa, *Homo sapiens*, human; Mml, *Macaca mulatta*, rhesus macaque; NWM, New World monkey; ORF, open reading frame; OWM, Old World monkey; ss, splice site.

**Additional file 4: Supplementary Figure 4. Loss of orthologous relationship during ape, OWM and NWM PSG evolution.** Phylogenetic trees were constructed based on N domain exons nucleotide sequences of PSG genes from great ape (A), OWM (B) and NWM (C) species using the Maximum Likelihood method (MEGA6 software). The trees with the highest log likelihood are shown. The percentage of trees in which the nucleotide sequences clustered together is shown next to the branches. Primate families/subfamilies and species can be identified by colored branches and colored symbols, respectively, shown next to the phylogenetic trees which were generated as described in Supplementary Figure 1. (A) Most of the human, bonobo, chimpanzee and gorilla (Homininae) PSG genes form orthologous clusters while only a few PSG genes within the great ape family exhibit an orthologous relationship (marked by gray trapezoids). Part of orangutan and most gibbon PSG genes cluster in a paralogous manner. (B) In OWM, PSG genes cluster according to the Colobinae (blue) and Cercopithecinae subfamilies (red colors). (C) With one possible exception (tufted capuchin, Sapajus capella; white-fronted capuchin, *Cebus albifrons*) NWM PSG genes form paralogous clusters. NWM, New World monkeys; OWM, Old World monkeys; PSG, pregnancy-specific glycoprotein. For common species names refer to Supplementary Table 1.

**Additional file 5: Supplementary File 1** N exon nucleotide sequences of primate CEACAM genes. Contains nucleotide sequences of N domain exons and accession numbers of PSG genes of all primates analyzed. Of note: identical numbers in PSG gene names in different primates does not imply an orthologous relationship.

**Additional file 6: Supplementary Table 1.** CEACAM1-like genes in primates. This table lists the common names of primate species, their abbreviation, Latin name, taxonomic classification, genomic data source and the number and types of CEACAM1-related genes and pseudogenes.

## Abbreviations

CEA: Carcinoembryonic antigen
CEACAM: CEA-related cell-cell adhesion molecule;
dN: Number of non-synonymous substitutions per non-synonymous site
dS: Number of synonymous substitutions per synonymous site
EST: Expressed sequence tag
GPI: Glycosylphosphatidylinositol
Ig: Immunoglobulin
IgC: Immunoglobulin constant
IgV: Immunoglobulin variable
ITAM: Immunoreceptor tyrosine-based activation motif
ITIM: Immunoreceptor tyrosine-based inhibition motif
ITSM: Immunoreceptor tyrosine-based switch motif
K/RGD/E: Lysine/arginine-glycine-aspartic acid/glutamic acid
LYHY: Leucine-tyrosine-histidine-tyrosine
MEME: Mixed effects model of evolution
ML: Maximum likelihood
N: N-terminal
NWM: New World monkey
OWM: Old World monkey
PSG: Pregnancy-specific glycoprotein
RGD: Arginine-glycine-aspartic acid
SEM: Standard error of the mean
TGFß1: transforming growth factor ß1
UPGMA: Unweighted pair group with arithmetic mean
WGS: Whole genome shot-gun
