# Supplementary material for: The immune-modulating pregnancy-specific glycoproteins evolve rapidly and their presence correlates with hemochorial placentation in primates
Source: BMC Genomics. 2021 Feb 18;22:128. doi: 10.1186/s12864-021-07413-8 (PMC7893922; doi:10.1186/s12864-021-07413-8)
Supplement: Supplementary file 1 — Additional file 1: Supplementary Figure 1. Evolutionary relationship of the primate species of this study. The Maximum Likelihood method (MEGA6 software) was used to construct the phylogenetic trees based on concatenated nucleotide sequences of exons coding for extracellular domains of conserved CEACAMs i.e. CEACAM16 N1 and N2, and CEACAM19 N from 56 primate and three non-primate species (two shrews, one flying lemur). The Northern tree shrew and the Ugandan red colobus were not included due to incompleteness of their retrieved CEACAM16N1 and CEACAM19 N exon sequences, respectively. The tree with the highest log likelihood is shown. The percentage of trees in which the nucleotide sequences clustered together is shown next to the branches. The branching point which leads to PSG-positive primates is indicated. The number of PSG genes with N exon open reading frames is indicated in red. Primate suborders (Haplorhini, Strepsirrhini) and OWM subfamilies (Colobinae, Cercopithecinae) and type of placentation is indicated in the right margin. The scale below the dendrogram shows the number of substitutions per site. NWM, New World monkeys; OWM, Old World monkeys; PSG, pregnancy-specific glycoprotein. [file 12864_2021_7413_MOESM1_ESM.pptx]

## Slide 1
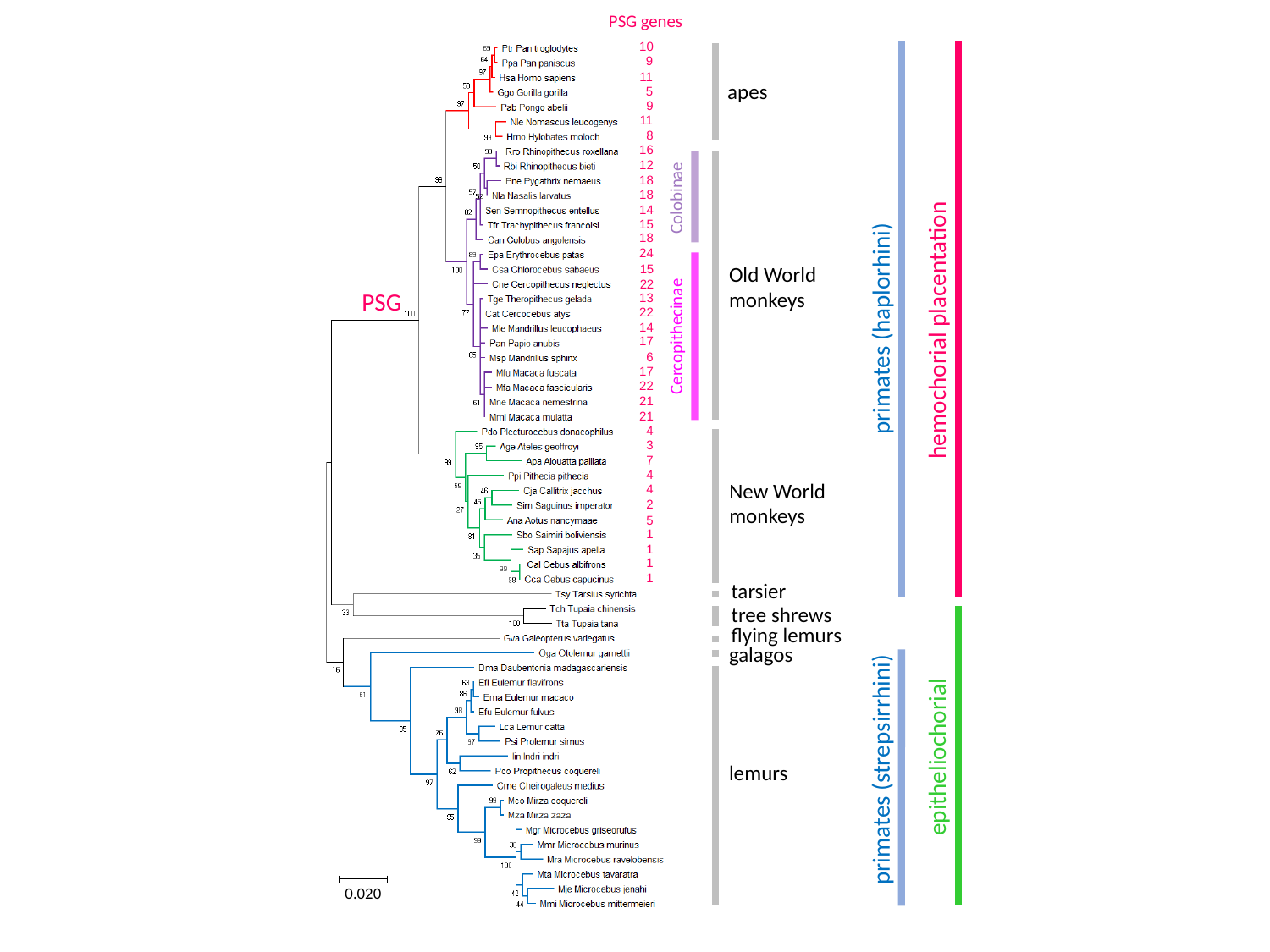

PSG genes
10
9
11
apes
5
9
11
8
16
12
18
Colobinae
18
14
15
18
24
Old World
monkeys
15
22
PSG
13
22
primates (haplorhini)
hemochorial placentation
14
Cercopithecinae
17
6
17
22
21
21
4
3
7
4
New World
monkeys
4
2
5
1
1
1
1
tarsier
tree shrews
flying lemurs
galagos
epitheliochorial
primates (strepsirrhini)
lemurs
0.020
