# Supplementary material for: The immune-modulating pregnancy-specific glycoproteins evolve rapidly and their presence correlates with hemochorial placentation in primates
Source: BMC Genomics. 2021 Feb 18;22:128. doi: 10.1186/s12864-021-07413-8 (PMC7893922; doi:10.1186/s12864-021-07413-8)

**A**

**Apes** latent TGFβ1 secretion motif disintegrin-like motif

**Bonobo** 10 20 30 40 50 60 70 80 90 100

| | | | | | | | | |

Ppa_PSG1N QVTIEAQPPKVSKGKDVLLLVHNLPQNLTGYIWYKGQMRDLYHYITSYVVDGQRIIYGPAYSGRETVYSNASLLIQNVTWEDAGSYTLHIIKGGDETRGVTGRFTFTLY

Ppa_PSG8N QVTIEAQPTKVSEGKDVLLLIHNLPQNLTGYIWYKGQMRDLYHYITSYVVDGQRIIYGPAYSGRETIYSNASLLIQNVTREDAGSYTLHIIKGGDETRGVTGHFTFTLY

Ppa_PSG2N QVTIEARPPKVSEGKDVLLLVHNLPQNLTGYIWYKGQIRDLYHYITSYVVDGQIIIYGPAYSGRETVYSNASLLIQNVTREDAGSYTLHIIKRGDGTRGITGNFTFTLY

Ppa_PSG11N QVMIEAQPPKVSEGKDVLLLVHNLPQNLTGYIWYKGQIRDLYHYITSYVVDGQIIIYGPAYSGRETVYSNASLRIQNVTREDAGSYTLHIIKRGDGTRGITGNFTFTLY

Ppa_PSG10N QVTIEAQPPKVSEGKDVLLLVHNLPQNLTGYMWYKGQIRDLYHYITSYVVDGQRITYGPTYSGRETVYSNASLLIQNVTWEDAGSYTLHIIQRGDGTRGVTGNFTFTLY

Ppa_PSG6N QVIIEAQPPKVSEGKDVLLLVHNLPQNLTGYIWYKGQMRDLYHYITSYVVDGQXIIYGPAYSGRETVYSNASLLIQNVTQEDAGSYTLHIIKRGDGTGGVTGHFTFTLY

Ppa_PSG7N QVTIEAQPPKVSEGKDVLLLVHNLPQNLTGYIWYKGQIRDLYHYVTSYVVDGQIIIYGPAYSGRETVYSNASLLIQNVTREDAGSYTLHIIKRGDGTGGVTGHFTFTLY

Ppa_PSG4N QVTIEALPPKVSEGKDVLLLVHNLPQNLAGYIWYKGQMTYLYHYITSYVVDSQRIIYGPAYSGRETVYSNASLLIQNVTWEDAGSYTLHIIKRGDGTGGVTGHFTFTLY

Ppa_PSG3N QVTIEAQPTKVSKGKDVILLVHNLPQNLAGYIWYKGQMTDLYHYITSYVVDGQIIIYGPAYSGRETVYSNASLLIQNVTREDAGSYTLHIVKRGDGTRGITGHFTFTLY

Ppa_PSG5N QVTIEALPPKVSEGKDVLLLVHNLPQNLAGYIWYKGQLMDLYHYITSYVVDGQINVYGPAYTGRETVYSNASLLIQNVTREDAGSYTLHIIKRGDRTRGVTGYFTFNLY

Ppa_PSG9N EVTIEAQPPKVSEGKDVLLLVHNLPQNLPGYFWYKGQLTDLYHYIISYIVDGKIIIYGPAYSGRETVYSNASLLIQNVTREDAGTYTLHIIKRSDETREEIRYFTVTLY

**Chimpanzee**  10 20 30 40 50 60 70 80 90 100

| | | | | | | | | |

Ptr_PSG2N QVTIEAQPPKVSEGKDVLLLVHNLPQNLTGYIWYKGQIRDLYHYITSYVVDGQIIIYGPAYSGRETVYSNASLLIQNVTREDAGSYTLHITKRGDGTRGITGNFTFTLY

Ptr_PSG11N QVMIEAQPPKVSEGKDVLLLVHNLPQNLTGYIWYKGQIRDLYHYITSYVVDGQIIIYGPAYSGRETVYSNASLRIQNVTREDAGSYTLHIIKRGDGTRGITGNFTFTLY

Ptr_PSG10N QVTIEAQPPKVSEGKDVLLLVHNLPQNLTGYMWYKGQIRDLYHYITSYVVDGQRITYGPTYSGRETVYSNASLLIQNVTREDAGSYTLHIIQRGDGTRGVTGNFTFTLY

Ptr_PSG1N QVTIEAQPPKVSKGKDVLLLVHNLPQNLTGYIWYKGQMRDLYHYITSYVVDGQIIIYGPAYSGRETVYSNASLLIQNVTWEDAGSYTLHIIKGGDETRGVTGRFTFTLY

Ptr_PSG8N QVTIEAQPTKVSEGKDVLLLIHNLPQNLTGYIWYKGQMRDLYHYITSYVVDSQRIIYGPAYSGRETIYSNASLLIQNVTREDAGSYTLHIIKGGDETRGVTGHFTFTLY

Ptr_PSG4N QVTIEALPPKVSEGKDVLLLVHNLPQNLAGYIWYKGQMTYLYHYITSYVVDSQRIIYGPAYSGRETVYSNASLLIQNVTWEDAGSYTLHIIKRGDGTGGVTGHFTFTLY

Ptr_PSG3N QVTIEAQPTKVSKGKDVLLLVHNLPQNLAGYIWYKGQMTDLYHYITSYVVDGQIIIYGPAYSGRETVYSNASLLIQNVTREDAGSYTLHIVKRGDGTRGITGHFTFTLY

Ptr_PSG5N QVTIEALPPKVSEGKDVLLLVHNLPQNLAGYIWYKGQLMDLYHYITSYVVDGQINIYGPAYTGRETVYSNASLLIQNVTREDAGSYTLHIIKRGDRTRGVTGYFTFNLY

Ptr_PSG9N EVTIEAQPPKVSEGKDVLLLVHNLPQNLPGYFWYKGEITDLYHYIISYIVDGKIIIYGPAYSGRETVYSNASLLIQNVTREDAGTYTLHIIKRSDETREEIRYFSFTLY

**Gorilla** 10 20 30 40 50 60 70 80 90 100

| | | | | | | | | |

Ggo_PSG7N QVTIEAQPPKVSEGKDVLLLVHNLPQNLTGYIWYKGQIRDLYHYVTSYVVDGQIIIYGPAYSGRETVYSNASLLIQNVTWEDAGSYTLHIIKRGDGTGGETGNFTFTLY

Ggo_PSG11N QVTIEAQPPKVSEGKDVLLLVHNLPQNLTGYIWYKGQIRDLYHYITSYVVDGQIIIYGPAYSGRETVYSNASLLIQNVTREDAGSYTLHIIKRGDGTGGVTGNFTFTLY

Ggo_PSG3N QVTIEAEPTKVSKGKDVLLLVHNLPQNLAGYIWYKGQMTDLYHYITSYVVDGQIIIYGPAYSGRETVYSNASLLIQNVTREDAGSYTLHIVKRGDGTRGETGNFTFTLY

Ggo_PSG4N QVTIEAQPPKVSEGKDVLLLVHNLPQNLAGYIWYKGQMTYLYHYITSYAVDGQRIIYGPAYSGRETVYSNASLLIQNVTWEDAGSYTLHIIKRGDGTGGVTGHFTFTLY

Ggo_PSG5N QVTIEAQPPKVSEGKDVLLLVHNLPQNLAGYIWYKGQLMDLYHYITSYVVDGQINIYGPAYSGRETVYSNASLLIQNVTQEDAGSYTLHIIKRGDRTRGVTGHFTFNLY

**Human**  10 20 30 40 50 60 70 80 90 100

| | | | | | | | | |

Hsa_PSG1N QVTIEAQPPKVSEGKDVLLLVHNLPQNLTGYIWYKGQMRDLYHYITSYVVDGEIIIYGPAYSGRETAYSNASLLIQNVTREDAGSYTLHIIKGDDGTRGVTGRFTFTLH

Hsa_PSG8N QVTIEAQPTKVSEGKDVLLLVHNLPQNLTGYIWYKGQIRDLYHYITSYVVDGQIIIYGPAYSGRETIYSNASLLIQNVTQEDAGSYTLHIIMGGDENRGVTGHFTFTLY

Hsa_PSG7N QVTIEAQPPKVSEGKDVLLLVHNLPQNLTGYIWYKGQIRDLYHYVTSYVVDGQIIKYGPAYSGRETVYSNASLLIQNVTQEDTGSYTLHIIKRGDGTGGVTGRFTFTLY

Hsa_PSG10N QVTTEAQPPKVSEGKDVLLLVHNLPQNLTGYMWYKGQIRDLYHYITSYVVDGQIITYGPAYSGRETVYPNASLLIQNVTREDGVSYTLHIIQRGDGTRGVTGNFTFTLY

Hsa_PSG2N QVTIEAQPPKVSEGKDVLLLVHNLPQNLTGYIWYKGQIRDLYHYITSYVVDGQIIIYGPAYSGRETAYSNASLLIQNVTREDAGSYTLHIIKRGDGTRGVTGYFTFTLY

Hsa_PSG11N QVMIEAQPPKVSEGKDVLLLVHNLPQNLTGYIWYKGQIRDLYHYITSYVVDGQIIIYGPAYSGRETVYSNASLLIQNVTREDAGSYTLHIIKRGDGTRGVTGYFTFTLY

Hsa_PSG4N QVTIEAQPPKVSEGKDVLLLVHNLPQNLAGYIWYKGQMTYVYHYITSYVVDGQRIIYGPAYSGRERVYSNASLLIQNVTQEDAGSYTLHIIKRRDGTGGVTGHFTFTLH

Hsa_PSG6N QVIIEAKPPKVSEGKDVLLLVHNLPQNLTGYIWYKGQMTDLYHYITSYVVHGQ-IIYGPAYSGRETVYSNASLLIQNVTQEDAGSYTLHIIKRGDGTGGVTGYFTVTLY

Hsa_PSG5N QVTIEALPPKVSEGKDVLLLVHNLPQNLAGYIWYKGQLMDLYHYITSYVVDGQINIYGPAYTGRETVYSNASLLIQNVTREDAGSYTLHIIKRGDRTRGVTGYFTFNLY

Hsa_PSG3N QVTIEAEPTKVSKGKDVLLLVHNLPQNLAGYIWYKGQMKDLYHYITSYVVDGQIIIYGPAYSGRETVYSNASLLIQNVTREDAGSYTLHIVKRGDGTRGETGHFTFTLY

Hsa_PSG9N EVTIEAQPPKVSEGKDVLLLVHNLPQNLPGYFWYKGEMTDLYHYIISYIVDGKIIIYGPAYSGRETVYSNASLLIQNVTRKDAGTYTLHIIKRGDETREEIRHFTFTLY

**Orangutan** 10 20 30 40 50 60 70 80 90 100

| | | | | | | | | |

Pab_PSG3N QVTIEAQPPKVSEGKDVLLLVHNLPQNLAGYIWYKGQTRDLNHYITSYVADSKIIIHGPAHSGRETVYSNASLLIQNVTREDAGSYTLHIIKRGDGIRGITGHFTFTLY

Pab_PSG6N QVTIEAQPPKVSEGKDVLLLVHNLPKNLTGYIWYKGQMRDLYHYITSYVVDGQTIIYGPAYSGRETVYSNASLLIQNVTRDDAGSYTLHIIKRGDGTRGITGHFTFTLY

Pab_PSG1N QVTIEAQPPKVFEVKDVLLLVHNLPQNLAGYIWYKGQMTDLYHYITSYVVDGERIIYGPAYSGRETVYSNASLLIQNVTREDAGSYTLHIIKRGDGTRGETGHFSVNLY

Pab_PSG4N QVTIEAQPPKVSAGKDVLLLVHNLPQNLAGYIWYKGQIMDLYHYITSYVVDGQTIIYGPAYSGRETVYSNASLLIQNVTREDAGSYTLHIIKRGDRTRGVTGYFTFTLY

Pab_PSG5N QVTIEAQPPKVSAGKDVLLLVHNLPQNLAGYIWYKGQIMDLYHYITSYVVDGQTIIYGPAYSGRETVYSNASLLIQNVTREDAGSYTLHIIKRGDRTRGVTGYFTFTLY

Pab_PSG2N QVMIEAQPPKVSEGKDVLLLVHSLPQNLTGYIWYKGQMKDLYHYITSYEVDGQIIIHGPAYSGRETVYSNASLLIQNVTREDAGSYTLHIIKRGDGTRRVTGNFTFTFC

Pab_PSG7N QVMIEAQPPKVSEVKDVLLLVHNLPQNLAGYIWYKRQMMDLYHYITSYVVDGQIIIYGPAYSGRETVYSNASLLIHNVTGEDAVSYTLHIIKRGDGTRRVTGNFTFTLY

Pab_PSG8N QVTIEAQPPKVSEGKDVLLLVHNLPQNLTGYIWYKRKMTDLYHYITSYVVDGQIIIYGPAHSGRETVYSNASLLIQNVTREDAGSYTLHIIKGGDGTRGVTGNFTFTLY

Pab_PSG10N QVTIEAQPPKVSEGKDVLLLVHNLPQNLTGYIWYKRQMKDLYHYITSYVVDGQIIIYGPAYSGRETVYSNASLLIQNVTREDAGSYTLHIIKGGDGTRRETGNFTFTLY

**Silvery gibbon** 10 20 30 40 50 60 70 80 90 100

| | | | | | | | | |

Hmo_PSG7N QVMIEAQPPKISEGKDVLLLVHNLPQNLAGYMWYKGQMTDLYHYIISYIADSQTIIPGPAYSGRETVYSNASLLIQKVTREDAGSYNLHIIKRGDETTGITGHFTVTLY

Hmo_PSG8N QVMIEAQPPKISEGKDVLLLVHNLPQNLPGYMWYKGKMKDLYHYIVSYVADSQIIIPGPAYSGREIVYSNASLLIKNVTREDAGSYNLHIIKRGDETTGVTGHFTVTLY

Hmo_PSG4N QVTIEAQPPKISEGKDVLLHVHNLPQNLTGYMWYRGQMTDLYHYIVSYVVDNDIIISGPAYTERETVYSNASLLIQNVTREDAGSYTLHIIKRGDDTTGITGHFTATLY

Hmo_PSG2N QVTIEAQPPKLSEGKDVLLLVHNLPQNLTGYTWYKGQMTDLYHYITSYVVDNDIIISGPAYTGRETVYSNASLLIQNVTWEDTGPYTLHIIKQGDETRGATGHFTITLY

Hmo_PSG6N QVTIEAQPPKLSEGKDVLLLVHNLPQNLTGYTWYKGQMMDLYHYITSYVVDNDIIISGPAYTGRETVYSNASLLIQNVTREDTGPYTLHIIKRGDETRGATGNFTVTLY

Hmo_PSG1N QVTIEAQPPKVSEGKDVLLLVHNLPQNLAAYIWYEGQMMDIHHYITSYVVDDQTIVYGPAYSGRETVYSNASLLIQNVTGEDAGSYTLQIIKRGDGIRGATGHFTFTLY

Hmo_PSG3N QVTIEAHPPKLSEGKDVLLLVHNLPKNLAGYIWYKGQMTDLQHYITSYLVHNHKIIPGPEYTGRETVYSNASLLIEDVTREDAGSYTLQIIKPGDGIRGATGHFTVTLY

**White-cheeked gibbon** 10 20 30 40 50 60 70 80 90 100

| | | | | | | | | |

Nle_PSG3N QVTIEAQPPKLSEGKDVLLLVHNLPQNLTGYTWYKGQMTDLYHYITSYVVDNDIIISGPAYTGRETVYSNASLLIQNVTWEDTGPYTLHIIKRGDETREATGNFTITLY

Nle_PSG10N QVTIEAQPPKLSKGKDVLLLVHNLPQNLTGYTWYKGQMMDLYHYITSYVVDNDIIISGPAYTGRETVYSNASLLIQNVTQEDTGPYTLHIIKRGDETREATGNFTITLY

Nle_PSG2N QVTIEAQPPKLSEGKDVLLLVHNLPQNLTGYTWYKGQMTDLYHYITSYVVDNDIIISGPAYTGRETVYSNASLLIQNVTQEDTGPYTLHIIKRGDETREATGNFTVTLY

Nle_PSG6N QVMIEAQPPKISEGKDVLLHVHNLPQNLTGYIWYKGQMTDHYHYITSYVVDNQIIISGPAYTGRETVYSNASLLIQNVTQEDTGSYTLHIIKRGDETTGVTGHFTVTLH

Nle_PSG11N QVTIEAQPPKLSEGKDVLLHVHNLPQNLTGYIWYKGQMTDHYHYITSYVVDNQIIISGPAYTGRETVYSNASLLIQNVTREDTGSYTLHIIKRGDETTGVTGHFTVTLH

Nle_PSG8N QVMIEAQPPKISEGKDVLLFVHNLPQNLSGYIWYRGQATDIYHYIASYVVDNDIIIYGPAYTERKTVYSNASLLIQNVTREDAGSYTLHIIKRGDETTGVTGHFTVTLY

Nle_PSG1N QVTIEAQPPKLSEGKDVLLLVHNLPQNLAAYIWYEGQMMDIHHYITSYVVDDQTIVYGPAYSGRETVYSNASLLIQNVTGEDAGSYTLQIIKRGDGIRGATGHFTFTLY

Nle_PSG7N QVMIEAQPPKVSEGKDVLLLVHNLPHNLASYSWHKGRMMDLQHYITSYVVASQIILSGPAYSGRETLYSNASLLIQNVTREDAGSYTLNIRQRGDGIRGVTGHFTFTLY

Nle_PSG4N QVTIEAQPPKLSEGKDILLLVHNLPKNLAGYIWYKGQMTDLQHYITSYLVHNHKVIPGPEYTGRETVYCNASLLIEDVTREDAGSYTLQIIKRGDGIRGATGHFTVTLY

Nle_PSG9N QVMIEAQPPKISEGKDVLLLVHNLPQNLAGYMWYKGQMTDLYHYIISYIADSQTIIPGPAYSGRETVYSNASLLIQKVTREDAGSYNLHIIKRGDETTGVTGHFTVTLY

Nle_PSG5N QVTIEAQPPKISEGKDVLLLVHNLPQNLDGYFWYKGQMTGVQHYITAYAADRQIITSGPAYSGRVTLYSNGSLLIQNVTQEDTGSYTLEIIKRVGETRGVTGYFNVTLY

**Old World monkeys**

**Black and white**

**Colobus monkey**  10 20 30 40 50 60 70 80 90 100

| | | | | | | | | |

Can_PSG1N QVTIEAQPTKVPEGKDVLLLVHNLPQNVTGYIWYKGQIMDLYHYITAYTIDTEMIISGPAYSGRETIYSNASLLIQNVTQNDTGSYTIQITQRGDGTKGVTGHFTLY

Can_PSG15N QVTIEAQPTKVSEGKDVLLLVHNLPQNVTGYIWYKGQIMDLYHYITAYTIDTEMIIFGPAYSGRETIYSNASLLIQNVTQNDTGSYTIEITQRGDGTKGVTGHFTLY

Can_PSG11N QVTIEAQPTKVPEGKDVLLLVHNLPQNIAAYIWYKGQIMDVRHYITAYIIDTEMIILGPAYSGRETIYSNASLLIQNVTQNDTGSYTIQITQRGHGTKGVTGHFTLY

Can_PSG13N QVTIEAQPKKVSEGKDVLLLVHNLPQNLAGYIWYKGQIMDLYHYITAYTIDTEMIIFGPAYSGRETVYSNASLLIQNVTQNDTGSYTIQIIKRGDKTNGVTGHFTLH

Can_PSG16N QVTIEAQPTKVSEGKDVLLLVQNLPQNLTGYNWYKGQIMDLYHYVTAYTIDTEITIFGPAYSGRETVYSNGSLLIQNVTQKDTGSYTIQITKRGDRTEGVTGHYTLY

Can_PSG4N QVTIEAQPTKVSEGKDVLLLVHNLPQNLTGYIWYKGQIIDLHQFITAYTIDTETIISGPAYSGRETVYSNASLLIQNVTRKDTGSYTIQIIKRGDKIKGVTGHFTLY

Can_PSG14N QVTIEAQPTKVSEGKDVLLLVQNLPQNVVGYIWYKGQIIDLHHYITAYTIDTETIIFGPAYSGRETVYSNASLLIQSVTKQDIGSYTIKIIKRGDGTEGVTGHFTLY

Can_PSG3N QVTIEAQPTKVSEGKDVLLLVHNLPQNPIGYIWYKGQIMDIDHYITSYVIDAETIIPGPAYSGRETVYSNASLLIQNVTRKDTGSYTIQIIKLGDKTKGVTGHFTLY

Can_PSG12N QVTIEAQPTKVSEGNDVLLLVHNLPQNLTGYIWYKGQIMDYYHYITSYVIDPETIIFGPAYSGRETVYSNASLLIQNVTRKDTGSYTIQIIKRGDRTEGVTGHFTLY

Can_PSG8N QVMIETQPTNVSEGKDVLLLVHNLPQNPTGYIWYKGQITDIHNYITSYVIDTDTIISGPAYSGRETVYSNASLLIQNDTQKDTESYTIQIKKRGDSTKGVTGHFTLY

Can_PSG2N QVMIEAQPNKVSEGKDVLLLVHNLPQNLAAYIWYKGQIMDLHHYITAYVIDPETIIFGPAYSGRETVYSNASLLIQNVTQKDTGSYTIQIIKQGDRTKGVTGHFTLY

Can_PSG6N QVMIEAQPTKVSEAKNVLLLVHNLPQNVAAYLWYKGQIMDVHHYITGYVMETEGIMFGPAYSGRETVYSNGSLLIQNVTWKDTGSYTIQIIKRGDKPKEVIGHFTLY

Can_PSG17N QVMIEAQPTKVSKGKDVLLLVHNLPQNVAAYIWYKGQIMDLHHYIMGYVIEAEAIIFGPAYSGRETVYSNASLLIQNVTWKDTGSYTIQIIKRGDKTKGVTGHFTLH

Can_PSG18N QVTIEAQPTKVSEGKDVLLLVHNLPQNLIGYIWYKGQIMDVHHYIMGYVIEAEAIIFGPAYSGRETVYSNASLLIQNVTRKDTGSYTIQVIKRGDKTKGVTGHFTLY

Can_PSG5N QVMIEAQPTKVSKGKDVLLLVHNLPQNVAAYIWYKGQIMDVHHYITGYVIDPETIIFGPAYTGRERLYSNASLLIQKATQKDTGSYTIKITKRGDKTKGVTGHFTLY

Can_PSG9N QVMIEAQPTNVSEGNDVLLLVHNLPQNPAAYIWYKGQIMDVHHYITAYVIETERIVFGPAYSGRETVYSNASLLIQSLNQKDAGSYTIEIIKRGDGNEGVTGNFTLY

Can_PSG7N QVMIEAQPIKVSEGKDVLLLVHNLPQNAAAYTWYKGQIMDFYQFITAYTRYPDRILFGPAYSGRETLYSNGSLGIQNVTKQDTGSYTVKVMKRIDDTKGVTGHFTLY

Can_PSG10N QVMIEAQPTKVSEGKDVLLLVHNLPQNPAAYVWYKGQIMDFYQFITAYSRDPDRILFGPAYSGRETLYSNGSLRIQNVTKQDTGSYTVKVMKQVDDTKGVTGYLLSV

**Black snub-nosed** 10 20 30 40 50 60 70 80 90 100

**monkey** | | | | | | | | | |

Rbi_PSG1N QVMIEAQPNKVSEGKDVLLLVHNLPQNLAAYIWYKGQIMDLHHYITAYVIDTDTIIFGPAYSGRETVYSNASLLIQNVTQKDTGSYTIQIIKRGDRTEAVTGHFTLY

Rbi_PSG9N QVMIEAQPNKVSEGKDVLLLVHNLPQNVAAYIWYKGQIIDFHHYITGYVMEAEANIFGPAHSGRETVYSNASLLIQNVTQKNTGSYTIQIMKLGNRTEGVTGHFTLY

Rbi_PSG4N QVTIEAQPTNVSEGNDVLLLVHNLPQNPAAYIWYKGQIMDLHHYITAYVIETERIVFGPAYSGRETVYSNASLLIQSVNQKDAGSYTVEIIKRGDGNEGVTGNFTLY

Rbi_PSG2N HVMIEAQPTEVSEGKDVLLLVHNLPQNPTGYIWYKGQIMDIHNYITSYVIDTDTIIFGPAYSGRETVYSNASLLIQNVTQKDTGSYTIQIIKRGDSTKGVTGHFTLY

Rbi_PSG3N QVTIEAQPTKVSEGKDVLLLVHNLPQNLTGYIWYKGQIMDYHHYITAYVIDPETIIFGPAYSGRETVYSNASLLIQNVTRNDTGSYTIKIIKRGDRTEGVTGHFTLY

Rbi_PSG6N QVTIEAQPTKVSEGKDVLLLVHNLPQNIAAYIWYKGQIMDLRHYITAYIIDTEMIILGPAYSGRETVYSNASLMIQNVTQNDTGSYTIQITQRGDGAKGVTGHFTLY

Rbi_PSG10N QVTIEARPTKVSEGKDVLLLVHNLPQNVTGYVWYKGQIMDLYHYITAYIIDTEMIIFGPAYSGRETVYSNASLLIQNVTQNDTGSYTIQITQRGGGIKGVTGHFTLY

Rbi_PSG5N QVMIEAQPTKVSEGKDVLLLVHNLPHNLAGYIWYKGKIMDLYHYITAYTIDTEMIIFGPAYSGRETVYSNASLLIQNVTQKDTGSYTIQIIKRGDKTKGVTGHFTLY

Rbi_PSG8N QVTIEAQPTKVSEGKDVLLLVHNLPQNLTGYIWYKGQIMDLHQFITAYTIDTETIISGPAYSGRETVYSNASLLIQNVTKNDTGSYTIQIIKRGDKIKRVTGHFTLY

Rbi_PSG7N QVTTEAQPTKVSEGKDVLLLVHNLPQNVVGYIWYKGQIMDLRHYITAYTIDNE-IIFGPAYSGREKIYSNASLLIPNVTKQDIGSYTIKIIKRGDETKGVTGHFTLY

Rbi_PSG11N QVMIEAQPTKVSEGKDVLLLVHNLPRNVAAYVWYKGQIMDFYQFITAYSRDPDRILFGPAYSGRETLYSNGSLRIQNVTKQDTGSYTIKVMKRIDETKGVTGHFTLY

Rbi_PSG12N QVMIEAQPTKVSEGKDVLLLVHNLPQNPAAYVWYKGQIMDFYQFIIEYTRYPDRILFGPAYSGRETLYSNGSLLIQNVTKQDTGSYTVKIMKRIDETKGVTGHFTLY

**Crab-eating macaque** 10 20 30 40 50 60 70 80 90 100

| | | | | | | | | |

Mfa_PSG1N QVTIEAQPTKVSEGKDVLLLVHNLPQNLAAYIWYKGQIMDLHHYITSYVIDTEIIVFGPAYSGRETVYSNASLLIQNVTQKDTGSYTIQIIKRGDTTKGVTGHFTLY

Mfa_PSG14N QVTIEAQPTKVSEGKDVLLLVHNLPTNVVGYIWYKGQIMDLHHYITSYVIDTEIIVFGPAYSGRETVYSNASLLIQNVTQKDTGSYTIQIIKRGDITKGVTGHLTLY

Mfa_PSG15N QVTIEAQPTEVSEGKDVLLLVHNLPQNPTGYSWYKGQITDIHHYITSYVIDTEMIVFGPAYSGRETVYSNASLLIQNVTQKDTGSYTIQIIQRGDTTKGVTGHFTLY

Mfa_PSG3N QVTIEAQPAKVSEGKDVLLLVHNLPQNVAGYSWYKGQIMDLHHYITSYVIDTEIIIFGPAYSGRETVYSNASLLIQNVTQKDTGSYTIQITKRGDGTKRVTGHFTLY

Mfa_PSG6N QVTIEAQPTKVSEGKDILLLVHNVPQNVAGYIWYKGQIMDLQHYITSYVIDTEIIIFGPAYSGRERVYSNASLLIQNVSRKDTGSYTIQIIKRGDKIKGVTGHFTLY

Mfa_PSG10N QVTIEAQPAKVSEGKDVLLLVHNLPQNLTGYIWYKGQKTDLHLYVTSYVKDTETVIAGPAYSGRETVYSNASLLIQNVTQKDTGSYTIQITKRGDRTEGETAHFTLY

Mfa_PSG21N QVTIEAQPAKVSEGKDVLLLVHNLPQNVTGYIWYKGQKTDHHLYITSYVIDAETIILGPAYSGRETVYSNASLLIQNVTWKDTGSYTIEIIKRGDRTEGVTGHYTLY

Mfa_PSG4N QVTIEAQPAKVSEGKDVLLLVHNLPQNLTGYIWYKGQIMDHHHYITSYVIDTETIIFGPAYNERETVYSNASLLIQNVTKNDTGSYTIQIIKRGDRTEGVTGHFTLY

Mfa_PSG17N QVTIEAQPTKVSEGKDVLLLVQNLPQNLIAYIWYKGQKTDFHHYITSYVIDAETIIVGPAYSGRETVYSNASLLIQNVTQNDTGSYTIQMIKQGDKTKGVIGHFTLY

Mfa_PSG2N QVTIEAQPAKVSEGKDVLLLVHNLPQNLAACIWYKGQIMDLQHYITAYVIDAETIIFGPAYSGRETVYSNASLLIQNVTQKDTGSYTIQIIQRGDKTKGVTGHFTLY

Mfa_PSG8N QVTIEAQPAKVSEGKDVLLLVQNLPQNLTGYSWYKGQIIDLQHYITAYTIDTEMIVFGPAYSGRETVYSNASLLIQNVTKNDTGSYTIQITKRGDETKGLTGHFTLY

Mfa_PSG16N QVTIEAQPTKLSEGKDVLLLVHNLPQNLTGYSWYKGQIIDLQHYITAYTIDTEMIVLGPAYSGRETVYSNASLLIQNVTKNDTGSYTIQITKRGDETKGVTGHFTLY

Mfa_PSG5N QVMIEAQPTKVSEGKDVLLLVYNLPQNLTGYSWYKGQIMDLQHYIAAYTIDTEMIVFGPAYSGRETVYSNASLLIQNVTKNDTGSYTIQITKQGDETKGVTGHFTLY

Mfa_PSG9N QVTIEAQPAKVSEGKDVLLLVHNLPQNLTGYIWYKGQIMDLQHYITAYAIDTETIIFGPVYSGRETIYSNASLLIQSVTKNDTGSYTIQIIKRGDRTEGVTGHYTLY

Mfa_PSG11N QVTIEAQPTKVSEGKDILLLVHNVPQNVAGYIWYKGQIMDLQHYITAYAIDTETIIFGPVYSGRETIYSNASLLIQSVTKNDTGSYTVEIVKRGEGTEGVTGHFTLY

Mfa_PSG19N QVTIEAQPTKVSEGKDVLLLVHNLPTNVVGYIWYKGQIMDLQHYITAYTIDTEMIIFGPAYSGRETVYSNASLLIQSVTKNDTGSYTIQIIKRGHRTEGVTGHFTLY

Mfa_PSG20N QVTIEXQPTKVSEGKDVLLLVHNLPTNVVGYIWYKGQIMDLQHYITAYTTDTEMILFGPAYSGRETVYSNASLLIQSVTXNDTGSYTIQIIKRGHRTEGVTGHYTLY

Mfa_PSG22N QVTIEAQPTKVSEGKDVLLLVHNLPTNVVGYIWYKGQIMDLQHYITAYTTDTEMILFGPAYSGRETVYSNASLLIQNVTWKDTGSYTIQIIERGEGTEGVTGHFTLY

Mfa_PSG7N QVTIEAQPAKVSEGKDVLLLVHNLPQNLTGYVWYKGQIMDLHQFITAYTIDTDTIIFGPAYSGRETVYSNASLLIQNVTRKDTGSYTIQIIKRGDKIKRITGHFTLY

Mfa_PSG18N QVTIEAQPTKVSEGKDVLLLVHNLPQNLTGYXWYKGQIMDLHQFITAYTIDTDTIIFGPAYSGRERVYSNASLLIQNVXRKDTGSYTIQIIKRGDKIKRITGHFTLY

Mfa_PSG12N QVTIEAQPAKVSEGKDVLLLVQNLPENLTGYVWFKGQIMDFHQFITAYTIDTETIIFGPAYSGRETVYSNASLLIQNVTQNDTGSYTIEIIKRGDKIKGVTGHFTLY

Mfa_PSG13N QVTIEAQPAKVSEGKDVLLLVQNLPQNLTGYVWFKGQITTFHQFIIAYKIDSRKITVGPVYSGRERVYSNASLLIQNVSRKDTGSYTIQIIKRGDKTKGVTGHFTLY

**De Brazza’s monkey** 10 20 30 40 50 60 70 80 90 100

| | | | | | | | | |

Cne_PSG1N QVVIEAQPAKVSEGKDVLLRVHNLPQNLTGYIWYKGQIMDLQHYITAYTIDTEMIIFGSAYSGRETVYSNASLLIQNVTKNDTGSYTIQITK-RGDETKGVTGHFTLY

Cne_PSG2N QVVIEAQPAKVSEGKDVLLRVHNLPQNLTGYIWYKGQIMDLQHYITAYTIDTEMIIFGSAYSGRETVYSNASLLIQNVTKNDTGSYTIQITK-RGDETKGVTGHFTLY

Cne_PSG3N QDMIEAQPTKVSKGKDVLLLVHNLPQNLTGYIWYKGQIMDLHHYIAAYTIDTEMIIFGPAYSGRETLYSNASLLIQNVTQNDTGSYTIQIRK-RDDETKGVTGHFTLY

Cne_PSG13N QVTIEAQPAKVPEGKDVLLLVHNLPQNLTGYIWYKGQITDHHHYITSYVIDTETIIFGSAYSGRETVYSNASLLIQNVTRNDTGSYTIQIIK-RGDRTEGITGHFTLY

Cne_PSG14N QVTIEAQPAKVPEGKDVLLLVHNLPQNLTGYIWYKGQITDHHHYITSYVIDTETIIFGSAYSGRETVYSNASLLIQNVTRNDTGSYTIQIIK-RGDRTEGITGHFTLY

Cne_PSG9N QVTIETQPTKVSEGKDVLLLVHNLPQNLTGYIWYKGQIMDHHHYITSYVIDTETIIFGPAYSGRETVYSNASLLIQNVTWNDTGSYTIQIIK-RGDRTEGITGHYTLY

Cne_PSG10N QVTIEAQPTKVSEGKDVLLLVHNLPQNLTGYIWYKGQKTDLHLYVTSYVKDTETIIAGPAYSGRETVYSNASLLIQNVTQKDTGPYTIQITK-RGDRTEGETAHFTLY

Cne_PSG12N QVTIEAQPTKVSEGKDVLLLVHNLPQNLTGYIWYKGQKMDLHHYITSYVIDAETIIAGPAYSGREIVYSNASLLIQNVTRKDTGSYTIQTIK-QGDNSKGVIGHFTLY

Cne_PSG4N QVTIEAQPTKLSEGKDVLLLVHNLPQNLIGYSWYKGQIMDLHHYITSYVIDTEMIVFGPAYSGRETVYSNASLLIQNVTRKDTGSYTIQIIK-RGDKIKRITGHFTLY

Cne_PSG5N QVTIEAQPTKVSEGKDVLLRVHNLPQNVAGYIWYKGQIMDLQHYITSYVIDTEMIVFGPAYSGRETVYSNASLLIQNVTQNDTGSYIIQITN-RCDETKGVTEHFTLY

Cne_PSG7N QVTIEAQPAKVSEGKDVLLRVHNLPQNLAAYIWYKGQIMDLHHYITSYVIDTEIIVFGPAYSGRETVYSNASLLIQNVTQKDTGSYTIQIIM-RGNRTKGVTGHFTLY

Cne_PSG11N QVTIEAQPTEVSEGKDVLLLVHNLPQNPTGYIWYKGQITESHNYITSYVIDTEMILFGPAYSGRETVYSNASLLIQNVTQ-DTGSYTIQIIQ-RGDSTKRVTGHFTLY

Cne_PSAG17N QVTIEAQPTKVSEGKDILLLVQNLPQNLTGYIWFKGHITNYHQFIIAYAIDSKNITVGPAYSGRERVYSNASLLIQNVTQKDTGSYTIEIIK-QGDKTKGVTGHFTLY

Cne_PSG18N QVTIEAQPTKVSEGKDILLLVQNLPQNLTGYVWFKGQITNYHQFIIAYAIDSKNITVGPAYSGRETVYSNASLLIQNVTQKDTGSYTIEIIK-QGDKTKGVTGHFTLY

Cne_PSG19N QVTIEAQPAKVSEGKDILLLVQNLPQNLTGYVWFKGQKTNYHQFIIAYAIDSKNITVGPAYSGRETVYSNASLLIQNVTQKVTGSYTIQIIK-RGDKIKGVTGHFTLY

Cne_PSG15N QVTIEAQPAKVSEGKDILLLVQNLPQNLTGYIWFKGQITNFHQFIIAYTIDSKKITVGPAYSGRERVYSNASLLIQNVTQKDTGSYTIQITK-RGDKIKGVTGHFTLY

Cne_PSG6N QVTIEAQPTKVSEGKDILLLVHNLPQNVAGYIWYKGQIMDLQYYITAYEIDTEMIIFGPAYSGRETVYSNASLLIQSVNQKDAGSYTIEIIK-RGDGTEGVTGHFTLY

Cne_PSG8N QVTIEAQPTNVSEGKDILLLVHNLPQNVAGYIWYKGQIMDLQHYITAYTIDTETIIFGPAYSGRETVYSNASLLIQSVNQKDVGSYTVEIIIKRGDGTEGVTGHFTLY

Cne_PSG16N QVMIEAQPTKVSEGKDVLLLVRNLPQKVSGYVWYKGQIMDFHQFITAYTIDTERIIFGTAYSGRETLYSNGSLLIRNVTKQDTGSYTVKIIE-RAEETKEVTVHFTLY

**Drill**  10 20 30 40 50 60 70 80 90 100

| | | | | | | | | |

Mle_PSG2N QVTIEAQPAKVSEGKDVLLLVHNLPQNVAGYSWYKGQIMDLQHYITAYTTDTEMIIFGPAYSGRETVYSNASLLIQNVTQKDTGSYTIQITKRGDGTKRVTGHFTLYL

Mle_PSG5N QVTIEAQPTKVSEGKDVLLLVYNLPENLTGYSWYKGQIMDLQHYIAAYTTDTEMIIFGPAYSGRETVYSNASLLIQNVTKNDTGSYTIQITKRGDGTKGVTGHFTLYL

Mle_PSG4N QVTIEAQPTKVSEGKDVLLLVHNLPQNPTGYSWYKGQITDIHHYITSYVIDTEMIVFGPAYSGRETVYSNASLLIQNVTQNDTGSYTIEIIQRGDTTKGVTGHFTLYP

Mle_PSG8N QVTIEAHPTEVSEGKDVLLLVHNLPQNPTGYIWYKGQIMDSHNYITSYVIDTEMIVFGPAYSGRETVYSNASLLIQNVTWKDTGSYTIQIIKRGDTTKGVTGHFTLYV

Mle_PSG1N QVTIEAQPTKVSEGKDVLLLVHNLPQNLAAYIWYKGQIMDLHHYITSYVIDTEIIVFGPAYSGRETVYSNASLLIQNVTQKDTGSYTIQIIKRGDTTKGVTGHFTLYP

Mle_PSG3N QVRIEAQPTKVSEGKDVLLLVHNLPQNLTGYIWYKGQKTDLHLYITSYVKDTETVIAGPAYSGRETIYSNASLLIQNVTQKDTGSYTIEITKRGDRTEGETGHFTLYL

Mle_PSG11N QVTIEAQPAKVSEGKDVLLLVHNLPQNLTGYIWYKGQKTDLHLYITSYVIDTERIILGPAYSGRETVYSNASLLIQNVTQKDTGSYTIQIIKRGHRTEGVTGHYTLYL

Mle_PSG7N QVTIEAQPTNISEGNDVLLLVHNLPKNPVAYIWYKGQIMDLQHYITAYTIDTERIIFGPAYSGRERVYSNASLLIQSVNQKDAGSYTVKIIKRGYRTEGVTGHFTLYV

Mle_PSG6N QVTIEAQPTKVSEGKDVLLLVHNLPTNVVGYIWYKGQIMDLQHYITAYTIDTEKIIFGPAYSGRETVYSNASLLIQNVTKNDTGSYSIQITNPCDETKGVTGHFTLYV

Mle_PSG9N QVMIEAQPTKVSEGKDVLLPVRNLPQKVAAYIWYKGQIMDFHQFITAYTIDTERIIFGPAFSGRETLYSNGSLLIRNVTKNDTGSYTVKIRNPAEETKGVIVHFTLYP

Mle_PSG10N QVMIEAQPTKVSEGKDVLLLVRNLPQKVAAYVWYKGQIMDFHQFITAYTIDPERIIFGYAYSGRETLYSNGSLLIRNVTKQDTGSYTVKIMNRMEETKGVTVHFTLYL

**Francois’ langur** 10 20 30 40 50 60 70 80 90 100

| | | | | | | | | |

Tfr_PSG1aN QVTIEAQPTKVSEGKDVLLLVHNLPQNLTGYIWYKGQIMDLYHYITAYTIDTEIIIFGPAYSGRETVYSNASLLIQNVTQKDTGSYTIKIIKRGDRTEGVTGHFTLY

Tfr_PSG1bN QVTIEAQPTKVSEGKDVLLLVHNLPQNLTGYIWYKGQMMDLYHYITAYTIDTEITIFGPAYSGRETVYSNASLLIQNVTQKDTGSYTIKIIKRGDRTEGVTGHFTLY

Tfr_PSG4N QVTIEAQPTKVSEGKDVLLLVHNLPQNVIGYVWYKGQIMDLYHYITAYIIDTEMIIFGPAYSGRETVYSNASLLIQNVTQNDTGSYTIQITQRGDGIKGVTGHFTLY

Tfr_PSG6N QVTIEAQPTKVSEGKDVLLLVHNLPQNIAAYIWYKGQIMDLRHYITAYIIDTEMIILGPAYSGRQTVYSNASLLIQNVTQNDTGSYTIRITQRGDGTKGVTGHFTLY

Tfr_PSG3N QVMIEAQPTKVSEGKDVLLLVHNLPQNLAGYIWYKGQIMDLYHYITAYTIDTEMIIFGPAYSGRETVYSNASLLIQNVTQNDTGSYTIQIIKRGDKTKGVTGHFTLY

Tfr_PSG9N QVTIEAQPTKVSEGKDVLLLVHDLPQNLTGYIWYKGQIMDLHQFITAYTIDTETIIFGPAYSGRETVYSNASLLIRNVTQNDTGSYMIQIIKRGDKIKRVTGHFTLY

Tfr_PSG10N QVTTEAQPTKVSEGK-HVLLVHNLPQNVVGYIWYKGQIMDVHQFITAYTIDTETIIFGPVYSGREKIYSNASLLIQNVTQNDTGSYTIKIIKRGDETKEVTGHFTLY

Tfr_PSG7N QVTIEAQPTKVSEGKDVLLLVHNLPQNLIGYIWYKGQVMDFHHYITSYVIDAETVIIGPAYSGRETVYSNASLLIQNVTRKDTGSYTIQIIKLGDRTKGVTGHFTLY

Tfr_PSG11N HVMIEAQPTEVSEGKDVLLLVHNLPQNPTGYIWYKGQITDIHNYITSYVIDTDTIIFGPAYSGRETVYSNASLLIQNVTRKDTGSYTIQIIKRGDSTKGVTGHFTLY

Tfr_PSG5N QVMIEAQPTKVSEGKDVLLLVHNLPQNLAAYIWYKGQIMDLHHYITAYVKDTDTIIFGPAYSGRETVYSNASLLIQNVTRKDTGSYTIQIIKLGDRTKGVTGHFTLY

Tfr_PSG8N QVTIEAQPTKVSDGKDVLLLVHNLPQNVAAYIWYKGQIIDLHHYIMGYVIEAEAILFGPAYSGRETVYSNASLLIQNVTQKDTGSYTIQIIKRGDKTKGVTGHFTLY

Tfr_PSG15N QVMIEAQPTKVSEGKNVLLLVHNLPQNVAAYIWYKGQIMDVHHYITGYVMEAEAVIFGPAYSGRETVYSNASLLIQNVTQKDTGSYTIQIIKRGDRIEGVTGHFTLY

Tfr_PSG12N QVMIEAQPTKVSEGKDVLLLVHNLPQNVAAYIWYKGQIMDVHHYVTGYVIHPETIIFGPAYTGRERLYSNASLLIQNVTQKDTGSYTIKITKRGDKTKRVTGHFTLY

Tfr_PSG13N QVTIEAQPTNVSEGNDVLLLVHNLPQNPAAYIWYKGHLMDLHHYITAYVIETERIVFGPAYSGRETVYSNASLLIQSVNQKDAVSYTVEIIKQGDGNEGVTGNFTLY

Tfr_PSG2N QVMIEAQPTKVSEGKDVLLLVHNLPQNVAAYVWYQGQIMDFYQFITAYSRDPDRILFGPAYSGRETVYSNGSLRIQNVTKQDTGSYTVKVMKRIDETKGVTGHFTLY

Tfr_PSG14N QVMIETQPTKVSEGKDVLLLVHNLPQNPAAYVWYKGQIMDFYQFIIAYTRYPDRILFGPAYSGRETLYSNGSLLIQNVTKQDTGSYTVKIMKRIDDTKGVTGHFTLY

**Gelada baboon** 10 20 30 40 50 60 70 80 90 100

| | | | | | | | | |

Tge_PSG5N QVTIEAQPTNISEGNDVLLLVHNLPQNPAAYIWYKGQIMDLHHYITAYTIDTERIIFGPAYSGRERVYSNASLLIQSVNQKDAGSYTIKIIKRGDGTEGVTGHFTLY

Tge_PSG13N QVTIEAQPTNISEGNDVLLLVHNLPKNPAAYIWYKGQILDLHHYITAYTVDTERIIFGSAYSGRERVYSNASLLIQSVNQKDAGSYTVQIIKQGDRTEGVTGHFTLY

Tge_PSG6N QVTIEAQPAKVSEGKDVLLLVHNLPQNLAACIWYKGQIMDLQHYITAYVIDAETIIFGPAYSGRETVYSNASLLIQNVTQKDTGSYTIQIIQRGDKTKGVTGHFTLY

Tge_PSG7N QVTIEAQPTKVSEGKDVLLLVQNLPQNLIAYIWYKGQKTDFRHYITSYVIDAETIIVGPAYSGRETVYSNASLLIQNVTQKDTGSYTIQMIKQGDKTKGVIGHFTLY

Tge_PSG2N QVTIEAQPTKVSEGKDVLLLVHNLPQNPTGYIWYKGQIMDLHHHITSYVVDTEIIVFGPAYSGRETVYSNASLLIQNVTQKDTGSYTIQIIQRGDTTKGVTGHFTLY

Tge_PSG10N QVTIEAQPTEVSEGKDVLLLVHNLPQNPTGYSWYKGQITDLHHYITSYVIDTEMIVFGPAYSGRETVYSNASLLIQNVTQKDTGSYTIEIIQRGDTTKGVTGHFTLY

Tge_PSG3N QVTIEAQPTEVSEGKDVLLLVHNLPQNPTGYIWYKGQITDSHNYITSYVIDTEMIIFGPAYSGRETVYSNASLLIQNVTRKDTGSYTIQIIKRGDSTKGVTGHYTLY

Tge_PSG9N QVTIEAQPTKVSEGKDVLLLVHNLPQNLTGYIWYKGQKTDLHLYVTSYVKDTETVIAGPAYSGRETVYSNASLLIQNVTQKDTGSYTIEITKRGDRTEGEIGHFTLY

Tge_PSG12N QVTIEAQPTKVSEGKDVLLLVHNLPQNLTGYIWYKGQKMDHHHYITSYVIDTETIIFGPAYSERETVYSNASLLIQNVTKNDTGSYTIQIIKRGHRTEGVTGHYTLS

Tge_PSG4N QVTIEAQPTKVSEGKDVLLLVQNLPQNLTGYVWYKGQKTDLHQFITAYTIDTETIISGPAYSGRETVYSNASLLIQNVTRNDTGSYTIQIIQRGDKIKRITGHFTLY

Tge_PSG11N QVTIEAQPAKVSEGKDVLLLVQNLPENLTGYVWFKGQIMDFHQFITAYTIDTETIIFGPAYSGRETVYSNASLLIQNVTQNDTGSYTIEIIKRGDKIKGVTGHFTLY

Tge_PSG1N QVTIEAQPTKVSEGKDVLLLVHNLPQNVAGYSWYKGQIMDLHHYITAYTIDTEMIIVGPAYSGRETVYSNASLLIQNVTQKDTGSYTIQIIKRGDGTKRVTGHFTLY

Tge_PSG8N QVMIEAQPTKVSEGKDVLLPVRNLPQEVAAYIWYKGQIMDFHQFITAYTIDTERIIFGPAYSGRETVYSNGSLLIRNVTKQDTGSYTVKIRKPAEETKGVTVHFTLY

**Gold snub-nosed**  10 20 30 40 50 60 70 80 90 100

**monkey**  | | | | | | | | | |

Rro_PSG7N QVMIEAQPNKVSEGKDVLLLVHNLPQNVAAYIWYKGQIIDFHHYITGYVMEAEANIFGPAHSGRETVYSNASLLIQNVTQKNTGSYTIQIMKLGNRTEGVTGHFTLY

Rro_PSG11N QVMIEAQPSKVSNGKDVLLLVHNLPQNVAAYIWYKGQIRDLHHYIMGYVIEAEAILSGPAYSGRETVYSNASLLIQNVTQKDTGSYTVQIIKQGDKTEGVTGHFTLY

Rro_PSG1N QVMIEAQPNKVSEGKDVLLLVHNLPQNLAAYIWYKGQIMDLHHYITAYVIDTDTIIFGPAYSGRETVYSNASLLIQNVTQKDTGSYTIQIIKRGDRTEAVTGRFTLY

Rro_PSG8N QVTIEAQPTKVSEGKDVLLLVHNLPQNLIGYIWYKGQVTDFHHYITSYVIDVETIIFGPAYSGRETVYSNASLLIQNVTRKDTGSYTIQIIKLGDRTKGVTGHFTLY

Rro_PSG9N QVTIEAQPTKVSEGKDVLLLVHNLPQNLIGYIWYKGQVMDFHHYITSYVIDAETIITGPAYSGRETVYSNASLLIQNVTRKDTGSYTIQIIKLGDRTKGATGHFTLY

Rro_PSG2N QVTIEAQPTKVSEGKDVLLLVHNLPQNLTGYIWYKGQIMDYHHYITAYVIDPETIIFGPAYSGRETVYSNASLLIQNVTRNDTGSYTIKIIKRGDRTEGVTGHFTLY

Rro_PSG5N QVTIEARPTKVSEGKDVLLLVHNLPQNVTGYVWYKGQIMDLYHYITAYIIDTEMIIFGPAYSGRETVYSNASLLIQNVTQNDTGSYTIQITQRGDGIKGVTGHFTLY

Rro_PSG10N QVTIEAQPTKVSEGKDVLLLVHNLPQNIAAYIWYKGQIMDLRHYITAYIIDTEMIILGPAYSGRETVYSNASLLIQNVTQNDTGSYTIQITQRGDGAKGVTGHFTLY

Rro_PSG3N QVMIEAQPTKVSEGKDVLLLVHNLPQNLAGYIWYKGKIMDLYHYITAYTIDTEMIIFGPAYSGRETVYSNASLLIQNVTQNDTGSYTIQIIKRGDKTKGVTGHFTLY

Rro_PSG4N QVTIEARPTKVSEGKDVLLLVHNLPQNLTGYIWYKGQIMDLHQFITAYTIDTETIISGPAYSGRETVYSNASLLIQNVTKNDTGSYTIQIIKRGDKTKGVTGHFTLY

Rro_PSG12N QVTTEAQPTKVSEGKDVLLLVHNLPQNVVGYIWYKGQIMDLRHYITAYTIDNETIIFGPAYSGREKIYSNASLLIPNVTKQDIGSYTIKIIKRGDETKGVTGHFTLY

Rro_PSG6N QVMIEAQPTKVSEGKDVLLLVHNLPRNVAAYVWYKGQIMDFYQFITAYSRDPDRILFGPAYSGRETLYSNGSLRIQNVTKQDTGSYTIKVMKRIDETKGVTGHFTLY

Rro_PSG13N QVMIEAQPTKVSEGKDVLLLVHNLPQNPAAYVWYKGQIMDFYQFIIEYTRYPDRILFGPAYSGRETLYSNGSLLIQNVTKQDTGSYTVKIMKRIDETKGVTGHFTLY

**Green monkey** 10 20 30 40 50 60 70 80 90 100

| | | | | | | | | |

Csa_PSG5N QVRIEAQPTKVSEGKDILLLVHNLPQNVAGYIWYKGQIMDLQYYITAYAIDTEMLIFGPAYSGRETVYSNASLLIQSVNQKDAGSYTVEIIKRGEGTEGVTGHFTLY

Csa_PSG8N QVTIEAQPTKVSEGKDILLLVHNLPQNVAGYIWYKGQIMDLQYYITAYAIDTEMLIFGPAYSGRETVYSNASLLIQSVNQNDAGSYTVEIIKRGEVTEGVTGHFTLY

Csa_PSG2N QVTIEAQPAKVSEGNDVLLRVHNLPQNLAAYIWYKGQIMDLHHYITSYVIDTEIIVFGHAYSGRETVYSNASLLIQNVTQKDTGSYTIQIIMRGDRTKGVTGHFTLY

Csa_PSG12N QVTIEAQPAKVSEGNDVLLRVHNLPQNLAAYIWYKGQIMDLHHYITSYVIDTEVIVFGPAYSGRETVYSNASLLIQNVTQKDTGSYTIQIIMRGDRTKGVTGHFTLY

Csa_PSG1N QVVIEAQPAKVSEGKDVLLLVHNLPQNLAACIWYKGQIMDLQHYITAYVKDAETIIFGPAYSGRETVYSNASLLIQNVTQKDTGSYTIQIIKRGDKTKGVTGHFTLY

Csa_PSG3N QVTVEAQPTKVSEGKDVLLLVHNLPQNLTGYSWYKGQITDHHHYITSYVIDTETIIFGPAYSGRETVYSNASLLIQNVTRNDTGSYTIQIIKRGDRTEGITGHYTLY

Csa_PSG11N QVTIEAQPTKVSEGKDVLLLVHNLPQNLTGYIWYKGQKTDLHLYVTSYVKDTETIIAGPAYTGRETVYSNASLLIQNVTQKDTGSYTIQITKRGDRTEGETAHFTLY

Csa_PSG10N QVTIEAQPTEVSEGKDVLLLVHNLPQNPTGYSWYKGQITDIHHYITSYVIDTEIIIFGPAYSGRETVFSNASLLIQNVTQKDTGSYTIQIIQRGDTTKGVTGHFILY

Csa_PSG13N QVTIEAQPIEISEGKDVLLLVHNLPQNPTGYIWYKGHIMESHNYFTSYVIDTEMIIFGSAYSGRETVYSNASLLIQNVTQKDTGSYTIQIIKRGDSTKGVTGHFTLY

Csa_PSG4N QVTIEAQPTKVSEGKDVLLLVHNLPQNVAGYIWYKGQIMDLQHYIIAYTIDTEMIIFGSAYSGRETVYSNASLLIQNVTKNDTGSYTIQIRKRGDESKGVTGHFTLY

Csa_PSG7N QVTIEAQPTKVSKGKDVLLLVHNLPQNLTGYSWYKGQIMDLQHYIAAYTIDTEMIIFGPAYSGRETVYSNASLLIQNVTKNDTGSYTIQIRKRGDKTKGVTGHFTLY

Csa_PSG6N QVTIEAQPTKVSEGKDVLLLVHNLPQNVAGYSWYKGQIMDLHHYITAYTIDTEMIILGPAYSGRETVYSNASLLIQNVTQKDTGSYTIQITKRGDGTKRVTGHFTLY

Csa_PSG9N QVTIEAQPTKIYEGKDVLLLVQNLPQNLTGYVWFKGQIMDLHQFITAYTIDTETIIFGPAYSGRETVYSNASLLIQNVTQNDTGSYTIEIIKRGDKIKGVTGHFTLY

Csa_PSG14N QVTIEAQPTKVSEGKDILLLVQNLPQNLTGYVWFKGQITNYHQFIIAYAIDSKNITVGPAYSGRETVYSNASLLIQNVTQKVTGSYTIEIIKRGDKIKGVTGHFTLY

Csa_PSG15N QVMIEAQPTKVSEGKDVLLLVRNLPQKVAAYVWYKGQIMDFHQFITAYTIDTERFIFGPACSGRETLYSNGSLLIRNVTKQDTGSYTVKIIERAEETKGVTVHFTLY

**Hanuman langur** 10 20 30 40 50 60 70 80 90 100

| | | | | | | | | |

Sen_PSG8N QVMIEAQPTKVSEGKDVLLLVHNLPQNVAAYIWYKGQIMDVHHYITGYVIHPETIIFGPAYTGRERLYSNASLLIQKVTQKDTGSYTIKITKRGDKTKRVTGHFTLY

Sen_PSG13N QVMIEAQPTKVSDGKDVLLLVHNLPQNVAAYIWYKGQIIDLHHYIMGYVIEAEAILFGPAYSGRETVYSNASLLIQNVTQKDTGSYTIQIIKRGDKTKGVTGHFTLY

Sen_PSG14N QVTIEAQPTNVSEGNDVLLLVHNLPQNPAAYIWYKGQIMDLHHYITAYVIETERIVFGPAYSGRETVYSNASLLIQSVNQKDAVSYTVEIIKRGDGNEGVTGNFTLY

Sen_PSG6N QVMIEAQPTKVSEGKDVLLLVHNLPQNLAAYIWYKGQIMDIHHYITAYVKDTDTIIFGPAYSGRETVYSNASLLIQNVTQKDTGSYTIQIIKRGDRTEAVTGHFTLY

Sen_PSG3N QVTIEAQPTKVSEGKDVLLLVHNLPQNIAAYIWYKGQIMDLRHYITAYIIDTEMIILGPAYSGRETVYSNASLLIQNVTQNDTGSYTIQITQRGDGIKGVTGHFTLY

Sen_PSG7N QVTIEAQPTKVSEGKDVLLLVHNLPQNIAAYIWYKGQIMDLRHYITAYIIDTEMIILGPAYSGRETVYSNASLLIQNVTQNDTGSYTIQITQRGDGIKGVTGHFTLY

Sen_PSG2N QVTIEAQPTKVSEGKDVLLLVHNLPQNVIGYVWYKGQIMDLYHYITAYIIDTEMIIFGPAYSGRETVYSNASLLIQNVTQNDTGSYTIQITQRGDGIKGVTGHFTLY

Sen_PSG5N QVMIEAQPTKVSEGKDVLLLVHNLPQNLAGYIWYKGQIMDLYHYITAYTIDTEMIIFGPAYSGRETVYSNASLLIQNVTQNDTGSYTIQIIKRGDKTKGVTGHFTLY

Sen_PSG9N QVTIEAQPTKVSEGKDVLLLVHNLPQNLIGYIWYKGQVMDFHHYITSYVIDAETVIIGPAYSGRETVYSNASLLIQNVTRKDTGSYTIQIIKLGDRTKGVTGHFTLY

Sen_PSG10N QVTIEAQPTKLSEGKDVLLLVHNLPQNLIGYIWYKGQVMDLHHYITSYVIDVETIIFGPAYSGRETVYSNASLLIQNVTWKDTGSYTIQIIKLGDRTKGVTGHFTLY

Sen_PSG4N QVTIEAQPTKVSEGKDVLLLVHNLPQNLTGYIWYKGQMMDLYHYITAYTIDTEITIFGPAYSGRETVYSNASLLIQNVTQKDTGSYTIKIIKRGDRIEGVTGHFTLY

Sen_PSG1N QVTTEAQPTKVSEGKDVLLLVHNLPQNVVGYIWYKGQIMDVHQFITAYTIDTETIILGPVYSGREKIYSNASLLIQNVTQNDTGSYTIKIIKRGDETKGVTGHFTLY

Sen_PSG11N QVMIEAQPTKVSEGKDVLLLVHNLPQNVAAYVWYQGQIMDFYQFITAYSRDPDRILFGPAYSGRETVYSNGSLRIQNVTKEDTGSYTVKVMKRIDETKGVTGHFTLY

Sen_PSG12N QVMTEAQPTKVSEGKDVLLLVHNLPQNPAAYVWYKGQIMDFYQFIIAYTRYPDRLLFGPAYSGRETLYSNGSLLIQNVTKQDTGSYTVKIMKRIDDTKGVTGHFTLY

**Japanese macaque** 10 20 30 40 50 60 70 80 90 100

| | | | | | | | | |

Mfu_PSG8N QVTIEAQPTKVSEGKDILLLVHNVPQNVAGYIWYKGQIMDLQHYITAYAIDTETIIFGPVYSGRETMYSNASLLIQSVNQKDAGSYTVEIVKRGEGTEGVTGHFTLY

Mfu_PSG12N QVTIEAQPTNISEGNDVLLLVHNLPKNPAAYIWYKGQIMDLQNYITAYTIDTERIIFGPAYSGRERVYSNASLLIQSVNQKDAGSYTVKIIKRGYRTEGVTGHFTLY

Mfu_PSG7N QVTIEAQPTKVSEGKDVLLLVQNLPENLTGYVWFKGQIMDFHQFITVYTIDTETIIFGPAYSGRETVYSNASLLIQNVTQNDTGSYTIEIIKRGDKIKGVTGHFTLY

Mfu_PSG9N QVTIEAQPTKVSEGKDVLLLVQNLPENLTDYVWFKGQIMDFHQFITVYTIDTETIIFGPAYSGRETVYSNASLLIQNVTQNDTGSYTIEIIKRGDKIKGVTGHFTLY

Mfu_PSG10N QVTIEAQPAKVSEGKDVLLLVQNLPENLTGYVWFKGQIMDFHQFITAYTIDTETIIFGPAYSGRETVYSNASLLIQNVTQNDTGSYTIEIIKRGDKIKGVTGHFTLY

Mfu_PSG2N QVTIEAQPTKVSEGKDVLLLVHNLPQNVAGYSWYKGQIMDLHHYITSYVIDTEIIIFGPAYSGRETVYSNASLLIQNVTQKDTGSYTIQITKRGDGTKRVTGHFTLY

Mfu_PSG3N QVTIEAQPTKVSEGKDVLLLVHNLPQNLAAYIWYKGHIMDLHHYITSYVIDTEIIVFGPAYSGRETVYSNASLLIQNATQKDTGSYTIQIIKRGDTTKGVTGHFTLY

Mfu_PSG5N QVTIEAQPTEVSEGKDVLLLAHNLPQNPTGYSWYKGQITDIHHYITSYVIDTEMIVFGPAYSGRETVYSNASLLIQNVTQKDTGSYTIQIIQRGDTTKGVTGHFTLY

Mfu_PSG6 QVTIEAQPAKVSEGKDVLLLVQNLPQNLIAYIWYKGQKTDFHHYITSYVIDAETIIVGPAYSGRETVYSNASLLIQNVTKNDTGSYTIQMIKQGDKTKGVIGHFTLY

Mfu_PSG11N QVTIEAQPAKVSEGKDVLLLVHNLPQNVTGYIWYKGQKTDHHLYITSYVIDAETIILGPAYSGRETVYSNASLLIQNVTWKDTGSYTIEIIKRGDRTEGVTGHYTLY

Mfu_PSG4N QVTIEAQPTKVSEGKDVLLLVHNLPQNLTGYIWYKGQIMDHHHYITSYVIDTETIIFGPAYNERETVYSNASLLIQNVTKNDTGSYTIQIIKRGDRTEGVTGHYTLY

Mfu_PSG1N QVMIEAQPTKVSEGKDVLLLVYNLPQNLTGYSWYKGQIMDLQHYIAAYTIDTEMIVFGPAYSGRETVYSNASLLIQNVTKNDTGSYTIQITKQGDETKGVTGHFTLY

Mfu_PSG13N QVMIEAQPTKVSEGKDVLLLVRNLPQKVAAFVWYKGQIMDFHQFITAYTIDTERIIFGYAYSGRETLYSNGSLLIRNVTKQDTGSYTVKIMNRMEETKGVTVHFTLY

**Mandrill** 10 20 30 40 50 60 70 80 90 100

| | | | | | | | | |

Msp_PSG2N QVTIEAHPTEVSEGKDVLLLVHNLPQNPTGYIWYKGQIMDSHNYITSYVIDTEMIVFGPAYSGRETVYSNASLLIQNVTWKDTGSYTIQIIKRGDTTKGVTGHFTLY

Msp_PSG3N QVTIEAQPTKVSEGKDVLLLVHNLPQNLAAYIWYKGQIMDLHHYITSYVIDTEIIVFGPAYSGRETVYSNASLLIQNVTQNDTGSYTIQIIKRGDKTKGVTVHFTLY

Msp_PSG1N QVTIEAQPAKVSEGKDVLLLVHNLPQNVAGYSWYKGQIMDLQHYITAYTTDTEMIIFGPAYSGRETVYSNASLLIQNVTQKDTGSYTIQITKRGDGTKRVTGHFTLY

Msp_PSG4N QVTIEAQPAKVSEGKDVLLLVHNLPQNLTGYIWYKGQKTDHHLYITSYVIDTETIIFGPAYSGRETVYSNASLLIQNVTRKDTGSYTIEIIKRGDRTEGVTGHYTLY

Msp_PSG5N QVTIEAQPAKVSEGKDVLLLVQNLPQNLTGYVWYKGQKTDLHQFITAYTIDTETIISGPAYSGRETVYSNASLLIQNVTRKDTGSYTIQIIKRGDKIKRITGHFTLY

**Olive baboon** 10 20 30 40 50 60 70 80 90 100

| | | | | | | | | |

Pan_PSG3N QVTIEAQPTKVSEGKDVLLLVHNLPQNPTGYIWYKGQIMDLHHHITSYVIDTEIIVFGPAYSGRETVYSNASLLIQNVTQKDTGSYTIQIIQRGDSTKGVTGHFTLY

Pan_PSG4N QVTIEAQPTKVSEGKDVLLLVHNLPQNPTGYIWYKGQIMDLHHHITSYVKDTETVIAGPAYSGRETVYSNASLLIQNVTQKDTGSYTIQIIQRGDTTKGVTGHFTLY

Pan_PSG1N QVTIEAQPTKVSEGKDVLLLVHNLPQNLAACIWYKGQIMDLQHYITAYVIDAETIIFGPAYSGRKTVYSNASLLIQNVTQKDTGSYTIQIIQRGDKTKGVTGHFTLY

Pan_PSG10N QVTIEAQPTNISEGNDVLLLVHNLPQNPAAYIWYKGQIMDLHHYITAYTIDTERIIFGPAYSGRERVYSNASLLIQSVNQKDAGSCTIQIIKRGDGTEGVTGHFTLY

Pan_PSG13N QVTIEAQPTKVSEGKDVLLLVHNLPTNVVGYVWYKGQIMDLQHYITAYTTDTEMIIFGPEYSGRETVYSNASLLIQSVTKNDTGSYTIQIIKRGHRTEGVTGHYTLY

Pan_PSG14N EVTIEAQPTKVSEGKDVLLLVHNLPTNVVGYVWYKGQIMDLQHYITAYTTDTEMIIFGPEYSGRETVYSNASLLIQSVTKNDTGSYTIQIIKRGHRTEGVTGHYTLY

Pan_PSG2N QVTIEAQPTKVSEGKDVLLLVHNLPTNVVGYVWYKGQIMDLQHYITAYTTDTEMIIFGPEYSGRETVYSNASLLIQSVTKNDTGSYTIQIIKRGHRTEGETGHYTLY

Pan_PSG12N QVTIEAQPTKVSEGNDVLLLVHNLPTNVVGYIWYKGQIMDLQHYITAYTTDTEMIIFGPAYSGRETVYSNASLLIQSVTKNDTGSYTIQIIKRGHRTEGVTGHYTLY

Pan_PSG7N QVTIEAQPNKVSEGKDVLLLVHNLPQNLTGYSWYKGQIMDLHHYIAAYTTDTEMIIFGPEYSGRETVYSNASLLIQNVTKNDTGSYTIQITKRGDETKGVTGHFTLY

Pan_PSG5N QVTIEAQPTKVSEGKDVLLLVHNLPQNVAGYSWYKGQIMDLHHYITAYTIDTEMIIVGPAYSGRETVYSNASLLIQNVTRKDTGSYTIQIIKRGDGTKRVTGHFTLY

Pan_PSG16N QVMIEAQPTKVSEGKDVLLPVRNLPQEVAAYIWYKGQIMDFHQFITAYTIDTERIIFGPAYSGRETVYSNGSLLIRNVTKNDTGSYTVKIRKPAEETKGVTVHFTLY

Pan_PSG8N QVTIEAQPTKVSEGKDVLLLVHNLPQNLTGYIWYKGQKTDLHLYVTSYVKDTETVIAGPAYSGRETVYSNASLLIQNVTQKDTGSYTIEITKQGDRTEGETGHYTLY

Pan_PSG6N QITIEAQPNKVSEGKDVLLLVHNLPQNLTGYIWYKGQKTDHHHYITSYVIDTETIIFGPAYSERETVYSNASLLIQNVTKNDTGSYTIQIIKRGDRTEGVTGHYTLS

Pan_PSG9N QVTIEAQPTKVSEGKDVLLLVHNLPQNLTGYIWYKGQKTDHHHYITSYVIDTETIIFGPAYSERETVYSNASLLIQKVTKNDTGSYTIQIIKRGDRTEGVTGHYTLY

Pan_PSG11N QVTIEAQPTKVSEGKDVLLLVQNLPQNLTGYVWYKGQKTDLHQFITAYTIDTETIISGPAYSGRETVYSNASLLIQNVTRNDTGSYTVQIIQRGDKIKRITGHFTLY

Pan_PSG15N QVTIEAQPTKVSEGKDVLLLVQNLPQNLTGYVWFKGQKTNFHQFIIAYKIDSKKITIGPAYSGRERVYSNASLLIQNVTRKDTGSYTIQIIQRGDKITGVTGHFTLY

**Pig-tailed macaque** 10 20 30 40 50 60 70 80 90 100

| | | | | | | | | |

Mne_PSG4aN QVTIEAQPTKVSEGKDVLLLVHNLPQNLTGYSWYKGQIIDLQHYITAYTIDTEMIVFGPVYSGRETVYSNASLLIQNVTKNDTGSYTIQITKRGDETKGLTGHFTLY

Mne_PSG4bN QVTIEAQPTKVSEGKDVLLLVHNLPQNLTGYSWYKGQIIDLQHYITAYTIDTEMIVFGPAYSGRETVYSNASLLIQNVTKNDTGSYTIQITKRGDETKGVTGHFTLY

Mne_PSG5N QVMIEAQPTKLSEGKDVLLLVYNLPQNLTGYSWYKGQIMDLQHYIAAYTIDTEMIVFGPAYSGRETVYSNASLLIQNVTKNDTGSYTIQITKQGDETKGVTGHFTLY

Mne_PSG11N QVTIEAQPAKVSEGKDVLLLVHNLPQNIAGYIWYKGQIMDLQHYITAYTIDTEMIIFGPAYSGRETVYSNASLLIQNVTKNDTGSYSIQITNPCDETKAVTGHFTLY

Mne_PSG15N QVTIEAQPAKVSEGKDVLLLVHNLPQNVAGYSWYKGQIMDLQHYITSYVIDTEIIIFGPAYSGRETVYSNASLLIQNVTQKDTGSYTIQITKRGDGTKRVTGHFTLY

Mne_PSG16N QVTIEAQPTEVSEGKDVLLLVHNLPQNPTGYSWYKGQITDIHHYITSYVIDTEMIVFGPAYSGRETVYSNASLLIQNVTQKDTGSYTIQIIQRSDTTKGVTGHFTLY

Mne_PSG18N QVTIEAQPTEVSEGKNVLLLVHNLTQNPTGYIWYKGQITDSHNYITSYVIDTEMIIFGPAYSGRETVYSNASLLIQNVTRKDTGSYTIQIIKRGDSTKGVTGHFTLY

Mne_PSG1N QVTIEAQPTKVSEGKDVLLLVHNLPQNLAAYIWYKGQIMDLHHYITSYVTDTEIIVFGPAYSGRETVYSNASLLIQNVTQKDTGSYTIQIIKRGDTTKGVTGHFTLY

Mne_PSG14N QVTIEAQPTKVSEGKDVLLLVQNLPENLTGYVWFKGQIMDFHQFITAYTIDTETIIFGPAYSGRETVYSNASLLIQNVTQNDTGSYTIEIIKRGDKIKGVTGHFTLY

Mne_PSG21N QVTIEAQPAKVSEGKDVLLLVQNLPQNLTGYVWFKGQITTFHQFIIAYKIDSRKITVGPAYSGRERVYSNASLLIQNVSRKDTGSYSIQIIKRGDKIKGVTGHFTLY

Mne_PSG6N QVTIEAQPTKVSEGKDVLLLVHNLPQNLTGYVWYKGQIMDLHQFITAYTIDTETIIFGPAYSGRERVYSNASLLIQNVTRKDTGSYTIQIIKRGDKIKRITGHFTLY

Mne_PSG10N QVTIEAQPTKVSEGKDVLLLVHNLPQNLTGYIWYKGQKTDLHLYVTSYVKDTETVIAGPAYSGRETVYSNASLLIQNVTQKDTGSYTIQITKRGDRTEGETAHFTLY

Mne_PSG13N QVTIEAQPTKVSEGKDVLLLVHNLPQNLTGYIWYKGQKTDLHLYVTSYVKDTETVIAGPAYSGRETVYSNASLLIQNVTKKDTGSYTIQIKKRGDRTEGETAHFTLY

Mne_PSG17N QVTIEAQPTKVSEGKDVLLLVHNLPQNLTGYIWYKGQKTDHYLYITSYVIDAETIIFGPAYSGRETVYSNASLLIQNVTWKDTGSYTIEIIKRGDRTEGVTGHYTLY

Mne_PSG9N QVTIEAQPTKVSEGKDVLLLVHNLPQNLIAYIWYKGQKTDFRHYITSYVIDAETIIVGPAYSGRETVYSNASLLIQNVTKNDTGSYTIQMIKQGDKTKGVIGHFTLY

Mne_PSG3N QVTIEAQPNKVSEGKDVLLLVHNLPQNLTGYIWYKGQIMDLQHYITSYVIDTETIIFGPAYNERETVYSNASLLIQNVTKNDTGSYTIQIIKRGDRTEGVTGHYTLY

Mne_PSG2N QVTIEAQPAKVSEGKDVLLLVHNLPQNLAACIWYKGQIMDLQHYITAYVIDAETIIFGPAYSGRETVYSNASLLIQNVTQKDTGSYTIQIIQRGDKTKGVTGHFTLY

Mne_PSG8N QVTIEAQPTKVSEGKDVLLLVHNLPKNIAGYIWYKGQIMDLQHYITAYTTDTEMILFGPAYSGRETVYSNASLLIQSVTKNDTGSYTIQIIKRGHRTEGVTGHYTLY

Mne_PSG7N QVTIEAQPTNISEGNDVLLLVHNLPKNPAAYIWYKGQIMDLHHYITAYTIDTERIIFGPAYSRRERVYSNASLLIQSVNQKDAGSYTVKIIKRGDGTEGVTGHFTLY

Mne_PSG20N QVTIEAEPTNIYEGNDVLLLVHNLTKNPAAFIWYKGQIMDRHHYITAYIIATESIIFGPAYSGRERVYSNASLLIQSVNQKDAGSYTVKIIKRGYKTEGVTGHFTLY

Mne_PSG12N QVTIEAQPTKVSEGKDILLLVHNVPQNVAGYIWYKGQIMDLQHYITAYAIDTETIIFGPVYSGRETIYSNASLLIQSVNQKDAGSYTVEIVKRGEGTEGVTGHFTLY

Mne_PSG19N QVTIEAQPTKVSEGKDILLLVNNVPQNIAGYSWYKGQIMDLQHYITAYAIDTETIIFGPAYSGRETIYSNASLLIQSVNQKDAGSYTVEIIKRGEGTEGVTGHFTLY

**Proboscis monkey** 10 20 30 40 50 60 70 80 90 100

| | | | | | | | | |

Nla_PSG7N QVTIEAQPTKVSEGKDVLLLVHNLPQNVTGYVWYKGQIMDLYHYITAYIIDTEMIIFGPAYSGRETVYSNASLLIQNVTQNDTGSYTIQITQRGDGIKGVTGHFTLY

Nla_PSG8N QVTIEAQPTKVSEGKDVLLLVHNLPQNIAAYIWYKGQIMDLRHYITAYIIDTEMIILGPAYSGRETVYSNASLLIQNVTQNDTGSYTIQITQRGDGTKGVTGHFTLY

Nla_PSG13N QVTIEAQPAKVSEGKDVLLLVHNLPQNVTGYIWYKGQIMDLHHYITAYTIDTEIIISGPAYSGRETVYSNASLLIQNVTQNDTGSYTIQITQRGDGTKGVTGHFTLY

Nla_PSG2N QVMIEAQPTKVSEGKDVLLLVHNLPQNLTGYVWYKGQIMDYHHYITAYTIDTETTIFGPAYSGRETVYSNASLLIQNVTQKDTGSYTIQITQRGDGTKGVTGHFTLY

Nla_PSG5N QVTIEAQPTKVSEGKDVLLLVHNLPQNLTGYIWYKGQIMDLHQFITAYTIDTETIISGPAYSGRETVYSNASLLIQNVTQNDTGSYTIQIIKRGDKIKRVTGHFTLY

Nla_PSG9N QVMIEAQPTKVSEGKDVLLLVHNLPQNVAAYIWYKGQIIDFHHYITGYVMEAEANIFGPAYSGRETVYSNASLLIQNVTQKDTGSYTIQIMKLGNRTEGVTGHFTLY

Nla_PSG10N QVMIEAQPTKVSEGKDVLLLVHNLPQNVAAYIWYKGQIIDFHHYITGYVMEAEANIFGPAYSGRETVYSNASLLIQNVTQKDTGSYTIQIMKLGNRTEGVTGHFTLY

Nla_PSG1N QVMIEAQPTKVSEGKNVLLLVHNLPQNVAAYIWYKGQIMDVHHYITGYVMEAETNIFGPAYSGRETVYSNASLLIQNVTQKDTGSYTIQIMKLGNRTEGVTGHFTLY

Nla_PSG11N QVMIEAQPTKVSEGKDVLLLVHNLPQNVAAYIWYKGQIMDLHHYITGYVIEAEAILFGPAYSERETVYSNASLLIQKVTQKDTGSYTIQIIKRGDKTKGVTGHFTLY

Nla_PSG16N QVTIEAQPTKVSEGKDVLLIVPNLPQNLIGYIWYKGQIMDLHHYIMGYVIEAEAIIFGPAYSGQETVYSNASLLIQNVTQNDTGSYTIQILKRGDKTKGVTGHFTLY

Nla_PSG17N QVMIEAQPTKVSEGKDVLLLVHNLPQNVAAYIWYKGQITDVHHYITGYVIDPETIIFGPAYTGRERLYSNASLLIQKVTQKDTGSYTIKITKRGDKTKGVTGHFTLY

Nla_PSG12N QVTIEAQPTKVSEGKDVLLLVHNLPQNLIGYIWYKGQVTDFHHYITSYVIDVETIIFGPAYSGRETVYSNASLLIQNVTWKDTGSYTIQIIKLGDRTKGVTGHFTLY

Nla_PSG14N QVTIEAQPTKVSEGKDVLLLVHNLPQNLIGYIWYKGQITDFHHYVTSYVIDAETTIFGPAYSGRETVYSNASLLIQNVTWKDTGSYTIQIIKQGDKTKGVTGHFTLY

Nla_PSG6N HVMIEAQPTEVYEGKDVLLLVHNLPQNPTGYIWYKGQITDIHNYITSYVIDTDTIIFGPAYSGRETVYSNASLLIQNVTQKDTGSYSIQIIKRGDSTKGVTGHFTLY

Nla_PSG3N QVTIEAQPTNVSEGNNVLLLVHNLPQNPAAYIWYKGQIMDLHHYITAYVIETERIVFGPAYSGRETVYSNASLLIQSVNQKDAGSYTVEIIKRGDGNEGVTGNFTLY

Nla_PSG4N QVMIEAQPNKVSEGKDVLLLVHNLPQNLAAYIWYKGQIMDLHHYITAYVIDTDTIIFGPAYSGRETVYSNASLLIQNVTQKDTGSYTIQIIKRGDRTEAVTEHFTLY

Nla_PSG15N QVTIEAQPTKVSEGKDVLLLVHNLPQNVVAYIWYKGQIMDLHHYITAYTIDTETIISGPVYSGREKIYSNASLLIQNVTKQDIRSYTIKIIKRGDETKGVTGNFTLY

Nla_PSG18N QVMIEAQPTKVSEGKDVLLLVHNLPQKVAAYVWYKGQIMDFYQFITAYSRDPDRILFGPAYSGRETLYSNGSLRIQNVTKQDTGSYTVKVMKRIDETKGVTGHFTLY

**Red guenon** 10 20 30 40 50 60 70 80 90 100

| | | | | | | | | |

Epa_PSG1N QVTIEAHPTKVSEGKDVLLLVHNLPQNLTGYIWYKGQIMDLQHYITAYTIDTEMIIFGSAYSGRETVYSNASLLIQNVTKNDTGSYTIQITKR-GDESKGVTGHFTLY

Epa_PSG2N QVTIEAQPTKVSEGKDVLLLVHNLPQNLTGYSWYKGQIMDLHHYIAAYIIDTEMIIFGPAYSGRETVYSNASLLIQNVTKNDTGSYTIQITKR-GDESKGVTGHFTLY

Epa_PSG3N QVTIEAQPTKVSEGKDVLLLVHNLPQNVAGYIWYKGQIMDLQHYITAYTIDTEMIVFGPAYTGRETVYSNASLLIQNVTQNDTGSYTIQITNR-CDETKGVTGHFALY

Epa_PSG4N QVMIEAQPTKVSEGKDVLLLVHNLPQNVAGYSWYKGQIMDLHHYITAYTIDTEMIIFGPAYSGRETVYSNASLLIQNVTQNDTGSYTIQITKR-GDGTKRVTGHFTLY

Epa_PSG5N QVTIEAQPTEVSEGKDVLLLVHNLPQNVAGYSWYKGQIMDLQHYITAYTIDTEMIIFGPAHSGRETVYSNASLLIQNVTQKDTGSYTIQIIKR-GDSTKRVTGHFTLY

Epa_PSG6N QVMIEAQPAKVSEGKDVLLRVHNLPQNLAAYIWYKGQIMDLQHYITAYVIDTEIIVFGPAYSGRETVYSNASLLIQNVTQKDTGSYTIQIIMR-GDRTKGVTGHFTLY

Epa_PSG17N QVVIEAQPAKVSEGKDVLLLVHNLPQNLAACIWYKGQIMDLQHYITAYVKDAETIIFGPAYSGRETVYSNASLLIQNVTRKDTGSYTIQIIKR-GDKTKGVTGHFTLY

Epa_PSG11N QVTIEAQPIEVSEGKDVLLLVHNLPQNPTGYIWYKGQITESHNYITSYVIDTEMIILVPAYSGRETVYSNASLLIQNVTQKDTGSYTIQIIKR-GDSTKGVTGHFTLY

Epa_PSG12N QVTIEAQPTEVSEGKDVLLLVHNLPQNPTGYSWYKGQITDIHHYITSYVIDTEIIIFGPAYSGRETVFSNASLLIQNVTQKDTGSYTIQIIQR-GDTTKGVTGHFTLY

Epa_PSG13N QVTVEAQPTKVSEGKDVLLLVHNLPQNLTGYIWYKGQITDHHHYITSYVIDTETIIFGPAYSGRETVYSNASLLIQNVTRNDTGSYTIQIIKR-GDRTEGITGHYTLY

Epa_PSG15N QVTIEAQPAKVSEGKDVLLLVHNLPQNLTGYIWYKGQKTDLHLYVTSYVKDTETIIAGPAYSGRETVYSNASLLIQNVTQKDTGSYTIQITKR-GDRTEGETAHFTLY

Epa_PSG21N QVTIEAQPTKVSERKDVLLLVHNLPQNLTGYIWYKGQKMDLHHYIISYVIDAETIIAGPAYSGREIVYSNASLLIQNVTRKDTGSYTIQTIKQ-GDNSKGVIGHFTLY

Epa_PSG7N QVTIEAQPTKVSEGKDVLLLVHNLPQNLIGYSWYKGQIMDLHHYITSYVIDTEMIVFGPAYSGRETVYSNASLLIQNVTRKDTGSYTIQIIKR-GDKIKRITGHYTLY

Epa_PSG9N QVRIEAQPTKVSEGKDILLLVHNLPQNVAGYIWYKGQIMDLQYYITAYAIDTEMIIFGPAYSGRETLYSNASLLIQSVNQKDAGSYTIEIIKR-GEGTEGVTGHFTLY

Epa_PSG10N QVRIEAQPTKVSEGKDILLLVHNLPQNVAGYIWYKGQIMDLQYYITAYAIDTEMLIFGPAYSGRETLYSNASLLIQSVNQKDAGSYTIEIIKR-GEGTEGVTGHFTLY

Epa_PSG8N QVTIEAQPTKVSEGKDILLLVHNLPQNVAGYIWYKGQIMDLQYYITAYAIDTEMLIFGPAYSGRETVYSNASLLIQSVNQKDAGSYTVEIIKR-GEVTEGVTGHFTLY

Epa_PSG14N QVRIEAQPTKVSEGKDILLLVHNLPQNVAGYIWYKGQIMDLQHCITAYTIDTETIIFGPAHSGRETVYSNASLLIQSVNQKDAGSYTVEIIIKQGDGTEGVTGHFTLY

Epa_PSG18N QVTIEAQPTNISEGNDVLLLVHNLPQNPAAYIWYKGQIMDLQHYITAYTIDTERIVFGPAYSGRERVYSNASLLIQSVNQKDAGSYTIEIIIKQGDRTERVTGHFTLY

Epa_PSG20N QVMIEAQPTKVSEGKDVLLLVRNLPQKVAAYVWYKGQIMDFHQFITAYTIDTERVIFGPACSGRETLYSNGSLLIRNVTKQDTGSYTVKIIER-AEETKGVTVHFTLY

Epa_PSG22N QVTIEAQPAKVSEGKDILLLVQNLPQNLTGYVWFKGQKTNYHQFIIAYAIDSKNITVGPAYSGRERVYSNASLLIQNVTQKVTGSYTIEIIKR-GDKIKGVTGHFTLY

Epa_PSG23N QVTIAAQPAKVSEGKDILLLVQNLPQNLTGYVWFKGQERNYHQFIIAYAIESKKITVGPAYSGRERAYSNASLLIQNVTRKDTGSYTIEIIKR-GDKIKGVTGHFTLY

Epa_PSG19N QVTIEAQPTKVSEGKDILLLVQNLPQNLTGYVWFKGQITNYHQFIIAYARDSKNITVGPAYSGRETVYSNASLLIQNVTQKDTGSYTIEIIKQ-GDETKGVTGHFTLY

Epa_PSG16N QVTIEAQPTKISKGKDVLLLVENLPQNLTGYVWFKGQIMDLHQFITVYTIDTETIILVPAYSGRETVYSNASLLIQNVTQNDTGSYTIEIIKR-GDKIKGVTGHFTLY

**Red-shanked** **douc** 10 20 30 40 50 60 70 80 90 100

**langur** | | | | | | | | | |

Pne_PSG1N QVTIEAQPTKVSEGKDVLLLVHNLPQNVTGYVWYKGQIMDLYHYITAYIIDTEMIIVGPAYSGRETVYSNASLLIQNVTQNDTGSYMIQITQRGDETKGVTGHFTLY

Pne_PSG4N QVTIEAQPTKVSEGKDVLLLVHNLPQNVTGYVWYKGQIMDLYHYITAYIIDTEMIIFGPAYSGRETVYSNASLLIQNVTQNDTGSYMIQITQRGDKTKGVTGHYSLY

Pne_PSG6N QVTIEAQPTKVSEGKDVLLLVHNLPQNVTGYVWYKGQIMDLYHYITAYVIDTEMIIFGPAYSGRETVYSNASLLIQSVTQNDTGSYTIQIAQRGDGTKGVTGHFTLY

Pne_PSG2N QVTIEAQPTKVSEGKDVLLLVHNLPQNIAAYIWYKGQIMDLRHYITAYIIDTEMIIVGPAYSGRETVYSNASLLIQNVTQNDTGSYTIQITQRGDGTKGVTGHFTLY

Pne_PSG5N QVTIEAQPTKVSEGKDVLLLVHNLPQNLAGYIWYKGKIMDLYHYITAYTIDTEMIIFGPAYSGRETVYSNASLLIQNVTQNDTGSYTIQIIKRGDKTKGVTGYFTLY

Pne_PSG9N QVTTEAQPTKVSEGKDVLLLVHNLPQNVVGYIWYKGQIMDLHHYITAYTIDTETIIFGPVYSGREKIYSNASLLIQNVTQNDTGSYTIQIIKRGDGTKGVTGHFTLY

Pne_PSG12N QVTIEAQPTKVSEGKSVLLLVHNLPQNLTGYIWYKGQIMDLHQFITAYTIDTETIISGPAYSGRETVYSNASLLIQNVTQNDTGSYTIQIIKRGEKIKRVTGHFTLY

Pne_PSG3N QVTIEAQPTKVSEGKDVLLLVHNLPQNLIGYIWYKGQIMDFHHYVTSYVIDAETTIFGPAYSGRETVYSNASLLIQNVTRKDTGSYTIQIIKQGDKTKGVTGHFTLY

Pne_PSG10N QVTIEAQPTKISEGKDVLLLVHNLPQNLIGYIWYKGQVMDFHHYITSYVIDAETIIIGPAYSGRETVYSNASLLIQNVTRKDTGSYTIQIIKLGDRTKGVTGHFTLY

Pne_PSG8N QVTIEAQPTKVSEGKDVLLLVHNLPQNLTGYVWYKGQIMDYHHYITAYVIDPETIIFGPAYSGRETVYSNASLLIQNVTRKDTGSYTIQIIKRGDSTKGVTGHFTLY

Pne_PSG11N HVMIEAQPNEISEGKDVLLLVHNLPQNPTGYIWYKGQITDIHNYITSYVIDTDTIIFGPAYSGRETVYSNASLLIQNVTQKDTGSYTIQIIKRGDSTKGVTGHFTLY

Pne_PSG13N DVMIEAQPTEVSEGKDVLLLVHNLPQNPTGYIWYKGQITDIHNYITSYVIDTDTIIFGPAYSGRETVYSNASLLIQNVTQKDTGSYTIQIIKRGDSTKGVTGHFTLY

Pne_PSG7N QVMIEAQPTKVSEGKDVLLLVHNLPQNLAAYIWYKGQIMDLHHYITAYVIDTDTIIFGPAYSGRETVYSNASLLIQNVTQKDTGSYTIQIIKRGDRTEAVTGHFTLY

Pne_PSG14N QVTIEAHPTNVSEGNDVLLLVHNLPQNPAAYIWYKGQIMDLHHYITAYVIETERIVFGPAYSGRETVYSNASLLIQSVNQKDAGSYTVEIIKRGDGNEGVTGNFTLY

Pne_PSG15N QVMIEAQPTKVSEGKDVLLLVHNLPQNVAAYIWYKGQIGDLHHYIMRYVIEAEAILFGPAYSGRETVYSNASLLIQNVTQKDTGSYTIQIIKRGDKTKGVTGHFTLY

Pne_PSG16N QVIIEAQPTKVSEGKDVLLLVHNLPQNVAAYIWYKGQITDVHHYITGYVIDPETIIFGPAYTGRERLYSNASLLIQKVTQKDTGSYTIKITKRGDKTKGVTGHFTLY

Pne_PSG17N QVMIEAQPTKVSEGKDVLLLVHNLPQNPAAYVWYKGQIMDFYQFIIAYTRYPDRILFGPAYSGRETLYSNGSLLIQNVTKQDTGSYTVKIMKRIDETKGVTGHFTLY

Pne_PSG18N QVMIEAQPTKVSEGKDVLLLVHNLPRNVAAYVWYKGQIMDFYQFITAYSRDPDRILFGPAYSGRETLYSNGSLRIQNVTKQDTGSYTVKVMKRIDETKGVTGHFTLY

**Rhesus macaque** 10 20 30 40 50 60 70 80 90 100

| | | | | | | | | |

Mml_PSG2N QVTIEAQPTKVSEGKDVLLLVHNLPQNLAAYIWYKGHIMDLHHYITSYVIDTEIIVFGPAYSGRETVYSNASLLIQNVTQKDTGSYTIQIIKRGDTTKGVTGHFTLY

Mml_PSG3N QVTIEAQPTKVSEGKDVLLLVHNLPQNVAGYSWYKGQIMDLHHYITSYVIDTEIIIFGPAYSGRETVYSNASLLIQNVTQKDTGSYTIQITKRGDGTKRVTGHFTLY

Mml_PSG8N QVTIEAQPTEVSEGKDVLLLVHNLPQNPTGYSWYKGQITDIHHYITSYVIDTEMIVFGPAYSGRETVYSNASLLIQNVTQKDTGSYTIQIIQRGDTTKGVTGHFTLY

Mml_PSG4N QVTIEAQPAKVSEGKDVLLLVHNLPQNVTGYIWYKGQKTDHHLYITSYVIDAETIILGPAYSGRETVYSNASLLIQNVTWKDTGSYTIEIIKRGDRTEGVTGHYTLY

Mml_PSG13N QVTIEAQPAKVSEGKDVLLLVHNLPQNLTGYIWYKGQKTDLHLYVTSYVKDTETVIAGPAYSGRETVYSNASLLIQNVTQKDTGSYTIQITKRGDRTEGETAHFTLY

Mml_PSG5N QVTIEAQPTKVSEGKDVLLLVHNLPQNLTGYIWYKGQIMDHHHYITSYVIDTETIIFGPAYNERETVYSNASLLIQNVTKNDTGSYTIQIIKRGDRTEGVTGHYTLY

Mml_PSG15N QVTIEAQPTKVSEGKDVLLLVQNLPQNLIAYIWYKGQKTDFHHYITSYVIDAETIIVGPAYSGRETVYSNASLLIQNVTQNDTGSYTIQMIKQGDKTKGVIGHFTLY

Mml_PSG20N QVTIEAQPAKVSEGKDVLLLVQNLPQNLIAYIWYKGQKTDFHHYITSYVIDAETIIVGPAYSGRETVYSNASLLIQNVTKNDTGSYTIQMIKQGDKTKGVIGHFTLY

Mml_PSG1N QVTIEAQPAKVSEGKDVLLLVHNLPQNLAACIWYKGQIMDLQHYITAYVIDAETIIFGPAYSGRETVYSNASLLIQNVTQKDTGSYTIHIIQRGDKTKGVTGHFTLY

Mml_PSG10N QVTIEAQPAKVSEGKDVLLLVQNLPENLTGYVWFKGQIMDFHQFITAYTIDTETIIFGPAYSGRETVYSNASLLIQNVTQNDTGSYTIEIIKRGDKIKGVTGHFTLY

Mml_PSG21N QVTIEAQPAKVSEGKDVLLLVQNLPQNLTGYVWFKGQITTFHQFIIAYKIDSRKITVGPAYSGRERVYSNASLLIKNVSRKDTGSYTIQIIQRGDKTKGVTGHFTLY

Mml_PSG14N QVTIEAQPTKVSEGKDVLLLVHNLPQNLTGYVWYKGQIMDLHQFITAYTIDTDTIIFGPAYSGRERVYSNASLLIQNVTRKDTGSYTIQIIKRGDKIKRITGHFTLY

Mml_PSG17N QVTIEAQPAKVSEGKDVLLLVHNLPQNLTGYSWYKGQIIDLQHYITAYTIDTEMIVFGPAYSGRETVYSNASLLIQNVTKNDTGSYTIQITKRGDETKGLTGHFTLY

Mml_PSG18N QVTIEAQPAKVSEGKDVLLLVHNLPQNLTGYSWYKGQIIDLQHYITAYTIDTEMIVFGPAYSGRETVYSNASLLIQNVTKNDTGSYTIQITKRGDETKGLTGHFTLY

Mml_PSG6N QVMIEAQPTKVSEGKDVLLLVYNLPQNLTGYSWYKGQIMDLQHYIAAYTIDTEMIVFGPAYSGRETVYSNASLLIQNVTKNDTGSYTIQITKQGDETKGVTGHFTLY

Mml_PSG9N QVTIEAQPTKLSEGKDVLLLVHNLPQNLTGYSWYKGQIIDLQHYITAYTIDTEMIVLGPAYSGRETVYSNASLLIQNVTKNDTGSYTIQITKRGEGTEGVTGHFTLY

Mml_PSG11N QVTIEAQPTNISEGNDVLLLVHNLPQNPAAYIWYKGQIMDLHHYITAYTIDTERIIFGPAYSGRERVYSNASLLIQSVNQKDAGSYTVKIIKQGDGTEGVTGHFTLY

Mml_PSG19N QVTIEAEPTNISEGNDVLLLVHNLPKNPAAYIWYKGQIMDLQNYITAYTIDTERIILGPAYSGRERVYSNASLLIQSVNQKDAGSYTVKIIKRGYRTEGVTGHFTLY

Mml_PSG7N QVTIEAQPTKVSEGKDILLLVHNVPQNVAGYIWYKGQIMDLQHYITAYAIDTETIIFGPVYSGRETIYSNASLLIQSVNQKDAGSYTVEIVKQGEGTEGVTGHFTLY

Mml_PSG12N QVTIEAQPTKVSEGKDVLLLVHNLPTNVVGYIWYKGQIMDLQHYITAYTTDTEMILFGPAYSGRETVYSNASLLIQSVTKNDTGSYTIQIIKRGHRTEGVTGHYTLY

Mml_PSG16N QVTIEAQPTKVSEGKDVLLLVHNLPTNVVGYIWYKGQIMDLQHYITAYTTDTEMIIFGPAYSGRETVYSNASLLIQSVTKNDTGSYTIQIIKRGHRTEGVTGHFTLY

**Sooty mangabey**  10 20 30 40 50 60 70 80 90 100

| | | | | | | | | |

Cat_PSG12N QVTIEAQPTKVSEGKDVLLLVQNLPQNLIAYIWYKGQKTDFRHYITSYVIDAETIIVGPAYSGRETVYSNASLLIQNVTQKDTGSYTIQMIKQGDKTKGVIGHFTLY

Cat_PSG18N QVTIEAQPAKVSEGKDVLLLVHNLPQNLAACIWYKGQIMDLQHYITAYVIDAETIIFGPAYSGRETVYSNASLLIQNVTQKDTGSYTIQIIQRGDKTKGVTGHFTLY

Cat_PSG3N QVTIEAQPTKVSEGKDVLLLVHNLPQNLAAYIWYKGQIMDLHHYITSYVIDTEIIVFGPAYSGRETVYSNASLLIQNVTQKDTGSYTIQIIQRGDTTKGVTGHFTLY

Cat_PSG9N QVMIEAQPTKDSEGKDVLLLVHNLPQNLTGYIWYKGQKTDHHHYITSYVIDTETIIFGPAYSERETVYSNASLLIQNVTKNGTGSYTIQIIKRGHRTEGVTGHYTLS

Cat_PSG19N QVTIEAQPTKVSEGKDVLLLVHNLPQNLTGYIWYKGQITDHHHYITSYVIDTETIIFGPAYSERETVYSNASLLIQNVTQNDTGSYTIQIIKRGHRTEGVTGHYTLY

Cat_PSG6N QVTIEAQPAKVSEGKDVLLLVHNLPQNLTGYIWYKGQKTDHHLYITSYVIDTETIIFGPAYSGRETVYSNASLLIQNVTRKDTGSYTIEIIQRGDRTEGVTGHYTLY

Cat_PSG7N QVRIEAQPTKVSEGNDVLLLVHNLPQNLTGYIWYKGQKTDLHLYVTSYVKDTETVIAGPAYSGRETVYSNASLLIQNVTQKDTGSYTIQITKRGDRTEGETGHFTLY

Cat_PSG8N QVTIEAQPTEVSEGKDVLLLVHNLPQNPTGYSWYKGQITDIHHYITSYVIDTEMIVFGPAYSGRETVYSNASLLIQNVTQKDTGSYTIQIIQRGDTTKGVTGHFTLY

Cat_PSG11N QVTIEAHPTEVSEGKDVLLLVHNLPQNPTGYIWYKGQITDSHNYITSYVIDTEMIIFGPAYSGRETVYSNASLLIQNVTRKDTGSYTIQIIKRGDTTKGITGHYTLY

Cat_PSG14N QVTIEAQPAKVSEGKDVLLLVHNLPQNLTGYVWFKGQITSFHQFIIAYKIDSKKITVGPAYSGRERVYSNASLLIQNVTQKDTGSYTIQIIKRGDKIKGVTGHFTLY

Cat_PSG20N QVTIEAQPAKVSEGKDVLLLVQNLPENLTGYVWFKGQIMDFHQFITAYTIDTETIIFGPAYSGRETVYSNASLLIQNVTQNDTGSYTIEIIKRGDKIKGVTGHFTLH

Cat_PSG1N QVTIEAQPTKVSEGKDVLLLVHNLPQNLTGYSWYKGQIMDLQHYITAYTIDTEMIVFGPAYSGRERVYSNASLLIQNVTKNDTGSYTIQITKRGDETKGVTGHFTLY

Cat_PSG2N QVTIEAQPTKVSEGKDVLLLVYNLPQNLTGYRWYKGQIMDLQHYIAAYTTDTEMIIFGPAYSGRETVYSNASLLIQNVTKNDTGSYTIQITKRGDGTKGVTGHFTLY

Cat_PSG16N QVTIEAQPAKVSEGKDVLLLVHNLPQNVAGYSWYKGQIMDLHHYITAYTTDTEMIIFGPAYSGRETIYSNASLLIQNVTQKDTGSYTIQITKRGDGTKRVTGHFTLY

Cat_PSG5N QVTIESQPTKVSEGKDVLLLVHNLPTNVVGYIWYKGQIMDLQHYITAYTTDTEMIIFGPAYSGRETIYSNASLLIQSVTKNDTGSYTIQIIKRGHRTEGVTGHYTLY

Cat_PSG17N QVTIEAQPTKVSEGKDVLLLVHNLPTNVVGYIWYKGQIMDLQHYITAYTTDTEMIIFGPAYSGRETIYSNASLLIQSVTKNDTGSYTIQIIKRGHRTEGVTGHYTLY

Cat_PSG10N QVTIEAQPTNISEGNDVLLLVHNLPKNPAAYIWYKGQIMDLQHYITAYTIYTERIIFGPAYSGRETVYSNASLLIQSVNQKDAGSYTVKIIKRGYRTEGVTGHFTLY

Cat_PSG22N QVTIEAQPTNISEGNDVLLLVHNLPKNPAAYIWYKGQIMDLQHYITAYTIDTERIILGPAYSGRERVYSNASLLIQSVNQKDAGSYTIKIIKRGYRTEGVTGHFTLY

Cat_PSG21N QVTIEAQPTNISEGNDVLLLVHNLPQNPAAYIWYKGQIMDLQDYITAYTIDTERIIFGPAYSGRERVYSNASLLIQSVNQKDAGSYTVKIIERGDGTEGVTGHFTLY

Cat_PSG13N QVMIEAQPTKVSEGKDVLLPVRNLPQKVAAYIWYKGQIMDFHQFITAYTIDTERIIFGPAFSGRETLYSNGSLLIRNVTKNDTGSYTVKIRNPAEETKGVTVHFTLY

Cat_PSG15N QVMIEAQPTKVSEGKDVLLLVRNLPQKVAAYVWYKGQIMDFHQFITAYTIDPERIIFGYAYSGRETLYSNASLLIRNVTKQDTGSYTVKIMNRMEETKGVTVHFTLY

Cat_PSG4N QVTIEAQPTKVSEGKDVLLLVHNLPTKVAGYIWYKGQIMDLQHYITAYTIDTEKIIFGPAYSGRETVYSNASLLIQNVTKNDTGSYSIQITNPCDETKGITGHFTLY

**Ugandan red colobus** 10 20 30 40 50 60 70 80 90 100

| | | | | | | | | |

Pte_PSG7N QVTIEAQPTNVSEGNNVLLLVHNLPQNPAAYIWYKGQIMDLHHYITAYVIETERIVFGPAYSGRETVYSNASLLIQSLNQKDAGSYTVEIIKRGDGNEGVTGNFTLY

Pte_PSG9N QVTIEAQPTKVSEGKDVLILVHNLPQNVVGYIWYKGQIMDLHHYITAYTIDTETIILGSAYSGRETVYSNASLLIQSVTKQDTGSYTIKIIKRGDGNEGVTGHFTLY

Pte_PSG1N QVTIEAQPTTVSEGKDVLLLVHNLPQNVTGYIWYKGQIMDLHHYITAYTIDTETIILGPAYSGREAIYSNASLLIQNVTQNDTGSYTIQITQRGDGTKGVTGHFTLY

Pte_PSG2N QVMIEAQPTKVSEGKDVLLLVHNLPQNVTGYIWYKGQIMDLHHYITSYVIDTEMIILGPAYSGRETVYSNASLLIQNVTRNDTGSYTIQMTQRGDGTKGVTGHFTLY

Pte_PSG3N QVTIEAQPTKVSEGKDVLLLVHNLPQNLAGYIWYKGQIMDLHHYLTAYVIDTEMIILGPAYSGRETIYSNASLLIQNVTQNDTGSYTIQFIKRGDKTKGVTGHFTLY

Pte_PSG4N QVLIEAQPNKVSEGKDVLLLVHNLPQNLAAYIWYKGQIMDLHHYITAYVIDTEIIIYGPAYSGRETIYSNASLLIQNVTRNDTGSYTIQTIKRGDSTKGVTGHFTLY

Pte_PSG10N QVMIEDQPTEVSEGKDVLLLVHNLPQNPTGYIWYKGQITDIHNYITSYVIDAEMIIFGPAYSGRETVYSNASLLIQNVTQKDTGSYNIQITKRGDSTKGVTECFTLY

Pte_PSG11N EVMIEAQPTKVSEGKDVLLLVHNLPQNVAAYIWYKGQITDVHHYITGYVIDPETIIFGPAYTGRERVYSNASLLIQKVTQKDAGSYTIKITKRGDKTKGVTGHFTLY

Pte_PSG5N QVTIEAQPTKVSEGKDVLLLVHNLPQNPIGYIWYKGQIMDIHHYITSYVIDAETIISGPAYSGRETVYSNASLLIQNVTQNDTGSYTIQIMKLGDKTEGLTGHFTLY

Pte_PSG6N QVTIEAQPTKVSEGKDVLLLVHNLPQNLTGYIWYKGQIMDYHHYITSYVIDPETIIFGPAYSGRETVYSNASLLIENVTRNDTGSYTIKIIKRGDRTEGITGHFTLY

Pte_PSG8N QVMIETQPTKVSEGKDVLLLVHNLPPNPAAYVWYKGQIMDFNQFIIAYTTYPDRILFGPASSGRETLYSNGSLGIQNVTKQDTGSYTIRVMKRMDGTKGVTGHFTLY

**New World monkeys**

**Black-handed** 10 20 30 40 50 60 70 80 90 100

**spider monkey** | | | | | | | | | |

Age_PSG2N QIIIEAQPHVVSEGKDVLLLVHNLPQNPAGYSWYRGKITDIDHYIAAYLTRAQICISGPAYSGRETIYSNASLLIQNVTQNDAGSYILQVAMRGDRNKGVTGHFTLH

Age_PSG3N QLMIEAQPHVVSEGKDVLLLVHNLPQNPAGYSWYRGKIMDIDHYIAAYLTRAEICISGPADSGRETVYPNASLLIQNVTQNDAGSYILQVAMLGDWIKRVTGHFTLH

Age_PSG1N QVMIEAQPHVVSEGKDVLLLVHNLPQNLTGYSWYRGKIMDINHYLTAYLIDTQITITGPAYSGRETIYSNASLLIQNVTQNDAGSYILQVAMRGDRNKGVTGHFTLH

**Bolivian titi** 10 20 30 40 50 60 70 80 90 100

| | | | | | | | | |

Pdo_PSG1N QVTIEAQPHIVSEGKDVLLLVHNLPQNLTGYSWYRGKVMDIHHYLTAYLIEKKIIIYGHAYSGRETIYSNASLLIQNVTLNDTGSYTLQVVNQGERNKGVTAHFTLH

Pdo_PSG3N QVTIEAQPHIVSEGKDVLLLVHNLPQNLTGYSWYRGKVMDIHHYITAYLIEKEIIIYGHAYSGRETIYSNASLLIQNVTLNDTGSYTLQVVNQGERNKGVTAHFTLH

Pdo_PSG2N QVRIEAQPHIVSEGKDVLLLVRNLPQNIIGYSWYRGKVMDIQHYITAYLIGTQVIIYGPAYSGRETLYSNASLLIQNVTQNDAGSYTMKVINQGERNKGVTAHFTLH

**Mantled** **howler** 10 20 30 40 50 60 70 80 90 100

**monkey** | | | | | | | | | |

Apa_PSG1N QVMIEAQPHVVSEGKDVLLLVHNLPQNLTGYRWYRGKIMDINHYLTAYLIDTQISISGPAYSGRETIYSNASVLIQNVTQNDTGSYTLQVSTRGDRNKGITGHFTLH

Apa_PSG4N QVMIEAQPHIVSEGKDVLLLVHNLPQNLTGYRWYRGKIIDINHYLAAYLIDTQISISGPAHSGRETIYPNASLLIQNVTQNDTGSYTLQVSTQGDRNKGVTGHFTLH

Apa_PSG3N QVMIEAQPHVVSEGKDVLLLVHNLPQNPTGYSWYRGKIMDINHYLAAYLIDTQISISGPAHSGRETIYPNASLLIQNVTQNDAGSYTLQVSTQGDRNKGVTGHFTLH

Apa_PSG2N QVMIEAQPHVVSEGKDVLLLVHNLPQNPTGYSWYRGKIMDINHYLAAYLTDTQISIFGPAYSGRETIYPNASLLIQNVTQNDTGSYTLQVAMPGDRNKGVTRHFTLH

Apa_PSG5N QVMIEAQPHVVSEGKDVLLLVHNLPQNPTGYSWYRGKIMDINHYLAAYLTDTQIRISGPAHSGREIIYSNASLLIQNVTQNDAGSYTLQVAMPGDRNKGVTGHFTLH

Apa_PSG6N QVMIEAQPHVVSEGKDVLLLVHNLPKNPTAYGWFRGNITDIDHYIAAYLTGTQKSISGPAYSGREKIYHNASLLIRNVTQKDAGSYILHVAVRGDWIKRVIGHFTLH

Apa_PSG7N KLMIEAQPHVVSEGKDVLLRVHNLPKNPTAYGWFRGNITDIDHYIAAYLTHSEIGLTGPAYTGREEIYHNASLLIRKVTQKDAGSYILHIAVRGDWIKRVIGHFTLH

**Marmoset**  10 20 30 40 50 60 70 80 90 100

| | | | | | | | | |

Cja_PSG3aN QVMIEAQPHVVSEGKDVLLLVHNLPQNLIGYSWYRGKIMDINHYMTAFLIDRHITIFGPAHTGRETIYPNASLLIEKVTQNDAGPYTLQVITQGDRNNGETGHFTLH

Cja_PSG3bN QVMIEAQPHVVSEGKDVLLLVHNLPQNLIGYSWYRGKIMDINHYMTAFLIDRHITIFGPAHTGRETIYPNASLLIEKVTQNDAGPYTLQVATQGDRNNGETGHFTLH

Cja_PSG1N QVMIEAQPHVVSEGKDVLLLVHNLPQNLIGYSWYRGKIMDITHYMTAFLIDGHITIFGPAHTGRETIYPNASLLIEKVTQNDAGPYTLQVITQGDRNNGETGHFTLH

Cja_PSG4N QVMIEAQPHVVSEGKDVLLLVHNLPQNLIGYSWYRGKIMDINYYITAYLIETDITIFGPAHTGRETIYPNASLLIEKVTQNDAGPYTLQVITQGDRNNGETGHFTLH

Cja_PSG2N QVMIEAQPHVVSEGKDVLLLVHNLPQNSIGYSWFRGKIKDINYYITAYLIETDITIFGPAYTGRETIYPNASLLIETVTQNDAGPYTLQVITQGDRNNGETEHFTLH

**Ma’s night monkey**  10 20 30 40 50 60 70 80 90 100

| | | | | | | | | |

Ana_PSG1N QVMIEAQPHVVSEGKDVLLLVHNLPQNLTGYSWYRGKIKDINHYLTAYLIETREIILGPAYTGRETIYSNASLLIEKVTLNDTGSYTLQVTTHGDRNKGAIGHFTVY

Ana_PSG2N QVMIEAQPHVVSEGKDVLLLVHNLPQNLTGYSWYRGKIKDINHYLTAYLIDTHEIILGPAYSGRETIYSNASLLIENVTQNDTGSYTLQVTTHGDRNKRVTGHYTLH

Ana_PSG3N QVMIEAQPHVVSEGKDVLLLVHNLPQNLTGYSWYRGKIKDIHHYLTAYLIDTHEIIFGPAYSGRETIYSNASLLIEKVTLNDAGSYTLEVNTHGDRNKGVTRHFTLH

Ana_PSG4N QVMIEAQPHVVSEGKDVLLLVHNLPPNLIGYIWYRGKIKDNDHYLTAYLIDTHEIIFGPAYSGRETIYSNASLLIEKVTLNDAGSYTLQVTTHGDRNKGITRHFTLH

Ana_PSG5N QVMIEAQPHVVSEGKDVLLLVHNLPQNLTGYSWYRGKIKDISHYLTAYLIETREVIFGPAYSGREKIYSNASLLIKNVTLNDAGSYTLQVNTRGDINKGATGHFTLH

**Tamarin** 10 20 30 40 50 60 70 80 90 100

| | | | | | | | | |

Sim_PSG1N QVMIEAQPHVVSEGKDVLLLVHNLPQNPTGYSWYRGKIKDINHYITAYLIDTEITIFGPAHTGRETIYPNASLLIENVTQNDAGLYTLQVTTQGDRNKGETGHFTLH

Sim_PSG2N QVMIEAQPHVVSEGKDVLLLVHNLPQNLTGYSWYRGNIMDIKHYITAFLIERHITIFGPAHTGRETIYPNASLLIENVNHNDAGLYTLQVATQGDRNKGETGHFTLH

**White-faced saki**  10 20 30 40 50 60 70 80 90 100

| | | | | | | | | |

Ppi_PSG2N QVVIEAQPHVVSEGKDVLLLVHNLPQNLTGYSWYRGPIMDVHHYVTAYLIEKEVIIQGPAYSGRETIYSNASLLIQNVTQNDAGIYTLHITKQGEKNKGVTGHFILH

Ppi_PSG4N QVVIEAQPHVVSEGKDVLLLVHNLPQNLTGYSWYRGPIMDVHHYVTAYLIEKEVIIQGPAYSGRETIYSNASLLIQNVTQNDAGIYTLHVTKQGEKNKGVTGYFILH

Ppi_PSG1N QVVIEAQPHVVSEGKDVLLLVRNLPQNLTGYSWYRGPIMDIHHYVTAYLIEKEIIIQGPAYSGRETIYSNASLLIQNLTQNDAGIYTLQVTKQGERNKGVTGHFILH

Ppi_PSG3N QVVIEAQPHVVSEGKDVILLARNLPQNVFHYSWYRGTVMDINHYITAYLIGTEVIIYGPAYSGRETIYSNASLLIQNVTQNDAGTYILQVTKQGERSKGVTGHFILH

**White-fronted capuchin**

**Tufted capuchin** 10 20 30 40 50 60 70 80 90 100

| | | | | | | | | |

Cal_PSG1N QVMTEAQLRIFSEGKDVLLLVHNLPQNLTGYTWYKGKVMDIHHYLTAYLIDTQITIVGHAYSGRETIFSNASLLIENVTQNEAGPYILQVTKQGARNEGETGHFTLK

Sap_PSG1N QVMAEAQLRIVSEGKDVLLLVHNLPQNLTGYSWYRGKVMDIHHYLTPYLIDTQITIVGHAYSGRETIFSNASLLIENVTQNEGGPYILQVTKQGARNEGETGHFTLK

B


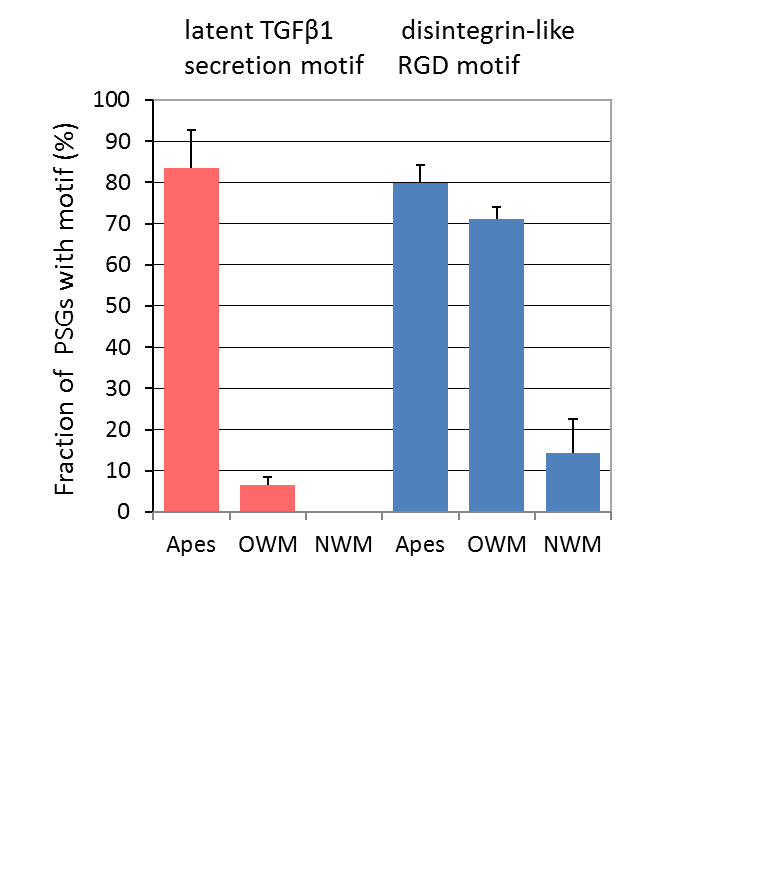

Supplement: Supplementary file 2 — Additional file 2: Supplementary Figure 2. Latent TGFβ1 secretion and putative disintegrin motifs in primate PSGs. (A) Amino acid sequences (one letter code) of mature N domains from ape, OWM and NWM species were aligned. Amino acids conserved in all PSG in a given species are shown in red, positions with conserved amino acid changes are shown in green, less conserved positions in blue. Non-conservative changes are shown in black. The LYHY motif shown to be responsible for latent TGFβ1 secretion is highlighted with filled-in red boxes, the putative disintegrin motifs with filled-in blue boxes. Disintegrin-like motifs with conservative amino acid changes are marked by blue open boxes. For the long form of the abbreviated Latin species names see Supplementary Table 1. (B) The fraction of PSGs with latent TGFβ1 secretion and disintegrin-like R/KGD/E motifs was calculated for each primate species and the means (± SEM) were plotted for apes, OWM and NWM. NWM, New World monkeys; OWM, Old World monkeys; PSG, pregnancy-specific glycoprotein; TGFβ1, tumor growth factor β1. [file 12864_2021_7413_MOESM2_ESM.docx]
