# Supplementary material for: The immune-modulating pregnancy-specific glycoproteins evolve rapidly and their presence correlates with hemochorial placentation in primates
Source: BMC Genomics. 2021 Feb 18;22:128. doi: 10.1186/s12864-021-07413-8 (PMC7893922; doi:10.1186/s12864-021-07413-8)
Supplement: Supplementary file 3 — Additional file 3: Supplementary Figure 3. IgC-type exon inclusion in human, rhesus and howler monkey PSG mRNAs. (A) a schematic exon organization of human, rhesus monkey and howler monkey PSG genes is shown. Two pairs of exons encoding IgC-like A- and B-type domains are present in primate PSG genes. Most of A1 and B2 exons contain intact consensus splice sites and open reading frames in transcribed human and rhesus monkey PSG genes. Due to the lack of PSG transcription information in howler monkey all exons of the Apa_PSG genes were analyzed. In contrast to A1 and B2 exons, only 1 out of 10, 7 out of 20, 2 out of 8 B1 exons in human, rhesus monkey and howler monkey PSG genes, respectively, exhibit both intact consensus splice sites and open reading frames. In rhesus monkey, only 4 out of 20 PSG contain intact A2 exons. However, these exons are not (B1 exons in human PSG) or rarely spliced-in (B1, in 2 out of 13, A2 in 4 out of 13 transcribed rhesus monkey PSG). (B) All human and rhesus monkey PSG transcripts encode functionally important N and B2 domains, conveying TGFβ1 secretion and TGFβ1 activation, respectively, while variably 1 or 2 but never 3 IgC-like domains seem to serve as “spacers” indicated by brackets. For howler monkey the domain organization of the expected largest PSG is shown. Apa, Alouatta palliata, howler monkey; Hsa, Homo sapiens, human; Mml, Macaca mulatta, rhesus macaque; NWM, New World monkey; ORF, open reading frame; OWM, Old World monkey; ss, splice site. [file 12864_2021_7413_MOESM3_ESM.pptx]

## Slide 1
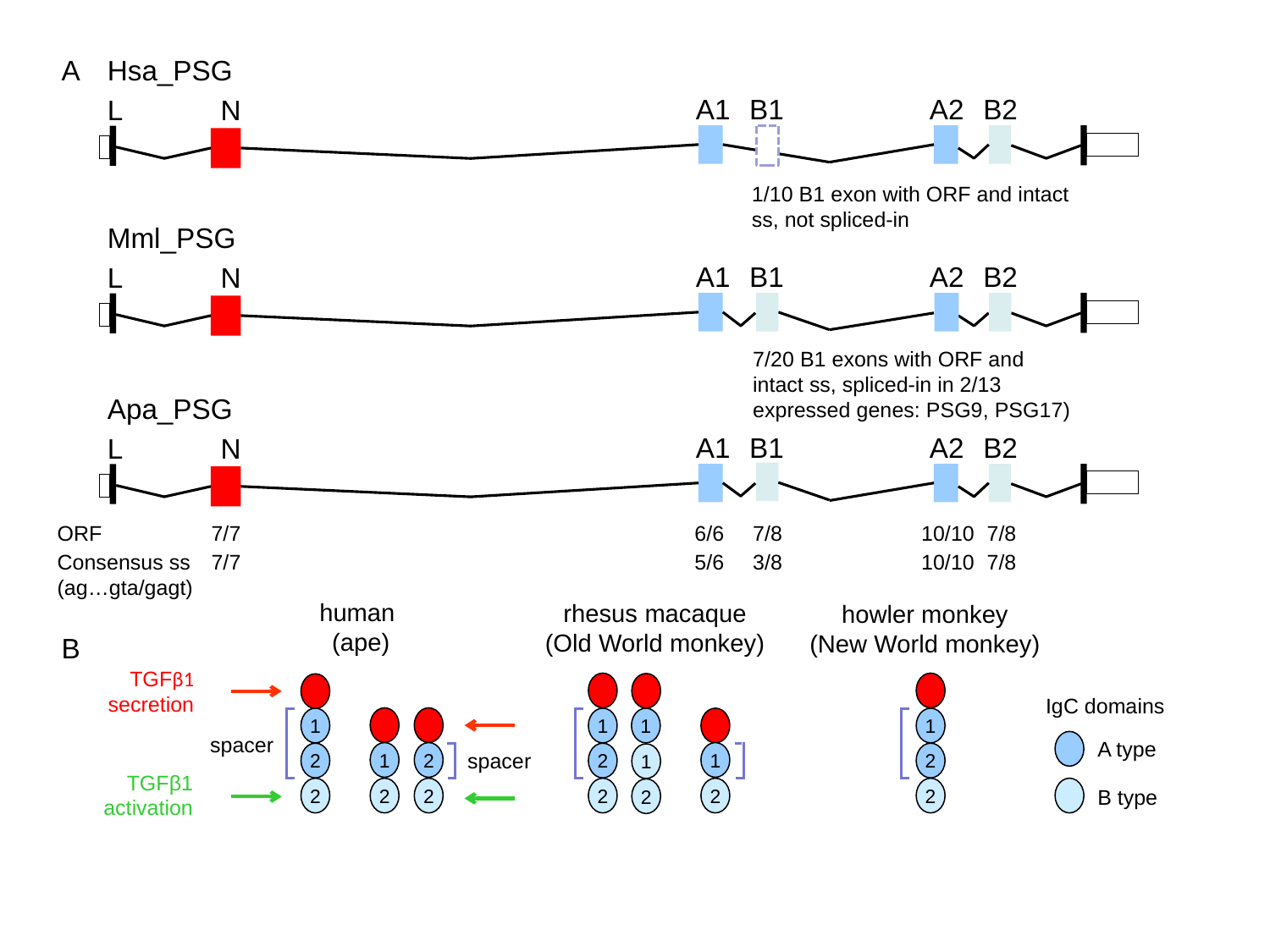

A
Hsa_PSG
A1
B1
A2
B2
L
N
1/10 B1 exon with ORF and intact ss, not spliced-in
Mml_PSG
A1
B1
A2
B2
L
N
7/20 B1 exons with ORF and intact ss, spliced-in in 2/13 expressed genes: PSG9, PSG17)
Apa_PSG
A1
B1
A2
B2
L
N
ORF
7/7
6/6
7/8
10/10
7/8
Consensus ss
(ag…gta/gagt)
7/7
5/6
3/8
10/10
7/8
rhesus macaque
(Old World monkey)
howler monkey
(New World monkey)
human
(ape)
B
TGFβ1
secretion
IgC domains
1
1
1
1
spacer
A type
spacer
1
2
1
2
2
2
1
TGFβ1
activation
B type
2
2
2
2
2
2
2
