# Supplementary figures and images for: The immune-modulating pregnancy-specific glycoproteins evolve rapidly and their presence correlates with hemochorial placentation in primates
Source: BMC Genomics. 2021 Feb 18;22:128. doi: 10.1186/s12864-021-07413-8 (PMC7893922; doi:10.1186/s12864-021-07413-8)

## Slide 1
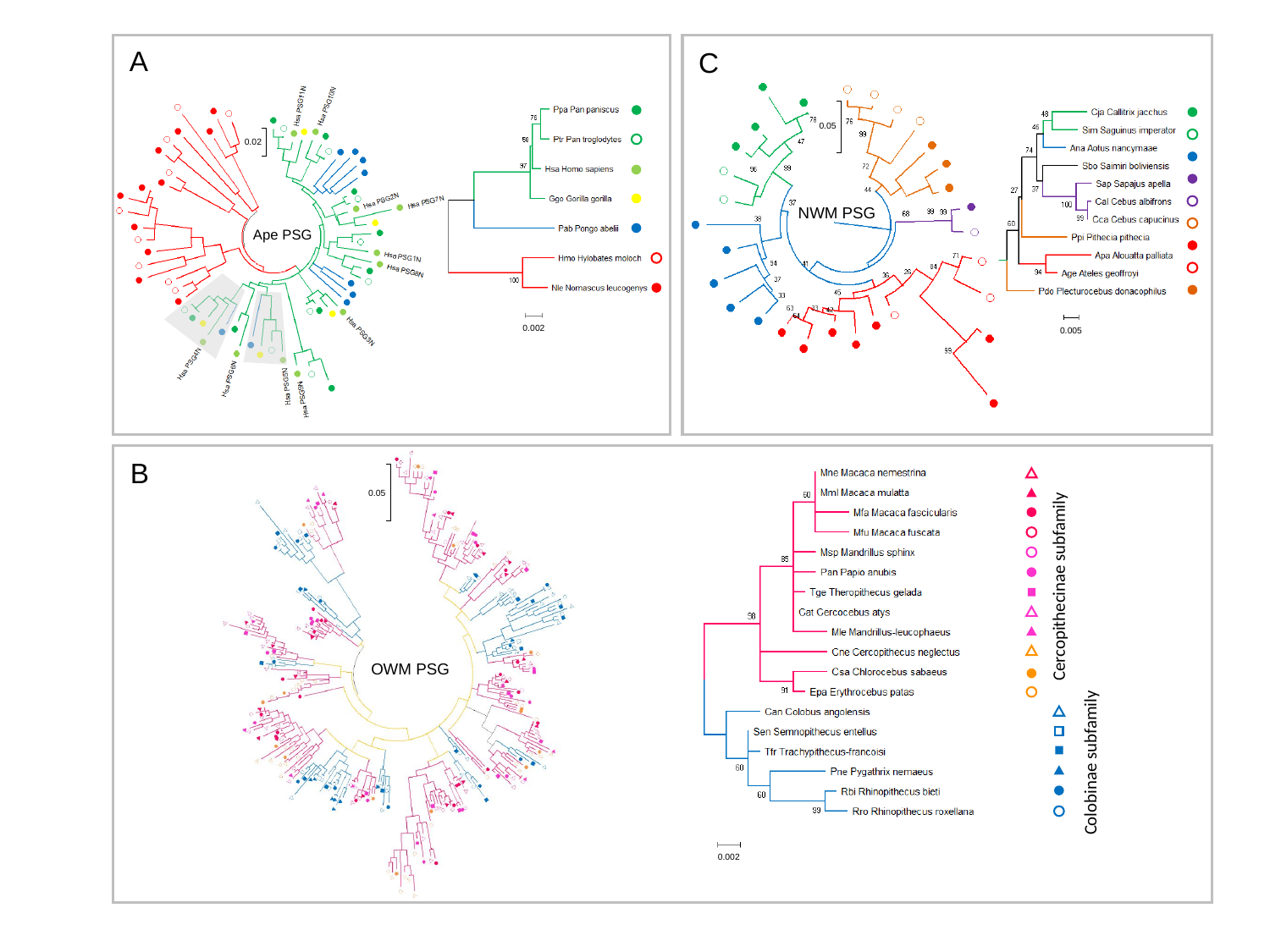

A
C
0.05
0.02
NWM PSG
Ape PSG
0.002
0.005
B
Cercopithecinae subfamily
0.05
OWM PSG
Colobinae subfamily
0.002

Supplement: Supplementary file 4 — Additional file 4: Supplementary Figure 4. Loss of orthologous relationship during ape, OWM and NWM PSG evolution. Phylogenetic trees were constructed based on N domain exons nucleotide sequences of PSG genes from great ape (A), OWM (B) and NWM (C) species using the Maximum Likelihood method (MEGA6 software). The trees with the highest log likelihood are shown. The percentage of trees in which the nucleotide sequences clustered together is shown next to the branches. Primate families/subfamilies and species can be identified by colored branches and colored symbols, respectively, shown next to the phylogenetic trees which were generated as described in Supplementary Figure 1. (A) Most of the human, bonobo, chimpanzee and gorilla (Homininae) PSG genes form orthologous clusters while only a few PSG genes within the great ape family exhibit an orthologous relationship (marked by gray trapezoids). Part of orangutan and most gibbon PSG genes cluster in a paralogous manner. (B) In OWM, PSG genes cluster according to the Colobinae (blue) and Cercopithecinae subfamilies (red colors). (C) With one possible exception (tufted capuchin, Sapajus capella; white-fronted capuchin, Cebus albifrons) NWM PSG genes form paralogous clusters. NWM, New World monkeys; OWM, Old World monkeys; PSG, pregnancy-specific glycoprotein. For common species names refer to Supplementary Table 1. [file 12864_2021_7413_MOESM4_ESM.pptx]
