# Supplementary material for: The immune-modulating pregnancy-specific glycoproteins evolve rapidly and their presence correlates with hemochorial placentation in primates
Source: BMC Genomics. 2021 Feb 18;22:128. doi: 10.1186/s12864-021-07413-8 (PMC7893922; doi:10.1186/s12864-021-07413-8)
Supplement: Supplementary file 5 — Additional file 5: Supplementary File 1 N exon nucleotide sequences of primate CEACAM genes. Contains nucleotide sequences of N domain exons and accession numbers of PSG genes of all primates analyzed. Of note: identical numbers in PSG gene names in different primates does not imply an orthologous relationship. [file 12864_2021_7413_MOESM5_ESM.docx]

**Supplementary File 1 - N exon nucleotide sequences of primate PSG genes**

ORF, open reading frame; PSGP, PSG pseudogene; WGS, whole genome shotgun

>Age_PSG1N (Ateles geoffroyi; black-handed spider monkey) WGS PVHS01054656.1 CGTCACTTTTAAACTTCTGGAACCCACCCACCACTGCCCAAGTCATGATTGAAGCCCAGCCACACGTTGTTTCAGAGGGGAAGGATGTTCTTCTACTTGTCCACAATTTGCCCCAGAATCTTACTGGCTACAGCTGGTACAGAGGGAAAATTATGGACATCAACCATTACCTTACAGCATATTTAATAGACACTCAAATAACTATAACTGGGCCTGCATACAGTGGACGAGAAACAATATATTCCAATGCATCCCTGCTGATCCAGAACGTCACCCAGAATGACGCAGGATCCTACATCCTACAAGTCGCCATGCGAGGTGATAGGAATAAAGGAGTAACTGGACATTTCACCTTACACT

>Age_PSG2N (Ateles geoffroyi; black-handed spider monkey) WGS PVHS01050490.1

CGTCACTTTTAAATTTCTGGAACCCACCCACCACTGCCCAAATCATCATTGAAGCCCAGCCACACGTTGTTTCAGAGGGGAAGGATGTTCTTCTACTTGTCCACAATTTGCCCCAGAATCCTGCTGGCTACAGCTGGTACAGAGGGAAAATTACGGACATCGACCATTACATTGCAGCATATTTAACACGCGCTCAAATATGTATATCTGGGCCGGCATACAGTGGACGAGAAACAATATATTCCAATGCATCCCTGCTGATCCAGAACGTCACCCAGAATGACGCAGGATCCTACATCCTACAAGTCGCCATGCGAGGTGATAGGAATAAAGGAGTAACTGGACATTTCACCTTACACC

>Age_PSG3N (Ateles geoffroyi; black-handed spider monkey) WGS PVHS01043663.1

CGTCACTTTTAAACTTCTGGAACCCACCCACCACTGCCCAACTCATGATTGAAGCCCAGCCACACGTTGTTTCAGAGGGGAAGGATGTTCTTCTACTTGTCCACAATTTGCCCCAGAATCCTGCTGGCTACAGCTGGTACAGAGGGAAAATTATGGACATCGACCATTACATTGCAGCATATTTAACACGCGCTGAAATATGTATATCTGGGCCGGCAGACAGTGGACGAGAAACAGTATATCCCAATGCATCCCTGCTGATCCAGAACGTCACCCAGAATGACGCAGGATCCTACATCCTACAAGTCGCCATGCTAGGTGATTGGATTAAGAGAGTAACTGGACATTTCACCTTACACC

>Age_PSGLN (Ateles geoffroyi; black-handed spider monkey) WGS PVHS01040236.1

CCTCACTTGTAAGCTTCTGGAACGCGCCCACCACTGCCCAGGACCCTGAAGCTCTCACTGTGGAATTCATGCCACCCAGAGCCACAGAGGGGAAGAATGTTTTCGTGCTTGTCCACAACTTGCCAAAGTCGTATGTTGGCATCATGTGGCACAGAGGGGCCACCGTGGAGCGTAGTCATTTCATCATAGCATACAGAACATTTGACAGACAATATCTAATAGGGGCTTTATACAGCGGCCAAGAGATATTGTACAGCAATGGAAGCCTGCTGCTCCAGAACGTCACCCGGAATGACACAGGACTGTACAACCTGCACCTCGTAAACTGGAATTTGGTTGATAACGAAGTAACTGGCGAGTTACGCATGTAGG

>Ana_PSG1N (Aotus nancymaae; Ma's night monkey) WGS JYKP02024301.1

CATCACTTTTAAACTTCTGGAACCCATCCACCACTGCCCAAGTCATGATCGAAGCCCAGCCACATGTTGTTTCAGAGGGGAAGGATGTTCTTCTACTTGTCCACAATTTGCCCCAGAATCTTACTGGCTACAGCTGGTACAGAGGGAAAATCAAGGACATCAACCATTACCTTACAGCATATTTAATAGAAACTCGTGAAATTATATTGGGGCCTGCATACACTGGACGAGAAACAATATATTCCAATGCATCCCTGCTGATTGAGAAGGTCACCCTGAATGACACAGGATCCTACACACTGCAAGTCACCACGCATGGTGATAGGAATAAAGGAGCAATTGGACATTTCACCGTATACC

>Ana_PSG2N (Aotus nancymaae; Ma's night monkey) XM_012435584.1

GATCACTTTTAAACTTCTGGAACCCACCCACCACTGCGCAAGTCATGATCGAAGCCCAGCCACACGTTGTTTCAGAGGGGAAGGATGTTCTTCTACTTGTCCACAATTTGCCCCAGAATCTTACTGGCTACAGCTGGTACAGAGGGAAAATCAAGGACATCAACCATTACCTTACAGCATATTTAATAGACACTCATGAAATTATATTGGGGCCTGCATACAGTGGACGAGAAACAATATATTCCAATGCATCCCTGCTGATCGAGAACGTCACCCAGAATGACACAGGATCCTACACACTGCAAGTCACCACGCACGGTGATAGGAATAAAAGAGTAACTGGACATTACACCTTACACC

>Ana_PSG3N (Aotus nancymaae; Ma's night monkey) XM_021668424.1

CGTCACTTTTAAACTTCTGGAACCCACCCACCACTGCCCAAGTCATGATCGAAGCCCAGCCACACGTTGTTTCAGAGGGGAAGGATGTTCTTCTACTTGTCCACAATTTGCCCCAGAATCTTACTGGCTACAGCTGGTACAGAGGGAAAATCAAGGACATCCACCATTACCTTACCGCATATTTAATAGACACTCACGAAATTATATTTGGGCCTGCATACAGTGGACGAGAAACAATATATTCCAATGCATCCCTGCTGATTGAGAAGGTCACCCTGAATGACGCAGGATCCTACACACTGGAAGTCAACACGCACGGTGATAGGAATAAAGGAGTAACTCGACATTTCACCTTACACC

>Ana_PSG4N (Aotus nancymaae; Ma's night monkey) XM_021668418.1 CATCAGTTTTAAACTTCTGGAACCCACCCACCACTGCCCAAGTCATGATCGAAGCCCAGCCACACGTTGTTTCAGAAGGGAAGGATGTTCTTCTACTTGTCCACAATTTGCCCCCAAATCTTATTGGCTACATCTGGTACAGAGGGAAAATCAAGGACAACGACCATTACCTCACAGCATATTTAATAGACACTCATGAAATTATATTTGGGCCTGCATACAGTGGACGAGAAACAATATATTCCAATGCATCCCTGCTGATTGAGAAGGTCACCCTGAATGACGCAGGATCCTACACACTGCAAGTCACCACGCACGGTGATAGGAATAAAGGAATAACTCGACATTTCACCTTACACC

>Ana_PSG5N (Aotus nancymaae; Ma's night monkey) WGS JYKP02024318.1; XM_021670395.1

CGTCACTTTTAAACTTCTGGAACCCACCCACCACTGCCCAAGTCATGATCGAAGCCCAGCCACACGTTGTTTCAGAGGGGAAGGATGTTCTTCTACTTGTCCACAATTTGCCCCAGAATCTTACTGGCTACAGCTGGTACAGAGGGAAGATCAAGGACATCAGCCATTACCTTACAGCATATTTAATAGAAACTCGCGAAGTTATATTTGGGCCTGCATACAGTGGACGAGAAAAAATATATTCCAATGCATCCCTGCTGATCAAGAACGTCACCCTGAATGATGCAGGATCCTACACACTGCAAGTCAACACACGAGGAGATATTAATAAAGGAGCAACTGGACATTTCACCTTACACA

>Apa_PSG1N (Alouatta palliata; mantled howler monkey) WGS PVKV010033604.1

CATCACTTTTAAACTTCTGGAACCCACCCACTACTGCCCAAGTCATGATTGAAGCCCAGCCACATGTTGTTTCAGAGGGGAAGGATGTTCTTCTACTTGTCCACAATTTGCCCCAGAATCTTACTGGCTACAGATGGTACAGAGGGAAAATTATGGACATCAACCATTACCTTACAGCATATTTAATAGACACTCAAATAAGTATATCTGGGCCTGCATACAGTGGACGAGAAACAATATATTCCAATGCATCCGTGCTGATCCAGAACGTCACCCAGAATGATACAGGATCCTACACCCTACAAGTCAGCACGCGAGGTGATAGGAATAAAGGAATAACTGGACATTTCACCTTACACC

>Apa_PSG2N (Alouatta palliata; mantled howler monkey) WGS PVKV010021532.1

CATCACTTTTAAACTTCTGGAACCCACCCACTACTGCCCAAGTCATGATTGAAGCCCAGCCACACGTTGTTTCAGAGGGGAAGGATGTTCTTCTACTTGTCCACAATTTGCCCCAGAATCCTACTGGCTACAGCTGGTACAGAGGGAAAATTATGGACATCAACCATTACCTGGCAGCATATTTAACAGACACTCAAATAAGTATATTTGGGCCTGCATACAGTGGACGAGAAACAATATATCCCAATGCATCCCTGCTGATCCAGAACGTCACCCAGAATGATACAGGATCCTACACCCTACAAGTCGCCATGCCAGGTGATAGGAATAAAGGAGTAACTAGACATTTCACCTTACACC

>Apa_PSG3N (Alouatta palliata; mantled howler monkey) WGS PVKV010019933.1

CATCACTTTTAAACTTCTGGAACCCACCCACTACTGCCCAAGTCATGATTGAAGCCCAGCCACACGTTGTTTCAGAGGGGAAGGATGTTCTTCTACTTGTCCACAATTTGCCCCAGAATCCTACTGGCTACAGCTGGTACAGAGGGAAAATTATGGACATCAACCATTACCTGGCAGCATATTTAATAGACACGCAAATAAGTATATCTGGGCCTGCACACAGTGGACGAGAAACAATATATCCCAATGCATCCCTGCTGATCCAGAACGTCACCCAGAATGACGCAGGATCCTACACCCTACAAGTCAGCACGCAAGGTGACAGGAATAAAGGAGTAACTGGACATTTCACCTTACACC

>Apa_PSG4N (Alouatta palliata; mantled howler monkey) WGS PVKV010037424.1

CATCACTTTTAAACTTCTGGAACCCACCCACCACGGCCCAAGTCATGATTGAAGCCCAGCCACACATTGTTTCAGAGGGGAAGGATGTTCTTCTACTTGTCCACAATTTGCCCCAGAATCTTACTGGCTACAGATGGTACAGAGGGAAAATTATAGACATCAACCATTACCTGGCAGCATATTTAATAGACACTCAAATAAGTATATCTGGGCCTGCACACAGTGGACGAGAAACAATATATCCCAATGCATCCCTGCTGATCCAGAACGTCACCCAGAATGATACAGGATCCTACACCCTACAAGTCAGCACGCAAGGTGATAGGAATAAAGGAGTAACTGGACATTTCACCTTACACT

>Apa_PSG5N (Alouatta palliata; mantled howler monkey) WGS PVKV010030243.1

CATCACTTTTAAACTTCTGGAACCCACCCACTACTGCCCAAGTCATGATTGAAGCCCAGCCACACGTTGTTTCAGAGGGGAAGGATGTTCTTCTACTTGTCCACAATTTGCCCCAGAATCCTACTGGCTACAGCTGGTACAGAGGGAAAATTATGGACATCAACCATTACCTGGCAGCATATTTAACAGACACTCAAATAAGAATATCTGGGCCTGCACACAGTGGACGAGAAATAATATATTCCAATGCATCCCTGCTGATCCAGAACGTCACCCAGAATGACGCAGGATCCTACACCCTACAAGTCGCCATGCCAGGTGATAGGAATAAAGGAGTAACTGGACATTTCACCTTACACC

>Apa_PSG6N (Alouatta palliata; mantled howler monkey) WGS PVKV010079325.1

CATCACTTTTAAACTTCTGGAACCCACCCACCATGGCCCAAGTCATGATTGAAGCCCAGCCACACGTTGTTTCAGAGGGGAAGGATGTTCTTCTACTTGTCCACAATCTGCCCAAGAATCCTACTGCCTACGGCTGGTTCAGAGGGAACATTACGGACATCGACCATTACATTGCAGCATATTTAACAGGCACTCAAAAAAGTATATCTGGGCCTGCATACAGTGGACGAGAAAAAATATATCACAATGCATCCCTGCTGATCCGGAATGTCACCCAGAAGGACGCAGGATCCTACATCCTACACGTCGCCGTGCGAGGTGATTGGATTAAACGAGTAATTGGACATTTCACCTTACACC

>Apa_PSG7N (Alouatta palliata; mantled howler monkey) WGS PVKV010103102.1

CATCACTTTTAAACTTCTGGAACCCACCCACCATGGCCAAACTCATGATTGAAGCCCAGCCACACGTTGTTTCCGAGGGGAAGGATGTTCTTCTACGTGTCCACAATCTGCCCAAGAATCCTACTGCCTACGGTTGGTTCAGAGGGAACATTACGGACATCGACCATTACATTGCAGCATATTTAACACACAGTGAAATAGGTTTAACTGGGCCTGCATACACTGGACGAGAAGAAATATATCACAATGCATCCCTGCTGATCCGGAAAGTCACCCAGAAAGACGCAGGATCCTACATCCTACACATCGCCGTGCGAGGTGATTGGATTAAAAGAGTAATTGGACATTTCACCTTACACC

>Apa_PSGLN (Alouatta palliata; mantled howler monkey) WGS PVKV010028955.1

CCTCACTTGTAAGCTTCTGGAATGCGCCCACCACTGCCCAGGACTCTGAAGCTGTCACTGTGGAATTCGTGCCACCCAGAGCCACGGAGGGGAAGAATGTTCTCATGCTTGTCCACAACTTGCCAAAGTCGTATGTTGGCGTCATGTGGCACAGAGGGGCCACCGTGGGGCGTAGTCATTTCATCATAGCATACAGAACATTTGATAGACAATATCTAATAGGGGCTTTATACAGCGGCCGAGAGATATTGTACAGCAACGGAAGCCTGCTGCTCCAGAACGTCACCCGGAATGACAGAGGACTGTACAACCTGCACCTCGTAAACTGGAATTTGGTTGATAACGAAGTAACTGGCGAGTTACACATGTAGG

>Cal_PSG1N (Cebus albifrons; white-fronted capuchin) WGS PVKJ010062608.1

CATCACTTTTAAACTTCTGGAACCCACCCACCACTGCCCAAGTCATGACTGAAGCCCAGCTACGCATTTTTTCAGAGGGGAAGGATGTTCTTCTACTTGTCCACAATTTACCCCAGAATCTTACTGGCTACACCTGGTACAAAGGGAAAGTGATGGACATCCACCATTACCTTACAGCATATTTAATAGACACTCAAATAACTATAGTTGGGCATGCATACAGTGGACGAGAAACAATATTTTCCAATGCATCCCTGCTGATCGAGAACGTCACCCAGAATGAAGCAGGACCCTACATCCTACAAGTCACCAAGCAAGGTGCTAGGAATGAAGGAGAAACCGGACATTTCACCTTAAAAC

>Cal_PSGLN (Cebus albifrons; white-fronted capuchin) WGS PVKJ010031959.1 CCTCACTTGTAAGCTTCTGGAACCCTCCCACCACTGCCCAGGACCTTGAAGTTCTCACTATGGAATTCGTGCCACCCAGAGCCACAGAGGGGAAGAATGTTCTCATAATTGTCCACAACTTGCCAAAGTCATATATTGGCGTCATGTGGCACAGAGGGGCCACGGTGGAGCGTAGTCATTTCATTATGGCATATAGAACGATTGATAGACTATATATAACAGGGCCTTTATACAGCGGCCGAGAGATACTGCATAGCAACGGAAGCCTGCTGCTCCAGAACGGCACCCGGAATGACAGAGGACTATACAACCTGCAGCTCATGAAATGGGATTTGCTTGTTAACAGAGTAACTGGCGAGTTACGCATGTAGC

>Can_PSG1N (Colobus angolensis palliatus; black and white colobus monkey) XM_011962523.1 CATCACTTTTAATCTTCTGGAACCTGCCCACCACTGCTCAAGTCACAATTGAAGCCCAGCCAACCAAAGTTCCTGAGGGGAAGGATGTTCTGCTACTTGTTCACAATTTGCCCCAGAATGTTACTGGCTACATCTGGTACAAAGGGCAAATAATGGACCTCTACCATTACATTACAGCATATACAATAGACACTGAAATGATTATATCTGGGCCTGCATACAGTGGACGAGAAACAATATATTCCAATGCATCCCTGTTGATCCAGAATGTCACTCAGAATGACACAGGATCCTACACCATTCAAATCACACAGCGAGGTGATGGGACTAAAGGAGTAACTGGACATTTCACCTTATACT

>Can_PSG2N (Colobus angolensis palliatus; black and white colobus monkey) XM_011960532.1 CATCACTTTTAATCTTCTGGAACCCGCCCATCGCTGCCCAAGTCATGATTGAAGCACAGCCAAACAAAGTTTCTGAGGGGAAGGATGTTCTTCTACTTGTCCACAATTTGCCCCAGAATCTTGCTGCCTACATCTGGTACAAAGGGCAAATAATGGACCTCCACCATTACATTACAGCATATGTAATAGACCCTGAAACAATTATATTTGGGCCTGCATACAGTGGACGAGAAACAGTATATTCCAATGCATCCCTGCTGATTCAGAATGTCACCCAGAAGGACACAGGATCCTACACCATACAAATCATAAAGCAAGGTGATAGGACTAAAGGAGTAACTGGACATTTCACCTTATACC

>Can_PSG3N (Colobus angolensis palliatus; black and white colobus monkey) XM_011960537.1 CATCACTTTTAATCTTCTGGAACCCGCCCACCACTGCCCAAGTCACGATTGAAGCCCAGCCAACCAAAGTTTCCGAGGGGAAGGATGTTCTGCTACTTGTCCACAACTTGCCCCAGAATCCTATTGGCTACATCTGGTACAAAGGGCAAATAATGGACATCGACCATTACATTACATCATATGTAATAGACGCTGAAACAATTATACCTGGGCCTGCATACAGTGGACGAGAAACAGTATATTCCAATGCATCCCTGCTGATCCAGAATGTCACCCGGAAGGACACAGGATCCTACACCATACAAATCATAAAGCTAGGTGATAAGACTAAAGGAGTAACTGGACATTTCACCTTATACC

>Can_PSG4N (Colobus angolensis palliatus; black and white colobus monkey) XM_011963132.1 TATTACTTTTCATCTTCTGGAACCCGCCCACCACTGCCCAAGTCACGATTGAAGCCCAGCCAACCAAAGTTTCCGAGGGGAAGGATGTTCTTCTACTTGTCCACAATTTGCCCCAGAATCTTACTGGCTACATCTGGTACAAAGGGCAAATAATAGACCTCCACCAATTCATTACAGCATATACAATAGACACTGAAACAATTATATCTGGGCCTGCATACAGTGGACGAGAAACAGTATATTCCAATGCATCCCTGCTGATCCAGAATGTCACCCGGAAGGACACAGGATCCTACACCATACAAATTATAAAACGAGGTGATAAGATTAAAGGAGTAACTGGACATTTCACCTTATACC

>Can_PSG5N (Colobus angolensis palliatus; black and white colobus monkey) XM_011936589.1 CATCACTTTTAATCTTCTGGAAACCGCCCACCGCTGCCCAAGTCATGATTGAAGCTCAGCCAACCAAAGTTTCCAAGGGGAAGGATGTTCTTCTACTTGTCCACAACTTGCCCCAGAATGTTGCTGCCTACATCTGGTACAAAGGGCAAATAATGGACGTCCACCATTACATTACGGGATATGTAATAGACCCTGAAACAATTATATTTGGGCCTGCATACACTGGACGAGAAAGACTATATTCCAACGCATCCCTGCTGATTCAGAAAGCAACCCAGAAGGACACAGGATCCTACACCATAAAAATCACAAAGCGAGGTGATAAGACTAAAGGAGTAACTGGACATTTCACCTTATACT

>Can_PSG6N (Colobus angolensis palliatus; black and white colobus monkey) XM_011927947.1 CATCACTTTTAATCTTCTGGAACCCGCCCACCGCTGCCCAAGTCATGATTGAAGCTCAGCCAACCAAAGTTTCTGAGGCGAAGAATGTTCTTCTACTTGTCCACAATTTGCCCCAGAATGTTGCTGCCTACCTCTGGTACAAAGGGCAAATAATGGACGTCCACCATTACATTACGGGATATGTAATGGAGACTGAAGGAATTATGTTTGGGCCTGCATACAGTGGACGAGAAACAGTATATTCCAATGGATCCCTGCTGATTCAGAATGTCACCTGGAAGGACACAGGATCCTACACCATACAAATCATAAAGCGAGGTGATAAGCCTAAAGAAGTAATTGGACATTTCACCTTATACT

>Can_PSG7N (Colobus angolensis palliatus; black and white colobus monkey) WGS JYKR01145156.1 CATCACTTTTAATCTTCTGGAAACCGCCCACCACTGCCCAAGTCATGATTGAAGCTCAACCAATCAAAGTTTCTGAGGGGAAGGATGTTCTGCTACTTGTCCACAATTTGCCCCAGAATGCTGCTGCCTACACCTGGTACAAAGGGCAAATAATGGACTTCTACCAATTCATTACAGCATATACAAGATACCCTGATAGAATTCTATTTGGGCCTGCATACAGTGGACGAGAAACACTATATTCCAATGGATCCCTGGGGATCCAGAATGTCACCAAGCAGGACACAGGATCCTACACCGTAAAAGTCATGAAGCGAATTGATGATACTAAAGGAGTAACTGGACATTTCACCTTATACT

>Can_PSG8N (Colobus angolensis palliatus; black and white colobus monkey) XR_001003844.1

CATCACTTTTCATCTTCTGGAACCCGCCTACCATGGCCCAAGTCATGATTGAAACTCAGCCAACCAATGTTTCTGAGGGGAAGGATGTTCTTCTACTTGTCCACAATTTGCCCCAGAATCCTACTGGCTACATCTGGTACAAAGGGCAAATAACGGATATCCACAATTACATTACATCATATGTAATAGACACTGATACAATTATATCTGGGCCTGCATACAGTGGACGAGAAACAGTATATTCCAATGCATCCCTGCTGATCCAGAATGACACCCAGAAGGACACAGAATCCTACACCATACAAATCAAAAAGCGAGGTGATAGCACTAAAGGAGTAACTGGACATTTCACCTTATACC

>Can_PSG9N (Colobus angolensis palliatus; black and white colobus monkey) XR_001003843.1

CATCACTTTTAATCTTCTGGAACCCGCCCACCACTGCCCAAGTCATGATTGAAGCTCAGCCAACCAATGTTTCTGAGGGGAACGATGTTCTTCTACTTGTACACAATTTGCCCCAGAATCCTGCTGCCTATATCTGGTACAAAGGGCAAATAATGGACGTCCACCATTACATTACAGCATATGTAATAGAAACTGAAAGAATTGTATTTGGGCCTGCATACAGTGGACGAGAAACAGTATATTCCAATGCATCCCTGCTGATCCAGAGTCTCAACCAGAAGGATGCAGGATCCTACACCATAGAAATCATAAAGCGAGGTGATGGGAATGAAGGAGTAACTGGAAATTTCACCTTATACC

>Can_PSG10N (Colobus angolensis palliatus; black and white colobus monkey) XR_001003846.1

CATCACTTTTAATCTTCTGGAACCCGCCCACCACTGCCCAAGTCATGATTGAAGCTCAACCAACCAAAGTTTCTGAGGGGAAGGATGTTCTTCTACTTGTCCACAATTTGCCCCAGAATCCTGCTGCCTACGTCTGGTACAAAGGGCAAATAATGGACTTCTACCAATTTATTACAGCATATTCAAGAGACCCTGATAGAATTCTATTTGGGCCTGCATACAGTGGACGAGAAACACTATATTCCAATGGATCCTTGCGGATCCAGAATGTCACCAAGCAGGACACAGGATCCTACACCGTAAAAGTCATGAAGCAAGTTGATGATACTAAAGGAGTGACTGGCTATCTTCTCTCTGTTA

>Can_PSG11N (Colobus angolensis palliatus; black and white colobus monkey) XR_001003845.1

CATCACTTTTAATCTTCTGGAATCCGCCCACCACTGCGCAAGTCACGATTGAAGCCCAGCCAACCAAAGTTCCTGAGGGGAAGGATGTCCTGCTACTTGTTCACAATTTGCCCCAGAATATTGCTGCCTATATCTGGTACAAAGGGCAAATAATGGACGTCCGCCATTACATTACAGCATATATAATAGACACTGAAATGATTATATTGGGGCCTGCATACAGTGGACGAGAAACAATATATTCCAATGCATCCCTGCTGATCCAGAATGTCACCCAGAATGACACAGGATCCTACACCATTCAAATCACACAGCGAGGTCATGGAACTAAAGGAGTAACTGGACATTTCACCTTATACT

>Can_PSG12N (Colobus angolensis palliatus; black and white colobus monkey) XR_001001172.1

CATCACTTTTAATCTTCTGGAACCCGCCCACCACTGCTCAAGTCACGATTGAAGCCCAGCCAACCAAAGTTTCTGAGGGGAATGATGTTCTTCTACTTGTCCACAATTTGCCCCAGAATCTTACTGGCTACATCTGGTACAAAGGGCAAATAATGGACTATTACCATTACATTACATCATATGTAATAGACCCTGAAACAATTATATTTGGGCCTGCATACAGTGGACGAGAAACAGTATATTCCAATGCATCCCTGCTGATCCAGAATGTCACCCGGAAGGACACAGGATCCTACACCATACAAATCATAAAGCGAGGTGATAGGACTGAAGGAGTAACTGGACATTTCACCTTATACC

>Can_PSG13N (Colobus angolensis palliatus; black and white colobus monkey) XR_001003848.1

CATCACTTTTAATCTTCTGGAACCCACACACCTCTGCGCAAGTCACGATTGAAGCCCAGCCAAAAAAAGTTTCTGAGGGGAAGGATGTTCTGCTACTTGTCCACAATTTGCCCCAGAACCTTGCTGGATACATCTGGTACAAAGGGCAAATAATGGACCTCTACCATTATATTACAGCATATACAATAGACACTGAAATGATTATATTTGGGCCTGCATACAGTGGACGAGAAACAGTATATTCCAATGCATCCCTGCTGATCCAGAATGTCACCCAAAATGACACAGGATCCTACACCATACAAATCATAAAGCGAGGTGATAAGACTAATGGAGTAACCGGACATTTCACCTTACACC

>Can_PSG14N (Colobus angolensis palliatus; black and white colobus monkey) XR_001003849.1

CATCACTTTTAATCTTCTGGAACCCGCCCACCACTGCCCAAGTCACGATTGAAGCTCAGCCAACCAAAGTCTCTGAGGGGAAGGATGTTCTTCTACTTGTCCAAAATTTGCCCCAGAATGTTGTTGGCTACATCTGGTACAAAGGGCAAATAATAGACCTCCACCATTACATTACCGCATATACAATAGACACTGAAACAATTATATTTGGGCCTGCATACAGTGGACGAGAAACAGTATATTCCAATGCATCCCTGCTGATCCAGAGTGTCACCAAGCAGGACATAGGATCCTACACCATAAAAATCATAAAGCGAGGTGATGGGACTGAAGGAGTAACTGGACATTTCACCTTATACC

>Can_PSG15N (Colobus angolensis palliatus; black and white colobus monkey) WGS JYKR01156837.1

CATCACTTTTAATCTTCTGGAACCCGCCCACCACTGCTCAAGTCACGATTGAAGCCCAGCCAACCAAAGTTTCTGAGGGGAAGGATGTTCTGCTACTTGTCCATAATTTGCCCCAGAATGTTACTGGCTACATCTGGTACAAAGGGCAAATAATGGACCTCTACCATTACATTACAGCATATACAATAGACACTGAAATGATTATATTTGGGCCTGCATACAGTGGACGAGAAACTATATATTCCAATGCATCCCTGCTGATCCAGAATGTCACGCAGAATGACACAGGATCCTACACCATTGAAATCACACAGCGAGGTGATGGGACTAAAGGAGTAACTGGACATTTCACCTTATACC

>Can_PSG16N (Colobus angolensis palliatus; black and white colobus monkey) WGS JYKR01037186.1

CATCACTTTTAATCTTCTGGAACCCGCCCACCACTGCTCAAGTCACGATTGAAGCCCAGCCAACCAAAGTTTCTGAGGGGAAGGATGTTCTGCTACTTGTCCAGAATTTGCCCCAGAATCTTACTGGCTACAACTGGTACAAAGGGCAAATAATGGACCTCTACCATTACGTTACAGCATATACAATAGACACTGAAATAACTATATTTGGGCCTGCATACAGTGGACGAGAAACAGTATATTCCAATGGATCCCTGCTGATCCAGAATGTCACCCAGAAGGACACAGGATCCTACACCATACAAATCACAAAGCGAGGTGATAGGACTGAAGGAGTAACTGGACATTACACCTTATACc

>Can_PSG17N (Colobus angolensis palliatus; black and white colobus monkey) WGS JYKR01156856.1

CATCACTTTTAATCTTCTGGAACCCGCCCACCACTGCCCAAGTCATGATTGAAGCCCAGCCAACCAAAGTTTCCAAGGGGAAGGATGTTCTTCTACTTGTTCACAATTTGCCCCAGAATGTTGCTGCCTACATCTGGTACAAAGGGCAAATAATGGACCTCCACCATTACATTATGGGATATGTAATAGAGGCTGAAGCAATTATATTTGGGCCTGCATACAGTGGACGAGAAACAGTATATTCCAATGCATCCCTGCTGATTCAGAATGTCACCTGGAAGGACACAGGATCCTACACCATACAAATCATAAAGCGAGGTGATAAGACTAAAGGAGTAACTGGACATTTCACGTTACACT

>Can_PSG18N (Colobus angolensis palliatus; black and white colobus monkey) WGS JYKR01156871.1

CATCACTTTTAATCTTCTGGAACCCACCCATCCCTGTGCAAGTCACGATTGAAGCCCAGCCAACAAAAGTTTCTGAGGGGAAGGATGTTCTGCTACTTGTCCACAATTTGCCCCAGAATCTTATTGGATACATCTGGTACAAAGGGCAAATAATGGACGTCCACCATTACATTATGGGATATGTAATAGAGGCTGAAGCAATTATATTTGGGCCTGCATACAGTGGACGAGAAACAGTATATTCCAATGCATCCCTGCTGATCCAGAATGTCACCCGGAAGGACACAGGATCCTACACCATTCAAGTCATAAAGCGAGGTGATAAGACTAAAGGAGTAACTGGACATTTCACCTTATACc

>Can_PSGP2N (Colobus angolensis palliatus; black and white colobus monkey) WGS JYKR01145176.1

CATCACTTTTCATCTTCTGGAACCTGCCTACCATGGCCCAAGTCATGATTGAAGCTCAGCCAACTGAAGTTTCTGAGGGGAAGGATGTTCTTCTACTTGTCCACAATTTGCCACAGAATCCTACTGGCTACATCTGGTACAAAGGGCAAATAACGGATATCCACAATTACATTACATCATATGTAATAGACACTGATACAATTATATCTGGGCCTGCATACAGTGGACGAGAAACAGTATATTCCAATGCATCCCTGCTCATCCAGAATGTCACCCATAAGGACACAGGATCCTACACCATACAAATCATAAAGTGAGGTGATAGCACTAAAGGAGTAACTGGACATTTCACCTTATACC

>Can_PSGP3N (Colobus angolensis palliatus; black and white colobus monkey) WGS JYKR01145191.1

CATCACTTTCAATCTTCTGGAACCCGCCCACCACTGCCCAAGTCATGATTGAAGCTCAGCCAACCAAAGTTTCTGAAGGGAAGGATATTCTTCTACTTGTCCACAATTTGCCCCAGAATCCTGCTGCCTACTTCTGGTACAAAGGGCAAATAATGGACCTCCACCATTACATTCAGCATATACAATAGACACTGAAAGAGTTATATTTGGGCCTCCATACAGTGGACGAGAAACAGTATATTCCAACGCTTCCCTGCTGATCCAGAGTGTCACCTAGAAGGACGCAGGATCCTACACCGTAGAAATCATAAAGCAAGGTGATAGGACTGAAGGAGTAACTGGACATTTCACCTTATACc

>Can_PSGP4N (Colobus angolensis palliatus; black and white colobus monkey) WGS XR_001003847.1

CATCACTTTCAGTCTTCTGGAACCCACCCAACGCTGCTCAAGTCACGATTGAAGCCCAGCCAATGAAAGTTTCTGAGGGGAAGGATGTTCTTCTACTTGTCCACAATTTGCCCCAGAATCTTACTGGCTACATCTGGTACAAAGGGCAAATAACGGACCTCCACCATTACATTAAATCATATGTAATAGACTCTGAAACAATTATATTTGGGCCTGCATACAGTGGATGAGAAACAGTATATTCCAATGCATCCCTGCTGATCCAGAATGTTACCCGGAAGGACACAGGATCCTACACCATACAAATCATAAAGCGAGGTGATAGGACTGAAGGAGTAACTGGACATTACACTTTATACC

>Cat_PSG1N (Cercocebus atys; sooty mangabey) XM_012030792.1

CATCACTTTTAATCTTCTGGAACCCACCTACCACTGCCCAAGTCACGATTGAAGCTCAGCCAACCAAAGTTTCTGAGGGGAAGGATGTTCTGCTACTTGTTCACAATTTGCCCCAGAATCTTACTGGCTACAGCTGGTACAAAGGGCAAATAATGGACCTCCAGCATTACATTACAGCATATACAATAGACACTGAAATGATTGTATTTGGGCCTGCATACAGTGGACGAGAAAGAGTATATTCCAATGCATCCCTGCTGATCCAGAATGTCACCAAGAATGACACAGGATCCTACACCATTCAAATCACAAAGCGAGGTGATGAGACTAAAGGAGTAACTGGACATTTCACCTTATACC

>Cat_PSG2N (Cercocebus atys; sooty mangabey) XR_001010804.1

CATCACTTTTAATCTTCTGGAACCCGCTCACCACTGCCCAAGTCACGATTGAAGCACAGCCAACCAAAGTTTCTGAGGGAAAGGATGTTCTGCTACTTGTCTACAATTTGCCCCAGAATCTTACTGGCTACAGGTGGTACAAAGGGCAAATAATGGACCTCCAGCATTACATTGCAGCATATACAACAGACACTGAAATGATTATATTTGGGCCTGCATACAGTGGACGAGAAACAGTATATTCCAATGCATCCCTGTTGATCCAGAATGTCACCAAGAATGACACAGGATCCTACACCATTCAAATCACAAAGCGAGGTGATGGGACTAAAGGAGTAACTGGACATTTCACCTTATACC

>Cat_PSG3N (Cercocebus atys; sooty mangabey) XM_012086966.1

CATCACTTTTAATCTTCTGGAACACGCCCACCACTGCCCAAGTCACGATTGAAGCCCAGCCAACCAAAGTGTCTGAGGGGAAGGATGTTCTGCTACTTGTCCACAATTTGCCCCAGAATCTTGCTGCCTACATCTGGTACAAAGGGCAAATAATGGACCTCCACCATTACATTACATCATATGTAATAGACACTGAAATAATTGTATTTGGGCCTGCATACAGTGGACGAGAAACAGTATATTCCAATGCATCCCTGCTGATCCAGAATGTCACCCAGAAGGACACAGGATCCTACACCATACAAATCATACAGCGAGGTGATACCACTAAAGGAGTAACTGGACATTTCACCTTATACC

>Cat_PSG4N (Cercocebus atys; sooty mangabey) XM_012030798.1

CATCACTTTTAATCTTCTGGGACCCGCCCACCACTGCCCAAGTCACGATTGAAGCTCAGCCAACCAAAGTTTCTGAGGGGAAGGATGTTCTTCTACTTGTCCACAATTTGCCCACGAAAGTTGCTGGCTACATCTGGTACAAAGGGCAAATAATGGACCTCCAGCATTACATTACAGCATATACAATAGACACTGAAAAGATTATATTTGGGCCTGCATACAGTGGACGAGAAACAGTATATTCTAATGCATCCCTGCTGATCCAGAATGTCACCAAGAATGACACAGGATCCTACAGCATTCAAATCACAAACCCATGTGATGAGACTAAAGGAATAACTGGACATTTCACCTTATACG

>Cat_PSG5N (Cercocebus atys; sooty mangabey) XM_012049289.1

CATCACTTTTAATCTTCTGGAACCCGCCCACCACTGCCCAAGTCACGATTGAATCTCAGCCAACCAAAGTTTCTGAGGGGAAGGATGTTCTTCTACTTGTCCACAATTTGCCCACGAACGTTGTTGGCTACATCTGGTACAAAGGGCAAATAATGGACCTCCAGCATTACATTACAGCATATACAACAGACACTGAAATGATTATATTTGGGCCTGCATACAGTGGACGAGAAACAATATATTCCAATGCATCCCTCCTGATCCAGAGTGTTACCAAGAATGACACAGGATCCTACACCATACAAATCATAAAGCGAGGACATAGGACTGAAGGAGTAACTGGACATTACACCTTATACC

>Cat_PSG6N (Cercocebus atys; sooty mangabey) XM_012086967.1

CATCACTTTTAATCTTCTGGAACCCACCCACCACTGCTCAAGTCACAATTGAAGCCCAGCCAGCCAAAGTTTCCGAGGGGAAGGATGTTCTTCTACTTGTCCACAATTTGCCCCAGAATCTTACTGGCTACATCTGGTACAAAGGGCAAAAAACGGACCACCACCTTTACATTACATCATATGTGATAGACACTGAAACAATTATATTTGGGCCTGCATACAGTGGACGAGAAACAGTATATTCCAATGCATCCCTGCTGATCCAGAATGTCACCCGGAAGGACACAGGATCCTACACCATAGAAATCATACAGCGAGGTGATAGGACTGAAGGAGTAACTGGACATTACACCTTATACC

>Cat_PSG7N (Cercocebus atys; sooty mangabey) XM_012086968.1

CATCACTTTTAATCTTCTGGAACCCACCCACCACTGCTCAAGTCAGAATTGAAGCACAGCCAACCAAAGTTTCCGAGGGGAACGATGTTCTTCTACTTGTCCACAATTTGCCCCAGAATCTTACTGGCTACATCTGGTACAAAGGGCAAAAAACGGACCTCCACCTTTACGTTACATCATATGTAAAAGACACTGAAACAGTTATAGCTGGGCCTGCATACAGTGGACGAGAAACAGTATATTCCAATGCATCCCTGCTGATCCAGAATGTCACCCAGAAGGACACAGGATCCTACACCATACAAATCACAAAGCGAGGTGATAGGACTGAAGGAGAAACTGGACATTTCACCTTATATC

>Cat_PSG8N (Cercocebus atys; sooty mangabey) WGS JZLG01051048.1; XM_012039355.1

TATCACTTTTAATCATCTGGAACCCACCTACCACGGGTCAAGTCACGATTGAAGCTCAGCCAACCGAGGTTTCTGAGGGGAAGGATGTTCTTCTACTTGTCCACAATTTGCCCCAGAATCCTACTGGCTACAGCTGGTACAAAGGGCAAATAACAGACATCCACCATTACATTACATCATATGTAATAGACACTGAAATGATTGTATTTGGGCCTGCATACAGTGGACGAGAAACAGTATATTCCAATGCATCCCTGCTGATCCAGAATGTCACCCAGAAGGACACAGGATCCTACACCATACAAATCATACAGCGAGGTGATACCACTAAAGGAGTAACTGGACATTTCACCTTATACC

>Cat_PSG9N (Cercocebus atys; sooty mangabey) XM_012039898.1

AATCACTTTTAATCTTCTGGAACCCACCCACCACTGCTCAAGTCATGATTGAAGCCCAGCCAACCAAAGATTCCGAGGGGAAGGATGTTCTTCTACTTGTCCACAATTTGCCGCAGAATCTTACTGGCTACATCTGGTACAAAGGGCAAAAAACGGACCACCACCATTATATTACATCATATGTGATAGACACTGAAACAATTATATTTGGGCCTGCATACAGTGAACGAGAAACAGTATACTCCAATGCATCCCTGCTGATCCAGAATGTCACCAAGAATGGCACAGGATCCTACACCATACAAATCATAAAGCGAGGTCATAGGACTGAAGGAGTAACTGGACATTACACCTTATCCC

>Cat_PSG10N (Cercocebus atys; sooty mangabey) XM_012055136.1

CATCACTTTTAATCTTCTGGAACTCGCCCACCACTGCCCAAGTCACAATTGAAGCTCAGCCAACCAATATTTCCGAGGGGAATGATGTTCTTCTACTTGTACACAATTTACCCAAGAATCCTGCTGCCTACATCTGGTACAAAGGGCAAATAATGGACCTCCAACATTACATTACAGCATATACAATATACACTGAAAGAATTATATTTGGGCCTGCATACAGTGGACGAGAAACAGTATATTCCAATGCATCCCTGCTGATCCAGAGTGTGAACCAGAAGGACGCAGGATCCTACACCGTAAAAATCATAAAGCGAGGTTACAGGACTGAAGGAGTAACTGGACATTTCACCTTATATG

>Cat_PSG11N (Cercocebus atys; sooty mangabey) XR_001010806.1

CATCACTTTTCATCTTTTGGAACCCACCTATCGCGGCCCAAGTCACGATTGAAGCTCATCCAACCGAAGTTTCTGAGGGGAAGGATGTTCTTCTACTTGTCCACAATTTGCCCCAGAATCCTACTGGCTACATCTGGTACAAAGGGCAAATAACGGATAGCCACAATTACATTACATCATATGTAATAGACACTGAAATGATTATATTTGGGCCTGCATACAGTGGACGAGAAACAGTATATTCCAATGCATCCCTGCTGATCCAGAATGTCACCCGGAAGGACACAGGATCCTACACCATACAAATCATAAAGCGAGGTGATACCACTAAAGGAATAACTGGACATTACACCTTATACC

>Cat_PSG12N (Cercocebus atys; sooty mangabey) XM_012035230.1

CATCACTTTTAATCTTCTGGAACACACCCACCACTGCCCAAGTCACGATTGAAGCACAGCCAACCAAAGTTTCCGAAGGGAAGGATGTTCTTCTACTTGTCCAGAATTTGCCCCAGAATCTTATTGCCTACATCTGGTACAAAGGGCAAAAAACGGACTTCCGCCATTACATTACATCATATGTAATAGATGCTGAAACAATTATAGTTGGGCCTGCATACAGTGGACGAGAAACAGTATATTCCAATGCATCCCTGCTGATCCAGAATGTCACCCAGAAGGACACAGGATCCTACACCATACAAATGATAAAGCAAGGTGATAAGACTAAAGGAGTAATTGGACATTTCACCTTATACC

>Cat_PSG13N (Cercocebus atys; sooty mangabey) XM_012035231.1

CATCACTTTTAATCTTGTGGAACCCGCCCACCACTGCCCAAGTCATGATTGAAGCTCAGCCTACCAAAGTTTCTGAGGGGAAGGATGTTCTTCTACCTGTCCGCAATTTGCCCCAGAAAGTTGCTGCCTACATCTGGTACAAAGGGCAAATAATGGACTTCCACCAATTCATTACAGCATATACAATAGACACTGAAAGAATTATATTTGGGCCTGCATTCAGTGGACGAGAAACACTATATTCCAATGGATCCCTGCTGATCCGGAATGTCACCAAGAATGACACAGGATCTTACACCGTAAAAATCAGGAACCCAGCTGAGGAGACTAAAGGAGTAACTGTACATTTCACCTTATATC

>Cat_PSG14N (Cercocebus atys; sooty mangabey) XM_012030790.1

CATCACTTTTAATCTTCTGGAACCCGCCCACCACTGCCCAAGTCACGATTGAAGCCCAGCCAGCCAAAGTTTCCGAGGGGAAGGATGTTCTTCTACTTGTCCACAATTTGCCCCAGAATCTTACTGGCTACGTCTGGTTCAAAGGGCAAATAACGAGCTTCCACCAATTCATTATAGCATATAAAATAGACAGTAAAAAAATTACAGTTGGGCCTGCATACAGTGGACGAGAAAGAGTATATTCCAATGCATCCCTGCTGATTCAGAATGTCACCCAGAAGGACACAGGATCCTACACCATACAAATTATAAAGCGAGGTGATAAGATTAAAGGGGTAACTGGACATTTCACCTTATACG

>Cat_PSG15N (Cercocebus atys; sooty mangabey) XR_001010805.1

CGTCATTTTTAGTCTTCTGGAACCCGCCCACCACTGCCCAAGTCATGATTGAAGCTCAGCCTACCAAAGTTTCCGAGGGGAAGGATGTTCTTCTACTTGTCCGCAATTTGCCCCAGAAAGTTGCTGCCTATGTCTGGTACAAAGGGCAAATAATGGACTTCCACCAATTCATTACAGCATATACAATAGATCCTGAAAGAATTATATTTGGGTATGCATACAGTGGACGAGAAACACTATATTCCAATGCATCCCTGCTGATCCGGAATGTTACAAAGCAGGACACAGGGTCCTACACCGTAAAAATCATGAATCGAATGGAGGAGACTAAAGGAGTAACTGTACATTTCACCTTATACC

>Cat_PSG16N (Cercocebus atys; sooty mangabey) WGS JZLG01045649.1

CATCACTTTTAATCTTCTGGAACCTGCCCACCACTGCCCAAGTCACGATTGAAGCCCAGCCAGCCAAAGTTTCTGAGGGGAAGGATGTTCTGCTACTTGTCCACAATTTGCCCCAGAATGTTGCTGGCTACAGCTGGTACAAAGGGCAAATAATGGACCTCCACCATTACATTACAGCATATACAACAGACACTGAAATGATTATATTTGGGCCTGCATACAGTGGACGAGAAACAATATATTCCAATGCATCCCTGCTGATCCAGAATGTCACCCAGAAGGACACAGGATCCTACACCATTCAAATCACAAAGCGAGGTGATGGGACTAAAAGAGTAACTGGACATTTCACTTTATACC

>Cat_PSG17N (Cercocebus atys; sooty mangabey) WGS JZLG01051486.1

CATCACTTTTAATCTTCTGGAACCCGTCCACCACTGCCCAAGTCACGATTGAAGCTCAGCCAACCAAAGTTTCTGAGGGGAAGGATGTTCTTCTACTTGTCCACAATTTGCCCACGAACGTTGTTGGCTACATCTGGTACAAAGGGCAAATAATGGACCTCCAGCATTACATTACAGCATATACAACAGACACTGAAATGATTATATTTGGGCCTGCATACAGTGGACGAGAAACAATATATTCCAATGCATCCCTGCTGATCCAGAGTGTCACCAAGAATGACACAGGATCCTACACCATACAAATCATAAAGCGAGGTCATAGGACTGAAGGAGTAACCGGACATTACACCTTATACC

>Cat_PSG18N (Cercocebus atys; sooty mangabey) WGS JZLG01031241.1

CATCACTTTTAATCTTCTGGAACCCACCCACCACTGCTCAAGTCACAATTGAAGCCCAGCCAGCCAAAGTTTCTGAGGGGAAGGATGTTCTTCTACTTGTCCACAATTTGCCCCAGAATCTTGCTGCCTGCATCTGGTACAAAGGGCAAATAATGGACCTCCAACATTACATTACAGCATATGTAATAGATGCTGAAACAATTATATTTGGGCCTGCATACAGTGGACGAGAAACAGTATATTCCAATGCATCCCTGCTGATCCAGAATGTCACCCAGAAGGACACAGGATCCTACACCATACAAATCATACAGCGAGGTGATAAGACTAAAGGAGTAACTGGACATTTCACCTTATATG

>Cat_PSG19N (Cercocebus atys; sooty mangabey) WGS JZLG01040679.1

CATCACTTTTAATCTTCTGGAACCCACCCACCACTGAACAAGTCACGATTGAAGCCCAGCCAACCAAAGTTTCCGAGGGGAAGGATGTTCTTCTACTTGTCCACAATTTGCCCCAGAATCTTACTGGCTACATCTGGTACAAAGGGCAAATAACGGACCACCACCATTACATTACATCATATGTGATAGACACTGAAACAATTATATTTGGGCCTGCATACAGTGAACGAGAAACAGTATATTCCAATGCATCCCTGCTGATCCAGAATGTCACCCAGAATGACACAGGATCCTACACCATACAAATCATAAAGCGAGGTCATAGGACTGAAGGAGTAACTGGACATTACACCTTATACC

>Cat_PSG20N (Cercocebus atys; sooty mangabey) WGS JZLG01073884.1

CATCACTTGTAATCTTCTGGAACCCGCCCACCACTGCCCAAGTCACGATTGAAGCCCAGCCAGCCAAAGTTTCCGAGGGGAAGGATGTTCTGCTACTTGTCCAGAATTTGCCTGAGAATCTTACTGGCTACGTCTGGTTCAAAGGGCAAATAATGGACTTCCACCAATTCATTACAGCGTATACAATAGACACTGAAACAATTATATTTGGGCCTGCATACAGTGGACGAGAAACAGTATATTCCAATGCATCCCTGCTGATCCAGAATGTCACCCAGAATGACACAGGATCCTACACCATAGAAATTATAAAGCGAGGTGATAAGATTAAAGGAGTAACTGGACATTTCACCTTACACC

>Cat_PSG21N (Cercocebus atys; sooty mangabey) WGS JZLG01051050.1

CATCACTTTTAATCTTCTGGAACTCGCCCATCACTGCCCAAGTCACAATTGAAGCTCAGCCAACCAATATTTCCGAGGGGAATGATGTTCTTCTACTTGTACACAATTTGCCCCAGAATCCTGCTGCCTACATCTGGTACAAAGGGCAAATAATGGACCTCCAAGATTACATTACAGCATATACAATAGACACTGAAAGAATTATATTTGGGCCTGCATACAGTGGACGAGAAAGAGTATATTCCAATGCATCCCTGCTGATCCAGAGTGTGAACCAGAAGGACGCAGGATCCTACACCGTAAAAATCATAGAGCGAGGTGACGGGACTGAAGGAGTAACTGGACATTTCACCTTATACG

>Cat_PSG22N (Cercocebus atys; sooty mangabey) WGS JZLG01045632.1

CATCACTTTTAATCTTCTGGAACTCGCCCACCACTGCCCAAGTCACAATTGAAGCTCAGCCAACCAATATTTCCGAGGGGAATGATGTTCTTCTACTTGTACACAATTTACCCAAGAATCCTGCTGCCTACATCTGGTACAAAGGGCAAATAATGGACCTCCAACATTACATTACAGCATATACAATAGACACTGAAAGAATTATACTGGGGCCTGCATACAGTGGACGAGAAAGAGTATATTCCAATGCATCCCTGCTGATCCAGAGTGTGAACCAGAAGGACGCAGGATCCTACACCATAAAAATCATAAAGCGAGGTTACAGGACTGAAGGAGTAACTGGACATTTCACCTTATAtg

>Cat_PSGP1N (Cercocebus atys; sooty mangabey) WGS JZLG01040669.1

CATCACTTTTAATCTTCTGGAACCCGCTCACCACTGCCCAAGTCACGATTGAAGCTCAGCCAACCAAAGTCTCTGAGGGGAAGGATGTTCTTCTACTTGTCCAGAATTTGCCCCAGAATCTTACTGGCTACGTCTGGTACAAAGGGCAAAAAATGGACCTCCACCAATTCATTATAGCGTATACAATAGACACTGAAACAATTATATTTGGGCCTGCATACAGTGGATGAGAAACAGTATATTCGAATGCATCCCTGCTGATTCAGAATGTCACCCGGGAGGACACAGGATCCTACACCATACAAATTATAAAGCGAGGTGATAAGATTAAAAGAATAACTGGACATTTCACCTTATACC

>Cat_PSGP2N (Cercocebus atys; sooty mangabey) WGS JZLG01045642.1

CATCACTTTTAATCTTCTGGAACACACCCACCACTGCCCAAGTCATGATTGAAGCACAGCCAACCAAAGTTTCCGAGGGGAAGGATGTTCTTCTACTTGTCCAGAATTTGCCCCAGAATCTTATTGCCTACATCTGGTACAAAGGGCAAAAAACGGACTTCCGCCATTACATTACATCATATGTAATAGATGCTGAAACAATTATAGTTGGGCCTGCATACAGTGGATGAGAAACAGTATATTCCAATGCATCCCTGCTGATCCAGAATGTCACCCAGAAGGACACAGGATCCTACACCATACAAATGATAAAGCAAGGTGATAAGACTAAAGGAGTAATTGGACATTTCACCTTATACC

>Cca_PSGLN (Cebus capucinus imitator; capucin monkey) WGS LVWQ01087580.1; XM_017502548.1

CCTCACTTGTAAGCTTCTGGAACCCTCCCACCACTGCCCAGGACCTTGAAGTTCTCACTATGGAATTCGTGCCACCCAGAGCCACAGAGGGGAAGAATGTTCTCATGATTGTCCACAACTTGCCAAAGTCATATATTGGCGTCATGTGGCACAGAGGGGCCACGGTGGAGCGTAGTCATTTCATTATAGCATATAGAACGATTGATAGACTATATATAACAGGGCTTTTATACAGCGGCCGAGAGATGCTGCATAGCAATGGAAGCCTGCTGCTCCAGAACGTCACCCGGAATGACACAGGACTATACAACCTGCAGCTCACGAAATGGGATTTGCTTGTTAACAGAGTAACTGGCGAGTTACGCATGTAGC

>Cja_PSG1N (Callithrix jacchus; marmoset) WGS BBXK01059171.1

CATCACTTTTAAACTTCTGGAACCCACCCACCACTGCCCAAGTCATGATTGAAGCCCAGCCACACGTTGTTTCAGAGGGGAAGGATGTTCTTCTGCTTGTCCACAATTTGCCCCAGAATCTTATTGGCTACAGCTGGTACAGAGGGAAAATCATGGACATCACCCACTACATGACAGCATTTTTAATAGACGGTCACATAACTATATTTGGGCCAGCACACACTGGACGAGAAACAATATATCCCAATGCATCCCTGCTGATCGAGAAGGTCACCCAGAATGACGCAGGACCCTACACCCTACAAGTCATCACGCAAGGTGATAGGAATAATGGAGAAACTGGACATTTCACCTTACACC

>Cja_PSG2N (Callithrix jacchus; marmoset) WGS ACFV01182921.1

CATCACTTTTAAACTTCTGGAGCCCACCCACCACTGCCCAAGTCATGATTGAAGCCCAGCCACACGTTGTTTCAGAGGGGAAGGATGTTCTTCTGCTTGTCCACAATTTGCCCCAGAATTCTATTGGCTACAGCTGGTTCAGAGGGAAAATTAAGGACATCAACTATTACATTACAGCTTATTTAATAGAAACTGACATAACTATATTTGGGCCTGCATACACTGGACGAGAAACGATATATCCCAATGCATCCCTGCTGATCGAGACGGTCACCCAAAATGACGCAGGACCCTACACCCTGCAAGTCATCACGCAAGGTGATAGGAATAATGGAGAAACTGAACATTTCACCTTACACC

>Cja_PSG3aN (Callithrix jacchus; marmoset) WGS ACFV01191621.1

CATCACTTTTAAACTTCTGGAACCCACCCACCACTGCCCAAGTCATGATTGAAGCCCAGCCACACGTTGTTTCAGAGGGGAAGGATGTTCTTCTGCTTGTCCACAATTTGCCCCAGAATCTTATTGGCTACAGCTGGTACAGAGGGAAAATCATGGACATCAACCACTACATGACAGCATTTTTAATAGACCGTCACATAACTATATTTGGGCCTGCACACACTGGACGAGAAACAATATATCCCAATGCATCCCTGCTGATCGAGAAGGTCACCCAGAATGACGCAGGACCCTACACCCTACAAGTCATCACACAAGGTGATAGGAATAATGGAGAAACTGGACATTTCACCTTACACC

>Cja_PSG3bN (Callithrix jacchus; marmoset) WG ACFV01188321.1 PSG3a allele 3 bp difference CATCACTTTTAAACTTCTGGAACCCACCCACCACTGCCCAAGTCATGATTGAAGCCCAGCCACACGTTGTTTCAGAGGGGAAGGATGTTCTTCTGCTTGTCCACAATTTGCCCCAGAATCTTATTGGCTACAGCTGGTACAGAGGGAAAATCATGGACATCAACCACTACATGACAGCATTTTTAATAGACCGTCACATAACTATATTTGGGCCTGCACACACTGGACGAGAAACAATATATCCCAATGCATCCCTGCTGATCGAGAAGGTCACCCAGAATGACGCAGGACCCTACACCCTACAAGTCgcCACgCAAGGTGATAGGAATAATGGAGAAACTGGACATTTCACCTTACACC

>Cja_PSG4N (Callithrix jacchus; marmoset) WGS JRUL010020763.1

catcacttttaaacttctggaacccacccaccactgcccaagtcatgattgaagcccagccacacgttgtttcagaggggaaggatgttcttctgcttgtccacaatttgccccagaatcttattggctacagctggtacagagggaaaatcatggacatcaactattacattacagcttatttaatagaaactgacataactatatttgggcctgcacacactggacgagaaacgatatatcccaatgcatccctgctgatcgagaaggtcacccagaatgacgcaggaccctacaccctacaagtcatcacgcaaggtgataggaataatggagaaactggacatttcaccttacacc

>Cne_PSG1N (Cercopithecus neglectus; De Brazza's monkey) WGS PVKI010292136.1

CATCACTTTTAATCTTCTGGAACCTGCCCACCACTGCCCAAGTTGTGATTGAAGCCCAGCCAGCCAAAGTTTCCGAGGGGAAGGATGTTCTGCTACGTGTCCACAATTTGCCCCAGAATCTTACTGGCTACATCTGGTACAAAGGGCAAATAATGGACCTCCAGCATTACATTACAGCATATACAATAGACACTGAAATGATTATATTTGGGTCTGCATACAGTGGACGAGAAACAGTATATTCCAATGCATCCCTGCTGATCCAGAATGTCACCAAGAATGACACAGGATCCTACACCATTCAAATCACAAAGCGAGGTGATGAGACTAAAGGAGTAACTGGACATTTCACCTTATACC

>Cne_PSG2N (Cercopithecus neglectus; De Brazza's monkey) WGS PVKI010049126.1

CATCACTTTTTATCTTCTGGAACCCGCCTACCACTGCCCAAGTCACTATTGAAGCCCAGCCAACCAACGTTTCTGAGGGGAAGGATGTTCTTCTACTTGTCTACAATTTGCCCCAGAATCTTACTGGCTACAGCTGGTACAAAGGGCAAATAATGGACCTCCACCATTACATTGCAGCATATACAATAGACACTGAAATGATTATATTTGGGCCTGCATACAGTGGACGAGAAACACTATATTCCAATGCATCCCTGCTGATCCAGAATGTCACCAAGAATGACACAGGATTCTACACCATTCGAATCACAAAGCGAGGTGATGAGAGTAAAGGAGTAACTGGACATTTCACCTTATACC

>Cne_PSG3N (Cercopithecus neglectus; De Brazza's monkey) WGS PVKI010079003.1

CATCACTTTTCATCTTCTGGAACCCGCCTACCACTGCCCAAGACATGATTGAAGCCCAGCCAACCAAAGTTTCTAAGGGGAAGGATGTTCTTCTACTTGTCCACAATTTGCCCCAGAATCTTACTGGCTACATCTGGTACAAAGGGCAAATAATGGACCTCCACCATTACATTGCAGCATATACAATAGACACTGAAATGATTATATTTGGGCCTGCATACAGTGGACGAGAAACACTATATTCCAATGCATCCCTGCTGATCCAGAATGTCACACAGAATGACACAGGATCCTACACCATTCAAATCAGAAAGCGAGATGATGAGACTAAAGGGGTAACTGGACATTTCACCTTATACC

>Cne_PSG4N (Cercopithecus neglectus; De Brazza's monkey) WGS PVKI010334896.1

CATCACTTTTAATCTTCTGGAACCCACCCGCCACTGCCCAAGTCACGATTGAAGCACAGCCAACCAAACTTTCTGAGGGGAAGGATGTTCTGCTACTTGTCCACAATTTGCCCCAGAATCTTATTGGCTACAGCTGGTACAAAGGGCAAATAATGGACCTCCACCATTACATTACATCATATGTAATAGACACTGAAATGATTGTATTTGGGCCTGCATACAGTGGACGAGAAACAGTATATTCCAATGCATCCCTGCTGATCCAGAATGTCACCCGGAAGGACACAGGATCCTACACCATACAAATCATAAAGCGAGGTGATAAGATTAAAAGAATAACTGGACATTTCACCTTATACC

>Cne_PSG5N (Cercopithecus neglectus; De Brazza's monkey) WGS PVKI010075549.1

CATCACTTTTAATCTTCTGGAACCCGCCCACCACTGCCCAAGTCACGATTGAAGCTCAGCCAACTAAAGTTTCCGAGGGGAAGGATGTTCTGCTACGTGTCCACAATTTGCCCCAGAATGTTGCTGGCTACATCTGGTACAAAGGGCAAATAATGGACCTGCAGCATTACATTACATCATATGTAATAGACACTGAAATGATTGTATTTGGGCCTGCATACAGTGGACGAGAAACAGTATATTCCAATGCATCCCTGCTGATCCAGAATGTCACCCAGAACGACACAGGATCCTACATCATTCAAATCACAAACCGATGTGATGAGACTAAAGGAGTAACTGAACATTTCACCTTATACC

>Cne_PSG6N (Cercopithecus neglectus; De Brazza's monkey) WGS PVKI010059295.1

CATCACTTTTAATCTTCTGGAACCCGCCCACCACTGCGCAAGTCACGATTGAAGCACAGCCAACCAAAGTTTCTGAGGGGAAGGATATTCTTCTACTTGTCCACAATTTGCCCCAGAATGTTGCTGGCTACATCTGGTACAAAGGGCAAATAATGGACCTCCAGTATTACATTACAGCATATGAAATAGACACTGAAATGATTATATTTGGGCCTGCATACAGTGGACGAGAAACAGTATATTCCAATGCATCCCTGCTGATCCAGAGTGTCAACCAGAAGGATGCAGGATCCTACACCATAGAAATCATAAAGCGAGGTGATGGGACTGAAGGAGTAACTGGACATTTCACCTTATACC

>Cne_PSG7N (Cercopithecus neglectus; De Brazza's monkey) WGS PVKI010085723.1

CATCACTTTTAATCTTCTGGAACCCACCCACTACTGCCCAAGTCACGATTGAAGCCCAGCCAGCCAAAGTTTCCGAGGGGAAGGATGTTCTGCTACGTGTCCACAATTTGCCCCAGAATCTTGCTGCCTACATCTGGTACAAAGGGCAAATTATGGACCTCCACCATTACATTACATCGTATGTAATAGACACTGAAATAATTGTATTTGGGCCTGCATACAGTGGACGAGAAACAGTATATTCCAATGCATCCCTGCTGATCCAGAATGTCACCCAGAAGGACACAGGATCCTACACCATACAAATCATAATGCGAGGAAATAGGACTAAAGGAGTAACTGGACATTTCACCTTATACC

>Cne_PSG8N (Cercopithecus neglectus; De Brazza's monkey) WGS PVKI010055597.1

CATCACTTTTAATCTCCTGGAACCCGCCCACCACTGCCCAAGTCACGATTGAAGCTCAGCCAACCAACGTTTCTGAGGGGAAGGATATTCTTCTACTTGTCCACAATTTGCCCCAGAATGTTGCTGGCTACATCTGGTACAAAGGGCAAATAATGGACCTCCAGCATTACATTACAGCATATACAATAGACACTGAAACAATTATATTTGGGCCTGCATACAGTGGACGAGAAACAGTATATTCCAATGCATCCCTGCTGATCCAGAGTGTCAACCAGAAGGATGTAGGATCCTACACCGTAGAAATCATCATAAAGCGAGGTGATGGGACTGAAGGAGTAACTGGACATTTCACCTTATACC

>Cne_PSG9N (Cercopithecus neglectus; De Brazza's monkey) WGS PVKI010106788.1

CATCACTTTTAATCTTCTGGAACCCGCCCACCACTGCCCAAGTCACAATTGAAACCCAGCCAACCAAAGTTTCTGAGGGGAAGGATGTTCTTCTACTTGTCCACAATTTGCCCCAGAATCTTACTGGCTACATCTGGTACAAAGGGCAAATAATGGACCACCACCATTATATTACATCATATGTGATAGACACTGAAACAATTATATTTGGGCCTGCATACAGTGGACGAGAAACAGTATATTCCAATGCATCCCTGCTGATCCAGAATGTCACCTGGAACGACACAGGATCCTACACCATACAAATCATAAAGCGAGGTGATAGGACTGAAGGAATAACTGGACATTACACCTTATACT

>Cne_PSG10N (Cercopithecus neglectus; De Brazza's monkey) WGS PVKI010481731.1

CATCACTTTTAATCTTCTGGAACCCACCCACCACTGCTCAAGTCACGATTGAAGCCCAGCCAACCAAAGTTTCTGAGGGGAAAGATGTTCTTCTACTTGTCCACAATTTGCCCCAGAATCTTACTGGCTACATCTGGTACAAAGGGCAAAAAACGGACCTCCACCTTTACGTTACATCATATGTAAAAGACACTGAAACAATTATAGCTGGGCCTGCATACAGTGGACGAGAAACAGTATATTCCAATGCATCCCTGCTGATCCAGAATGTCACCCAGAAGGACACAGGACCCTACACCATACAAATCACAAAGCGAGGTGATAGGACTGAAGGAGAAACTGCACATTTCACCTTATATC

>Cne_PSG12N (Cercopithecus neglectus; De Brazza's monkey) WGS PVKI010138569.1

CATCACTTTTCATCTTCTGGAACCCACCTACCACGGCTCAAGTCACAATTGAAGCTCAGCCAACTGAAGTTTCTGAGGGGAAGGATGTTCTTCTACTTGTCCACAATTTGCCCCAGAATCCTACTGGCTACATCTGGTACAAAGGGCAAATAACGGAGAGCCACAATTACATTACATCATATGTAATAGACACTGAAATGATTTTATTTGGGCCTGCATACAGTGGACGAGAAACAGTATATTCCAATGCATCCCTGCTGATCCAGAATGTCACCCAGGACACAGGATCCTACACCATACAAATAATACAGCGAGGTGATAGCACTAAAAGAGTAACTGGACATTTCACCTTATACC

>Cne_PSG13N (Cercopithecus neglectus; De Brazza's monkey) WGS PVKI010228178.1

CATCACTTTTAATCTTCTGGAACACGCCCACCACTGCGCAAGTCACGATTGAAGCACAGCCAACCAAAGTTTCTGAGGGGAAGGATGTTCTTCTACTTGTCCACAATTTGCCCCAGAATCTTACTGGCTACATCTGGTATAAAGGGCAAAAAATGGACCTCCACCATTACATTACATCATATGTAATAGACGCTGAAACAATTATAGCTGGGCCTGCATACAGTGGACGAGAAATAGTATATTCCAATGCATCCCTACTGATCCAGAATGTCACCCGGAAGGACACAGGATCCTACACCATACAAACCATAAAGCAAGGTGATAACAGTAAAGGAGTAATTGGACATTTCACCTTATACC

>Cne_PSG14N (Cercopithecus neglectus; De Brazza's monkey) WGS PVKI010023673.1

CATCACTTTTAAACTTCTGGAACCCACCCACCACTGCTCAAGTCACAATTGAAGCCCAGCCAGCCAAAGTTCCCGAGGGGAAGGATGTTCTTCTACTTGTCCACAATTTGCCCCAGAATCTTACTGGCTACATCTGGTACAAAGGGCAAATAACGGACCACCACCATTACATTACATCATATGTGATAGACACTGAAACAATTATATTTGGGTCTGCATACAGTGGACGAGAAACAGTATATTCCAATGCATCCCTGCTGATCCAGAATGTGACCCGGAACGACACAGGATCCTACACCATACAAATCATAAAGCGAGGTGATAGGACTGAAGGAATAACTGGACATTTCACCTTATACC

>Cne_PSG15N (Cercopithecus neglectus; De Brazza's monkey) WGS PVKI010031202.1

TATCAATTTTAATCATCTGGAACCGTCCTACCACGGCTCAAGTCATGATTGAAGCTCAGCCAACCGAGGTTTCTGAGGGGAAGGATGTTCTTCTACTTGTCCACAATTTGCCCCAGAACCCTACTGGCTACAGCTGGTACAAAGGGCAAATAACAGACATCCACCATTACATTACATCATATGTAATAGACACTGAAATTATTATATTTGGGCCTGCATACAGTGGACGAGAAACAGTATTTTCCAATGCATCCCTGCTGATCCAGAATGTCACCCAGAAGGACACAGGATCCTACACCATACGAATCATACAGCGAGGTGATACCACTAAAGGAGTAACTGGACGTTTCACCTTATACC

>Cne_PSG16N (Cercopithecus neglectus; De Brazza's monkey) WGS PVKI010091282.1

TATTACTTTTCATCTTCTGGAACCCTCCCACCACTGCCCAAGTCACGATTGAAGCCCAGCCAGCCAAAGTTTCCGAGGGGAAAGATATTCTTCTACTTGTCCAGAATTTGCCCCAGAATCTTACTGGCTACATCTGGTTCAAAGGGCAAATAACGAATTTCCACCAATTCATTATAGCATATACAATAGACAGTAAAAAAATTACAGTTGGGCCTGCATACAGTGGACGAGAAAGAGTATATTCCAATGCATCCCTGCTGATCCAGAATGTCACCCAGAAGGACACAGGATCCTACACCATACAAATTACAAAGCGAGGTGATAAGATTAAAGGAGTAACTGGACATTTCACCTTATATG

>Cne_PSG17N (Cercopithecus neglectus; De Brazza's monkey) WGS PVKI010022557.1

CATCATTTTTAATCTTGTGGAACACGCCCACCACTGCCCAAGTCATGATTGAAGCTCAGCCTACCAAAGTTTCTGAGGGGAAGGATGTTCTTCTACTTGTCCGCAATTTGCCCCAGAAAGTTTCTGGCTATGTCTGGTACAAAGGGCAAATAATGGACTTCCACCAATTCATTACAGCATATACAATAGACACTGAAAGAATTATATTTGGGACTGCATACAGTGGACGAGAAACACTATATTCCAATGGATCCCTGCTGATCCGGAATGTCACCAAGCAGGACACAGGATCCTACACCGTAAAAATCATAGAGCGAGCTGAGGAGACTAAAGAAGTAACTGTACATTTCACCTTATACC

>Cne_PSG18N (Cercopithecus neglectus; De Brazza's monkey) WGS PVKI010383241.1

TATTACTTTTCATCTTCTGGAACACGCCCACCACTGCCCAAGTCACGATTGAAGCCCAGCCAACTAAAGTTTCCGAGGGGAAAGATATTCTTCTACTTGTGCAGAATTTGCCCCAGAATCTTACTGGCTACATCTGGTTCAAAGGGCATATAACGAACTACCATCAATTCATTATTGCATATGCAATAGACAGTAAAAATATTACAGTTGGGCCTGCATACAGTGGACGAGAAAGAGTATATTCCAATGCATCCCTGCTGATCCAGAATGTCACCCAGAAGGACACAGGATCCTACACCATAGAAATCATAAAGCAAGGTGATAAGACTAAAGGAGTAACTGGACATTTCACCTTATATG

>Cne_PSG19N (Cercopithecus neglectus; De Brazza's monkey) WGS PVKI010332940.1

TATTACTTTTCATCTTCTGGAACACGCCCACCACTGCCCAAGTCACGATTGAAGCCCAGCCAACTAAAGTTTCCGAGGGGAAAGATATTCTTCTACTTGTCCAGAATTTGCCCCAGAATCTTACTGGCTACGTCTGGTTCAAAGGGCAAATAACGAACTACCATCAATTCATTATTGCATATGCAATAGACAGTAAAAATATTACAGTTGGGCCTGCATACAGTGGACGAGAAACAGTATATTCCAATGCATCCCTGCTGATCCAGAATGTCACCCAGAAGGACACAGGATCCTACACCATAGAAATCATAAAGCAAGGTGATAAGACTAAAGGAGTAACTGGACATTTCACCTTATACG

>Cne_PSG20N (Cercopithecus neglectus; De Brazza's monkey) WGS PVKI010299940.1

TATTACTTTTCATCTTCTGGAACCCGCCCACCACTGCCCAAGTCACGATTGAAGCCCAGCCAGCCAAAGTTTCTGAGGGGAAAGATATTCTTCTACTTGTGCAGAATTTGCCCCAGAATCTTACTGGCTACGTCTGGTTCAAAGGGCAAAAAACGAACTACCATCAATTCATTATTGCATATGCAATAGACAGTAAAAATATTACAGTTGGGCCTGCATACAGTGGACGAGAAACAGTATATTCCAATGCATCCCTGCTGATCCAGAATGTCACCCAGAAGGTCACAGGATCCTACACCATACAAATTATAAAGCGAGGTGATAAGATTAAAGGAGTAACTGGACATTTCACCTTATACG

>Cne_PSGP1N (Cercopithecus neglectus; De Brazza's monkey) WGS PVKI010081550.1 no ORF

CATCACTTTTAATCTTCTGGAACCCGCCCACCACTGCCCAAGTCACGATTGAAGCACAGCCAACCAAAGTTTCTGAGGGGAAGGATGTTCTTCTACTTGTCCACAATTTGCCCCAGAATGTTGCTGGCTATATGTGGTACAAAGGGCAAATAATGGACCTCCAGCATTACATTACAGCATATGAAATAGACACTGAAACAATTATATTTGGGCCTGCATACAGTGGATGAGAAACAGTATATTCCAATGCATCCCTGCTGATCCAGAGTGTCAACCAGAAGGATGCAGGATCCTACACCATAGAAATCATAAAGAGAGGTGAAGGGACTGAAGGAGTAACTGGACATTTCACCTTATACC

>Cne_PSGP2N (Cercopithecus neglectus; De Brazza's monkey) WGS PVKI010362859.1 no ORF

CATCACTTTTAATCTTCTGGAACTCACCTACCACTGCTCAAGTCACGATTGAAGCCCAGCCAACCAAAGTTTCTGAGGGGAAAGATGTTCTTCTACTTGTCCACAATTTGCCCCAGAATCTTACTGGCTACATCTGGTACAAAGGGCAAAAAACGGACCTCCACCTTTACGTTACATCATATGTAAAAGACACTGAAACAATTATAGCTGGGCCTGCATACAGTGGATGAGAAACAGTATATTCCAATGCATCCCTGCTGATCCAGAATGTCACCCAGAAGGACACAGGATCCTACACCATACAAATCACAAAGCGAGGTGATAGGACTGAAGGAGAAACTGCACATTTCACCTTATATC

>Cne_PSGP3N (Cercopithecus neglectus; De Brazza's monkey) WGS PVKI010159928.1 no ORF

CATCACTTTTAATCTTCTGGAACCCGCCCACCACTGCTCAAGTCACAATTGAAGCCCAGCCAACCAAAGTTCCCGAGGGGAAGGATGTTCTTCTACTTGTCCACAATTTGCCCCAGAATCTTACTGGTTACATCTGGTACAAAGGGCAAATAATGGACCTCCACCATTATATTACATCATATGTGATAGACATTGAAACAATTATATTTGGGCCTGCATACAGTGGACGAGAAACAGTATATTCCAATGCATCCCTGCTGATCTAGAATGTCACCCGGAAGGACACAGGATCCTACACCATACAAATCATAAAGCGAGGTGATAGGACTGAAGGAGTAACTGAACATTTCACCTTATACC

>Cne_PSGP4N (Cercopithecus neglectus; De Brazza's monkey) WGS PVKI010402691.1 no ORF

TATTACTTTTCATCTTCTGGAACCCGCCCACCACTGCCCAAGTCACAATTGAAGCCCAGTCAACCAAAGTTTCTGAGGGGAAGGATGTTCTTCTACTTGTCTACAATTTGCCCCAGAATCTTACTGGCTACGTCTGGTTCAAAGGGCAAATAATGGACTTCCACCAATTCATTACAGCGTATACGATACACACTGAAACTATTATATTTGGGCCTGCATACAGTGGATGAGAAACAGTATATTCCAATGCATCCCTGCTGATCCAGAATGTCACCCAGAAGGTCACAGGATCCTACACCATAGAAATTATAAAGCGAGGTGATAAGATTAAAGGAGTAACTGGACATTTCACCTTATACG

>Cne_PSGP5N (Cercopithecus neglectus; De Brazza's monkey) WGS PVKI010274648.1 no ORF

TATCACTTTTAATCATCTGGAACCCGCCTACCACGGCTCAAGTCACGATTGAAGCTCAGCCAACCGAGGTTGCTGAAGGGAAGGATGTTCTTCTACTTGTCCACAATTTGCCCCAGAATCCTACTGGCTACAGCTGGTACAAAGGGCAAATAACAGACATCCACCATTATATTACATCATATGTAATAGACACTGAAATTATTATATTTGGGCCTGCATACAGTGGACGAGAAACAGTATTTTCCAATGCATCCCTGCTGATCCAGAATGTCACCCAGAAGGACACAGGATCCTACACCATACAAATCATACAGTGAGGTGATACCACTAAAGGAGTAACTGGACATTTCACCTTATACC

>Csa_PSG1N (Chlorocebus sabaeus; green monkey) WGS AQIB01158203.1; XM_008019820.1

CATCACTTTTAATCTTCTGGAACCTGCCCACCACTGCCCAAGTCGTGATTGAAGCCCAGCCAGCCAAAGTTTCCGAGGGAAAGGATGTTCTTCTACTTGTCCACAATTTGCCCCAGAATCTCGCTGCCTGCATCTGGTACAAAGGGCAAATAATGGACCTCCAACATTACATTACAGCATATGTAAAAGATGCTGAAACAATTATATTTGGGCCTGCATACAGTGGACGAGAAACAGTATATTCCAATGCATCCCTGCTGATCCAGAATGTCACCCAGAAGGACACAGGATCCTACACCATACAAATCATAAAGCGAGGTGATAAGACTAAAGGAGTAACTGGACATTTCACCTTATACG

>Csa_PSG2N (Chlorocebus sabaeus; green monkey) WGS AQIB01158173.1; XM_008019817.1

CATCACTTTTAATCTTCTGGAACCCGCCTACCACTGCCCAAGTCACGATTGAAGCACAGCCAGCCAAAGTTTCCGAGGGGAATGATGTTCTGCTACGTGTCCACAATTTGCCCCAGAATCTTGCTGCCTACATCTGGTACAAAGGGCAAATTATGGACCTCCACCATTACATTACATCATATGTAATAGACACTGAAATAATTGTATTTGGGCATGCATATAGTGGACGAGAAACAGTATATTCCAATGCATCCCTGCTGATCCAGAATGTCACCCAGAAGGACACAGGATCCTATACCATACAAATCATAATGCGAGGAGATAGGACTAAAGGAGTAACTGGACATTTCACCTTATACC

>Csa_PSG3N (Chlorocebus sabaeus; green monkey) WGS AQIB01155303.1

CATCACTTTTAATCTTCTGGAACCCGCCCACCACTGCTCAAGTCACAGTTGAAGCCCAGCCAACCAAAGTTTCCGAGGGGAAGGATGTTCTTCTACTTGTCCACAATTTGCCCCAGAATCTTACTGGCTACAGCTGGTACAAAGGGCAAATAACGGACCACCACCATTACATTACATCATATGTGATAGACACTGAAACAATTATATTTGGGCCTGCATACAGTGGACGAGAAACAGTATATTCCAATGCATCCCTGCTGATCCAGAATGTCACCCGGAACGACACAGGATCCTACACCATACAAATCATAAAGCGAGGTGATAGGACTGAAGGAATAACTGGACATTACACCTTATACC

>Csa_PSG4N (Chlorocebus sabaeus; green monkey) WGS AQIB01156380.1

CATCACTTTTAATCTTCTGGAACCCGCCCACCACTGCCCAAGTCACGATTGAAGCTCAGCCAACCAAAGTTTCCGAGGGGAAGGATGTTCTTCTACTTGTCCACAATTTGCCCCAGAATGTTGCTGGCTACATCTGGTACAAAGGGCAAATAATGGACCTCCAGCATTACATTATAGCATATACAATAGACACTGAAATGATTATATTTGGGTCTGCATACAGTGGACGAGAAACAGTATATTCCAATGCATCCCTGCTGATCCAGAATGTCACCAAGAATGACACAGGATCCTACACCATTCAAATCAGAAAGCGAGGTGATGAGAGTAAAGGAGTAACTGGACATTTCACCTTATACC

>Csa_PSG5N (Chlorocebus sabaeus; green monkey) WGS AQIB01156665.1; XM_008019763.1

CATCACTTTTAATCTTCTGGAACCCGCCCACCACTGCACAAGTCAGGATTGAAGCTCAGCCAACCAAAGTTTCTGAGGGGAAGGATATTCTTCTACTTGTCCACAATTTGCCCCAGAATGTTGCTGGCTACATCTGGTACAAAGGGCAAATAATGGACCTCCAGTATTACATTACAGCATATGCAATAGACACTGAAATGCTTATATTTGGGCCTGCATACAGTGGACGAGAAACAGTATATTCCAATGCATCCCTGCTGATCCAGAGTGTCAACCAGAAGGATGCAGGATCCTACACCGTAGAAATCATAAAGCGAGGTGAAGGGACTGAAGGAGTAACTGGACATTTCACCTTATACC

>Csa_PSG6N (Chlorocebus sabaeus; green monkey) WGS AQIB01155314.1; XM_008019108.1

CATCATTTTTAATCTTCTGGAACCCGCCCACCACTGCCCAAGTCACGATTGAAGCACAGCCAACCAAAGTTTCTGAGGGGAAGGATGTTCTTCTACTTGTCCACAATTTGCCCCAGAATGTTGCTGGCTACAGCTGGTACAAAGGGCAAATAATGGACCTCCACCATTACATTACAGCATATACAATAGACACTGAAATGATTATACTTGGGCCCGCATACAGTGGACGAGAAACTGTATATTCCAATGCATCCCTGCTGATCCAGAATGTCACCCAGAAGGACACAGGATCCTACACCATTCAAATCACAAAGCGAGGTGATGGGACTAAAAGAGTAACTGGACATTTCACTTTATACC

>Csa_PSG7N (Chlorocebus sabaeus; green monkey) WGS AQIB01155309; XM_008019110.1

CATCACTTTTTATCTTCTGGAACCCGCCCACCACTGCCCAAGTCACGATCGAAGCCCAGCCAACCAAAGTTTCCAAGGGGAAGGATGTTCTTCTACTTGTCCACAATTTGCCCCAGAATCTTACTGGCTACAGCTGGTACAAAGGGCAAATAATGGACCTCCAGCATTACATTGCAGCATATACAATAGACACTGAAATGATTATATTTGGGCCTGCATACAGTGGACGAGAAACAGTATATTCCAATGCATCCCTGCTGATCCAGAATGTCACCAAGAATGACACAGGATCCTACACCATTCAAATCAGAAAGCGAGGTGATAAGACTAAAGGAGTAACTGGACATTTCACCTTATACC

>Csa_PSG8N (Chlorocebus sabaeus; green monkey) WGS AQIB01155308.1; XR_502619.1

CATCACTTTTAATCTTCTGGAACCCGCCCACCACTGCCCAAGTCACGATTGAAGCACAGCCAACCAAAGTTTCTGAGGGGAAGGATATTCTTCTACTTGTCCACAATTTGCCCCAGAATGTTGCTGGCTACATCTGGTACAAGGGGCAAATAATGGACCTCCAGTATTACATTACAGCATATGCAATAGACACTGAAATGCTTATATTTGGGCCCGCATACAGTGGACGAGAAACAGTATATTCCAATGCATCCCTGCTGATCCAGAGTGTCAACCAGAACGATGCAGGATCCTACACCGTAGAAATCATAAAGCGAGGTGAAGTGACTGAAGGAGTAACTGGACATTTCACCTTATACC

>Csa_PSG9N (Chlorocebus sabaeus; green monkey) WGS AQIB01156203.1

GATTACTTTTCATCTTCTGGAACCCGCCCACCACTGCCCAAGTCACGATTGAAGCTCAGCCAACCAAAATTTACGAGGGGAAGGATGTTCTTCTACTTGTCCAGAATTTGCCCCAGAATCTTACTGGCTACGTCTGGTTCAAAGGGCAAATAATGGACCTCCACCAATTCATTACAGCGTATACGATAGACACTGAAACAATTATATTTGGGCCTGCATACAGTGGACGAGAAACAGTATATTCCAATGCATCCCTGCTGATCCAGAATGTCACCCAGAATGACACAGGATCCTACACCATAGAAATTATAAAGCGAGGTGATAAGATTAAAGGAGTAACTGGACATTTCACCTTATACC

>Csa_PSG10N (Chlorocebus sabaeus; green monkey) XM_008019805.1

TATCACTTTTAATCATCTGGAACCCGCCTACCACGGCTCAAGTCACAATTGAAGCTCAGCCAACCGAGGTTTCTGAGGGGAAGGATGTTCTTCTACTTGTCCACAATTTGCCCCAGAATCCTACTGGCTACAGCTGGTACAAAGGGCAAATAACAGACATCCACCATTATATTACATCATATGTAATAGACACTGAAATTATTATATTTGGGCCTGCATACAGTGGACGAGAAACAGTATTTTCCAATGCATCCCTGCTGATCCAGAATGTCACCCAGAAGGACACAGGATCCTACACCATACAAATCATACAGCGAGGTGATACCACTAAAGGAGTAACTGGACATTTCATCTTATACC

>Csa_PSG11N (Chlorocebus sabaeus; green monkey) WGS AQIB01156013.1

CATCACTTTTAATCTTCTGGAACCCACCCACCACTGCTCAAGTCACGATTGAAGCCCAGCCAACCAAAGTTTCCGAGGGGAAAGATGTTCTTCTACTTGTCCACAATTTGCCCCAGAATCTTACTGGCTACATCTGGTACAAAGGGCAAAAAACGGACCTCCACCTTTACGTTACATCATATGTAAAAGACACTGAAACAATTATAGCTGGGCCTGCATACACTGGACGAGAAACAGTATATTCCAATGCATCCCTGCTGATCCAGAATGTCACCCAGAAGGACACAGGATCCTACACCATACAAATCACAAAGCGGGGTGATAGGACTGAAGGAGAAACTGCACATTTCACCTTATATC

>Csa_PSG12N (Chlorocebus sabaeus; green monkey) XM_008019750.1

CATCACTTTTAATCTTCTGGAACCCGCCTACCACTGCCCAAGTCACAATTGAAGCACAACCAGCCAAAGTTTCCGAGGGGAATGATGTTCTGCTACGTGTCCACAATTTGCCCCAGAATCTTGCTGCCTACATCTGGTACAAAGGGCAAATTATGGACCTCCACCATTACATTACATCATATGTAATAGACACTGAAGTAATTGTATTTGGGCCTGCATACAGTGGACGAGAAACAGTATATTCCAATGCATCCCTGCTGATCCAGAATGTCACCCAGAAGGACACAGGATCCTACACCATACAAATCATAATGCGAGGAGATAGGACTAAAGGAGTAACTGGACATTTCACCTTATACC

>Csa_PSG13N (Chlorocebus sabaeus; green monkey) XM_008019107.1

CATTACTTTTCATCTTCTGGAACCCACCTACCACGGCCCAAGTCACGATTGAAGCTCAGCCAATTGAAATTTCTGAGGGGAAGGATGTTCTTCTACTTGTCCACAATTTGCCCCAGAATCCTACTGGCTACATCTGGTACAAAGGGCATATAATGGAGAGCCACAATTACTTTACATCATATGTAATAGACACTGAAATGATTATATTTGGGTCTGCATACAGTGGACGAGAAACAGTATATTCCAATGCATCCCTGCTGATCCAGAATGTCACCCAGAAGGACACAGGATCCTACACCATACAAATCATAAAGCGAGGTGATAGCACTAAAGGAGTAACTGGACATTTCACCTTATACC

>Csa_PSG14N (Chlorocebus sabaeus; green monkey) XM_008019770.1

TATTACTGTTCATCTTCTGGAACCCGCCCACCACTGCCCAAGTCACGATTGAAGCCCAGCCAACCAAAGTTTCCGAGGGGAAAGATATTCTTCTACTTGTCCAGAATTTGCCCCAGAATCTTACTGGCTACGTCTGGTTCAAAGGGCAAATAACGAACTACCATCAATTCATTATTGCATATGCAATAGACAGTAAAAATATTACAGTTGGGCCTGCATACAGTGGACGAGAAACAGTATATTCCAATGCATCCCTGCTGATCCAGAATGTCACCCAGAAGGTCACAGGATCCTACACCATAGAAATTATAAAGCGAGGTGATAAGATTAAAGGAGTAACTGGACATTTCACGTTATACG

>Csa_PSG15N (Chlorocebus sabaeus; green monkey) WGS AQIB01155313.1

CATCATTTTTAATCTTGTGGAACACGCCCACCACTGCCCAAGTCATGATTGAAGCTCAGCCTACCAAAGTTTCTGAGGGGAAGGATGTTCTTCTACTTGTGCGCAATTTGCCCCAGAAAGTTGCTGCCTACGTCTGGTACAAAGGGCAAATAATGGACTTCCACCAATTCATTACAGCATATACAATAGACACTGAAAGATTTATATTTGGGCCTGCATGCAGTGGACGAGAAACACTATATTCCAATGGATCCCTGCTGATCCGGAATGTCACCAAGCAGGACACAGGATCCTACACCGTAAAAATCATAGAGCGAGCTGAGGAGACTAAAGGAGTAACTGTACATTTCACCTTATACC

>Csa_PSGP1N (Chlorocebus sabaeus; green monkey) WGS AQIB01156008.1

CATCACTTTTAATCTTCTGGAACCTGCCTACCACTGCCCAAGTCATGATTGAAGCACAGCCAGCCAAAGTTTCCGAGGGGAAGGATGTTCTGCTACGTGTCCACAATTTGCCCCAGAATCTTGCTGCCTACATCTGGTACAAAGGGCAAATTATGGACCTCCACCATTACATTACATCATATGTAATAGACACTGAAATAATTGTATTTGGGCCTGCATACAGTGGACGAGAAACAGTATATTCCAATGCATCCCTGCTGATCCAGAATGTCACCTAGAAGGACACAGGATCCTACACCATACAAATCATAATGCGAGGAGATAGGACTAAAGGAGTAACTGGACATTTCACCTTATACC

>Csa_PSGP2N (Chlorocebus sabaeus; green monkey) XM_008019771.1

CATCACTTTTAATCTTCTGGAACCCGCCCACTACTGCCCAAGTTGTGATTGAAGCCCAGCCAGCCAAAGTTTCCGAGGGGAAGGATGTTCTTCTACTTGTCCACAATTTGCCCCAGAATCTTGCTGCCTGCATCTGGTAAAAAGGGCAAATAATGGACCTCCAATATTACATTACAGCATATGTAAGAGATGCTGAAACAATTATATTTGGGCCTGCATACAGTGGACGAGAAACAGTATATTCCAATGCATCCCTACTGATCCAGAATGTCACCCAGAAGGACACAGGATCCTACACCATACAAATCATAAAGCGAGGTGATAAGACTAAAGGAGTAACTGGACATTTCACCTTATACC

>Csa_PSGP3N (Chlorocebus sabaeus; green monkey) WGS AQIB01155312.1

CATCACTTTTCATCTTCTGGAACCCACCTACCACGGCCCAAGTCACAATTGAAGCTCAGCCAACTGAAGTTTCTGAGGGGAAGGATGTTCTTCTACTTGTCCACAATTTGCCCCAGAATGTTGCTGGCTACATCTGGTACAAAGGCCAAATAATGGACCTCCAGCATTACATTACAGCGTATACAATAGACACTGAAATGATTATATTTGGGCCTGCATACAGTGGACGAGAAACAGTATATTCCAATGCATCCCTGCTGATCCAGAATGTCACCCAGAAGGACACAGGATCCTACACCATACAAATCATAAAGCGAGGTGATAGCACTTAAAGAGTAACTGGACATTTCACCTTATACC

>Csa_PSGP4N (Chlorocebus sabaeus; green monkey) XM_008019841.1

CATCACTTTTAATTTTCTGGAACTCGCCAACCACTGCCCAAGTCAGGATTGAAGCTCAGCCAACCAAAGTTTCTGAGGGGAAGGATATTCTTCTACTTGTCCACAATTTGCCCCAGAATGTTGCTGGCTACATCTGGTACAAAGGGCAAATAATGGACCTCCAGCATTACATTACAGCATATACAATAGACACTGAAACAATTATATTTGGGCCTGCATACAGTGGATGAGAAACAGTATATTCCAATGCATCCCTGCTGATCCAGAGTGTCAACCAGAAGGATGCAGGATCCTACACTGTAGAAATCATCATAAAGCGAGGTGATGGGACTGAAGGAGTAACTGGACATTTCACCTTATACC

>Csa_PSGP5N (Chlorocebus sabaeus; green monkey) XM_008019784.1

CATCACTTTTAATCTTCTGGAACTCGCCCACCACTGCCCAAGTCACAATTGAAGCTCAGCCAACCAAAGTTTCTGAGGGGAACGATGTTCTTCTACTTGTACACAATTTGCCCCAGAATCCTGCTGCCTACATCTGGTACAAAGGGCAAATAATGGACCTCCAGCATTACATTACAGCATATGCAATAGACACTGAAAGAATTGTATTTGGGCCTGCATACAGTGGACGAGAAAGAGTATATTCCAATGCATCCCTGATGATCCAGAGTGTGAACCAGAAGGACGCAGGATCCTACACCGTAGGAATCATCATAAAGCGAGGTGATAGGACTGAAAGAGTAACTTGACATTTCACCTTATACG

>Epa_PSG1N (Erythrocebus patas; red guenon) WGS PVJV010029220.1

CATCACTTTTAATCTTCTGGAACCCGCCCACCACTGCCCAAGTCACGATTGAAGCTCACCCAACCAAAGTTTCCGAGGGGAAGGATGTTCTTCTACTTGTCCACAATTTGCCCCAGAATCTTACTGGCTACATCTGGTACAAAGGGCAAATAATGGACCTCCAGCATTACATTACAGCATATACAATAGACACTGAAATGATTATATTTGGGTCTGCATACAGTGGACGAGAAACAGTATATTCCAATGCATCCCTGCTGATCCAGAATGTCACCAAGAATGACACAGGATCCTACACCATTCAAATCACAAAGCGAGGTGATGAGAGTAAAGGAGTAACTGGACATTTCACCTTATACC

>Epa_PSG2N (Erythrocebus patas; red guenon) WGS PVJV010077887.1

CATCACTTTTAATCTTCTGGAACCCGCCCACCACTGCCCAAGTCACGATCGAAGCCCAGCCAACCAAAGTTTCTGAGGGGAAGGATGTTCTTCTACTTGTCCACAATTTGCCCCAGAATCTTACTGGCTACAGCTGGTACAAAGGGCAAATAATGGACCTCCACCATTACATTGCAGCATATATAATAGACACTGAAATGATTATATTTGGGCCTGCATACAGTGGACGAGAAACAGTATATTCCAATGCATCCCTGCTGATCCAGAATGTCACCAAGAATGACACAGGATCCTACACCATTCAAATCACAAAGCGAGGTGATGAGAGTAAAGGAGTAACTGGACATTTCACCTTATACC

>Epa_PSG3N (Erythrocebus patas; red guenon) WGS PVJV010083747.1

CATCACTTTTAATCTTCTGGAACCCGCCCACCACTGCCCAAGTCACGATTGAAGCCCAGCCAACCAAAGTTTCTGAGGGGAAGGATGTTCTTCTACTTGTCCACAATTTGCCCCAGAATGTTGCTGGCTACATCTGGTACAAAGGGCAAATAATGGACCTCCAGCATTATATTACAGCATATACAATAGACACTGAAATGATTGTATTTGGGCCTGCATACACTGGACGAGAAACAGTATATTCCAATGCATCCCTGCTGATCCAGAATGTCACCCAGAATGACACAGGATCCTACACCATTCAAATCACAAACCGATGTGATGAGACTAAAGGAGTAACTGGACATTTCGCCTTATACT

>Epa_PSG4N (Erythrocebus patas; red guenon) WGS PVJV010025917.1

CATCATTTTTAATCTTCTGGAACCCGCCCACCACTGCCCAAGTCATGATTGAAGCACAGCCAACCAAAGTTTCTGAGGGGAAGGATGTTCTTCTACTTGTCCACAATTTGCCCCAGAATGTTGCTGGCTACAGCTGGTACAAAGGGCAAATAATGGACCTCCACCATTACATTACAGCATATACAATAGACACTGAAATGATTATATTTGGGCCTGCATACAGTGGACGAGAAACTGTATATTCCAATGCATCCCTGCTGATCCAGAATGTCACCCAGAATGACACAGGATCCTACACCATTCAAATCACAAAGCGAGGTGATGGGACTAAAAGAGTAACTGGACATTTCACTTTATACC

>Epa_PSG5N (Erythrocebus patas; red guenon) WGS PVJV010050926.1

CATCACTTTTCATCTTCTGGAACCCACCTACCACGGCCCAAGTCACAATTGAAGCTCAGCCAACTGAAGTTTCTGAGGGGAAGGATGTTCTTCTACTTGTCCACAATTTGCCCCAGAATGTTGCTGGCTACAGCTGGTACAAAGGCCAAATAATGGACCTCCAGCATTACATTACAGCGTATACAATAGACACTGAAATGATTATATTTGGGCCTGCACACAGTGGACGAGAAACAGTATATTCCAATGCATCCCTGCTGATCCAGAATGTCACCCAGAAGGACACAGGATCCTACACCATACAAATCATAAAGCGAGGTGATAGCACTAAAAGAGTAACTGGACATTTCACCTTATACC

>Epa_PSG6N (Erythrocebus patas; red guenon) WGS PVJV010079066.1

CATCACTTTTAATCTTCTGGAACCCGCCTACCACTGCCCAAGTCATGATTGAAGCACAGCCAGCCAAAGTTTCCGAGGGGAAGGATGTTCTGCTACGTGTCCACAATTTGCCCCAGAATCTTGCTGCCTACATCTGGTACAAAGGGCAAATTATGGACCTCCAACATTACATTACAGCATATGTAATAGACACTGAAATAATTGTATTTGGGCCTGCATACAGTGGACGAGAAACAGTATATTCCAATGCATCCCTGCTGATCCAGAATGTCACCCAGAAGGACACAGGATCCTACACCATACAAATCATAATGCGAGGAGATAGGACTAAAGGAGTAACTGGACATTTCACCTTATACG

>Epa_PSG7N (Erythrocebus patas; red guenon) WGS PVJV010085191.1

CATCACTTTTAATCTTCTGGAACCCGCCCACCACTGCCCAAGTCACGATTGAAGCACAGCCAACCAAAGTTTCCGAGGGGAAGGATGTTCTTCTACTTGTCCACAATTTGCCCCAGAATCTTATTGGCTACAGCTGGTACAAAGGGCAAATAATGGACCTCCACCATTACATCACATCATATGTAATAGACACTGAAATGATTGTATTTGGGCCCGCATACAGTGGACGAGAAACAGTATATTCCAATGCATCCCTGCTGATTCAGAATGTCACCCGGAAGGACACAGGATCCTACACCATACAAATTATAAAGCGAGGTGATAAGATTAAAAGAATAACTGGACATTACACCTTATACC

>Epa_PSG8N (Erythrocebus patas; red guenon) WGS PVJV010036770.1

CATCACTTTTAATCTTCTGGAACCCGCCCACCACTGCCCAAGTCACGATTGAAGCACAGCCAACCAAAGTTTCTGAGGGGAAGGATATTCTTCTACTTGTCCACAATTTGCCCCAGAATGTTGCTGGCTACATCTGGTACAAAGGGCAAATAATGGACCTCCAGTATTACATTACAGCATATGCAATAGACACTGAAATGCTTATATTTGGGCCTGCATACAGTGGACGAGAAACAGTATATTCCAATGCATCCCTGCTGATCCAGAGTGTCAACCAGAAGGATGCAGGATCCTACACCGTAGAAATCATAAAGCGAGGTGAAGTGACTGAAGGAGTAACTGGACATTTCACCTTATACC

>Epa_PSG9N (Erythrocebus patas; red guenon) WGS PVJV010195844.1

nATCACTTTTAATCTTCTGGAACCCGCCCACCACTGCGCAAGTCAGGATTGAAGCTCAGCCAACCAAAGTTTCTGAGGGGAAGGATATTCTTCTACTTGTCCACAATTTGCCCCAGAATGTTGCTGGCTACATCTGGTACAAAGGGCAAATAATGGACCTCCAGTATTACATTACAGCATATGCAATAGACACTGAAATGATTATATTTGGGCCTGCATACAGTGGACGAGAAACACTATATTCCAATGCATCCCTGCTGATCCAGAGTGTCAACCAGAAGGATGCAGGATCCTACACCATAGAAATCATAAAGCGAGGTGAAGGGACTGAAGGAGTAACTGGACATTTCACCTTATACC

>Epa_PSG10N (Erythrocebus patas; red guenon) WGS PVJV010194933.1

nATCACTTTTAATCTTCTGGAACCCGCCCACCACTGCGCAAGTCAGGATTGAAGCTCAGCCAACCAAAGTTTCTGAGGGGAAGGATATTCTTCTACTTGTCCACAATTTGCCCCAGAATGTTGCTGGCTACATCTGGTACAAAGGGCAAATAATGGACCTCCAGTATTACATTACAGCATATGCAATAGACACTGAAATGCTTATATTTGGGCCTGCATACAGTGGACGAGAAACACTATATTCCAATGCATCCCTGCTGATCCAGAGTGTCAACCAGAAGGATGCAGGATCCTACACCATAGAAATCATAAAGCGAGGTGAAGGGACTGAAGGAGTAACTGGACATTTCACCTTATACC

>Epa_PSG11N (Erythrocebus patas; red guenon) WGS PVJV010025522.1 splice donor GTGATT

CATCACTTTTCATCTTCTGGAACCCACCTACCACGGCCCAAGTCACGATTGAAGCTCAGCCAATTGAAGTTTCTGAGGGGAAGGATGTTCTTCTACTTGTCCACAATTTGCCCCAGAATCCTACTGGCTACATCTGGTACAAAGGGCAAATAACGGAGAGCCACAATTACATTACGTCATATGTAATAGACACTGAAATGATTATATTGGTGCCTGCATACAGTGGACGAGAAACAGTATATTCCAATGCATCCCTGCTGATCCAGAATGTCACCCAGAAGGACACAGGATCCTACACCATACAAATCATAAAGCGAGGTGATAGCACTAAAGGAGTAACTGGACATTTCACCTTATACC

>Epa_PSG12N (Erythrocebus patas; red guenon) WGS PVJV010021434.1

TATCACTTTTAATCATCTGGAACCCGCCTACCACGGCTCAAGTCACGATTGAAGCTCAGCCAACCGAGGTTTCTGAGGGGAAGGATGTTCTTCTACTTGTCCACAATTTGCCCCAGAATCCTACTGGCTACAGCTGGTACAAAGGGCAAATAACAGACATCCACCATTATATTACATCATATGTAATAGACACTGAAATTATTATATTTGGGCCTGCATACAGTGGACGAGAAACAGTATTTTCCAATGCATCCCTGCTGATCCAGAATGTCACCCAGAAGGACACAGGATCCTACACCATACAAATCATACAGCGAGGTGATACCACTAAAGGAGTAACTGGACATTTCACCTTATACC

>Epa_PSG13N (Erythrocebus patas; red guenon) WGS PVJV010050539.1

CATCACTTTTAATCTTTTGGAACCCACCCACCACTGCTCAAGTCACAGTTGAAGCCCAGCCAACCAAAGTTTCCGAGGGGAAGGATGTTCTTCTACTTGTCCACAATTTGCCCCAGAATCTTACTGGCTACATCTGGTACAAAGGGCAAATAACGGACCACCACCATTACATTACATCATATGTGATAGACACTGAAACAATTATATTTGGGCCTGCATACAGTGGACGAGAAACAGTATATTCCAATGCATCCCTGCTGATCCAGAATGTCACCCGGAACGACACAGGATCCTACACCATACAAATCATAAAGCGAGGTGATAGGACTGAAGGAATAACTGGACATTACACCTTATACC

>Epa_PSG14N (Erythrocebus patas; red guenon) WGS PVJV010067241.1

CATCACTTTTAATCTTCTGGAACTCGCCAACCACTGCCCAAGTCAGGATTGAAGCTCAGCCAACCAAAGTTTCTGAGGGGAAGGATATTCTTCTACTTGTCCACAATTTGCCCCAGAATGTTGCTGGCTACATCTGGTACAAAGGGCAAATAATGGACCTCCAGCATTGCATTACAGCATATACAATAGACACTGAAACAATTATATTTGGGCCTGCACACAGTGGACGAGAAACAGTATATTCCAATGCATCCCTGCTGATCCAGAGTGTCAACCAGAAGGATGCAGGATCCTACACTGTAGAAATCATCATAAAGCAAGGTGATGGGACTGAAGGAGTAACTGGACATTTCACCTTATACC

>Epa_PSG15N (Erythrocebus patas; red guenon) WGS PVJV010011321.1

CATCACTTTTAATCTTCTGGAACCCACCTACCACTGCTCAAGTCACGATTGAAGCCCAGCCAGCCAAAGTTTCCGAGGGGAAAGATGTTCTTCTACTTGTCCACAATTTGCCCCAGAATCTTACTGGCTACATCTGGTACAAAGGGCAAAAAACGGACCTCCACCTTTACGTTACATCATATGTAAAAGACACTGAAACAATTATAGCTGGGCCTGCATACAGTGGACGAGAAACAGTATATTCCAATGCATCCCTGCTGATCCAGAATGTCACCCAGAAGGACACAGGATCCTACACCATACAAATCACAAAGCGAGGTGATAGGACTGAAGGAGAAACTGCACATTTCACCTTATATC

>Epa_PSG16N (Erythrocebus patas; red guenon) WGS PVJV010080325.1

GATTACTTTTCATCTTCTGGAACCCACCCACCACTGCCCAAGTCACGATTGAAGCCCAGCCAACCAAAATTTCCAAGGGGAAGGATGTTCTTCTACTTGTCGAGAATTTGCCCCAGAATCTTACTGGCTACGTCTGGTTCAAAGGGCAAATAATGGACCTCCACCAATTCATTACAGTGTATACGATAGACACTGAAACAATTATATTGGTGCCTGCATACAGTGGACGAGAAACAGTATATTCCAATGCATCCCTGCTGATCCAGAATGTCACCCAGAATGACACAGGATCCTACACCATAGAAATTATAAAGCGAGGTGATAAGATTAAAGGAGTAACTGGACATTTCACCTTATACC

>Epa_PSG17N (Erythrocebus patas; red guenon) WGS PVJV010078331.1

CATCACTTTTAATCTTCTGGAACCTGCCCACCACTGCCCAAGTCGTGATTGAAGCCCAGCCAGCCAAAGTTTCCGAGGGGAAGGATGTTCTTCTACTTGTCCACAATTTGCCCCAGAATCTCGCTGCCTGCATCTGGTATAAAGGGCAAATAATGGACCTCCAACATTACATTACAGCATATGTAAAAGATGCTGAAACAATTATATTTGGGCCTGCATACAGTGGACGAGAAACAGTATATTCCAATGCATCCCTGCTGATCCAGAATGTCACCCGGAAGGACACAGGATCCTACACCATACAAATCATAAAGCGAGGTGATAAGACAAAAGGAGTAACTGGACATTTCACCTTATACG

>Epa_PSG18N (Erythrocebus patas; red guenon) WGS PVJV010055363.1

CATCACTTTTAATCTTCTGGAACTCGCCCACCACTGCCCAAGTTACAATTGAAGCTCAGCCAACCAATATTTCCGAGGGGAACGATGTTCTTCTACTTGTACACAATTTGCCCCAGAATCCTGCTGCCTACATCTGGTACAAAGGGCAAATAATGGACCTCCAGCATTACATTACAGCATATACAATAGACACTGAAAGAATTGTATTTGGGCCTGCATACAGTGGACGAGAAAGAGTATATTCCAATGCATCCCTGCTGATCCAGAGTGTGAACCAGAAGGACGCAGGATCCTACACCATAGAAATCATCATAAAGCAAGGAGATAGGACTGAAAGAGTAACTGGACATTTCACCTTATACC

>Epa_PSG19N (Erythrocebus patas; red guenon) WGS PVJV010071415.1

TATTACTTTTCATCTTCTGGAACCCGCCCACCACTGCCCAAGTCACGATTGAAGCCCAGCCAACCAAAGTTTCCGAGGGGAAAGATATTCTTCTACTTGTCCAGAATTTGCCCCAGAATCTTACTGGCTACGTCTGGTTCAAAGGGCAAATAACGAACTACCATCAATTCATTATAGCATATGCAAGAGACAGTAAAAATATTACAGTTGGGCCTGCATACAGTGGACGAGAAACAGTATATTCCAATGCATCCCTGCTGATCCAGAATGTCACCCAGAAGGACACAGGATCCTACACCATAGAAATCATAAAGCAAGGTGATGAGACTAAAGGAGTAACTGGACATTTCACCTTATACG

>Epa_PSG20N (Erythrocebus patas; red guenon) WGS PVJV010028321.1

CATCATTTTTAATCTTGTGGAACACGCCCACCACTGCCCAAGTCATGATTGAAGCTCAGCCTACCAAAGTTTCTGAGGGGAAGGATGTTCTTCTACTTGTCCGCAATTTGCCCCAGAAAGTTGCTGCCTATGTCTGGTACAAAGGGCAAATAATGGACTTCCACCAATTCATTACAGCATATACAATAGACACTGAAAGAGTTATATTTGGGCCTGCATGCAGTGGACGAGAAACACTATATTCCAATGGATCCCTGCTGATCCGGAATGTCACCAAGCAGGACACAGGATCCTACACCGTAAAAATCATAGAGCGAGCTGAGGAGACTAAAGGAGTAACTGTACATTTCACCTTATACC

>Epa_PSG21N (Erythrocebus patas; red guenon) WGS PVJV010015692.1

CATCACTTTTAATCTTCTGGAATACGCCCACCACTGTGCAAGTCACGATTGAAGCACAGCCAACCAAAGTTTCCGAGCGGAAGGATGTTCTTCTACTTGTCCACAATTTGCCCCAGAATCTTACTGGCTACATCTGGTATAAAGGGCAAAAAATGGACCTCCACCATTACATTATATCATATGTAATAGATGCTGAAACAATTATAGCTGGGCCTGCATACAGTGGACGAGAAATAGTATATTCCAATGCATCCCTACTGATCCAGAATGTCACCCGGAAGGACACAGGATCCTACACCATACAAACCATAAAGCAAGGTGATAACAGTAAAGGAGTAATTGGACATTTCACCTTATACC

>Epa_PSG22N (Erythrocebus patas; red guenon) WGS PVJV010076250.1

TATTACTTTTCATCTTCTGGAACCCGCCCACCACTGCCCAAGTCACGATTGAAGCCCAGCCAGCCAAAGTTTCGGAGGGGAAAGATATTCTTCTACTTGTCCAGAATTTGCCCCAGAATCTTACTGGCTACGTCTGGTTCAAAGGGCAAAAAACGAACTACCATCAATTCATTATTGCATATGCAATAGACAGTAAAAATATTACAGTTGGGCCTGCATACAGTGGACGAGAAAGAGTATATTCCAATGCATCCCTGCTGATCCAGAATGTCACCCAGAAGGTCACAGGATCCTACACCATAGAAATTATAAAGCGAGGTGATAAGATTAAAGGAGTAACTGGACATTTCACCTTATACG

>Epa_PSG23N (Erythrocebus patas; red guenon) WGS PVJV010040430.1

TATTACTTTTCATCTTCTGGAACCCGCCCACCACTGCCCAAGTCACGATTGCAGCCCAGCCAGCCAAAGTTTCCGAGGGGAAAGATATTCTTCTACTTGTCCAGAATTTGCCCCAGAATCTTACTGGCTATGTCTGGTTCAAAGGGCAAGAAAGGAACTACCACCAATTCATTATAGCATATGCAATAGAGAGTAAAAAAATTACAGTTGGGCCTGCATACAGTGGACGAGAAAGAGCATATTCCAATGCATCCCTGCTGATCCAGAATGTCACCCGGAAGGACACAGGATCCTACACCATAGAAATTATAAAGCGAGGTGATAAGATTAAAGGAGTAACTGGACATTTCACCTTATACA

>Epa_PSGP1N PSG5PN (Erythrocebus patas; red guenon) WGS PVJV010037484.1 no ORF

CATCACTTTTAATCTTCTGGAACCCGCCCACCACTGCCCAAGTCATGATTGAAGCCCAGCCAACCAAAGTTTCTAAGGGGAAGGATGTTCTTCTACTCGTCCACAATTTGCACCAGAATCTTACTGGCTACAGCTGGTACAAAGGGCAAATAATGGACCTCCACCATTACATTGCAGCATATACAATAGACACTGAAATGATTATATTTGGGCCTGCATACAGTGGACGAGAAACACTATATTCCAATGCATCCCTGCTGATCCAGAATGTCACCCAGAAGGACACAGGATCCTACACCATACAAATCATAAAGCGAGGTGATGAGAGTAAATGAGTAACTGGACATTTCACCTTATACC

>Ggo_PSG3N (Gorilla gorilla; gorilla) WSG SRLZ01001140.1

CATCACTTTTAAACTTCTGGAACCCGCCTACCACTGCCCAAGTCACGATTGAAGCTGAGCCAACCAAAGTTTCCAAGGGGAAGGATGTTCTTCTACTTGTCCACAATTTGCCCCAGAATCTTGCTGGCTACATCTGGTACAAAGGGCAAATGACGGACCTCTACCATTACATTACATCATACGTAGTAGATGGTCAAATAATTATATATGGGCCGGCATACAGTGGACGAGAAACAGTATATTCCAATGCATCCCTGCTGATCCAGAATGTCACCCGGGAGGACGCAGGATCCTACACCTTACACATCGTAAAGCGAGGTGATGGGACTAGAGGAGAAACTGGACATTTCACCTTCACCTTATACC

>Ggo_PSG4N (Gorilla gorilla; gorilla) WGS CABD030113773.1; SRLZ01001140.1; XM_019014736.1

CATCACTTTTAAACTTCTGGAACCTGCCTACCATTGCCCAAGTCACGATTGAAGCCCAGCCACCCAAAGTTTCTGAGGGGAAGGATGTTCTTCTACTTGTCCACAATTTGCCCCAGAATCTTGCTGGCTACATTTGGTACAAAGGGCAAATGACATACCTCTACCATTACATTACATCATATGCAGTAGACGGTCAAAGAATTATATATGGGCCTGCATACAGTGGAAGAGAAACAGTATATTCCAATGCATCCCTGCTGATCCAGAATGTCACCTGGGAGGACGCAGGATCCTACACCTTACACATCATAAAGCGAGGTGATGGGACTGGAGGAGTAACTGGACATTTCACCTTCACCTTATACT

>Ggo_PSG5N (Gorilla gorilla; gorilla) WGS CABD030113743.1; XM_019014755.1

CATCACTTTTAAACTTCTGGAACCTGCCTATCAGTGCCCAAGTTACGATTGAAGCCCAGCCACCGAAAGTGTCCGAGGGGAAGGATGTTCTTCTACTTGTCCACAATTTGCCCCAGAATCTTGCTGGCTACATCTGGTACAAAGGACAACTGATGGACCTCTACCATTACATTACATCATATGTAGTAGACGGTCAAATAAATATATATGGGCCGGCATACAGTGGACGAGAAACAGTATATTCCAATGCATCCCTGCTAATCCAGAATGTCACCCAGGAGGATGCAGGATCCTACACCTTACACATCATAAAGCGAGGTGATAGGACTAGAGGAGTAACTGGACATTTCACCTTCAACTTATACC

>Ggo_PSG7N (Gorilla gorilla; gorilla) WGS SRLZ01001140.1

CATCACTTTTAAACTTCTGGAACACGCCCACCACAGCCCAAGTCACGATTGAAGCCCAGCCACCAAAAGTTTCCGAGGGGAAGGATGTTCTTCTACTTGTCCACAATTTGCCCCAGAATCTTACTGGCTACATCTGGTACAAAGGGCAAATCAGGGACCTCTACCATTACGTTACATCATATGTAGTAGACGGTCAAATAATTATATATGGGCCTGCATATAGTGGACGAGAAACAGTATATTCCAATGCATCCCTGCTGATCCAGAATGTCACCTGGGAAGACGCAGGATCCTACACCTTACACATCATAAAGCGAGGTGATGGGACTGGAGGAGAAACTGGAAATTTCACCTTCACCTTATACC

>Ggo_PSG11N (Gorilla gorilla; gorilla) WGS SRLZ01001140.1

CATTACTTTTAAACTTCTGGAACCTGCCTACCACTGCCCAAGTCACGATTGAAGCCCAGCCTCCCAAAGTGTCCGAGGGAAAGGATGTTCTTCTACTTGTCCACAATTTGCCCCAGAATCTTACTGGCTACATCTGGTACAAAGGGCAAATAAGGGACCTCTACCATTACATTACATCATACGTAGTAGACGGTCAAATAATTATATATGGGCCGGCATACAGTGGACGAGAAACAGTATATTCCAATGCATCCCTGCTGATCCAGAATGTCACCCGGGAGGACGCAGGATCCTACACTTTACACATCATAAAGCGAGGTGATGGGACTGGAGGAGTAACTGGAAATTTCACCTTCACCTTATACT

>Ggo_PSGP1N (Gorilla gorilla; gorilla) no ORF; WGS CYUI03002619.1

CATCACTTTTAAACTTCTGGAACCGCCTACCACTGCCCAAGTCACGATTGAAGCCCAGCCACCCAAAGTTTCCGAGGGGAAGGATGTTCTTCTACTTGTCCACAATTTGCCCCAGAATCTTACTGGCTACATCTGGTACAAAGGGCAAATAAGGGACCTCTACCATTACATTACATCATATGTAGTAGACGGTCAAATAATTATATATGGGCCGGCATACAGTGGACGAGAAACAGTATATTCCAATGCATCCCTGCTGATCCAGAATGTCACCCGGGAGACGCAGGATCCTACACTTTACACATCATAAAGCGAGGTGATGGGACTGGAGGAGTAACTGGAAATTTCACCTTCACCTTATACc

>Ggo_PSGP2N (Gorilla gorilla; gorilla) WGS CYUI03001188.1

CATCACTTTTAAACTTCTGGAACCTGCCTACCACTGCCCAAGTCACGATTGAAGCCCAGCCACCAAAGTTTCCGAGGGGAAGGATGTTCTTCTACTTGTCCACAATTTGCCCCAGAATCTTACTGGCTACATCTGGTACAAAGGGCAAATAAGGACCTCTACCATTACATTACATCATACGTAGTAGACGGTCAAATAATTATATATGGGCCGGCATACAGTGGACGAGAAACAGTATATTCCAATGCATCCCTGCTGATCCAGAATGTCACCCGGGAGGATGCAGGATCCTACACTTACACATCATAAAGCGAGGTGATGGGACTGGAGGAGTAACTGGACATTTCACCTTCACCTTATACc

>Hmo_PSG1N (Hylobates moloch; silvery gibbon) WGS WKKJ01000015.1; XM_032172783.1

CATCACTTTTACACTTCTGGAACCCGCCCACCACTGCCCAAGTAACGATTGAAGCCCAGCCACCCAAAGTTTCCGAGGGAAAGGACGTTCTTCTACTTGTCCACAATTTGCCCCAGAATCTTGCTGCCTACATCTGGTACGAAGGGCAAATGATGGACATCCACCATTACATTACATCATATGTAGTAGATGATCAAACAATTGTATATGGGCCTGCATACAGTGGACGAGAAACAGTATATTCCAATGCATCCTTGCTGATCCAGAATGTCACGGGGGAGGATGCAGGATCCTACACCTTACAAATCATAAAGCGAGGTGATGGGATTAGAGGAGCAACTGGACATTTCACCTTCACCTTATACC

>Hmo_PSG2N (Hylobates moloch; silvery gibbon) WGS WKKJ01000015.1; XM_032172788.1

CATCACTTTTAAACTTCTGGAACGTGCCCACCACTGCCCAAGTCACTATTGAAGCCCAGCCACCCAAACTTTCCGAGGGGAAGGACGTTCTTCTACTTGTCCACAATTTGCCCCAGAATCTTACTGGCTACACCTGGTACAAAGGGCAAATGACGGACCTCTACCATTACATTACATCATATGTAGTAGACAATGACATAATTATATCTGGGCCTGCATACACTGGACGAGAAACAGTATATTCCAACGCATCCCTGCTGATCCAGAATGTCACGTGGGAGGACACAGGACCCTACACCTTACACATCATAAAGCAAGGTGATGAGACCAGAGGAGCAACTGGACATTTCACCATCACCTTATACC

>Hmo_PSG3N (Hylobates moloch; silvery gibbon) WGS WKKJ01000015.1; XM_032172786.1

CATCACTTTTACACATCTGGAATGCACCCACCACTGCCCAAGTCACGATTGAAGCCCATCCACCCAAACTTTCTGAGGGGAAGGATGTTCTTCTACTTGTCCACAATTTGCCCAAGAATCTTGCTGGCTACATCTGGTACAAAGGGCAAATGACGGACCTCCAGCATTACATTACATCATATCTAGTACACAATCATAAAATTATACCTGGGCCTGAATACACTGGACGAGAAACAGTATATTCCAATGCATCCCTGCTGATCGAGGATGTCACACGGGAGGACGCAGGATCCTACACCTTACAAATCATAAAGCCAGGTGATGGGATTAGAGGAGCAACTGGACATTTCACCGTCACCTTATACC

>Hmo_PSG4N (Hylobates moloch; silvery gibbon) WGS WKKJ01000380.1; XM_032147535.1

CATCACTTTTAAACTTCTGGAACTCGCCCGCCACTGCCCAAGTCACTATTGAAGCCCAGCCACCAAAAATTTCCGAGGGGAAGGATGTTCTTCTACATGTCCACAATTTGCCCCAGAATCTTACTGGCTACATGTGGTACAGAGGGCAAATGACAGACCTCTACCATTACATTGTATCATATGTAGTAGACAATGACATAATTATATCTGGGCCTGCATACACTGAACGAGAAACAGTATATTCCAATGCATCCCTGCTGATCCAGAATGTCACCCGGGAGGACGCAGGATCCTACACCTTACACATCATAAAGCGAGGTGATGACACTACAGGAATAACTGGACATTTCACCGCCACCTTATACC

>Hmo_PSG6N (Hylobates moloch; silvery gibbon) WGS WKKJ01014248.1

CATCACTGTTAAACTTCTGGAACCCGCCCACCACTGCCCAAGTCACTATTGAAGCCCAGCCACCCAAACTTTCCGAGGGGAAGGACGTTCTTCTACTTGTCCACAATTTGCCCCAGAATCTTACTGGCTACACCTGGTACAAAGGGCAAATGATGGACCTCTACCATTACATTACATCATATGTAGTAGACAATGACATAATTATATCTGGGCCTGCATACACTGGACGAGAAACAGTATATTCCAACGCATCCCTGCTGATCCAGAATGTCACGCGGGAGGACACAGGACCCTACACCTTACACATCATAAAGCGAGGTGATGAGACTAGAGGAGCAACTGGAAATTTCACCGTCACCTTATACC

>Hmo_PSG7N (Hylobates moloch; silvery gibbon) WGS WKKJ01012486.1

CATCACTTTTAAACTTCTGGAACCCGCCCACCACTGCCCAAGTCATGATTGAAGCCCAGCCACCCAAAATTTCCGAGGGGAAGGATGTTCTTCTACTTGTCCACAATTTGCCCCAGAATCTTGCTGGCTACATGTGGTACAAAGGGCAAATGACGGACCTCTACCATTACATTATATCATATATAGCAGACAGTCAAACAATTATACCTGGGCCTGCATACAGTGGCCGAGAAACAGTATATTCCAATGCATCCCTGCTCATCCAGAAAGTCACCCGGGAGGACGCAGGATCCTACAACTTACACATCATAAAGCGAGGTGATGAGACTACAGGAATAACTGGACATTTCACCGTCACCTTATACT

>Hmo_PSG8N (Hylobates moloch; silvery gibbon) WGS WKKJ01004921.1

CATCACTTTTAAACTTCTGGAACCCGCCCACCACTGCCCAAGTTATGATTGAAGCCCAGCCACCCAAAATTTCCGAGGGGAAGGATGTTCTTCTACTTGTCCACAATTTGCCCCAGAATCTTCCTGGCTACATGTGGTACAAAGGGAAAATGAAGGACCTCTACCATTACATTGTATCATATGTAGCAGACAGTCAAATAATTATACCTGGGCCTGCATACAGTGGACGAGAAATAGTATATTCAAATGCATCCCTGCTGATCAAGAATGTCACCCGGGAGGACGCAGGATCCTACAACTTACACATCATAAAGCGAGGTGATGAGACTACAGGAGTAACTGGACATTTCACCGTCACCTTATACC

>Hmo_PSGP1N (Hylobates moloch; silvery gibbon) WGS WKKJ01001351.1

CATCACTTTTACACTTCTGGAATGCACCCACCACGGCCCAAGTCACGATTGAAGCCCAGCCACCCAAAGTTTCTGAGGGGAAGGATGTTCTTCTACTTGTCCACAATTTGCCCCAGAATCTTGATGCCTATGTCTGGTACAAAGGGCAAATGACTGACTTCCACCATTACATTGCATCATATGTAGCAGACAGTGAAATAATTGTGTCCGCACCTGCATACCGTGGACGAGTAACACTATATTCCAATGCATCCCTGCTGATCCAGAATGTCACCTAGGAGGACGCAGGATCCTACACATTTCACATCATAATGCGAGGTGATGAGACTAGAGGACTAAGTGGACATTTCACCTTCACCTTATACC

>Hsa_PSG1N (Homo sapiens; man)

CATCACTTTTAAACTTCTGGAACCTGCCCACCACTGCCCAAGTCACGATTGAAGCCGAGCCAACCAAAGTTTCCGAGGGGAAGGATGTTCTTCTACTTGTCCACAATTTGCCCCAGAATCTTACCGGCTACATCTGGTACAAAGGGCAAATGAGGGACCTCTACCATTACATTACATCATATGTAGTAGACGGTGAAATAATTATATATGGGCCTGCATATAGTGGACGAGAAACAGCATATTCCAATGCATCCCTGCTGATCCAGAATGTCACCCGGGAGGACGCAGGATCCTACACCTTACACATCATAAAGGGAGATGATGGGACTAGAGGAGTAACTGGACGTTTCACCTTCACCTTACACC

>Hsa_PSG2N (Homo sapiens; man)

CATCACTTTTAAACTTCTGGAACCTGCCCACCACTGCCCAAGTCACGATTGAAGCCCAGCCACCAAAAGTTTCCGAGGGGAAGGATGTTCTTCTACTTGTCCACAATTTGCCCCAGAATCTTACTGGCTACATCTGGTACAAAGGGCAAATCAGGGACCTCTACCATTACATTACATCATATGTAGTAGACGGTCAAATAATTATATATGGGCCTGCATATAGTGGACGAGAAACAGCATATTCCAATGCATCCCTGCTGATCCAGAATGTCACCCGGGAGGACGCAGGATCCTACACCTTACACATCATAAAGCGAGGTGATGGGACTAGAGGAGTAACTGGATATTTCACCTTCACCTTATACC

>Hsa_PSG3N (Homo sapiens; man)

CATTACTTTTAAACTTCTGGAACTTGCCTACCACTGCCCAAGTCACGATTGAAGCCGAGCCAACCAAAGTTTCCAAGGGGAAGGACGTTCTTCTACTTGTCCACAATTTGCCCCAGAATCTTGCTGGCTACATCTGGTACAAAGGGCAAATGAAGGACCTCTACCATTACATTACATCATACGTAGTAGATGGTCAAATAATTATATATGGGCCTGCATACAGTGGACGAGAAACAGTATATTCCAATGCATCCCTGCTGATCCAGAATGTCACCCGGGAGGACGCAGGATCCTACACCTTACACATCGTAAAGCGAGGTGATGGGACTAGAGGAGAAACTGGACATTTCACCTTCACCTTATACC

>Hsa_PSG4N (Homo sapiens; man)

CATCACTTTTAAACTTCTGGAATCCGCCCACAACTGCCCAAGTCACGATTGAAGCCCAGCCACCCAAAGTTTCTGAGGGGAAGGATGTTCTTCTACTTGTCCACAATTTGCCCCAGAATCTTGCTGGCTACATTTGGTACAAAGGGCAAATGACATACCTCTACCATTACATTACATCATATGTAGTAGACGGTCAAAGAATTATATATGGGCCTGCATACAGTGGAAGAGAAAGAGTATATTCCAATGCATCCCTGCTGATCCAGAATGTCACGCAGGAGGATGCAGGATCCTACACCTTACACATCATAAAGCGACGCGATGGGACTGGAGGAGTAACTGGACATTTCACCTTCACCTTACACC

>Hsa_PSG5N (Homo sapiens; man)

CATCACTTTTAAACTTCTGGAACCTGCCTATCACTGCTCAAGTCACGATTGAAGCCCTGCCACCCAAAGTTTCCGAGGGGAAGGATGTTCTTCTACTTGTCCACAATTTGCCTCAGAATCTTGCTGGCTACATCTGGTACAAAGGACAACTGATGGACCTCTACCATTACATTACATCATATGTAGTAGACGGTCAAATAAATATATATGGGCCTGCATACACTGGACGAGAAACAGTATATTCCAATGCATCCCTGCTGATCCAGAATGTCACCCGGGAAGACGCAGGATCCTACACCTTACACATCATAAAGCGAGGTGATAGGACTAGAGGAGTAACTGGATATTTCACCTTCAACTTATACC

>Hsa_PSG6N (Homo sapiens; man)

CATCACTTTTAAACTTCTGGAACCTGCCCACCACTGCCCAAGTAATAATTGAAGCCAAGCCACCCAAAGTTTCCGAGGGGAAGGATGTTCTTCTACTTGTCCACAATTTGCCCCAGAATCTTACTGGCTACATCTGGTACAAAGGGCAAATGACGGACCTCTACCATTACATTACATCATATGTAGTACACGGTCAAATTATATATGGGCCTGCCTACAGTGGACGAGAAACAGTATATTCCAATGCATCCCTGCTGATCCAGAATGTCACACAGGAGGATGCAGGATCCTACACCTTACACATCATAAAGCGAGGCGATGGGACTGGAGGAGTAACTGGATATTTCACTGTCACCTTATACT

>Hsa_PSG7N (Homo sapiens; man)

CATCACTTTTAAACTTCTGGAACCCGCCCACCACAGCCCAAGTCACGATTGAAGCCCAGCCACCAAAAGTTTCCGAGGGGAAGGATGTTCTTCTACTTGTCCACAATTTGCCCCAGAATCTTACTGGCTACATCTGGTACAAAGGACAAATCAGGGACCTCTACCATTATGTTACATCATATATAGTAGACGGTCAAATAATTAAATATGGGCCTGCATACAGTGGACGAGAAACAGTATATTCCAATGCATCCCTGCTGATCCAGAATGTCACCCAGGAAGACACAGGATCCTACACTTTACACATCATAAAGCGAGGTGATGGGACTGGAGGAGTAACTGGACGTTTCACCTTCACCTTATACC

>Hsa_PSG8N (Homo sapiens; man)

CATCACTTTTAAACTTCTGGAACCCACCCACGACTGCCCAAGTCACGATTGAAGCCCAGCCAACCAAAGTTTCTGAGGGGAAGGATGTTCTTCTACTTGTCCACAATTTGCCCCAGAATCTTACTGGCTACATCTGGTACAAAGGGCAAATCAGGGACCTCTACCATTACATTACATCATATGTAGTAGACGGTCAAATAATTATATATGGGCCTGCATACAGTGGACGAGAAACAATATATTCCAATGCATCCCTGCTGATCCAGAATGTCACCCAGGAAGACGCAGGATCCTACACCTTACACATCATAATGGGAGGTGATGAGAATAGAGGAGTAACTGGACATTTCACCTTCACCTTATATC

>Hsa_PSG9N (Homo sapiens; man)

CATCACTTTTAAACTTCTGGAACCCGCCCACCACTGCCGAAGTCACGATTGAAGCCCAGCCACCCAAAGTTTCTGAGGGGAAGGATGTTCTTCTACTTGTCCACAATTTGCCCCAGAATCTTCCTGGCTACTTCTGGTACAAAGGGGAAATGACGGACCTCTACCATTACATTATATCGTATATAGTTGATGGTAAAATAATTATATATGGGCCTGCATACAGTGGAAGAGAAACAGTATATTCCAACGCATCCCTGCTGATCCAGAATGTCACCCGGAAGGATGCAGGAACCTACACCTTACACATCATAAAGCGAGGTGATGAGACTAGAGAAGAAATTCGACATTTCACCTTCACCTTATACT

>Hsa_PSG10N (Homo sapiens; man) L14729.1

CATCACTTTTAAACTTCTGGAACCCACCTACCATTGCCCAAGTCACGACAGAAGCCCAGCCACCCAAAGTTTCCGAGGGGAAGGATGTTCTTCTACTTGTCCACAATTTGCCCCAGAATCTTACTGGTTACATGTGGTACAAAGGGCAAATAAGGGACCTCTACCATTACATTACATCATATGTAGTAGACGGTCAAATAATTACATATGGGCCTGCATACAGTGGACGAGAAACAGTATATCCCAATGCATCCCTGCTGATCCAGAATGTCACCCGGGAGGACGGAGTATCCTACACCTTACACATCATACAGCGAGGTGATGGGACTAGAGGAGTAACTGGAAATTTCACCTTCACCTTATACC

>Hsa_PSG11N (Homo sapiens; man)

CATTACTTTTAAACTTCTGGAACTTGCCTACCACTGCCCAAGTCATGATTGAAGCCCAGCCACCCAAAGTGTCCGAGGGGAAGGATGTTCTTCTACTTGTCCACAATTTGCCCCAGAATCTTACTGGCTACATCTGGTACAAAGGGCAAATCAGGGACCTCTACCATTACATTACATCATATGTAGTAGACGGTCAAATAATTATATATGGACCGGCATACAGTGGACGAGAAACAGTATATTCCAATGCATCCCTGCTGATCCAGAATGTCACCCGGGAGGACGCAGGATCCTACACCTTACACATCATAAAGCGAGGTGATGGGACTAGAGGAGTAACTGGATATTTCACCTTCACCTTATACC

>Mfa_PSG1N (Macaca fascicularis; crab-eating macaque) WGS AQIA01035928.1; XM_005590662.2 CATCACTTTTAATCTTCTGGAACACGCCCACCACTGCCCAAGTCACGATTGAAGCCCAGCCAACCAAAGTTTCTGAGGGGAAGGATGTTCTGCTACTTGTACACAATTTGCCCCAGAATCTTGCTGCCTACATCTGGTACAAAGGGCAAATAATGGACCTCCACCATTACATTACATCATATGTAATAGACACTGAAATAATTGTATTTGGGCCTGCATACAGTGGACGAGAAACAGTATATTCCAATGCATCCCTGCTGATCCAGAATGTCACCCAGAAGGACACAGGATCCTACACCATACAAATCATAAAGCGAGGTGATACCACTAAAGGAGTAACTGGACATTTCACCTTATACC

>Mfa_PSG2N (Macaca fascicularis; crab-eating macaque) WGS CAEC01261139.1; XM_005589363.2

CATCACTTTTAATCTTCTGGAACCCGCCCACCACTGCTCAAGTCACGATTGAAGCCCAGCCAGCCAAAGTTTCTGAGGGGAAGGATGTTCTTCTACTTGTCCACAATTTGCCCCAGAATCTTGCTGCCTGCATCTGGTACAAAGGGCAAATAATGGACCTCCAACATTACATTACAGCATATGTAATAGATGCTGAAACAATTATATTTGGGCCTGCATACAGTGGACGAGAAACAGTATATTCCAATGCATCCCTGCTGATCCAGAATGTCACCCAGAAGGACACAGGATCCTACACCATACAAATCATACAGCGAGGTGATAAGACTAAAGGAGTAACTGGACATTTCACCTTATACC

>Mfa_PSG3N (Macaca fascicularis; crab-eating macaque) XM_015444565.1

CATCACTTTTAATCTTCTGGAACCCGCCCACCACTGCCCAAGTCACGATCGAAGCACAGCCAGCCAAAGTTTCTGAGGGGAAGGATGTTCTGCTACTTGTCCACAATTTGCCCCAGAATGTTGCTGGCTACAGCTGGTACAAAGGGCAAATAATGGACCTCCACCATTACATTACATCATATGTAATAGACACTGAAATCATTATATTTGGGCCTGCATACAGTGGACGAGAGACAGTATATTCCAATGCATCCCTGCTGATCCAGAATGTCACCCAGAAGGACACAGGATCCTACACCATTCAAATCACAAAGCGAGGTGATGGGACTAAAAGAGTAACTGGACATTTCACTTTATACC

>Mfa_PSG4N (Macaca fascicularis; crab-eating macaque) XM_015444564.1

CATCACTTTTAATCTTCTGGAACCCGCCCACCACTGCTCAAGTCACGATTGAAGCCCAGCCAGCCAAAGTTTCCGAGGGGAAGGATGTTCTTCTACTTGTCCACAATTTGCCCCAGAATCTTACTGGCTACATCTGGTACAAAGGGCAAATAATGGACCACCACCATTACATTACATCATATGTGATAGACACTGAAACAATTATATTTGGGCCTGCATACAATGAACGAGAAACAGTATATTCCAATGCATCCCTGCTGATCCAGAATGTCACCAAGAATGACACAGGATCCTACACCATACAAATCATAAAGCGAGGTGATAGGACTGAAGGAGTAACTGGACATTTCACCTTATACC

>Mfa_PSG5N (Macaca fascicularis; crab-eating macaque) XM_015444564.1

CATCACTTTTAATCTTCTGGAACCCGCCCACCACTGCCCAAGTCATGATTGAAGCCCAGCCAACCAAAGTTTCTGAGGGGAAGGATGTTCTGCTACTTGTCTACAATTTGCCCCAGAATCTTACTGGCTACAGCTGGTACAAAGGGCAAATAATGGACCTCCAGCATTACATTGCAGCATATACAATAGACACTGAAATGATTGTATTTGGGCCTGCATACAGTGGACGAGAAACAGTATATTCCAATGCATCCCTGCTGATCCAGAATGTCACCAAGAATGACACAGGATCCTACACCATTCAAATAACAAAGCAAGGTGATGAGACTAAAGGAGTAACTGGACATTTCACCTTATACC

>Mfa_PSG6N (Macaca fascicularis; crab-eating macaque) XR_001487256.1

CATCACTTTTAATCTTCTGGAACCCGCCCACCACTGCGCAAGTCACGATTGAAGCTCAGCCAACCAAAGTTTCTGAGGGGAAGGATATTCTTCTACTTGTCCACAATGTGCCCCAGAATGTTGCTGGCTACATCTGGTACAAAGGGCAAATAATGGACCTCCAGCATTACATTACATCATATGTAATAGACACTGAAATCATTATATTTGGGCCTGCATACAGTGGACGAGAAAGAGTATATTCCAATGCATCCCTGCTGATCCAGAATGTCTCCCGGAAGGACACAGGATCCTACACCATTCAAATCATAAAGCGAGGTGATAAGATTAAAGGGGTAACTGGACATTTCACCTTATACG

>Mfa_PSG7N (Macaca fascicularis; crab-eating macaque) XM_015441029.1

CATCACTTTTAATCTTCTGGAACCCGCCCACCACTGCCCAAGTCACGATTGAAGCCCAGCCAGCCAAAGTTTCTGAGGGGAAGGATGTTCTTCTACTTGTCCACAATTTGCCCCAGAATCTTACTGGCTACGTCTGGTACAAAGGGCAAATAATGGACCTCCACCAATTCATTACAGCGTATACAATAGACACTGACACAATTATATTTGGGCCTGCATACAGTGGACGAGAAACAGTATATTCCAATGCATCCCTGCTGATCCAGAATGTCACCCGGAAGGACACAGGATCCTACACCATACAAATTATAAAGCGAGGTGATAAGATTAAAAGAATAACTGGACATTTCACCTTATACC

>Mfa_PSG8N (Macaca fascicularis; crab-eating macaque) WGS CAEC01261404.1; XM_015441027.1

CATCACTTTTAATCTTCTGGAACCCGCCCACCACTGCCCAAGTCACGATTGAAGCACAGCCAGCCAAAGTTTCTGAGGGGAAGGATGTTCTTCTACTTGTCCAGAATTTGCCCCAGAATCTTACTGGCTACAGCTGGTACAAAGGGCAAATAATAGACCTCCAGCATTACATTACAGCATATACAATAGACACTGAAATGATTGTATTTGGGCCTGCATACAGTGGACGAGAAACAGTATATTCCAATGCATCCCTGCTGATCCAGAATGTCACCAAGAATGACACAGGATCCTACACCATTCAAATCACAAAGCGAGGTGATGAGACTAAAGGATTAACTGGACATTTCACCTTATACC

>Mfa_PSG9N (Macaca fascicularis; crab-eating macaque) XM_015444691.1

CATCACTTTTAATCTTCTGGAACCCGCCCACCACTGCTCAAGTCACGATTGAAGCCCAGCCAGCCAAAGTTTCTGAGGGGAAGGATGTTCTTCTACTTGTCCACAATTTGCCCCAGAATCTTACTGGCTACATCTGGTACAAAGGGCAAATAATGGATCTCCAGCATTACATTACAGCATATGCAATAGACACTGAAACAATTATATTTGGGCCTGTATACAGTGGACGAGAAACAATATATTCCAATGCATCCCTGCTGATCCAGAGTGTCACCAAAAATGACACAGGATCCTACACCATACAAATCATAAAGCGAGGTGATAGGACTGAAGGAGTAACTGGACATTATACCTTATACC

>Mfa_PSG10N (Macaca fascicularis; crab-eating macaque) XM_015441025.1

CATCACTTTTAATCTTCTGGAACCCGCCCACCACTGCTCAAGTCACAATTGAAGCCCAGCCAGCCAAAGTTTCTGAGGGGAAGGATGTTCTTCTACTTGTCCACAATTTGCCCCAGAATCTTACTGGCTACATCTGGTACAAAGGGCAAAAAACGGACCTCCACCTTTACGTTACATCATATGTAAAAGATACTGAAACAGTTATAGCTGGGCCTGCATACAGTGGACGAGAAACAGTATATTCCAATGCATCCCTGCTGATCCAGAATGTCACCCAGAAGGACACAGGATCCTACACCATACAAATCACAAAGCGAGGTGACAGGACTGAAGGAGAAACTGCACATTTCACCTTATATC

>Mfa_PSG11N (Macaca fascicularis; crab-eating macaque) WGS AQIA01035943.1; XM_015441026.1

CATCACTTTTAATCTTCTGGAACCCGCCCACCACTGCGCAAGTCACGATTGAAGCTCAGCCAACCAAAGTTTCTGAGGGGAAGGATATTCTTCTACTTGTCCACAATGTGCCCCAGAATGTTGCTGGCTACATCTGGTACAAAGGGCAAATAATGGACCTCCAGCATTACATTACAGCATATGCAATAGACACTGAAACAATTATATTTGGGCCTGTATACAGTGGACGAGAAACAATATATTCCAATGCATCCCTGCTGATCCAGAGTGTCACCAAAAATGACACAGGATCCTACACCGTAGAAATCGTAAAGCGAGGTGAAGGGACTGAAGGAGTAACTGGACATTTCACCTTATACC

>Mfa_PSG12N (Macaca fascicularis; crab-eating macaque) XM_015442094.1

TATTACTTTTCATCTTCTGGAACCCGCCCACCACTGCCCAAGTCACGATTGAAGCCCAGCCAGCCAAAGTTTCCGAGGGGAAGGATGTTCTTCTACTTGTCCAGAATTTGCCTGAGAATCTTACTGGCTACGTCTGGTTCAAAGGGCAAATAATGGACTTCCACCAATTCATTACAGCGTATACAATAGACACTGAAACAATTATATTTGGGCCTGCATACAGTGGACGAGAAACAGTATATTCCAATGCATCCCTGCTGATCCAGAATGTCACCCAGAATGACACAGGATCCTACACCATAGAAATTATAAAGCGAGGTGATAAGATTAAAGGAGTAACTGGACATTTCACCTTATACG

>Mfa_PSG13N (Macaca fascicularis; crab-eating macaque) XM_015441028.1

TATTACTTTTCATCTTCTGGAACCCGCCCACCACTGCCCAAGTCACGATTGAAGCCCAGCCAGCCAAAGTTTCCGAGGGGAAGGATGTTCTTCTACTTGTCCAGAATTTGCCCCAGAATCTTACTGGCTACGTCTGGTTCAAAGGGCAAATAACGACCTTCCACCAATTCATTATAGCATATAAAATAGACAGTAGAAAAATTACAGTTGGGCCTGTATACAGTGGACGAGAAAGAGTATATTCCAATGCATCCCTGCTGATCCAGAATGTCTCCCGGAAGGACACAGGATCCTACACCATTCAAATCATAAAGCGAGGTGATAAGACTAAAGGAGTAACTGGACATTTCACCTTATACG

>Mfa_PSG14N (Macaca fascicularis; crab-eating macaque) WGS AEHL01216501.1

CATCACTTTTAATCTTCTGGAACACGCCCACCACTGCCCAAGTCACGATTGAAGCCCAGCCAACCAAAGTTTCTGAGGGGAAGGATGTTCTTCTACTTGTCCACAATTTGCCCACGAACGTTGTTGGCTACATCTGGTACAAAGGGCAAATAATGGACCTCCACCATTACATTACATCATATGTAATAGACACTGAAATAATTGTATTTGGGCCTGCATACAGTGGACGAGAAACAGTATATTCCAATGCATCCCTGCTGATCCAGAATGTCACCCAGAAGGACACAGGATCCTACACCATACAAATCATAAAGCGAGGTGATATCACTAAAGGAGTAACTGGACATTTAACCTTATACC

>Mfa_PSG15N (Macaca fascicularis; crab-eating macaque) WGS AEHL01216505.1 TATCACTTTTAATCATCTGGAACCCACCTACCACGGGTCAAGTCACGATTGAAGCTCAGCCAACCGAGGTTTCTGAGGGGAAGGATGTTCTTCTACTTGTCCACAATTTGCCCCAGAATCCTACTGGCTACAGCTGGTACAAAGGGCAAATAACAGACATCCACCATTACATTACATCATATGTAATAGACACTGAAATGATTGTATTTGGGCCTGCATACAGTGGACGAGAAACAGTATATTCCAATGCATCCCTGCTGATCCAGAATGTCACCCAGAAGGACACAGGATCCTACACCATACAAATCATACAGCGAGGTGATACCACTAAAGGAGTAACTGGACATTTCACCTTATACC

>Mfa_PSG16N (Macaca fascicularis; crab-eating macaque) WGS AQIA01035938.1

CATCACTTTTAATCTTCTGGAACCCTCCCACCACTGCCCAAGTCACGATTGAAGCCCAGCCAACCAAACTTTCTGAGGGGAAGGATGTTCTTCTACTTGTCCACAATTTGCCCCAGAATCTTACTGGCTACAGCTGGTACAAAGGGCAAATAATAGACCTCCAGCATTACATTACAGCATATACAATAGACACTGAAATGATTGTACTTGGGCCTGCATACAGTGGACGAGAAACAGTATATTCCAATGCATCCCTGCTGATCCAGAATGTCACCAAGAATGACACAGGATCCTACACCATTCAAATCACAAAGCGAGGTGATGAGACTAAAGGAGTAACTGGACATTTCACCTTATACC

>Mfa_PSG17N (Macaca fascicularis; crab-eating macaque) WGS AQIA01035923.1

CATCACTTTTAATCTTCTGGAACCCGCCCACCACTGCCCAAGTCACGATTGAAGCCCAGCCAACCAAAGTTTCCGAGGGGAAGGATGTTCTTCTACTTGTCCAGAATTTGCCCCAGAATCTTATTGCCTACATCTGGTACAAAGGGCAAAAAACGGACTTCCACCATTACATTACATCATATGTAATAGATGCTGAAACAATTATAGTTGGGCCTGCATACAGTGGACGAGAAACAGTATATTCCAATGCATCCCTGCTGATCCAGAATGTCACCCAGAATGACACAGGATCCTACACCATTCAAATGATAAAACAAGGTGATAAGACTAAAGGAGTAATTGGACATTTCACCTTATACC

>Mfa_PSG18N (Macaca fascicularis; crab-eating macaque) WGS CAEC01261518.1

CATCACTTTTAATCTTCTGGAACYCGCCCACCACTGCYCAAGTCACGATTGAAGCCCAGCCAACCAAAGTTTCTGAGGGGAAGGATGTTCTTCTACTTGTCCACAATTTGCCCCAGAATCTTACTGGCTACRTCTGGTACAAAGGGCAAATAATGGACCTCCACCAATTCATTACAGCGTATACAATAGACACTGACACAATTATATTTGGGCCTGCATACAGTGGACGAGAAAGAGTATATTCCAATGCATCCCTGCTGATCCAGAATGTCWCCCGGAAGGACACAGGATCCTACACCATACAAATTATAAAGCGAGGTGATAAGATTAAAAGAATAACTGGACATTTCACCTTATACC

>Mfa_PSG19N (Macaca fascicularis; crab-eating macaque) WGS AQIA01076865.1

CATCACTTTTAATCTTCTGGAACTCGCCCACCACTGCCCAAGTCACGATTGAAGCTCAGCCAACCAAAGTTTCTGAGGGGAAGGATGTTCTTCTACTTGTCCACAATTTGCCCACGAACGTTGTTGGCTACATCTGGTACAAAGGGCAAATAATGGACCTCCAGCATTACATTACAGCATATACAATAGACACTGAAATGATTATATTTGGGCCTGCATACAGTGGACGGGAAACAGTATATTCCAATGCATCCCTGCTGATCCAGAGTGTCACCAAGAATGACACAGGATCCTACACCATACAAATCATAAAGCGAGGTCATAGGACTGAAGGAGTAACTGGACATTTCACCTTATACC

>Mfa_PSG20N (Macaca fascicularis; crab-eating macaque) WGS CAEC01261372.1

CATCACTTTTAATCTTCTGGAACTCGCCCACCACTGCCCAAGTCACGATTGAAGCYCAGCCAACCAAAGTTTCTGAGGGGAAGGATGTTCTTCTACTTGTCCACAATTTGCCCACGAACGTTGTTGGCTACATCTGGTACAAAGGGCAAATAATGGACCTCCAGCATTACATTACAGCATATACAACAGACACTGAAATGATTCTATTTGGGCCTGCATACAGTGGACGAGAAACAGTATATTCCAATGCATCCCTGCTGATCCAGAGTGTCACCAARAATGACACAGGATCCTACACCATACAAATCATAAAGCGAGGTCATAGGACTGAAGGAGTAACTGGACATTACACCTTATACC

>Mfa_PSG21N (Macaca fascicularis; crab-eating macaque) WGS AQIA01074953.1

CATCACTTTTAATCTTCTGGAACCCGCCCACCACTGCTCAAGTCACGATTGAAGCCCAGCCAGCCAAAGTTTCCGAGGGGAAGGATGTTCTTCTACTTGTCCACAATTTGCCCCAGAATGTTACTGGCTACATCTGGTACAAAGGGCAAAAAACGGACCACCACCTTTACATTACATCATATGTAATAGACGCTGAAACAATTATACTTGGGCCTGCATACAGTGGACGAGAAACAGTATATTCCAATGCATCCCTGCTGATCCAGAATGTCACCTGGAAGGACACAGGATCCTACACCATAGAAATCATAAAGCGAGGTGATAGGACTGAAGGAGTAACTGGACATTATACCTTATACC

>Mfa_PSG22N (Macaca fascicularis; crab-eating macaque) WGS AEHL01034633.1

CATCACTTTTAATCTTCTGGAACTCGCCCACCACTGCCCAAGTCACGATTGAAGCCCAGCCAACCAAAGTTTCTGAGGGGAAGGATGTTCTTCTACTTGTCCACAATTTGCCCACGAACGTTGTTGGCTACATCTGGTACAAAGGGCAAATAATGGACCTCCAGCATTACATTACAGCATATACAACAGACACTGAAATGATTCTATTTGGGCCTGCATACAGTGGACGAGAAACAGTATATTCCAATGCATCCCTGCTGATCCAAAATGTCACCTGGAAGGACACAGGATCCTATACCATACAAATCATAGAGCGAGGTGAAGGGACTGAAGGAGTAACTGGACATTTCACCTTATACC

>Mfa_PSGP1N (Macaca fascicularis; crab-eating macaque) WGS CAEC01261143.1 nCATCACTTTTAATCTTCTGGAACYCGCCCACCACTGCTCAAGTCACRATTGAAGCCCAGCCARCCAAAGTTTCTGAGGGGAAGGATGTTCTTCTACTTGTCCACAATTTGCCCCAGAATCTTACTGGCTACATCTGGTACAAAGGGCAAAAAACGGAYTCCACCTTTACGTTACATCATATGTAAAAGATACTGAAACAGTTATAGCTGGGCCTGCATACAGTGGACGAGAAACAGTATATTCCAATGCATCCCTGCTGATCCAGAATGTCACCCAGAAGGACACAGGATCCTACACCATACAAATCACAAAGCGAGGTGATAGGACTGAAGGAGAAACTGCACATTTCACCTTATATC

>Mfu_PSG1N (Macaca fuscata fuscata; Japanese macaque) WGS BFBW01061201.1

CATCACTTTTAATCTTCTGGAACCCACCCACCACTGCCCAAGTCATGATTGAAGCCCAGCCAACCAAAGTTTCTGAGGGGAAGGATGTTCTGCTACTTGTCTACAATTTGCCCCAGAATCTTACTGGCTACAGTTGGTACAAAGGGCAAATAATGGACCTCCAGCATTACATTGCAGCATATACAATAGACACTGAAATGATTGTATTTGGGCCTGCATACAGTGGACGAGAAACAGTATATTCCAATGCATCCCTGCTGATCCAGAATGTCACCAAGAATGACACAGGATCCTACACCATTCAAATAACAAAGCAAGGAGATGAGACTAAAGGAGTAACTGGACATTTCACCTTATACC

>Mfu_PSG2N (Macaca fuscata fuscata; Japanese macaque) WGS BFBW01061211.1

CATCACTTTTAATCTTCTGGAACCCGCCCACCACTGCCCAAGTCACGATCGAAGCACAGCCAACCAAAGTTTCTGAGGGGAAGGATGTTCTGCTACTTGTCCACAATTTGCCCCAGAATGTTGCTGGCTACAGCTGGTACAAAGGGCAAATAATGGACCTCCACCATTACATTACATCATATGTAATAGACACTGAAATCATTATATTTGGGCCTGCATACAGTGGACGAGAAACAGTGTATTCCAATGCATCCCTGCTGATCCAGAATGTCACCCAGAAGGACACAGGATCCTACACCATTCAAATCACAAAGCGAGGTGATGGGACTAAAAGAGTAACTGGACATTTCACTTTATACC

>Mfu_PSG3N (Macaca fuscata fuscata; Japanese macaque) WGS BFBW01058450.1

CATCACTTTTAATCTTCTGGAACACGCCCACCACTGCCCAAGTCACGATTGAAGCCCAGCCAACCAAAGTTTCTGAGGGGAAGGATGTTCTGCTACTTGTACACAATTTGCCCCAGAATCTTGCTGCCTACATCTGGTACAAAGGGCACATAATGGACCTCCACCATTACATTACATCATATGTAATAGACACTGAAATAATTGTATTTGGACCTGCATACAGTGGACGAGAAACAGTATATTCCAATGCATCCCTGCTGATCCAGAATGCCACCCAGAAGGACACAGGATCCTACACCATACAAATCATAAAGCGAGGTGATACCACTAAAGGAGTAACTGGACATTTCACCTTATACC

>Mfu_PSG4N (Macaca fuscata fuscata; Japanese macaque) WGS BFBW01061208.1

CATCACTTTTAATCTTCTGGAACCCGACCACCACTGCTCAAGTCACGATTGAAGCCCAGCCAACCAAAGTTTCCGAGGGGAAGGATGTTCTTCTACTTGTCCACAATTTGCCCCAGAATCTTACTGGCTACATCTGGTACAAAGGGCAAATAATGGACCACCACCATTACATTACATCATATGTGATAGACACTGAAACAATTATATTTGGGCCTGCATACAATGAACGAGAAACAGTATATTCCAATGCATCCCTGCTGATCCAGAATGTCACCAAGAATGACACAGGATCCTACACCATACAAATCATAAAGCGAGGTGATAGGACTGAAGGAGTAACTGGACATTACACCTTATACC

>Mfu_PSG5N (Macaca fuscata fuscata; Japanese macaque) WGS BFBW01064650.1

TATCACTTTTAATCATCTGGATCCCACCTACCACGGGTCAAGTCACGATTGAAGCTCAGCCAACCGAGGTTTCTGAGGGGAAGGATGTTCTTCTACTTGCCCACAATTTGCCCCAGAATCCTACTGGCTACAGCTGGTACAAAGGGCAAATAACAGACATCCACCATTACATTACATCATATGTAATAGACACTGAAATGATTGTATTTGGGCCTGCATACAGTGGACGAGAAACAGTATATTCCAATGCATCCCTGCTGATCCAGAATGTCACCCAGAAGGACACAGGATCCTACACCATACAAATCATACAGCGAGGTGATACCACTAAAGGAGTAACTGGACATTTCACCTTATACC

>Mfu_PSG6 (Macaca fuscata fuscata; Japanese macaque) WGS BFBW01061546.1

CATCACTTTTAATCTTCTGGAACCCACCCACCACTGCTCAAGTCACAATTGAAGCCCAGCCAGCCAAAGTTTCTGAGGGGAAGGATGTTCTTCTACTTGTCCAGAATTTGCCCCAGAATCTTATTGCCTACATCTGGTACAAAGGGCAAAAAACGGACTTCCACCATTACATTACATCATATGTAATAGATGCTGAAACAATTATAGTTGGGCCTGCATACAGTGGACGAGAAACAGTATATTCCAATGCATCCCTGCTGATCCAGAATGTCACCAAGAATGACACAGGATCCTACACCATTCAAATGATAAAGCAAGGTGATAAGACTAAAGGAGTAATTGGACATTTCACCTTATACC

>Mfu_PSG7N Macaca fuscata fuscata; Japanese macaque) WGS BFBW01089564.1

CATCACTTTTAATCTTCTGGAACCCGCCCACCACTGCCCAAGTCACGATTGAAGCCCAGCCAACCAAAGTTTCCGAGGGGAAGGATGTTCTTCTACTTGTCCAGAATTTGCCTGAGAATCTTACTGGCTACGTCTGGTTCAAAGGGCAAATAATGGACTTCCACCAATTCATTACAGTGTATACAATAGACACTGAAACAATTATATTTGGGCCTGCATACAGTGGACGAGAAACAGTATATTCCAATGCATCCCTGCTGATCCAGAATGTCACCCAGAATGACACAGGATCCTACACCATAGAAATTATAAAGAGAGGTGATAAGATTAAAGGAGTAACTGGACATTTCACCTTATACG

>Mfu_PSG8N (Macaca fuscata fuscata; Japanese macaque) WGS BFBW01059728.1

CATCACTTTTAATCTTCTGGAACCCGCCCACCACTGCGCAAGTCACGATTGAAGCTCAGCCAACCAAAGTTTCTGAGGGGAAGGATATTCTTCTACTTGTCCACAATGTGCCCCAGAATGTTGCTGGCTACATCTGGTACAAAGGGCAAATAATGGACCTCCAGCATTACATTACAGCATATGCAATAGACACTGAAACAATTATATTTGGGCCTGTATACAGTGGACGAGAAACAATGTATTCCAATGCATCCCTGCTGATCCAGAGTGTCAACCAGAAGGATGCAGGATCCTACACCGTAGAAATCGTAAAGCGAGGTGAAGGGACTGAAGGAGTAACTGGACATTTCACCTTATACC

>Mfu_PSG9N (Macaca fuscata fuscata; Japanese macaque) WGS BFBW01076667.1

CATCACTTTTAATCTTCTGGAACCCGCCCACCACTGCCCAAGTCACGATTGAAGCCCAGCCAACCAAAGTTTCCGAGGGGAAGGATGTTCTTCTACTTGTCCAGAATTTGCCTGAGAATCTTACTGACTACGTCTGGTTCAAAGGGCAAATAATGGACTTCCACCAATTCATTACAGTGTATACAATAGACACCGAAACAATTATATTTGGGCCTGCATACAGTGGACGAGAAACAGTATATTCCAATGCGTCCCTGCTGATCCAGAATGTCACCCAGAATGACACAGGATCCTACACCATAGAAATTATAAAGCGAGGTGATAAGATTAAAGGAGTAACTGGACATTTCACCTTATACG

>Mfu_PSG10N (Macaca fuscata fuscata; Japanese macaque) WGS BFBW01089563.1

TATTACTTTTCATCTTCTGGAACCCGCCCACCACTGCCCAAGTCACGATTGAAGCCCAGCCAGCCAAAGTTTCCGAGGGGAAGGATGTTCTTCTACTTGTCCAGAATTTGCCTGAGAATCTTACTGGCTACGTCTGGTTCAAAGGGCAAATAATGGACTTCCACCAATTCATTACAGCGTATACAATAGACACTGAAACAATTATATTTGGGCCTGCATACAGTGGACGAGAAACAGTATATTCCAATGCATCCCTGCTGATCCAGAATGTCACCCAGAATGACACAGGATCCTACACCATAGAAATTATAAAGAGAGGTGATAAGATTAAAGGAGTAACTGGACATTTCACCTTATACG

>Mfu_PSG11N (Macaca fuscata fuscata; Japanese macaque) WGS BFBW01060462.1

CATCACTTTTAATCTTCTGGAACCCGCCCACCACTGCTCAAGTCACGATTGAAGCCCAGCCAGCCAAAGTTTCCGAGGGGAAGGATGTTCTTCTACTTGTCCACAATTTGCCCCAGAATGTTACTGGCTACATCTGGTACAAAGGGCAAAAAACGGACCACCACCTTTACATTACATCATATGTAATAGACGCTGAAACAATTATACTTGGGCCTGCATACAGTGGACGAGAAACAGTATATTCCAATGCATCCCTGCTGATCCAGAATGTCACCTGGAAGGACACAGGATCCTACACCATAGAAATCATAAAGCGAGGTGATAGGACTGAAGGAGTAACTGGACATTATACCTTATACC

>Mfu_PSG12N (Macaca fuscata fuscata; Japanese macaque) WGS BFBW01062874.1

CATCACTTTTAATCTTCTGGAACTCGCCGACCACTGCCCAAGTCACAATTGAAGCTCAGCCAACCAATATTTCCGAGGGGAACGATGTTCTTCTACTTGTGCACAATTTACCCAAGAATCCTGCTGCCTACATCTGGTACAAAGGGCAAATAATGGACCTCCAAAATTACATTACAGCATATACAATAGACACTGAAAGAATTATATTTGGGCCTGCATACAGTGGACGAGAAAGAGTATATTCCAATGCATCCCTGCTGATCCAGAGTGTGAACCAGAAGGACGCAGGATCCTACACCGTAAAAATCATAAAGCGAGGTTACAGGACTGAAGGAGTAACTGGACATTTCACCTTATATG

>Mfu_PSG13N (Macaca fuscata fuscata; Japanese macaque) WGS BFBW01061213.1

CATCATTTTTAATCTTGTGGAACACGCCCACCACTGCCCAAGTCATGATTGAAGCTCAGCCTACCAAAGTTTCTGAGGGGAAGGATGTTCTGCTACTTGTCCGCAATTTGCCCCAGAAAGTTGCTGCCTTTGTCTGGTACAAAGGGCAAATAATGGACTTCCACCAATTCATTACAGCATATACAATAGACACTGAAAGAATTATATTTGGGTATGCATACAGTGGAAGAGAAACACTATATTCCAATGGATCCCTGCTGATCCGGAATGTCACCAAGCAGGACACAGGATCCTACACCGTAAAAATCATGAATCGAATGGAGGAGACTAAAGGAGTAACTGTACATTTCACCTTGTACC

>Mle_PSG1N (Mandrillus leucophaeus; drill) XR_001005167.1 TATCACTTTTAATCTTCTGGAACACGCCCACCACTGCCCAAGTCACGATTGAAGCCCAGCCAACCAAAGTGTCTGAGGGGAAGGATGTTCTGCTACTTGTCCACAATTTGCCCCAGAATCTTGCTGCCTACATCTGGTACAAAGGGCAAATAATGGACCTCCACCATTACATTACATCATATGTAATAGACACTGAAATAATTGTATTTGGGCCTGCATACAGTGGACGAGAAACAGTATATTCCAATGCATCCCTGCTGATCCAGAATGTCACCCAGAAGGACACAGGATCCTACACCATACAAATCATAAAGCGAGGTGATACCACTAAAGGAGTAACTGGACATTTCACCTTATACC

>Mle_PSG2N (Mandrillus leucophaeus; drill) XM_011982068.1

CATCACTTTTAATCTTCTGGAACCTGCCCACCACTGCCCAAGTCACGATTGAAGCCCAGCCAGCCAAAGTTTCTGAGGGGAAGGATGTTCTGCTACTTGTCCACAATTTGCCCCAGAATGTTGCTGGCTACAGCTGGTACAAAGGGCAAATAATGGACCTCCAGCATTACATTACAGCATATACAACAGACACTGAAATGATTATATTTGGGCCTGCATACAGTGGACGAGAAACAGTATATTCCAATGCATCCCTGCTGATCCAGAATGTCACCCAGAAGGACACAGGATCCTACACCATTCAAATCACAAAGCGAGGTGATGGGACTAAAAGAGTAACTGGACATTTCACTTTATACC

>Mle_PSG3aN (Mandrillus leucophaeus; drill) XM_011982728.1

CATCACTTTTAATCTTCTGGAACCCACCCACCACTGCTCAAGTCAGAATTGAAGCACAGCCAACCAAAGTTTCCGAGGGGAAGGATGTTCTTCTACTTGTCCACAATTTGCCCCAGAATCTTACTGGCTACATCTGGTACAAAGGGCAAAAAACGGACCTCCACCTTTACATTACATCATATGTAAAAGACACTGAAACAGTTATAGCTGGGCCTGCATACAGTGGACGAGAAACAATATATTCCAATGCATCCCTGCTGATCCAGAATGTCACCCAGAAGGACACAGGATCCTACACCATAGAAATCACAAAGCGAGGTGATAGGACTGAAGGAGAAACTGGACATTTCACCTTATATC

>Mle_PSG3bN (Mandrillus leucophaeus; drill) WGS JYKQ01009331.1

CATCACTTTTAATCTTCTGGAACCCGCCCACCACTGCTCAAGTCAGAATTGAAGCACAGCCAACCAAAGTTTCCGAGGGGAAGGATGTTCTTCTACTTGTCCACAATTTGCCCCAGAATCTTACTGGCTACATCTGGTACAAAGGGCAAAAAACGGACCTCCACCTTTATGTTACATCATATGTAAAAGACACTGAAACAGTTATAGCTGGGCCTGCATACAGTGGACGAGAAACAATATATTCCAATGCATCCCTGCTGATCCAGAATGTCACCCAGAAGGACACAGGATCCTACACCATAGAAATCACAAAGCGAGGTGATAGGACTGAAGGAGAAACTGGACATTTCACCTTATATCGT

>Mle_PSG4N (Mandrillus leucophaeus; drill) XM_011972296.1

TATCACTTTTAATCATCTGGAACCCACCCACCACTGCTCAAGTCACAATTGAAGCCCAGCCAACCAAAGTTTCCGAGGGGAAGGATGTTCTTCTACTTGTCCACAATTTGCCCCAGAATCCTACTGGCTACAGCTGGTACAAAGGGCAAATAACAGACATCCACCATTACATTACATCATATGTAATAGACACTGAAATGATTGTATTTGGGCCTGCATACAGTGGACGAGAAACAGTATATTCCAATGCATCCCTGCTGATCCAGAATGTCACCCAGAATGACACAGGATCCTACACCATAGAAATCATACAGCGAGGTGATACCACTAAAGGAGTAACTGGACATTTCACCTTATACC

>Mle_PSG5N (Mandrillus leucophaeus; drill) XM_011982141.1 CATCACTTTTAATCTTCTGGAACCCGCTTACCACTGCCCAAGTCACGATTGAAGCACAGCCAACCAAAGTGTCTGAGGGGAAGGATGTTCTGCTACTTGTCTACAATTTGCCCGAGAATCTTACTGGCTACAGCTGGTACAAAGGGCAAATAATGGACCTCCAGCATTACATTGCAGCATATACAACAGACACTGAAATGATTATATTTGGGCCTGCATACAGTGGACGAGAAACAGTATATTCCAATGCATCCCTGCTGATCCAGAATGTCACCAAGAATGACACAGGATCCTACACCATTCAAATCACAAAACGAGGTGATGGGACTAAAGGAGTAACTGGACATTTCACCTTATACC

>Mle_PSG6N (Mandrillus leucophaeus; drill) XM_011982721.1

CATCACTTTTAATCTTCTGGAACCCGCCCACCACTGCCCAAGTCACGATTGAAGCTCAGCCAACCAAAGTTTCTGAGGGGAAGGATGTTCTTCTACTTGTCCACAATTTGCCCACGAACGTTGTTGGCTACATCTGGTACAAAGGGCAAATAATGGACCTCCAGCATTACATTACAGCATATACAATAGACACTGAAAAGATTATATTTGGGCCTGCATACAGTGGACGAGAAACAGTATATTCTAATGCATCCCTGCTGATCCAGAATGTCACCAAGAATGACACAGGATCCTACAGCATTCAAATCACAAACCCATGTGATGAGACTAAAGGAGTAACTGGACATTTCACCTTATACG

>Mle_PSG7N (Mandrillus leucophaeus; drill) XR_001005382.1

CATCACTTTTAATCTTCTGGAACTCGCCCACCACTGCCCAAGTCACAATTGAAGCTCAGCCAACCAATATTTCCGAGGGGAATGATGTTCTTCTACTTGTACACAATTTACCCAAGAATCCTGTTGCCTACATCTGGTACAAAGGGCAAATAATGGACCTCCAACATTACATTACAGCATATACAATAGACACTGAAAGAATTATATTTGGGCCTGCATACAGTGGACGAGAAAGAGTATATTCCAATGCATCCCTGCTGATCCAGAGTGTGAACCAGAAGGACGCAGGATCCTACACCGTAAAAATCATAAAGCGAGGTTACAGGACTGAAGGAGTAACTGGACATTTCACCTTATATG

>Mle_PSG8N (Mandrillus leucophaeus; drill) XM_011982066.1

CATCACTTTTCATCTTTTGGAACCCACCTATCGCGGCCCAAGTCACGATTGAAGCTCATCCAACCGAAGTTTCTGAGGGGAAGGATGTTCTTCTACTTGTCCACAATTTGCCCCAGAATCCTACTGGCTACATCTGGTACAAAGGGCAAATAATGGATAGCCACAATTACATTACATCATATGTAATAGACACTGAAATGATTGTATTTGGGCCTGCATACAGTGGACGAGAAACAGTATATTCCAATGCATCCCTGCTGATCCAGAATGTCACCTGGAAGGACACAGGATCCTACACCATACAAATCATAAAGCGAGGTGATACCACTAAAGGAGTAACTGGACATTTCACCTTATATG

>Mle_PSG9N (Mandrillus leucophaeus; drill) XM_011982069.1

CGTCATTTTTAATCTTCTGGAACCCGCCCACCACTGCCCAAGTCATGATTGAAGCTCAGCCTACCAAAGTTTCTGAGGGGAAGGATGTTCTTCTACCTGTCCGCAATTTGCCCCAGAAAGTTGCTGCCTACATCTGGTACAAAGGGCAAATAATGGACTTCCACCAATTCATTACAGCATATACAATAGACACTGAAAGAATTATATTTGGGCCTGCATTCAGTGGACGAGAAACACTATATTCCAATGGATCCCTGCTGATCCGAAATGTCACCAAGAATGACACAGGATCCTACACCGTAAAAATCAGGAACCCAGCTGAGGAGACTAAAGGAGTAATTGTACATTTCACCTTATATC

>Mle_PSG10N (Mandrillus leucophaeus; drill) XM_011982070.1

CGTCATTTTTAATCTTCTGGAACCCGCCCACCACTGCCCAAGTCATGATTGAAGCTCAGCCAACCAAAGTTTCCGAGGGGAAGGATGTTCTTCTACTTGTCCGCAATTTGCCCCAGAAAGTTGCTGCCTATGTCTGGTACAAAGGGCAAATAATGGACTTCCACCAATTCATTACAGCATATACAATAGACCCTGAAAGAATTATATTTGGGTATGCATACAGTGGACGAGAAACACTATATTCCAATGGATCCCTGCTGATCCGGAATGTTACTAAGCAGGACACAGGGTCCTACACCGTAAAAATCATGAATCGAATGGAGGAGACTAAAGGAGTAACTGTACATTTCACCTTATACC

>Mle_PSG11N (Mandrillus leucophaeus; drill) XM_011972295.1

CATCACTTTTAATCTTCTGGAACCCACCCACCACTGCTCAAGTCACAATTGAAGCCCAGCCAGCCAAAGTTTCCGAGGGGAAGGATGTTCTTCTACTTGTCCACAATTTGCCCCAGAATCTTACTGGCTACATCTGGTACAAAGGGCAAAAAACGGACCTCCACCTTTACATTACATCATATGTGATAGACACTGAAAGAATTATATTGGGGCCTGCATACAGTGGACGAGAAACAGTATATTCCAATGCATCCCTGCTGATCCAGAATGTCACCCAGAAGGACACAGGATCCTACACCATACAAATCATAAAGCGAGGTCATAGGACTGAAGGAGTAACTGGACATTACACCTTATACC

>Mle_PSG12N (Mandrillus leucophaeus; drill) WGS JYKQ01009310.1

CATCACTTTTCATCTTCTGGAACCCACCCACCACTGCTCAAGTCACGATTGAAGCCCAGCCAACCAAAGTTTCCGAGGGGAAGGATGTTCTTCTACTTGTCCACAATTTGCCCCAGAATCTTACTGGCTACATCTGGTACAAAGGGCAAATAACGGACCACCACCATTACATTACATCATATGTGATAGACACTGAAACAATTATATTTGGGCCTGCATACAGTGGACGAGAAACAGTATATTCCAATGCATCCCTGCTGATCCAGAATGTCACCC

>Mle_PSG13N (Mandrillus leucophaeus; drill) WGS JYKQ01009344.1

CATCACTTTTAATCTTCTGGAACCCGCCCACCACTGCTCAAGTCACAATTGAAGCCCAGCCAGCCAAAGTTTCTGAGGGGAAGGATGTTCTTCTACTTGTCCACAATTTGCCCCAGAATCTTGCTGCCTGCATCTGGTACAAAGGGCAAATAATGGACCTCCAACATTATATTACAGCATATGTAATAGATGCT

>Mle_PSGP1N (Mandrillus leucophaeus; drill) WGS JYKQ01009310.1

CATCACTTTTAATCTTCTGGAACACACCCACCACTGCCCAAGTCACGATTGAAGCACAGCCAACCAAAGTTTCCGAGGGGAAGGATGTTCTTCTACTTGTCCAGAATTTGCCCCAGAATCTTATTGCCTACATCTGGTACAAAGGGCAAAAAACGGACTTCCGCCATTACATTACATCATATGTAATAGATGCTGAAACAATTATAGTTGGGCCTGCATAGAGTGGATGAGAAACAGTATATTCCAATGCATCCCTGCTGATCCAGAATGTCACCCAGAAGGACACAGGATCCTACACCATACAAATGATAAAGCAAGGTGATAAGACTAAAGGAGTAATTGGACATTTCACCTTATACCGT

>Mml_PSG1aN (Macaca mulatta; rhesus macaque) XM_015124111.1

CATCACTTTTAATCTTCTGGAACCCGCCCACCACTGCTCAAGTCACAATTGAAGCCCAGCCAGCCAAAGTTTCTGAGGGGAAGGATGTTCTTCTACTTGTCCACAATTTGCCCCAGAATCTTGCTGCCTGCATCTGGTACAAAGGGCAAATAATGGACCTCCAACATTACATTACAGCATATGTAATAGATGCTGAAACAATTATATTTGGGCCTGCATACAGTGGACGAGAAACAGTATATTCCAATGCATCCCTGCTGATCCAGAATGTCACCCAGAAGGACACAGGATCCTACACCATACATATCATACAGCGAGGTGATAAGACTAAAGGAGTAACTGGACATTTCACCTTATACC

>Mml_PSG2N (Macaca mulatta; rhesus macaque) WGS AANU01233436.1

CATCACTTTTAATCTTCTGGAACACGCCCACCACTGCCCAAGTCACGATTGAAGCCCAGCCAACCAAAGTTTCTGAGGGGAAGGATGTTCTGCTACTTGTACACAATTTGCCCCAGAATCTTGCTGCCTACATCTGGTACAAAGGGCACATAATGGACCTCCACCATTACATTACATCATATGTAATAGACACTGAAATAATTGTATTTGGACCTGCATACAGTGGACGAGAAACAGTATATTCCAATGCATCCCTGCTGATCCAGAATGTCACCCAGAAGGACACAGGATCCTACACCATACAAATCATAAAGCGAGGTGATACCACTAAAGGAGTAACTGGACATTTCACCTTATACC

>Mml_PSG3N (Macaca mulatta; rhesus macaque) XM_001116082.1

CATCACTTTTAATCTTCTGGAACCCGCCCACCACTGCCCAAGTCACGATCGAAGCACAGCCAACCAAAGTTTCTGAGGGGAAGGATGTTCTGCTACTTGTCCACAATTTGCCCCAGAATGTTGCTGGCTACAGCTGGTACAAAGGGCAAATAATGGACCTCCACCATTACATTACATCATATGTAATAGACACTGAAATCATTATATTTGGGCCTGCATACAGTGGACGAGAAACAGTATATTCCAATGCATCCCTGCTGATCCAGAATGTCACCCAGAAGGACACAGGATCCTACACCATTCAAATCACAAAGCGAGGTGATGGGACTAAAAGAGTAACTGGACATTTCACTTTATACC

>Mml_PSG4N (Macaca mulatta; rhesus macaque) WGS VSDM01000033.1

CATCACTTTTAATCTTCTGGAACCCGCCCACCACTGCTCAAGTCACGATTGAAGCCCAGCCAGCCAAAGTTTCCGAGGGGAAGGATGTTCTTCTACTTGTCCACAATTTGCCCCAGAATGTTACTGGCTACATCTGGTACAAAGGGCAAAAAACGGACCACCACCTTTACATTACATCATATGTAATAGACGCTGAAACAATTATACTTGGGCCTGCATACAGTGGACGAGAAACAGTATATTCCAATGCATCCCTGCTGATCCAGAATGTCACCTGGAAGGACACAGGATCCTACACCATAGAAATCATAAAGCGAGGTGATAGGACTGAAGGAGTAACTGGACATTATACCTTATACC

>Mml_PSG5N (Macaca mulatta; rhesus macaque) WGS VSDM01000604.1

CATCACTTTTAATCTTCTGGAACCCGACCACCACTGCTCAAGTCACGATTGAAGCCCAGCCAACCAAAGTTTCCGAGGGGAAGGATGTTCTTCTACTTGTCCACAATTTGCCCCAGAATCTTACTGGCTACATCTGGTACAAAGGGCAAATAATGGACCACCACCATTACATTACATCATATGTGATAGACACTGAAACAATTATATTTGGGCCTGCATACAATGAACGAGAAACAGTATATTCCAATGCATCCCTGCTGATCCAGAATGTCACCAAGAATGACACAGGATCCTACACCATACAAATCATAAAGCGAGGTGATAGGACTGAAGGAGTAACTGGACATTACACCTTATACC

>Mml_PSG6N (Macaca mulatta; rhesus macaque) WGS VSDM01000604.1

CATCACTTTTAATCTTCTGGAACCCGCCCACCACTGCCCAAGTCATGATTGAAGCCCAGCCAACCAAAGTTTCTGAGGGGAAGGATGTTCTGCTACTTGTCTACAATTTGCCCCAGAATCTTACTGGCTACAGCTGGTACAAAGGGCAAATAATGGACCTCCAGCATTACATTGCAGCATATACAATAGACACTGAAATGATTGTATTTGGGCCTGCATACAGTGGACGAGAAACAGTATATTCCAATGCATCCCTGCTGATCCAGAATGTCACCAAGAATGACACAGGATCCTACACCATTCAAATAACAAAGCAAGGTGATGAGACTAAAGGAGTAACTGGACATTTCACCTTATACC

>Mml_PSG7N (Macaca mulatta; rhesus macaque) WGS JSUE03110158.1

CATCACTTTTAATCTTCTGGAACCCGCCCACCACTGCGCAAGTCACGATTGAAGCTCAGCCAACCAAAGTTTCTGAGGGGAAGGATATTCTTCTACTTGTCCACAATGTGCCCCAGAATGTTGCTGGCTACATCTGGTACAAAGGGCAAATAATGGACCTCCAGCATTACATTACAGCATATGCAATAGACACTGAAACAATTATATTTGGGCCTGTATACAGTGGACGAGAAACAATATATTCCAATGCATCCCTGCTGATCCAGAGTGTCAACCAGAAGGATGCAGGATCCTACACCGTAGAAATCGTAAAGCAAGGTGAAGGGACTGAAGGAGTAACTGGACATTTCACCTTATACC

>Mml_PSG8N (Macaca mulatta; rhesus macaque) WGS JSUE03326926.1

TATCACTTTTAATCATCTGGAACCCACCTACCACGGGTCAAGTCACGATTGAAGCTCAGCCAACCGAGGTTTCTGAGGGGAAGGATGTTCTTCTACTTGTCCACAATTTGCCCCAGAATCCTACTGGCTACAGCTGGTACAAAGGGCAAATAACAGACATCCACCATTACATTACATCATATGTAATAGACACTGAAATGATTGTATTTGGGCCTGCATACAGTGGACGAGAAACAGTATATTCCAATGCATCCCTGCTGATCCAGAATGTCACCCAGAAGGACACAGGATCCTACACCATACAAATCATACAGCGAGGTGATACCACTAAAGGAGTAACTGGACATTTCACCTTATACC

>Mml_PSG9N (Macaca mulatta; rhesus macaque) CN647552

CATCACTTTTAATCTTCTGGAACCCTCCCACCACTGCCCAAGTCACGATTGAAGCACAGCCAACCAAACTTTCTGAGGGGAAGGATGTTCTCCTACTTGTCCACAATTTGCCCCAGAATCTTACTGGCTACAGCTGGTACAAAGGGCAAATAATAGACCTCCAGCATTACATTACAGCATATACAATAGACACTGAAATGATTGTACTTGGGCCTGCATACAGTGGACGAGAAACAGTATATTCCAATGCATCCCTGCTGATCCAGAATGTCACCAAGAATGACACAGGATCCTACACCATTCAAATCACAAAGCGAGGTGAAGGGACTGAAGGAGTAACTGGACATTTCACCTTATATC

>Mml_PSG10N (Macaca mulatta; rhesus macaque) WGS JSUE03304713.1

CATCACTTTTCATCTTCTGGAACCCGCCCACCACTGCCCAAGTCACGATTGAAGCCCAGCCAGCCAAAGTTTCCGAGGGGAAGGATGTTCTTCTACTTGTCCAGAATTTGCCTGAGAATCTTACTGGCTACGTCTGGTTCAAAGGGCAAATAATGGACTTCCATCAATTCATTACAGCGTATACAATAGACACTGAAACAATTATATTTGGGCCTGCATACAGTGGACGAGAAACAGTATATTCCAATGCATCCCTGCTGATCCAGAATGTCACCCAGAATGACACAGGATCCTACACCATAGAAATTATAAAGCGAGGTGATAAGATTAAAGGAGTAACTGGACATTTCACCTTATACG

>Mml_PSG11N (Macaca mulatta; rhesus macaque) WGS AANU01233432.1

CATCACTTTTAGTCTTCTGGAACTCGCCCACCACTGCCCAAGTCACAATTGAAGCTCAGCCAACCAATATTTCCGAGGGGAACGATGTTCTTCTACTTGTACACAATTTGCCCCAGAATCCTGCTGCCTACATCTGGTACAAAGGGCAAATAATGGACCTCCACCATTACATTACAGCATATACAATAGACACTGAAAGAATTATATTTGGGCCTGCATACAGTGGACGAGAAAGAGTATATTCCAATGCATCCCTGCTGATCCAGAGTGTGAACCAGAAGGACGCAGGATCCTACACCGTAAAAATCATAAAGCAAGGTGACGGGACTGAAGGAGTAACTGGACATTTCACCTTATACG

>Mml_PSG12N (Macaca mulatta; rhesus macaque) WGS SBKD01001532.1

CATCACTTTTAATCTTCTGGAACTCGCCCACCACTGCCCAAGTCACGATTGAAGCTCAGCCAACCAAAGTTTCTGAGGGGAAGGATGTTCTTCTACTTGTCCACAATTTGCCCACGAACGTTGTTGGCTACATCTGGTACAAAGGGCAAATAATGGACCTCCAGCATTACATTACAGCATATACAACAGACACTGAAATGATTCTATTTGGGCCTGCATACAGTGGACGAGAAACAGTATATTCCAATGCATCCCTGCTGATCCAGAGTGTCACCAAGAATGACACAGGATCCTACACCATACAAATCATAAAGCGAGGTCATAGGACTGAAGGAGTAACTGGACATTACACCTTATACC

>Mml_PSG13N (Macaca mulatta; rhesus macaque) WGS JSUE03308953.1

CATCACTTTTAATCTTCTGGAACCCGCCCACCACTGCTCAAGTCACAATTGAAGCCCAGCCAGCCAAAGTTTCTGAGGGGAAGGATGTTCTTCTACTTGTCCACAATTTGCCCCAGAATCTTACTGGCTACATCTGGTACAAAGGGCAAAAAACGGACCTCCACCTTTACGTTACATCATATGTAAAAGATACTGAAACAGTTATAGCTGGGCCTGCATACAGTGGACGAGAAACAGTATATTCCAATGCATCCCTGCTGATCCAGAATGTCACCCAGAAGGACACAGGATCCTACACCATACAAATCACAAAGCGAGGTGATAGGACTGAAGGAGAAACTGCACATTTCACCTTATATC

>Mml_PSG14N (Macaca mulatta; rhesus macaque) WGS QNVO02000225.1

CATCACTTTTAATCTTCTGGAACCCGCCCACCACTGCCCAAGTCACGATTGAAGCCCAGCCAACCAAAGTTTCTGAGGGGAAGGATGTTCTTCTACTTGTCCACAATTTGCCCCAGAATCTTACTGGCTACGTCTGGTACAAAGGGCAAATAATGGACCTCCACCAATTCATTACAGCGTATACAATAGACACTGACACAATTATATTTGGGCCTGCATACAGTGGACGAGAAAGAGTATATTCCAATGCATCCCTGCTGATCCAGAATGTCACCCGGAAGGACACAGGATCCTACACCATACAAATTATAAAGCGAGGTGATAAGATTAAAAGAATAACTGGACATTTCACCTTATACC

>Mml_PSG15N (Macaca mulatta; rhesus macaque) WGS AANU01045613.1

CATCACTTTTAATCTTCTGGAACCCGCCCACCACTGCCCAAGTCACGATTGAAGCCCAGCCAACCAAAGTTTCTGAGGGGAAGGATGTTCTTCTACTTGTCCAGAATTTGCCCCAGAATCTTATTGCCTACATCTGGTACAAAGGGCAAAAAACGGACTTCCACCATTACATTACATCATATGTAATAGATGCTGAAACAATTATAGTTGGGCCTGCATACAGTGGACGAGAAACAGTATATTCCAATGCATCCCTGCTGATCCAGAATGTCACCCAGAATGACACAGGATCCTACACCATTCAAATGATAAAACAAGGTGATAAGACTAAAGGAGTAATTGGACATTTCACCTTATACC

>Mml_PSG16N (Macaca mulatta; rhesus macaque) WGS SBKD01001025.1

CATCACTTTTAATCTTCTGGAGCCCGCCCACCACTGCCCAAGTCACGATTGAAGCTCAGCCAACCAAAGTTTCTGAGGGGAAGGATGTTCTTCTACTTGTCCACAATTTGCCCACGAACGTTGTTGGCTACATCTGGTACAAAGGGCAAATAATGGACCTCCAGCATTACATTACAGCATATACAACAGACACTGAAATGATTATATTTGGGCCTGCATACAGTGGACGGGAAACAGTATATTCCAATGCATCCCTGCTGATCCAGAGTGTCACCAAGAATGACACAGGATCCTACACCATACAAATCATAAAGCGAGGTCATAGGACTGAAGGAGTAACTGGACATTTCACCTTATACC

>Mml_PSG17N (Macaca mulatta; rhesus macaque) CN647817

CATCACTTTTAATCTTCTGGAACCCGCCCACCACTGCCCAAGTCACGATTGAAGCACAGCCAGCCAAGGTTTCTGAGGGGAAGGATGTTCTTCTACTTGTCCACAATTTGCCCCAGAATCTTACTGGCTACAGCTGGTACAAAGGGCAAATAATAGACCTCCAGCATTACATTACAGCATATACAATAGACACTGAAATGATTGTATTTGGGCCTGCATACAGTGGACGAGAAACAGTATATTCCAATGCATCCCTGCTGATCCAGAATGTCACCAAGAATGACACAGGATCCTACACCATTCAAATCACAAAGCGAGGTGATGAGACTAAAGGATTAACTGGACATTTCACCTTATACC

>Mml_PSG18N (Macaca mulatta; rhesus macaque) WGS VSDM01000604.1

CATCATTTTTAATCTTGTGGAACACGCCCACCACTGCCCAAGTCATGATTGAAGCTCAGCCTACCAAAGTTTCTGAGGGGAAGGATGTTCTTCTACTTGTCCGCAATTTGCCCCAGAAAGTTGCTGCCTTTGTCTGGTACAAAGGGCAAATAATGGACTTCCACCAATTCATTACAGCATATACAATAGACACTGAAAGAATTATATTTGGGTATGCATACAGTGGAAGAGAAACACTATATTCCAATGGATCCCTGCTGATCCGGAATGTCACCAAGCAGGACACAGGATCCTACACCGTAAAAATCATGAATCGAATGGAGGAGACTAAAGGAGTAACTGTACATTTCACCTTGTACC

>Mml_PSG19N (Macaca mulatta; rhesus macaque) WGS QNVO02000257.1

CATCACTTTTAATCTTCTGGAACCCGCCCACCACTGCCCAAGTCACAATTGAAGCTGAGCCAACCAATATTTCCGAGGGGAATGATGTTCTTCTACTTGTGCACAATTTACCCAAGAATCCTGCTGCCTACATCTGGTACAAAGGGCAAATAATGGACCTCCAAAATTACATTACAGCATATACAATAGACACTGAAAGAATTATATTGGGGCCTGCATACAGTGGACGAGAAAGAGTATATTCCAATGCATCCCTGCTGATCCAGAGTGTGAACCAGAAGGACGCAGGATCCTACACCGTAAAAATCATAAAGCGAGGTTACAGGACTGAAGGAGTAACTGGACATTTCACCTTATATG

>Mml_PSG20N (Macaca mulatta; rhesus macaque) WGS VSDM01000783.1

CATCACTTTTAATCTTCTGGAACCCACCCACCACTGCTCAAGTCACAATTGAAGCCCAGCCAGCCAAAGTTTCTGAGGGGAAGGATGTTCTTCTACTTGTCCAGAATTTGCCCCAGAATCTTATTGCCTACATCTGGTACAAAGGGCAAAAAACGGACTTCCACCATTACATTACATCATATGTAATAGATGCTGAAACAATTATAGTTGGGCCTGCATACAGTGGACGAGAAACAGTATATTCCAATGCATCCCTGCTGATCCAGAATGTCACCAAGAATGACACAGGATCCTACACCATTCAAATGATAAAGCAAGGTGATAAGACTAAAGGAGTAATTGGACATTTCACCTTATACC

>Mml_PSG21N (Macaca mulatta; rhesus macaque) WGS VSDM01000019.1

TATTACTTTTCATCTTCTGGAACCCGCCCACCACTGCCCAAGTCACGATTGAAGCCCAGCCAGCCAAAGTTTCCGAGGGGAAGGATGTTCTTCTACTTGTCCAGAATTTGCCCCAGAATCTTACTGGCTACGTCTGGTTCAAAGGGCAAATAACGACCTTCCACCAATTCATTATAGCATATAAAATAGACAGTAGAAAAATTACAGTTGGGCCTGCATACAGTGGACGAGAAAGAGTATATTCCAATGCATCCCTGCTGATCAAGAATGTCTCCCGGAAGGACACAGGATCCTACACCATTCAAATCATACAGCGAGGTGATAAGACTAAAGGAGTAACTGGACATTTCACCTTATACG

>Mml_PSGP1N (Macaca mulatta; rhesus macaque) WGS AANU01115000.1

CATCACTTTTAATCTTCTGGAACCCGCCCACCACTGCTCAAGTCACGATTGAAGCCCAGCCAAACAAAGTTTCCGAGGGGAAGGATGTTCTTCTACTTGTCCACAATTTGCCCCAGAATCTTACTGGCTACATCTGGTACAAAGGGCAAAAAATGGACCACTACCTTTACATTACATCATATGTAATAGACGCTGAAACAATTATATTTGGGCCTGCATACAGTGGACGAGAAACAGTATATTCCAATGCATCCCTGCTGATCCAGAATGCCACCTGGAAGGACACAGGATCCTACACCATCGAAATCATAAAGTGAGGTGATAGGACTGAAGGAGTAACTGGACATTATACCTTATACC

>Mml_PSGP2N (Macaca mulatta; rhesus macaque) WGS VSDM01000604.1

CATCACTTTTAATCTTCTGGAACTCGCCCACCACTGCTCAAGTCACGATTGAAGCCCAGCCAACCAAAGTTTCTGAGGGGAAGGATGTTCTTCTACTTGTCCACAATTTGCCCCAGAATCTTACTGGCTACATCTGGTACAAAGGGCAAATAATGGACCACCACCATTACATTACATCATATGTGATAGACACTGAAACAATTATATTTGGGCCTGCATACAGTGGATGAGAAACAGTATATTCCAATGCATCCCTGCTGATCCAAAATGTCACCTGGAAGGACACAGGATCCTATACCATACAAATCATAGAGCGAGGTGAAGGGACTGAAGGAGTAACTGGACATTTCACCTTATACC

>Mml_PSGP3N (Macaca mulatta; rhesus macaque) WGS VSDM01000604.

CCTCACTTTTTAATCTTCTGGAACTCGCCGACCACTGCCCAAGTCACAATTGAAGCTGAAGCCAACAATATTTCTGAGGGGAACGATGGTTCTTCTACTGTGCACAATTTAACCAAGAATCCTGCTGCCTACATCTGGTACAAAGGGCAAATAATGGGACCTCCAACAGTTACATTACAGCATATATAATAGACACTGAAAGAATTATATTTGGGCCTGCATACAGTGGACGAGAAGAGTATATTCCAATGCATCCCTGCTGATCCAGAGTGTGAACCAGAAGGACGCAGGATCCTACTACCGTAAAAATCATTAAAGCGAGGGTTACAGGACTGAAGGAGTAACTGGACATTTCACCTTATATG

>Mne_PSG1N (Macaca nemestrina; pig-tailed macaque) XM_011718418.1

CATCACTTTTAATCTTCTGGAACACGCCCACCACTGCCCAAGTCACGATTGAAGCCCAGCCAACCAAAGTTTCTGAGGGGAAGGATGTTCTGCTACTTGTACACAATTTGCCCCAGAATCTTGCTGCCTACATCTGGTACAAAGGGCAAATAATGGACCTCCACCATTACATTACATCATATGTAACAGACACTGAAATAATTGTATTTGGGCCTGCATACAGTGGACGAGAAACAGTATATTCCAATGCATCCCTGCTGATCCAGAATGTCACCCAGAAGGACACAGGATCCTACACCATACAAATCATAAAGCGAGGTGATACCACTAAAGGAGTAACTGGACATTTCACCTTATACC

>Mne_PSG2N (Macaca nemestrina; pig-tailed macaque) XM_011764538.1

CATCACTTTTAATCTTCTGGAACCCGACCACCACTGCCCAAGTCACGATTGAAGCCCAGCCAGCCAAAGTTTCTGAGGGGAAGGATGTTCTTCTACTTGTCCACAATTTGCCCCAGAATCTTGCTGCCTGCATCTGGTACAAAGGGCAAATAATGGACCTCCAACATTACATTACAGCATATGTAATAGATGCTGAAACAATTATATTTGGGCCTGCATACAGTGGACGAGAAACAGTATATTCCAATGCATCCCTGCTGATCCAGAATGTCACCCAGAAGGACACAGGATCCTACACCATACAAATCATACAGCGAGGTGATAAGACTAAAGGAGTAACTGGACATTTCACCTTATATG

>Mne_PSG3N (Macaca nemestrina; pig-tailed macaque) XM_011715391.1

CATCACTTTTAATCTTCTGGAACCCGCCCACCACTGCTCAAGTCACGATTGAAGCCCAGCCAAACAAAGTTTCCGAGGGGAAGGATGTTCTTCTACTTGTCCACAATTTGCCCCAGAATCTTACTGGCTACATCTGGTACAAAGGGCAAATAATGGACCTCCAACATTACATTACATCATATGTGATAGACACTGAAACAATTATATTTGGGCCTGCATACAATGAACGAGAAACAGTATATTCCAATGCATCCCTGCTGATCCAGAATGTCACCAAGAATGACACAGGATCCTACACCATACAAATCATAAAGCGAGGTGATAGGACTGAAGGAGTAACTGGACATTACACCTTATACC

>Mne_PSG4aN (Macaca nemestrina; pig-tailed macaque) WGS JZLF01044281.1; XM_011711271.1

CATCACTTTTAATCTTCTGGAACCCGCCCACCACTGCCCAAGTCACGATTGAAGCCCAGCCAACCAAAGTTTCTGAGGGGAAGGATGTTCTTCTACTTGTCCACAATTTGCCCCAGAATCTTACTGGCTACAGCTGGTACAAAGGGCAAATAATAGACCTCCAGCATTACATTACAGCATATACAATAGACACTGAAATGATTGTATTTGGGCCTGTATACAGTGGACGAGAAACAGTATATTCCAATGCATCCCTGCTGATCCAGAATGTCACCAAGAATGACACAGGATCCTACACCATTCAAATCACAAAGCGAGGTGATGAGACTAAAGGATTAACTGGACATTTCACCTTATACC

>Mne_PSG4bN (Macaca nemestrina; pig-tailed macaque) WGS JZLF01050868.1

CATCACTTTTAATCTTCTGGAACCCTCCCACCACTGCCCAAGTCACGATTGAAGCCCAGCCAACCAAAGTTTCTGAGGGGAAGGATGTTCTTCTACTTGTCCACAATTTGCCCCAGAATCTTACTGGCTACAGCTGGTACAAAGGGCAAATAATAGACCTCCAGCATTACATTACAGCATATACAATAGACACTGAAATGATTGTATTTGGGCCTGCATACAGTGGACGAGAAACAGTATATTCCAATGCATCCCTGCTGATCCAGAATGTCACCAAGAATGACACAGGATCCTACACCATTCAAATCACAAAGCGAGGTGATGAGACTAAAGGAGTAACTGGACATTTCACCTTATACC

>Mne_PSG5N (Macaca nemestrina; pig-tailed macaque) XM_011715265.1

CATCACTTTTAATCTTCTGGAACCCGCCCACCACTGCCCAAGTCATGATTGAAGCCCAGCCAACCAAACTTTCTGAGGGGAAGGATGTTCTGCTACTTGTCTACAATTTGCCCCAGAATCTTACTGGCTACAGCTGGTACAAAGGGCAAATAATGGACCTCCAGCATTACATTGCAGCATATACAATAGACACTGAAATGATTGTATTTGGGCCTGCATACAGTGGACGAGAAACAGTATATTCCAATGCATCCCTGCTGATCCAGAATGTCACCAAGAATGACACAGGATCCTACACCATTCAAATCACAAAGCAAGGTGATGAGACTAAAGGAGTAACTGGACATTTCACCTTATACC

>Mne_PSG6N (Macaca nemestrina; pig-tailed macaque) XM_011711273.1

CATCACTTTTAATCTTCTGGAACCCGCCCACCACTGCCCAAGTCACGATTGAAGCCCAGCCAACCAAAGTTTCTGAGGGGAAGGATGTTCTGCTACTTGTCCACAATTTGCCCCAGAATCTTACTGGCTACGTCTGGTACAAAGGGCAAATAATGGACCTCCACCAATTCATTACAGCGTATACAATAGACACTGAAACAATTATATTTGGGCCTGCATACAGTGGACGAGAAAGAGTATATTCCAATGCATCCCTGCTGATCCAGAATGTCACCCGGAAGGACACAGGATCCTACACCATTCAAATTATAAAGCGAGGTGATAAGATTAAAAGAATAACTGGACATTTCACCTTATACC

>Mne_PSG7N (Macaca nemestrina; pig-tailed macaque) XM_011718421.1

CATCACTTTTAATCTTCTGGAACTCGCCCACCACTGCCCAAGTCACAATTGAAGCTCAGCCAACCAATATTTCCGAGGGGAACGATGTTCTTCTACTTGTGCACAATTTACCCAAGAATCCTGCTGCCTACATCTGGTACAAAGGGCAAATAATGGACCTCCACCATTACATTACAGCATATACAATAGACACTGAAAGAATTATATTTGGGCCTGCATACAGTAGACGAGAAAGAGTATATTCCAATGCATCCCTGCTGATCCAGAGTGTGAACCAGAAGGACGCAGGATCCTACACCGTAAAAATCATAAAGCGAGGTGACGGGACTGAAGGAGTAACTGGACATTTCACCTTATACG

>Mne_PSG8N (Macaca nemestrina; pig-tailed macaque) XM_011715268.1

CATCACTTTTAATCTTCTGGAACTCGCCCACCACTGCCCAAGTCACGATTGAAGCCCAGCCAACCAAAGTTTCTGAGGGGAAGGATGTTCTTCTACTTGTCCACAATTTGCCCAAGAACATTGCTGGCTACATCTGGTACAAAGGGCAAATAATGGACCTCCAGCATTACATTACAGCATATACAACAGACACTGAAATGATTCTATTTGGGCCTGCATACAGTGGACGAGAAACAGTATATTCCAATGCATCCCTGCTGATCCAGAGTGTCACCAAAAATGACACAGGATCCTACACCATACAAATCATAAAGCGAGGTCATAGGACTGAAGGAGTAACTGGACATTACACCTTATACC

>Mne_PSG9N (Macaca nemestrina; pig-tailed macaque) XM_011721936.1

CATCACTTTTAATCTTCTGGAACACACCCACCACTGCCCAAGTCACGATTGAAGCTCAGCCAACCAAAGTTTCCGAGGGGAAGGATGTTCTTCTACTTGTCCACAATTTGCCCCAGAATCTTATCGCCTACATCTGGTACAAAGGGCAAAAAACGGACTTCCGCCATTACATTACATCATATGTAATAGATGCTGAAACAATTATAGTTGGGCCTGCATACAGTGGACGAGAAACAGTATATTCCAATGCATCCCTGCTGATCCAGAATGTCACCAAGAATGACACAGGATCCTACACCATTCAAATGATAAAGCAAGGTGATAAGACTAAAGGAGTAATTGGACATTTCACCTTATACC

>Mne_PSG10N (Macaca nemestrina; pig-tailed macaque) XR_979070.1

CATCACTTTTAATCTTCTGGAACCCGCCCACCACTGCTCAAGTCACAATTGAAGCCCAGCCAACCAAAGTTTCCGAGGGGAAAGATGTTCTTCTACTTGTCCACAATTTGCCCCAGAATCTTACTGGCTACATCTGGTACAAAGGGCAAAAAACGGACCTCCACCTTTACGTTACATCATATGTAAAAGATACTGAAACAGTTATAGCTGGGCCTGCATACAGTGGACGAGAAACAGTATATTCCAATGCATCCCTGCTGATCCAGAATGTCACCCAGAAGGACACAGGATCCTACACCATACAAATCACAAAGCGAGGTGATAGGACTGAAGGAGAAACTGCACATTTCACCTTATATC

>Mne_PSG11N (Macaca nemestrina; pig-tailed macaque) XM_011715390.1

CATCACTTTTAATCTTCTGGAACCCGCCTACCACTGCCCAAGTCACGATTGAAGCTCAGCCAGCCAAAGTTTCTGAGGGGAAGGATGTTCTTCTACTTGTCCACAATTTGCCCCAGAATATTGCTGGCTACATCTGGTACAAAGGGCAAATAATGGACCTCCAGCATTACATTACAGCATATACAATAGACACTGAAATGATTATATTTGGGCCTGCATACAGTGGACGAGAAACAGTATATTCCAATGCATCCCTGCTGATCCAGAATGTCACCAAGAATGACACAGGATCGTACAGCATTCAAATCACAAACCCATGTGATGAGACTAAAGCAGTAACTGGACATTTCACCTTATACC

>Mne_PSG12N (Macaca nemestrina; pig-tailed macaque) XM_011715266.1

CATCACTTTTAATCTTCTGGAACCCGCCCACCACGGCGCAAGTCACGATTGAAGCTCAGCCAACCAAAGTTTCTGAGGGGAAGGATATTCTTCTACTTGTCCACAATGTGCCCCAGAATGTTGCTGGCTACATCTGGTACAAAGGGCAAATAATGGACCTCCAGCATTACATTACAGCATATGCAATAGACACTGAAACAATTATATTTGGGCCTGTATACAGTGGACGAGAAACAATATATTCCAATGCATCCCTGCTGATCCAGAGTGTCAACCAGAAGGATGCAGGATCCTACACCGTAGAAATCGTAAAGCGAGGTGAAGGGACTGAAGGAGTAACTGGACATTTCACCTTATACC

>Mne_PSG13N (Macaca nemestrina; pig-tailed macaque) WGS JZLF01053851.1; XM_011717365.1

CATCACTTTTAATCTTCTGGAACCCGACCACCACTGCTCAAGTCACAATTGAAGCACAGCCAACCAAAGTTTCTGAGGGGAAAGATGTTCTTCTACTTGTCCACAATTTGCCCCAGAATCTTACTGGCTACATCTGGTACAAAGGGCAAAAAACGGACCTCCACCTTTACGTTACATCATATGTAAAAGATACTGAAACAGTTATAGCTGGGCCTGCATACAGTGGACGAGAAACAGTATATTCCAATGCATCCCTACTGATCCAGAATGTCACCAAGAAGGACACAGGATCCTACACCATACAAATCAAAAAGCGAGGTGATAGGACTGAAGGAGAAACTGCACATTTCACCTTATATC

>Mne_PSG14N (Macaca nemestrina; pig-tailed macaque) WGS JZLF01050876.1; XM_011715389.1

TATTACTTTTAATCTTCTGGAACCCGCCCACCACTGCCCAAGTCACGATTGAAGCACAGCCAACCAAAGTTTCCGAGGGGAAGGATGTTCTTCTACTTGTCCAGAATTTGCCTGAGAATCTTACTGGCTACGTCTGGTTCAAAGGGCAAATAATGGACTTCCACCAATTCATTACAGCGTATACAATAGACACTGAAACAATTATATTTGGGCCTGCATACAGTGGACGAGAAACAGTATATTCCAATGCATCCCTGCTGATCCAGAATGTCACCCAGAATGACACAGGATCCTACACCATAGAAATTATAAAGCGAGGTGATAAGATTAAAGGAGTAACTGGACATTTCACCTTATACT

>Mne_PSG15N (Macaca nemestrina; pig-tailed macaque) WGS JZLF01049801.1

CATCACTTTTAATCTTCTGGAACCCGCCCACCACTGCCCAAGTCACGATCGAAGCACAGCCAGCCAAAGTTTCTGAGGGGAAGGATGTTCTGCTACTTGTCCACAATTTGCCCCAGAATGTTGCTGGCTACAGCTGGTACAAAGGGCAAATAATGGACCTCCAACATTACATTACATCATATGTAATAGACACTGAAATCATTATATTTGGGCCTGCATACAGTGGACGAGAGACAGTATATTCCAATGCATCCCTGCTGATCCAGAATGTCACCCAGAAGGACACAGGATCCTACACCATTCAAATCACAAAGCGAGGTGATGGGACTAAAAGAGTAACTGGACATTTCACTTTATACC

>Mne_PSG16N (Macaca nemestrina; pig-tailed macaque) WGS JZLF01055767.1

TATCACTTTTAATCATCTGGAACCCACCTACCACGGGTCAAGTCACGATTGAAGCTCAGCCAACCGAGGTTTCTGAGGGGAAGGATGTTCTTCTACTTGTCCACAATTTGCCCCAGAATCCTACTGGCTACAGCTGGTACAAAGGGCAAATAACAGACATCCACCATTACATTACATCATATGTAATAGACACTGAAATGATTGTATTTGGGCCTGCATACAGTGGACGAGAAACAGTATATTCCAATGCATCCCTGCTGATCCAGAATGTCACCCAGAAGGACACAGGATCCTACACCATACAAATCATACAGCGAAGTGATACCACTAAAGGAGTAACTGGACATTTCACCTTATACC

>Mne_PSG17N (Macaca nemestrina; pig-tailed macaque) WGS JZLF01053846.1

CATCACTTTTAATCTTCTGGAACCCACCCACCACTGCTCAAGTCACAATTGAAGCCCAGCCAACCAAAGTTTCCGAGGGGAAGGATGTTCTTCTACTTGTCCACAATTTGCCCCAGAATCTTACTGGCTACATCTGGTACAAAGGGCAAAAAACGGACCACTACCTTTACATTACATCATATGTAATAGACGCTGAAACAATTATATTTGGGCCTGCATACAGTGGACGAGAAACAGTATATTCCAATGCATCCCTGCTGATCCAGAATGTCACCTGGAAGGACACAGGATCCTACACCATAGAAATCATAAAGCGAGGTGATAGGACTGAAGGAGTAACTGGACATTATACCTTATACC

>Mne_PSG18N (Macaca nemestrina; pig-tailed macaque) WGS JZLF01049803.1

CATCACTTTTAATCTTCTGGAACCCACCTATCGTGGCCCAAGTCACGATTGAAGCCCAGCCAACCGAAGTTTCTGAGGGGAAGAATGTTCTTCTACTTGTCCACAATTTGACCCAGAATCCTACTGGCTACATCTGGTACAAAGGGCAAATAACGGATAGCCACAATTACATTACATCATATGTAATAGACACTGAAATGATTATATTTGGGCCTGCATACAGTGGACGAGAAACAGTATATTCCAATGCATCCCTGCTGATCCAGAATGTCACCAGGAAGGACACAGGATCCTACACCATACAAATCATAAAGCGAGGTGATAGCACTAAAGGAGTAACTGGACATTTTACCTTATACC

>Mne_PSG19N (Macaca nemestrina; pig-tailed macaque) WGS JZLF01050883.1

CATCACTTGTAATCTTCTGGAACCCGCCCACCACTGCGCAAGTCACGATTGAAGCTCAGCCAACCAAAGTTTCTGAGGGGAAGGATATTCTTCTACTTGTCAACAATGTGCCCCAGAATATTGCTGGCTACAGCTGGTACAAAGGGCAAATAATGGACCTCCAGCATTACATTACAGCATATGCAATAGACACTGAAACAATTATATTTGGCCCTGCATACAGTGGACGAGAAACAATATATTCCAATGCATCCCTGCTGATTCAAAGTGTCAACCAGAAGGATGCGGGATCCTACACTGTAGAAATCATAAAGCGAGGTGAAGGGACTGAAGGAGTAACTGGACATTTCACCTTATACC

>Mne_PSG20N (Macaca nemestrina; pig-tailed macaque) WGS JZLF01057426.1

CATCACTTTTAATCTTCTGGAACTCGCCGACCACTGCCCAAGTCACAATTGAAGCTGAGCCAACCAATATTTATGAGGGGAATGATGTTCTTCTACTTGTGCACAATTTAACCAAGAATCCTGCTGCCTTCATCTGGTACAAAGGGCAAATAATGGACCGCCACCATTACATTACAGCATATATAATAGCCACTGAAAGTATTATATTTGGGCCTGCATACAGTGGACGAGAAAGAGTATATTCCAATGCATCCCTGCTGATCCAGAGTGTGAACCAGAAGGACGCAGGATCCTACACCGTAAAAATCATAAAGCGAGGTTACAAGACTGAAGGAGTAACTGGACATTTCACCTTATATG

>Mne_PSG21N (Macaca nemestrina; pig-tailed macaque) WGS JZLF01044278.1

TATTACTTTTCATCTTCTGGAACCCGCCCACCACTGCCCAAGTCACGATTGAAGCCCAGCCAGCCAAAGTTTCCGAGGGGAAGGATGTTCTTCTACTTGTCCAGAATTTGCCCCAGAATCTTACTGGCTACGTTTGGTTCAAAGGGCAAATAACGACCTTCCACCAATTCATTATAGCATATAAAATAGACAGTAGAAAAATTACAGTTGGGCCTGCATACAGTGGACGAGAAAGAGTATATTCCAATGCATCCCTGCTGATCCAGAATGTCTCCCGGAAGGACACAGGATCCTACAGCATTCAAATCATAAAGCGAGGTGATAAGATTAAAGGGGTAACTGGACATTTCACCTTATACG

>Mne_PSGP1N (Macaca nemestrina; pig-tailed macaque) WGS JZLF01049798.1

CATCACTTTTAATCTTCTGGAACTCGCCCACCACTGCTCAAGTCACGATTGAAGCCCAGCCAACCAAAGTTTCTGAGGGGAAGGATGTTCTTCTACTTGTCCACAATTTGCCCCAGAATCTTACTGGCTACATCTGGTACAAAGGGCAAATAATGGACCTCCAACATTACATTACATCATATGTGATAGACACTGAAACAATTATATTTGGGCCTGCATACAGTGGATGAGAAACAGTATATTCCATTGCATCCCTGCTGATCCAAAATGTCACCTGGAAGGACACAGGATCCTATACCATACAAATCATAGAGCGAGGTGAAGGGACTGAAGGAGTAACTGGACATTTCACCTTATACC

>Msp_PSG1N (Mandrillus sphinx; Mandrill) WGS SRPC01039457.1

CATCACTTTTAATCTTCTGGAACCTGCCCACCACTGCCCAAGTCACGATTGAAGCCCAGCCAGCCAAAGTTTCTGAGGGGAAGGATGTTCTGCTACTTGTCCACAATTTGCCCCAGAATGTTGCTGGCTACAGCTGGTACAAAGGGCAAATAATGGACCTCCAGCATTACATTACAGCATATACAACAGACACTGAAATGATTATATTTGGGCCTGCATACAGTGGACGAGAAACAGTATATTCCAATGCATCCCTGCTGATCCAGAATGTCACCCAGAAGGACACAGGATCCTACACCATTCAAATCACAAAGCGAGGTGATGGGACTAAAAGAGTAACTGGACATTTCACTTTATACC

>Msp_PSG2N (Mandrillus sphinx; Mandrill) WGS SRPC01039457.1

CATCACTTTTCATCTTTTGGAACCCACCTATCGCGGCCCAAGTCACGATTGAAGCTCATCCAACCGAAGTTTCTGAGGGGAAGGATGTTCTTCTACTTGTCCACAATTTGCCCCAGAATCCTACTGGCTACATCTGGTACAAAGGGCAAATAATGGATAGCCACAATTACATTACATCATATGTAATAGACACTGAAATGATTGTATTTGGGCCTGCATACAGTGGACGAGAAACAGTATATTCCAATGCATCCCTGCTGATCCAGAATGTCACCTGGAAGGACACAGGATCCTACACCATACAAATCATAAAGCGAGGTGATACCACTAAAGGAGTAACTGGACATTTCACCTTATATG

>Msp_PSG3N (Mandrillus sphinx; Mandrill) WGS SRPC01029182.1

TATCACTTTTAATCTTCTGGAACACGCCCACCACTGCTCAAGTCACGATTGAAGCCCAGCCAACCAAAGTGTCTGAGGGGAAGGATGTTCTGCTACTTGTCCACAATTTGCCCCAGAATCTTGCTGCCTACATCTGGTACAAAGGACAAATAATGGACCTCCACCATTACATTACATCATATGTAATAGACACTGAAATAATTGTATTTGGGCCTGCATACAGTGGACGAGAAACAGTATATTCCAATGCATCCCTGCTGATCCAGAATGTCACCCAGAATGACACAGGATCCTACACCATACAAATCATAAAGCGAGGTGATAAGACTAAAGGAGTAACTGTACATTTCACCTTATACC

>Msp_PSG4N (Mandrillus sphinx; Mandrill) WGS SRPC01029182.1

CATCACTTTTAATCTTCTGGAACCCGCCCACCACTGCTCAAGTCACAATTGAAGCCCAGCCAGCCAAAGTTTCCGAGGGGAAGGATGTTCTTCTACTTGTCCACAATTTGCCCCAGAATCTTACTGGCTACATCTGGTACAAAGGGCAAAAAACGGACCACCACCTTTACATTACATCATATGTGATAGACACTGAAACAATTATATTTGGGCCTGCATACAGTGGACGAGAAACAGTATATTCCAATGCATCCCTGCTGATCCAGAATGTCACCCGGAAGGACACAGGATCCTACACCATAGAAATCATAAAGCGAGGTGATAGGACTGAAGGAGTAACTGGACATTACACCTTATACC

>Msp_PSG5N (Mandrillus sphinx; Mandrill) WGS SRPC01026172.1 no splice acceptor

CATCACTTTTAATCTTAATCTACCCGCCCACCACTGCCCAAGTCACGATTGAAGCCCAGCCAGCCAAAGTTTCCGAGGGGAAGGATGTTCTTCTACTTGTCCAGAATTTGCCCCAGAATCTTACTGGCTACGTCTGGTACAAAGGGCAAAAAACAGACCTCCACCAATTCATTACAGCATATACGATAGACACTGAAACAATTATATCTGGGCCTGCATACAGTGGACGAGAAACAGTATATTCCAATGCATCCCTGCTGATCCAGAATGTCACCCGGAAGGACACAGGATCCTACACCATACAAATTATAAAGCGAGGTGATAAGATTAAAAGAATAACTGGACATTTCACCTTATACC

>Msp_PSGP1N (Mandrillus sphinx; Mandrill) WGS SRPC01050543.1 no splice donor

CATCATTTTTAATCTTGTGGAACCCGCCCACCACTGCCCAAGTCATGATTGAAGCTCAGCCAACCAAAGTTTCCGAGGGGAAGGATGTTCTTCTACTTGTCCGCAATTTGCCCCAGAAAGTTGCTGCCTATGTCTGGTACAAAGGGCAAATAATGGACTTCCACCAATTCATTACAGCATATACAATAGACCCTGAAAGAATTATATTTGGGTATGCATACAGTGGACGAGAAACACTATATTCCAATGGATCCCTGCTGATCCGGAATGTCACCAAGAATGACACAGGATCCTACACCGTAAAAATCAGGAACCCAGCTGAAGAGACTAAAGGACTAATTGTACTCCTGCCAGGCTTCA

>Nla_PSG1N (Nasalis larvatus; proboscis monkey) WGS JMHX01319548.1

CATCACTTTTAATCTTCTGGAACCAGCCCACCACTGCCCAAGTCATGATTGAAGCACAGCCAACCAAAGTTTCTGAGGGGAAGAATGTTCTTCTACTTGTCCACAATTTGCCCCAGAATGTTGCTGCCTACATCTGGTACAAAGGGCAAATAATGGACGTCCACCATTACATTACGGGATATGTAATGGAGGCTGAAACAAATATATTTGGGCCTGCATACAGTGGACGAGAAACAGTATATTCCAATGCATCCCTGCTGATTCAGAATGTCACCCAGAAGGACACAGGATCCTACACCATACAAATCATGAAGCTAGGTAATAGGACTGAAGGAGTAACTGGACATTTCACCTTATACC

>Nla_PSG2N (Nasalis larvatus; proboscis monkey) WGS JMHX01319548.1

CATCACTTTTAATCTTCTGGAACCCGCCCACCACTGCTCAAGTCATGATTGAAGCCCAGCCAACCAAAGTTTCTGAGGGGAAGGATGTTCTTCTACTTGTCCACAATTTGCCCCAGAATCTTACTGGCTACGTCTGGTACAAAGGGCAAATAATGGACTACCACCATTACATTACAGCATATACAATAGACACTGAAACAACTATATTTGGGCCTGCATACAGTGGACGAGAAACAGTATATTCCAATGCATCCCTGCTGATCCAGAATGTCACCCAGAAGGACACAGGATCCTACACCATTCAAATCACACAGCGAGGTGATGGGACTAAAGGAGTAACTGGACATTTCACCTTATACC

>Nla_PSG3N (Nasalis larvatus; proboscis monkey) WGS JMHX01319548.1

CATCACTTTTAATCTTCTGGAACCCGCTCACCACTGCCCAAGTCACGATTGAAGCTCAGCCAACCAATGTTTCTGAGGGGAACAATGTTCTTCTACTTGTTCACAATTTGCCCCAGAATCCTGCTGCCTACATCTGGTACAAAGGGCAAATAATGGATCTCCACCATTACATTACAGCATATGTAATAGAAACTGAAAGAATTGTATTTGGGCCTGCATACAGTGGACGAGAAACAGTATATTCCAATGCATCCCTGCTGATCCAGAGTGTCAACCAGAAGGACGCAGGATCCTACACCGTAGAAATCATAAAGCGAGGTGATGGGAATGAAGGAGTAACTGGAAATTTCACCTTATACC

>Nla_PSGP2N (Nasalis larvatus; proboscis monkey) WGS JMHX01319548.1

CATCACTTTCAATCTTCTGGAACCCACCCACCACTGCCCAAGTCACGATTGAAGCTCAGCCAACCAAAGTTATGAAGGGAAGGATGTTCTTCTACTTGTCCGCAATTTGCCCCAGAATCCTGCTGCCTACTTCTGGTACAAAGGGCAAATAATGGACCTCCACCATTACATTCAGCAATACAATAGACACTGAAAGAGTTATATTTGGGCCTGCATACAGTGGACGAGAAACAGTATATTCCAATGCTTCCCTGCTGATCCAGAATGTCACCTAGAAGGACGCAGGATCCTACACCGTAGAAATCATAAAGTGAGGTGATAGGACTGAAGGAGTAACTGGACATTTCACCTTATACC

>Nle_PSG1N (Nomascus leucogenys; northern white-cheeked gibbon) XM_012496704.2; WGS ADFV01127702.1

CATCACTTTTAAACTTCTGGAACACGCCCACCACTGCCCAAGTCACGATTGAAGCCCAGCCACCAAAACTTTCCGAGGGAAAGGACGTTCTTCTACTTGTCCACAATTTGCCCCAGAATCTTGCTGCCTACATCTGGTACGAAGGGCAAATGATGGACATCCACCATTACATTACATCATATGTAGTAGATGATCAAACAATTGTATATGGGCCTGCATACAGTGGACGAGAAACAGTATATTCCAATGCATCCCTGCTGATCCAGAATGTCACGGGGGAGGATGCAGGATCCTACACCTTACAAATCATAAAGCGAGGTGATGGGATTAGAGGAGCAACTGGACATTTCACCTTCACCTTATACC

>Nle_PSG2N (Nomascus leucogenys; northern white-cheeked gibbon) WGS ADFV01127703.1

CATCACTTTTAAACTTCTGGAACCCGCCCACCACTGCCCAAGTCACTATTGAAGCCCAGCCACCCAAACTTTCCGAGGGGAAGGACGTTCTTCTACTTGTCCACAATTTGCCCCAGAATCTTACTGGCTACACCTGGTACAAAGGGCAAATGACGGACCTCTACCATTACATTACATCATATGTAGTAGACAATGACATAATTATATCTGGACCTGCATACACTGGACGAGAAACAGTATATTCCAACGCATCCCTGCTGATCCAGAATGTCACGCAGGAGGACACAGGACCCTACACCTTACACATCATAAAGCGAGGTGATGAGACTAGAGAAGCAACTGGAAATTTCACCGTCACCTTATACC

>Nle_PSG3N (Nomascus leucogenys; northern white-cheeked gibbon) WGS ADFV01127709.1

CGTCACTTTTAAACTTCTGGAACCCGCCCACCTCTGCCCAAGTCACTATTGAAGCCCAGCCACCAAAACTTTCTGAGGGGAAGGACGTTCTTCTACTTGTCCACAATTTGCCCCAGAATCTTACTGGCTACACCTGGTACAAAGGGCAAATGACGGACCTCTACCATTACATTACATCATATGTAGTAGACAATGACATAATTATATCTGGGCCTGCATACACTGGACGAGAAACAGTATATTCCAATGCATCCCTGCTGATCCAGAATGTCACGTGGGAGGACACAGGACCCTACACCTTACACATCATAAAGCGAGGTGATGAGACTAGAGAAGCAACTGGAAATTTCACCATCACCTTATACC

>Nle_PSG4N (Nomascus leucogenys; northern white-cheeked gibbon) XM_030797136.1; WGS VIFL01001378.1

CATCACTTTTACACATCTGGAACGCACCCACCACTGCCCAAGTCACGATTGAAGCCCAGCCACCCAAACTTTCTGAGGGGAAGGATATTCTTCTACTTGTCCACAATTTGCCCAAGAATCTTGCTGGCTACATCTGGTACAAAGGGCAAATGACGGACCTCCAGCATTACATTACATCATATCTAGTACACAATCATAAAGTTATACCTGGGCCTGAATACACTGGACGAGAAACAGTATATTGCAATGCATCCCTGCTGATCGAGGATGTCACACGGGAGGACGCAGGATCCTACACCTTACAAATCATAAAGCGAGGTGATGGGATTAGAGGAGCAACTGGACATTTCACCGTCACCTTATACC

>Nle_PSG5N (Nomascus leucogenys; northern white-cheeked gibbon) WGS ADFV01127702.1

CATCACTTTTACACTTCTGGAACCCGCCCACCACTGCCCAAGTCACGATTGAAGCCCAGCCACCCAAAATTTCCGAGGGGAAGGATGTTCTTCTACTTGTCCACAATTTGCCCCAGAATCTTGATGGCTACTTCTGGTACAAAGGGCAAATGACGGGCGTCCAGCATTACATTACAGCATATGCAGCAGACCGTCAAATAATTACATCTGGGCCTGCATACAGTGGACGAGTAACACTATATTCCAATGGATCCCTGCTGATCCAGAATGTCACCCAGGAGGACACAGGATCCTACACCTTAGAAATCATAAAGCGAGTTGGTGAGACTAGAGGAGTAACTGGATATTTCAACGTCACCTTATACT

>Nle_PSG6N (Nomascus leucogenys; northern white-cheeked gibbon) WGS ADFV01127707.1

CATCACTTTTAAACTTCTGGAACCCGCCCACCACTGCCCAAGTCATGATTGAAGCCCAGCCACCCAAAATTTCTGAGGGGAAGGATGTTCTTCTACATGTCCACAATTTGCCCCAGAATCTTACTGGCTACATCTGGTACAAAGGGCAAATGACGGACCATTACCATTACATTACATCATATGTAGTAGACAATCAGATAATTATATCTGGGCCTGCATACACTGGACGAGAAACAGTATATTCCAACGCATCCCTGCTGATCCAGAATGTCACGCAGGAGGACACAGGATCCTACACCTTACACATCATAAAGCGAGGTGATGAGACTACAGGAGTAACTGGACATTTCACTGTCACCTTACACC

>Nle_PSG7N (Nomascus leucogenys; northern white-cheeked gibbon) WGS ADFV01135414.1

CATCGCTTTTAAACTTCTGGAACCCGCCTACCACTGCCCAAGTCATGATTGAAGCCCAGCCACCCAAAGTTTCTGAGGGGAAGGATGTTCTTCTACTTGTCCACAATTTGCCCCATAATCTTGCCAGCTATAGCTGGCACAAAGGGCGAATGATGGACCTCCAGCATTACATTACATCATATGTAGTAGCCAGTCAAATAATTTTATCTGGGCCTGCATACAGTGGACGAGAAACACTATATTCCAATGCATCCCTGCTGATCCAGAATGTCACCCGGGAGGACGCAGGATCCTACACCTTAAACATCAGACAGCGAGGTGATGGGATTAGAGGAGTAACTGGACATTTCACCTTCACTTTGTACC

>Nle_PSG8N (Nomascus leucogenys; northern white-cheeked gibbon) WGS ADFV01127714.1

CATCACTTTTAAACTTCTGGAACCCGCCCGCCAGTGCCCAAGTCATGATTGAAGCCCAGCCACCCAAAATTTCTGAGGGGAAGGATGTTCTTCTATTTGTCCACAATTTGCCCCAGAATCTTTCTGGCTACATCTGGTACAGAGGGCAAGCGACAGACATCTACCATTACATTGCATCATATGTAGTAGACAATGACATAATTATATATGGGCCTGCATACACTGAACGAAAAACAGTATATTCCAATGCATCCCTGCTGATCCAGAATGTCACCCGGGAGGACGCAGGATCCTACACCTTACACATCATAAAGCGAGGTGATGAGACTACAGGAGTAACTGGACACTTCACCGTCACCTTATACC

>Nle_PSG9N (Nomascus leucogenys; northern white-cheeked gibbon) WGS ADFV01127704.1

CATCACTTTTAAACTTCTGGAACCCGCCCACCACTGCCCAAGTCATGATTGAAGCCCAGCCACCCAAAATTTCTGAGGGGAAGGATGTTCTTCTACTTGTCCACAATTTGCCCCAGAATCTTGCTGGCTACATGTGGTACAAAGGGCAAATGACGGACCTCTACCATTACATTATATCATATATAGCAGACAGTCAAACAATTATACCTGGGCCTGCATACAGTGGGCGAGAAACAGTATATTCCAATGCATCCCTGCTGATCCAGAAAGTCACGCGGGAGGACGCAGGATCCTACAACTTACACATCATAAAGCGAGGTGATGAGACTACAGGAGTAACTGGACATTTCACTGTCACCTTATACT

>Nle_PSG10N (Nomascus leucogenys; northern white-cheeked gibbon) WGS VIFL01002291.1

CATCACTTTTAAACTTCTGGAACCCGCCCACCACTGCCCAAGTCACTATTGAAGCCCAGCCACCCAAACTTTCCAAGGGGAAGGACGTTCTTCTACTTGTCCACAATTTGCCCCAGAATCTTACTGGCTACACCTGGTACAAAGGGCAAATGATGGACCTCTACCATTACATTACATCATATGTAGTAGACAATGACATAATTATATCTGGACCTGCATACACTGGACGAGAAACAGTATATTCCAACGCATCCCTGCTGATCCAGAATGTCACGCAGGAGGACACAGGACCCTACACCTTACACATCATAAAGCGAGGTGATGAGACTAGAGAAGCAACTGGAAATTTCACCATCACCTTATACC

>Nle_PSG11N (Nomascus leucogenys; northern white-cheeked gibbon) WGS VIFL01001624.1

CATCACTTTTAAACTTCTGGAACCCGCCCACCACTGCCCAAGTCACTATTGAAGCCCAGCCACCAAAACTTTCTGAGGGGAAGGATGTTCTTCTACATGTCCACAATTTGCCCCAGAATCTTACTGGCTACATCTGGTACAAAGGGCAAATGACGGACCATTACCATTACATTACATCATATGTAGTAGACAATCAGATAATTATATCTGGGCCTGCATACACTGGACGAGAAACAGTATATTCCAACGCATCCCTGCTGATCCAGAATGTCACCCGGGAGGACACAGGATCCTACACCTTACACATCATAAAGCGAGGTGATGAGACTACAGGAGTAACTGGACATTTCACTGTAACCTTACACC

>Pab_PSG1N (Pongo abelii; Sumatran orangutan) XM_002829303.3

CATCACTTTTAAACTTCTGGAACCTGCCTACCACTGCCCAAGTCACGATTGAAGCCCAGCCACCCAAAGTTTTCGAGGTGAAGGATGTTCTTCTACTTGTCCACAATTTGCCCCAGAATCTTGCTGGCTACATCTGGTACAAAGGGCAAATGACGGACCTCTACCATTACATTACATCATATGTAGTAGATGGTGAAAGAATTATATATGGGCCTGCATACAGTGGACGAGAAACAGTATATTCCAATGCATCCCTGTTGATCCAGAATGTCACCCGGGAAGATGCAGGATCCTACACCTTACACATCATAAAGCGAGGTGATGGGACAAGAGGAGAAACTGGACATTTCAGCGTCAACTTATACC

>Pab_PSG2N (Pongo abelii; Sumatran orangutan) XM_009236717.1

CATCACTTTTAAACTTCTGGAACCCGCTTACCACTGCCCAAGTCATGATTGAAGCCCAGCCACCCAAAGTTTCTGAGGGGAAGGATGTTCTTCTACTTGTCCACAGTTTGCCCCAGAATCTTACTGGCTACATCTGGTACAAAGGGCAAATGAAGGACCTCTACCATTACATTACATCATACGAAGTAGACGGTCAAATAATTATACATGGGCCTGCATACAGTGGACGAGAAACAGTATATTCCAATGCATCCCTGCTGATCCAGAATGTCACCCGGGAGGACGCAGGATCCTACACCTTACACATCATAAAGCGAGGTGATGGGACTAGAAGAGTAACTGGAAATTTCACCTTCACCTTCTGTG

>Pab_PSG3N (Pongo abelii; Sumatran orangutan) WGS ABGA01281962.1

CATCACTTTTAAACTTCTGGAACCCGCCTGCCACTGCCCAAGTCATGATTGAAGCCCAGCCACCCAAAGTTTCTGAGGTGAAGGATGTTCTTCTACTTGTCCACAATTTGCCCCAGAATCTTGCTGGCTACATCTGGTACAAAAGGCAAATGATGGACCTCTACCATTACATTACATCATATGTAGTAGACGGTCAAATAATTATATATGGGCCTGCATACAGTGGACGAGAAACAGTATATTCCAATGCATCCCTGCTGATCCACAATGTCACCGGGGAGGATGCAGTATCCTACACCTTACACATCATAAAGCGAGGTGATGGGACTAGAAGAGTAACTGGAAATTTCACCTTCACCTTATACC

>Pab_PSG4N (Pongo abelii; Sumatran orangutan) XM_024237493.1

CATCACTTTTAAACTTCTGGAACCCACCCACCACTGCCCAAGTCACGATTGAAGCCCAGCTACCCAAAGTTTCTGAGGCGAAGGATGTTCTTCTACTTGTCCACAATTTGCCCCAGAATCTTGCTGGCTACGTTTGGTACAAAGGGCAAATGACATACCTCTACCATTACATTACATCATATGTAGTAGACGGTCAAAGAATTATATATGGGCCTGCATACAGTGGACGAGAAACAGTATATTCCAATGCATCCCTGCTGATCCAGAATGTCACCTGGGAGGACGCAGGATCCTACACCTTACACATCATAAAGCGAGGTGATGGGACTGCAGGAGTAACTGGACATTTCACCTTCACCTTATACC

>Pab_PSG5N (Pongo abelii; Sumatran orangutan) XM_009232681.1

CATCACTTTTAAACTGCTGGAACCCGCCCACCACTGCCCAAGTCACGATTGAAGCCCAGCCACCCAAAGTTTCTGCGGGGAAGGATGTTCTTCTACTTGTCCACAATTTGCCCCAGAATCTTGCTGGCTACATCTGGTACAAAGGGCAAATAATGGACCTCTACCATTACATTACATCATACGTAGTAGACGGTCAAACAATTATATATGGGCCTGCATACAGTGGACGAGAAACAGTATATTCCAATGCATCCCTGCTGATCCAGAATGTCACCCGGGAGGACGCAGGATCCTACACCTTACACATCATAAAGCGAGGTGATAGGACTAGAGGAGTAACTGGATATTTCACCTTCACCTTATACC

>Pab_PSG6N (Pongo abelii; Sumatran orangutan) XM_009232675.1

CATCACTTTTAAACTTCTGGAACACGCCTACCACTGCTCAAGTCACGATTGAAGCCCAGCCACCCAAAGTTTCTGAGGGGAAGGATGTTCTTCTACTTGTCCACAATTTGCCCAAGAATCTTACTGGCTACATCTGGTACAAAGGGCAAATGAGGGACCTCTACCATTACATTACATCATACGTAGTAGACGGTCAAACAATTATATATGGGCCTGCATACAGTGGACGAGAAACAGTATATTCCAATGCATCCCTGTTGATCCAGAATGTCACCCGGGACGACGCAGGATCCTACACCTTACACATCATAAAGCGAGGTGATGGGACTAGAGGAATAACTGGACATTTCACCTTCACCTTATACC

>Pab_PSG8N (Pongo abelii; Sumatran orangutan) XM_024237485.1

CATCACTTTTAAACTTCTGGAATCCACCTACCACTGCCCAAGTCACGATTGAAGCCCAGCCACCCAAAGTTTCTGAGGGGAAGGATGTTCTTCTACTTGTCCACAATTTGCCCCAGAATCTTACTGGCTACATCTGGTACAAACGAAAAATGACGGACCTCTACCATTACATTACATCATATGTAGTAGATGGTCAAATAATTATATATGGGCCTGCACACAGTGGACGAGAAACAGTATATTCCAATGCATCCCTGCTGATCCAGAATGTCACCCGGGAGGACGCAGGATCCTACACCTTACACATCATAAAGGGAGGTGATGGGACTAGAGGAGTAACTGGAAATTTCACCTTCACCTTATACC

>Pab_PSG9N (Pongo abelii; Sumatran orangutan) XM_009232678.1

CATCACTTTTAAACTTCTGGAACCCACCCACCACTGCCCAAGTCACGATTGAAGCCCAGCCACCCAAAGTTTCCGAGGGGAAGGATGTTCTTCTACTTGTCCACAATTTGCCCCAGAATCTTGCTGGCTACATCTGGTACAAAGGGCAAACGAGGGACCTCAACCATTACATTACATCATATGTTGCAGACAGTAAAATAATTATACATGGGCCTGCACACAGTGGACGAGAAACAGTATATTCCAATGCATCCCTGCTGATCCAGAATGTCACCCGGGAGGACGCAGGATCCTACACCTTACACATCATAAAGCGAGGTGATGGGATTAGAGGAATAACTGGACATTTCACCTTCACCTTATACC

>Pab_PSG10N (Pongo abelii; Sumatran orangutan) XR_002912494.1

CATCACTTTTAAACTTCTGGAACCTGCCCATGACTGCCCAAGTCACGATTGAAGCCCAGCCACCCAAAGTTTCTGAGGGGAAGGATGTTCTTCTACTTGTCCACAATTTACCCCAGAATCTTACTGGCTACATCTGGTACAAAAGGCAAATGAAGGACCTCTACCATTACATTACATCATATGTAGTAGACGGTCAAATAATTATATATGGGCCTGCATACAGTGGACGAGAAACAGTATATTCCAATGCATCCCTGCTGATCCAGAATGTCACCCGGGAGGACGCAGGATCCTACACCTTACACATCATAAAGGGAGGTGATGGGACTAGAAGAGAAACTGGAAATTTCACCTTCACCTTATACC

>Pab_PSGP1N (Pongo abelii; orangutan) WGS ABGA01097053.1

CATCACTTTTAAACTTCTGGAACCTGCCAATCACTGCCTAAGTGACCATTGAAGCCCAGCCACCCAAAGTTTCTGAGGGGAAGGATGTTCTTCTACTTGTCCACAATTTGCCCCAGAATCTTGCTGGCTACATCTGGTACAAAGGGCAAATGACGGACCTCTACCATTACATAACATCATATGCAGTAGACAGTCAAATAAATATATATGGGCCTGCATACAGTGGACAAGAAACAGTGTATTCCAATGCATCCCTGCTGATCCAGAATGTCACCAGGGAGGACACAGGATACCATACCTTACACATGATAAAGCGAGGTGATAGGACTAGAGGAGTAACTGGACATTTCACCTTCACCTTATACT

>Pan_PSG1N (Papio anubis; olive baboon) WGS AHZZ02014419.1

CATCACTTTTAATCTTCTGGAACCCGCCCACCACTGCTCAAGTCACAATTGAAGCCCAGCCAACCAAAGTTTCTGAGGGGAAGGATGTTCTTCTACTTGTCCACAATTTGCCCCAGAATCTTGCTGCCTGCATCTGGTACAAAGGGCAAATAATGGACCTCCAACATTACATTACAGCATATGTAATAGATGCTGAAACAATTATATTTGGGCCTGCATACAGTGGACGAAAAACAGTATATTCCAATGCATCCCTGCTGATCCAGAATGTCACCCAGAAGGACACAGGATCCTACACCATACAAATCATACAGCGAGGTGATAAGACTAAAGGAGTAACTGGACATTTCACCTTATATG

>Pan_PSG2N (Papio anubis; olive baboon) WGS AHZZ02014436.1

CATCACTTTTAATCTTCTGGAACCCGCCCACCACTGCCCAAGTCACGATTGAAGCTCAGCCAACCAAAGTTTCCGAGGGGAAGGATGTTCTTCTACTTGTCCACAATTTGCCCACGAACGTTGTTGGCTACGTCTGGTACAAAGGGCAAATAATGGACCTCCAGCATTACATTACAGCATATACAACAGACACTGAAATGATTATATTTGGGCCTGAATACAGTGGACGAGAAACAGTATATTCCAATGCATCCCTGCTGATCCAGAGTGTCACCAAGAATGACACAGGATCCTACACCATACAAATCATAAAGCGAGGTCATAGGACTGAAGGAGAAACTGGACATTACACCTTATACC

>Pan_PSG3N (Papio anubis; olive baboon) WGS AHZZ02115059.1

CATCACTTTTAATCTTCTGGAACACGCCCACCACTGCCCAAGTCACGATTGAAGCCCAGCCAACCAAAGTTTCTGAGGGGAAGGATGTTCTGCTACTTGTCCACAATTTGCCCCAGAATCCTACTGGCTACATCTGGTACAAAGGGCAAATAATGGACCTCCATCATCACATTACATCATATGTAATAGACACTGAAATAATTGTATTTGGGCCTGCATACAGTGGACGAGAAACAGTATATTCCAATGCATCCCTGCTGATCCAGAATGTCACCCAGAAGGACACAGGATCCTACACCATACAAATCATACAGCGAGGTGATAGCACTAAAGGAGTAACTGGACATTTCACCTTATACC

>Pan_PSG4N (Papio anubis; olive baboon) WGS AHZZ02014422.1

CATCACTTTTAATCTTCTGGAACCCGCCCACCACTGCCCAAGTCACGATTGAAGCCCAGCCAACCAAAGTTTCTGAGGGGAAGGATGTTCTGCTACTTGTCCACAATTTGCCCCAGAATCCTACTGGCTACATCTGGTACAAAGGGCAAATAATGGACCTCCACCATCACATTACATCATATGTAAAAGACACTGAAACAGTTATAGCTGGGCCTGCATACAGTGGACGAGAAACAGTATATTCCAATGCATCCCTGCTGATCCAGAATGTCACCCAGAAGGACACAGGATCCTACACCATACAAATCATACAGCGAGGTGATACCACTAAAGGAGTAACTGGACATTTCACCTTATACG

>Pan_PSG5N (Papio anubis; olive baboon) WGS AHZZ02113263.1

CATCACTTTTAATCTTCTGGAACCTGCCCACCACTGCCCAAGTCACGATTGAAGCACAGCCAACCAAAGTTTCTGAGGGGAAGGATGTTCTGCTACTTGTCCACAATTTGCCCCAGAATGTTGCTGGCTACAGCTGGTACAAAGGGCAAATAATGGACCTCCACCATTACATTACAGCATATACAATAGACACTGAAATGATTATAGTTGGGCCTGCATACAGTGGACGAGAAACAGTATATTCCAATGCATCCCTGCTGATCCAGAATGTCACCCGGAAGGACACAGGATCCTACACCATACAAATCATAAAGAGAGGTGATGGGACTAAAAGAGTAACTGGACATTTCACTTTATACC

>Pan_PSG6N (Papio anubis; olive baboon) WGS AHZZ02039643.1

CATCACTTTTAATCTTCTGGAACCCGCTCACCGCTGCTCAAATCACGATTGAAGCCCAGCCAAACAAAGTTTCCGAGGGGAAGGATGTTCTTCTACTTGTCCACAATTTGCCCCAGAATCTTACTGGCTACATCTGGTACAAAGGGCAAAAAACGGACCACCACCATTACATTACATCATATGTGATAGACACTGAAACAATTATATTTGGGCCTGCATACAGTGAACGAGAAACAGTATATTCCAATGCATCCCTGCTGATCCAGAATGTCACCAAGAATGACACAGGATCCTACACCATACAAATCATAAAGCGAGGTGATAGGACTGAAGGAGTAACTGGACATTACACCTTATCCC

>Pan_PSG7N (Papio anubis; olive baboon) WGS AHZZ02095637.1

CATCACTTTTAATCTTCTGGAACCCGCCCACCACTGCCCAAGTCACGATTGAAGCTCAGCCAAACAAAGTTTCTGAGGGGAAGGATGTTCTGCTACTTGTCCACAATTTGCCCCAGAATCTTACTGGCTACAGCTGGTACAAAGGGCAAATAATGGACCTCCACCATTACATTGCAGCATATACAACAGACACTGAAATGATTATATTTGGGCCTGAATACAGTGGACGAGAAACAGTATATTCCAATGCATCCCTGCTGATCCAGAATGTCACCAAGAATGACACAGGATCCTACACCATTCAAATCACAAAGCGAGGTGATGAGACTAAAGGAGTAACTGGACATTTCACCTTATACC

>Pan_PSG8N (Papio anubis; olive baboon) WGS AHZZ02113027.1

CATCACTTTTAATCTTCTGGAACCCACCCACCACTGCTCAAGTCACAATTGAAGCCCAGCCAACCAAAGTTTCCGAGGGGAAGGATGTTCTTCTACTTGTCCACAATTTGCCCCAGAATCTTACTGGCTACATCTGGTACAAAGGGCAAAAAACGGACCTCCACCTTTACGTTACATCATATGTAAAAGACACTGAAACAGTTATAGCTGGGCCTGCATACAGTGGACGAGAAACAGTATATTCCAATGCATCCCTGCTGATCCAGAATGTCACCCAGAAGGACACAGGATCCTACACCATAGAAATCACAAAGCAAGGTGATAGGACTGAAGGAGAAACTGGACATTACACCTTATATC

>Pan_PSG9N (Papio anubis; olive baboon) WGS AHZZ02104779.1

CATCACTTTTAATCTTCTGGAACCCACCCACCACGGCTCAAGTCACGATTGAAGCCCAGCCAACCAAAGTATCCGAGGGGAAGGATGTTCTTCTACTTGTCCACAATTTGCCCCAGAATCTTACTGGCTACATCTGGTACAAAGGGCAAAAAACGGACCACCACCATTACATTACATCATATGTGATAGACACTGAAACAATTATATTTGGGCCTGCATACAGTGAACGAGAAACAGTATATTCCAATGCATCCCTGCTGATCCAGAAGGTCACCAAGAATGACACAGGATCCTACACCATACAAATCATAAAGCGAGGTGATAGGACTGAAGGAGTAACTGGACATTACACCTTATACC

>Pan_PSG10N (Papio anubis; olive baboon)

CATCACTTTTAATCTTCTGGAACTCGCCCACCACTGCCCAAGTCACAATTGAAGCTCAGCCAACCAATATTTCCGAGGGGAACGATGTTCTTCTACTTGTACACAATTTGCCCCAGAATCCTGCTGCCTACATCTGGTACAAAGGGCAAATAATGGACCTCCACCATTACATTACAGCATATACAATAGACACTGAAAGAATTATATTTGGGCCTGCATACAGTGGACGAGAAAGAGTATATTCCAATGCGTCCCTGCTGATCCAGAGTGTGAACCAGAAGGACGCAGGATCCTGCACCATACAAATCATAAAGCGAGGTGACGGGACTGAAGGAGTAACTGGACATTTCACCTTATATG

>Pan_PSG11N (Papio anubis; olive baboon) WGS VSMJ01002522.1

CATCACTTTTCATCTTCTGGAACCCACCCACCACTGCCCAAGTCACGATTGAAGCTCAGCCAACCAAAGTTTCCGAGGGGAAGGATGTTCTTCTACTTGTCCAGAATTTGCCCCAGAATCTTACTGGCTACGTCTGGTACAAAGGGCAAAAAACGGACCTCCACCAATTCATTACAGCATATACGATAGACACTGAAACAATTATATCTGGGCCTGCATACAGTGGACGAGAAACAGTATATTCCAATGCATCCCTGCTGATCCAGAATGTCACCCGGAATGACACAGGATCCTACACCGTACAAATCATACAGCGAGGTGATAAGATTAAAAGAATAACTGGACATTTCACCTTATACC

>Pan_PSG12N (Papio anubis; olive baboon) WGS VSMJ01008629.1

CATCACTTTTAATCTTCTGGAACCCGCCCACCACTGCCCAAGTCACGATTGAAGCTCAGCCAACCAAAGTTTCTGAGGGGAACGATGTTCTTCTACTTGTCCACAATTTGCCCACGAACGTTGTTGGCTACATCTGGTACAAAGGGCAAATAATGGACCTCCAGCATTACATTACAGCATATACAACAGACACTGAAATGATTATATTTGGGCCTGCATACAGTGGACGAGAAACAGTATATTCCAATGCATCCCTGTTGATCCAGAGTGTCACCAAGAATGACACAGGATCCTACACCATTCAAATCATAAAGCGAGGTCATAGGACTGAAGGAGTAACTGGACATTACACCTTATACC

>Pan_PSG13N (Papio anubis; olive baboon) WGS AHZZ02088387.1

CATCACTTTTAATCTTCTGGAACCCGCCCACCACTGCCCAAGTCACGATTGAAGCTCAGCCAACCAAAGTTTCCGAGGGGAAGGATGTTCTTCTACTTGTCCACAATTTGCCCACGAACGTTGTTGGCTACGTCTGGTACAAAGGGCAAATAATGGACCTCCAGCATTACATTACAGCATATACAACAGACACTGAAATGATTATATTTGGGCCTGAATACAGTGGACGAGAAACAGTATATTCCAATGCATCCCTGCTGATCCAGAGTGTCACCAAGAATGACACAGGATCCTACACCATACAAATCATAAAGCGGGGTCATAGGACTGAAGGAGTAACTGGACATTACACCTTATACC

>Pan_PSG14N (Papio anubis; olive baboon) WGS AHZZ02108982.1

CATCACTTTTAATCTTCTGGAACCCGCCCACCACTGCCGAAGTCACGATTGAAGCTCAGCCAACTAAAGTTTCCGAGGGGAAGGATGTTCTTCTACTTGTCCACAATTTGCCCACGAACGTTGTTGGCTACGTCTGGTACAAAGGGCAAATAATGGACCTCCAGCATTACATTACAGCATATACAACAGACACTGAAATGATTATATTTGGGCCTGAATACAGTGGACGAGAAACAGTATATTCCAATGCATCCCTGCTGATCCAGAGTGTCACCAAGAATGACACAGGATCCTACACCATACAAATCATAAAGCGAGGTCATAGGACTGAAGGAGTAACTGGACATTACACCTTATACC

>Pan_PSG15N (Papio anubis; olive baboon) WGS VSMJ01002484.1

CATCACTTTTAATCTTCTGGAACCCGCCTACCACTGCCCAAGTCACGATTGAAGCTCAGCCAACCAAAGTTTCTGAGGGGAAGGATGTTCTTCTACTTGTCCAGAATTTGCCCCAGAATCTTACTGGCTACGTCTGGTTCAAAGGGCAAAAAACGAACTTCCACCAATTCATTATAGCATATAAAATAGACAGTAAAAAAATTACAATTGGGCCTGCATACAGTGGACGAGAAAGAGTATATTCCAATGCATCCCTGCTGATCCAGAATGTCACCCGGAAGGACACAGGATCCTATACCATACAAATCATACAGCGAGGTGATAAGATTACAGGGGTAACTGGACATTTCACCTTATACG

>Pan_PSG16N (Papio anubis; olive baboon) WGS VSMJ01000463.1

CATCATTTTTAATCTTCTGGAACCCGCCCACCACTGCCCAAGTCATGATTGAAGCTCAGCCAACCAAAGTTTCTGAGGGGAAGGATGTTCTTCTACCTGTCCGCAATTTGCCCCAGGAAGTTGCTGCCTATATCTGGTACAAAGGGCAAATAATGGACTTCCACCAATTCATTACAGCATATACAATAGACACTGAAAGAATTATATTTGGGCCTGCATACAGTGGACGAGAAACAGTATATTCCAATGGATCCCTGCTGATCCGGAATGTCACCAAGAATGACACAGGATCCTACACCGTAAAAATCAGGAAGCCAGCTGAGGAGACTAAAGGAGTAACTGTACATTTCACCTTATATC

>Pan_PSG17N (Papio anubis; olive baboon) EY277292.1

CGTCACTTTTCATCTTCTGGAACCCACCTATCGCGGCCCAAGTCACGATTGAAGCTCAGCCAACCGAAGTTTCTGAGGGGAAGGATGTTCTTCTACTTGTCCACAATTTGCCCCAGAATCCTACTGGCTACATCTGGTACAAAGGGCAAATAACGGATAGCCACAATTACATTACATCATATGTAATAGACACTGAAATGATTATATTTAGGCCTGCATACAGTGGACGAGAAACAGTATATTCCAATGCATCCCTGCTGATCCAGAATGTCACCCGGAAGGACACAGGATCCTACACCATACAGATCATAAAGCGAGGTGATAGCACTAAAGGAGTAACTGGACATTTCACCTTATACC

>Pan_PSG18N (Papio anubis; olive baboon) WGS AHZZ02095895.1

CATCACTTTTAATCTTCTGGAACCCGCCCACCACTGCCCAAGTCACAATTGAAGCTCAGCCAACCAAAGTTTCCGAGGGGAAGGATGTTCTTCTACTTGTCCAGAATTTGCCCCAGAATCTTACTGGCTACGTCTGGTTCAAAGGGCAAATAACGAACTTCCACCAATTCATTATAGCATATAAAATAGACAGTAAAAAAATTACAGTTGGGCCTGCATACAGTGGACGAGAAAGAGTATATTCCAATGCATCCCTGCTGATCCAGAATGTCACCCGGAAGGACACAGGATCCTACACCATAGAAATTATAAAGCGAGGTGATAAGATTAAAGGGGTAACTGGACATTTCACCTTATACG

>Pan_PSGP1N (Papio anubis; olive baboon) WGS VSMJ01000174.1

CATCACTTTTAATCTTCTGGAACCCACCCACCACTGCTCAAGTCACAATTGAAGCCCAGCCAACCAAAGTTTCCGAGGGGAACGATGTTCTTCTACTTGTCCACAATTTGCCCCAGAATCTTACTGGCTACATCTGATACAAAGGGCAAAAAACGGACCTCCACCTTTACGTTACATCATATGTAATAGACGCTGAAACAATTATATTTGGGCCTGCATACAGTGGACGAGAAACAGTATATTCCAATGCATCCCTGCTGATCCAGAATGTCACCCGGAAGGACACAGGATCCTACACCATAGAAATCATAAAGCGAGGTGATAGGACTGAAGGAGAAACTGGACATTACACCTTATACC

>Pan_PSGP2N (Papio anubis; olive baboon) WGS AHZZ02014447.1

CATCACTTTTAATCTTCTGGAACCCGCCCACCATGCCCATGTCACGATTGAAGCTCAGCCAACCAAAGTTTCCGAGGGGAAGGATGTTCTTCTACTTGTCCACAATTTGCCCCAGAATCTTACTGGCTACAGCTGGTACAAAGGGCAAATAATGGACCTCCAGCATTACATTACAGCATATACAATAGACACTGAAATGATTGTATTTGGGCCTGCATACAGTGGACGAGAAACAGTATATTCCAATGCATCCCTGCTGATCCAGAATGTCACCAAGAATGACACAGGATCCTACACCATTCAAATCACAAAGCGAGGTGATGAGACTAAAGGAGTAACTGGACATTTCACCTTATACC

>Pan_PSGP3N (Papio anubis; olive baboon) WGS VSMJ01001414.1

CATCATTTTTAATCTTCTGGAACCCACCCACCACTGCCCAAGTCATGATTGAAGCTCAGCCAACCAAAGTTTCCGAGGGGAAGGATGTTCTTCTACTTGTCCGCAATTTGCCCCAGAAAGTTGCTGCCTATGTCTGGTACAAAGGGCAAATAATGGACTTCCACCAATTCATTACAGCATATACAATAGACCCTGAAAGAATTATATTTGGGTATGCATACAGTGGACGAGAAACGCTATATTCCAATGGATCCCTGCTGATCCGGAATGTTACCAAGCAGGACACAGGATCCTACACCGTAAAAATCATGAATCGAATGGAGGAGATTAAGGAGTAACTGTACATTTCACCTTATACC

>Pdo_PSG1N (Plecturocebus donacophilus; Bolivian titi) WGS PVKP010058365.1

CATCACTTTTAAACTTCTGGAACCCACCCACCACTGCCCAAGTCACGATTGAAGCCCAGCCACACATTGTTTCAGAGGGAAAAGATGTTCTTCTACTTGTCCACAATTTGCCCCAGAATCTTACTGGCTACAGCTGGTACAGAGGGAAAGTGATGGACATCCACCATTACCTTACAGCATATTTAATAGAAAAAAAAATAATTATATATGGGCATGCATACAGTGGACGAGAAACAATATATTCCAATGCATCCCTGCTGATCCAGAACGTCACCCTGAACGACACAGGATCCTACACCCTGCAAGTCGTCAATCAAGGTGAAAGGAATAAAGGAGTAACTGCACATTTCACCTTACACC

>Pdo_PSG2N (Plecturocebus donacophilus; Bolivian titi) WGS PVKP010108745.1

CATCACTTTTAAACTTCTGGAACCCACCCACCACTGCCCAAGTCAGGATTGAAGCCCAGCCACACATTGTTTCAGAGGGGAAGGATGTTCTTCTACTTGTTCGCAATTTGCCCCAGAATATTATTGGCTACAGCTGGTACAGAGGGAAAGTGATGGACATCCAACATTACATTACAGCATATTTAATAGGCACTCAAGTAATTATATATGGGCCTGCATACAGTGGACGAGAAACATTATATTCCAATGCATCCCTGCTGATCCAGAACGTCACCCAGAATGACGCAGGATCCTACACCATGAAAGTCATCAATCAAGGTGAAAGAAATAAAGGAGTAACTGCACATTTCACCTTACACT

>Pdo_PSG3N (Plecturocebus donacophilus; Bolivian titi) WGS PVKP010148516.1 CATTACTTTTAAACTTCTGGAACCCACCCACCACTGCCCAAGTCACGATTGAAGCCCAGCCACACATTGTTTCAGAGGGGAAGGATGTTCTTCTACTTGTCCACAATTTGCCCCAGAATCTTACTGGCTACAGCTGGTACAGAGGGAAAGTGATGGACATCCACCATTACATTACAGCATATTTAATAGAAAAAGAAATAATTATATATGGGCATGCATACAGTGGACGAGAAACAATATATTCCAATGCATCCCTGTTGATCCAGAACGTCACCCTGAATGACACAGGATCCTACACCCTGCAAGTCGTCAATCAAGGTGAAAGGAATAAAGGAGTAACTGCACATTTCACCTTACACC

>Pne_PSG1N (Pygathrix nemaeus; Red shanked douc langur) WGS PVHW010046110.1

CATCACTTTTAATCTTCTGGAACCCACCTACCAGTGCCCAAGTCACGATTGAAGCCCAGCCAACCAAAGTTTCTGAGGGGAAGGATGTTCTGCTACTTGTCCACAATTTGCCCCAGAATGTTACTGGCTACGTCTGGTACAAAGGGCAAATAATGGACCTCTACCATTACATTACAGCATATATAATAGACACTGAAATGATTATAGTTGGGCCTGCATACAGTGGACGAGAAACTGTATATTCCAATGCATCCCTGCTGATCCAGAATGTCACCCAGAATGACACAGGATCCTACATGATACAAATCACACAGCGAGGTGATGAGACTAAAGGAGTAACTGGACATTTCACCTTATACC

>Pne_PSG2N (Pygathrix nemaeus; Red shanked douc langur) WGS PVHW010095233.1

CATCACTTTTAATCTTCTGGAACCCGCCCACCACTGCCCAAGTCACGATTGAAGCCCAGCCAACCAAAGTTTCTGAGGGGAAGGATGTTCTGCTACTTGTTCACAATTTGCCCCAGAATATTGCTGCCTATATCTGGTACAAAGGGCAAATAATGGACCTCCGCCATTACATTACAGCATATATAATAGACACTGAAATGATTATAGTTGGGCCTGCATACAGTGGACGAGAAACTGTATATTCCAATGCATCCCTGCTGATCCAGAATGTCACCCAGAATGACACAGGATCCTACACCATTCAAATCACACAACGAGGTGATGGAACTAAAGGAGTAACTGGACATTTCACCTTATACC

>Pne_PSG3N (Pygathrix nemaeus; Red shanked douc langur) WGS PVHW010025071.1

CATCACTTTTAATCTTCTGGAACACGCCCACCACTGCCCAGGTCACGATTGAAGCCCAGCCAACCAAAGTTTCCGAGGGGAAGGATGTTCTGCTACTTGTCCACAACTTGCCCCAGAATCTTATTGGCTACATCTGGTACAAAGGGCAAATAATGGACTTCCACCATTACGTTACATCATATGTAATAGATGCTGAAACAACTATATTTGGGCCTGCATACAGTGGACGAGAAACTGTATATTCCAATGCATCCCTGCTGATCCAGAATGTCACCCGGAAGGACACAGGATCCTACACCATACAAATCATAAAGCAAGGTGATAAGACTAAAGGAGTAACTGGACATTTCACCTTATACC

>Pne_PSG4N (Pygathrix nemaeus; Red shanked douc langur) WGS PVHW010061428.1

CATCACTTTTAATCTTCTGGAACCCACCCACCACTGCCCAAGTCACGATTGAAGCCCAGCCAACCAAAGTTTCTGAGGGGAAGGATGTTCTTCTACTTGTCCACAATTTGCCCCAGAATGTTACTGGCTACGTCTGGTACAAAGGGCAAATAATGGACCTCTACCATTACATTACAGCATATATAATAGACACTGAAATGATTATATTTGGGCCTGCATACAGTGGACGAGAAACAGTATATTCCAATGCATCCCTGCTGATCCAGAATGTCACCCAGAATGACACAGGATCCTACATGATACAAATCACACAGCGAGGTGATAAGACTAAAGGAGTAACTGGGCATTACTCCTTATACC

>Pne_PSG5N (Pygathrix nemaeus; Red shanked douc langur) WGS PVHW010020878.1

CATCACTTTTAATCTTCTGGAACCCGCCCACCACTGCGCAAGTCACGATTGAAGCCCAGCCAACAAAAGTTTCTGAGGGGAAGGATGTTCTGCTACTTGTCCACAATTTGCCCCAGAATCTTGCTGGATACATCTGGTACAAAGGGAAAATAATGGACCTCTACCATTACATTACAGCATATACAATAGACACTGAAATGATTATATTTGGGCCTGCATACAGTGGACGAGAAACAGTATATTCCAATGCATCCCTGTTGATCCAGAATGTCACCCAAAATGACACAGGATCCTACACCATACAAATCATAAAGCGAGGTGATAAGACTAAAGGAGTAACAGGATATTTCACCTTATACC

>Pne_PSG6N (Pygathrix nemaeus; Red shanked douc langur) WGS PVHW010070328.1

CATCACTTTTAATCTTCTGGAACCCGCCCACCACTGCCCAAGTCACGATTGAAGCCCAGCCAACCAAAGTTTCTGAGGGGAAGGATGTTCTGCTACTTGTCCACAATTTGCCCCAGAATGTTACTGGCTACGTCTGGTACAAAGGGCAAATAATGGACCTCTACCATTACATTACGGCATATGTAATAGACACTGAAATGATTATATTTGGGCCTGCATACAGTGGACGAGAAACTGTATATTCCAATGCATCCCTGCTGATCCAGAGTGTCACCCAGAATGACACAGGATCCTACACCATTCAAATCGCACAACGAGGTGATGGAACTAAAGGAGTAACAGGACATTTCACCTTATACC

>Pne_PSG7N (Pygathrix nemaeus; Red shanked douc langur) WGS PVHW010011426.1

CATCACTTTTAATCTTCTGGAACACGCCCACCACTGCCCAAGTCATGATTGAAGCCCAGCCAACCAAAGTTTCTGAGGGGAAGGATGTTCTTCTACTTGTCCACAATTTGCCCCAGAATCTTGCTGCCTACATCTGGTACAAAGGGCAAATAATGGACCTCCACCATTACATTACAGCATATGTAATAGACACTGATACAATTATATTTGGGCCTGCATACAGTGGACGAGAAACAGTATATTCCAATGCATCCCTGCTGATCCAGAATGTCACCCAGAAGGACACAGGATCCTACACCATACAAATCATAAAGCGAGGTGATAGGACTGAAGCAGTAACTGGACATTTCACCTTATACC

>Pne_PSG8N (Pygathrix nemaeus; Red shanked douc langur) WGS PVHW010009276.1

CACCACTTTTAATCTTCTGGAACCCGCCCACCACTGCTCAAGTCACGATTGAAGCCCAGCCAACCAAAGTTTCTGAGGGGAAGGATGTTCTTCTACTTGTCCACAATTTGCCCCAGAATCTTACTGGCTACGTCTGGTACAAAGGGCAAATAATGGACTACCACCATTACATTACAGCATATGTAATAGACCCTGAAACAATTATATTTGGGCCTGCATACAGTGGACGAGAAACAGTATATTCCAATGCATCCCTGCTGATCCAGAATGTCACCCGGAAGGACACAGGATCCTACACCATACAAATCATAAAGCGAGGTGATAGCACTAAAGGAGTAACTGGACATTTCACCTTATACC

>Pne_PSG9N (Pygathrix nemaeus; Red shanked douc langur) WGS PVHW010021070.1

CATCACTTTTAATCTTCTGGAATGCGCCCACCACTGCCCAAGTCACGACTGAAGCTCAGCCAACCAAAGTCTCTGAGGGGAAGGATGTTCTTCTGCTTGTCCACAATTTGCCCCAGAATGTTGTTGGTTACATCTGGTACAAAGGGCAAATAATGGACCTCCACCATTACATTACAGCATATACAATAGACACTGAAACAATTATATTTGGGCCTGTATACAGTGGACGAGAAAAAATATATTCCAATGCATCCCTGCTGATCCAGAATGTCACCCAAAATGACACAGGATCCTACACCATACAAATCATAAAGCGAGGTGATGGAACTAAAGGAGTAACTGGACATTTCACCTTATACC

>Pne_PSG10N (Pygathrix nemaeus; Red shanked douc langur) WGS PVHW010036886.1

CATCACTTTTAATCTTCTGGAACACGCCCACCACTGCCCAAGTCACGATTGAAGCACAGCCAACCAAAATATCTGAGGGGAAGGATGTTCTGCTACTTGTCCACAACTTGCCCCAGAATCTTATTGGCTACATCTGGTACAAAGGCCAAGTAATGGACTTCCACCATTACATTACATCATATGTAATAGATGCTGAAACAATTATAATTGGGCCTGCATACAGTGGACGAGAAACAGTATATTCCAATGCATCTCTGCTGATCCAGAATGTCACCCGGAAGGACACAGGATCCTACACCATACAAATCATAAAGCTAGGTGATAGGACTAAAGGAGTAACTGGACATTTCACCTTATACT

>Pne_PSG11N (Pygathrix nemaeus; Red shanked douc langur) WGS PVHW010035154.1

CATCACTTTTAATCTTCTGGAACCCGCCTACCATGGCCCACGTCATGATTGAAGCTCAGCCAAACGAAATTTCTGAGGGGAAGGATGTTCTTCTACTTGTCCACAATTTGCCCCAGAATCCTACTGGCTACATCTGGTACAAAGGGCAAATAACGGATATCCACAATTACATTACATCATATGTAATAGACACTGATACAATTATATTTGGGCCTGCATACAGTGGACGAGAAACAGTATATTCCAATGCATCCCTGCTGATCCAGAATGTCACCCAGAAGGACACAGGATCCTACACCATACAAATCATAAAGCGAGGTGATAGCACTAAAGGAGTAACTGGACATTTCACCTTATACT

>Pne_PSG12N (Pygathrix nemaeus; Red shanked douc langur) WGS PVHW010012893.1

TATTACTTTTCATCTTCTGGAACCCGCCCATCACTGCCCAAGTCACGATTGAAGCTCAGCCAACCAAAGTTTCCGAGGGGAAGAGTGTTCTTCTACTTGTCCACAATTTGCCCCAGAATCTTACTGGCTACATCTGGTACAAAGGGCAAATAATGGACCTCCACCAATTCATTACAGCATATACAATAGACACTGAAACAATTATATCTGGGCCTGCATACAGTGGACGAGAAACAGTATATTCCAATGCATCCCTGCTGATCCAGAATGTCACCCAGAATGACACAGGATCCTACACGATACAAATCATAAAGCGAGGTGAAAAGATTAAACGAGTAACTGGACATTTCACCTTATACC

>Pne_PSG13N (Pygathrix nemaeus; Red shanked douc langur) WGS PVHW010036503.1

CATCACTTTTAATCTTCTGGAACCCGCCTACCATGGCCGACGTCATGATTGAAGCTCAGCCGACCGAAGTTTCTGAGGGGAAGGATGTTCTTCTACTTGTCCACAATTTGCCCCAGAATCCTACTGGCTACATCTGGTACAAAGGGCAAATAACGGATATCCACAATTACATTACATCATATGTAATAGACACCGATACAATTATATTTGGGCCTGCATACAGTGGACGAGAAACAGTATATTCCAATGCATCCCTGCTGATCCAGAATGTCACCCAGAAGGACACAGGATCCTACACCATACAAATCATAAAGCGAGGTGATAGCACTAAAGGAGTAACTGGACATTTCACCTTATACC

>Pne_PSG14N (Pygathrix nemaeus; Red shanked douc langur) WGS PVHW010035176.1

CATCACTTTTAATCTTCTGGAACGCGCCCACCACTGCCCAAGTCACAATTGAAGCTCATCCAACCAATGTTTCTGAGGGGAATGATGTTCTTCTACTTGTTCACAATTTGCCCCAGAATCCTGCTGCCTACATCTGGTACAAAGGGCAAATAATGGATCTCCACCATTACATTACAGCATATGTAATAGAAACTGAAAGAATTGTATTTGGGCCTGCATACAGTGGACGAGAAACAGTATATTCCAATGCATCCCTGCTGATCCAGAGTGTCAACCAGAAGGACGCAGGATCCTACACCGTAGAAATCATAAAGCGAGGTGATGGGAATGAAGGAGTAACTGGAAATTTCACCTTATACC

>Pne_PSG15N (Pygathrix nemaeus; Red shanked douc langur) WGS PVHW010022911.1

CATCACTTTTAATCTTCTGGAACCTGCCCATCGCTGCCCAAGTCATGATTGAAGCCCAGCCAACCAAAGTTTCCGAGGGGAAGGATGTTCTTCTGCTTGTTCACAATTTGCCCCAGAATGTTGCTGCCTACATCTGGTACAAAGGGCAAATAGGGGACCTCCACCATTACATTATGCGATATGTAATAGAGGCTGAAGCAATTTTATTTGGGCCTGCATACAGTGGACGAGAAACAGTATATTCCAATGCATCCCTGCTGATTCAGAATGTCACCCAGAAGGACACAGGATCCTACACCATACAAATCATAAAGCGAGGTGATAAGACTAAAGGAGTAACTGGACATTTCACGTTATACT

>Pne_PSG16N (Pygathrix nemaeus; Red shanked douc langur) WGS PVHW010018997.1

CATCACTTTTAATCTTCTGGAACCCGCCCATCGCTGCCCAAGTCATAATTGAAGCCCAGCCAACCAAAGTTTCCGAGGGGAAGGATGTTCTTCTACTTGTTCACAATTTGCCCCAGAATGTAGCTGCCTACATCTGGTACAAAGGGCAAATAACGGACGTCCACCATTACATTACGGGTTATGTAATAGACCCTGAAACAATTATATTTGGGCCTGCATACACTGGACGAGAAAGACTATATTCCAACGCATCCCTGCTGATTCAGAAAGTCACCCAGAAGGACACAGGATCCTACACCATAAAAATCACAAAGCGAGGTGATAAGACTAAAGGAGTAACTGGACATTTCACCTTATACT

>Pne_PSG17N (Pygathrix nemaeus; Red shanked douc langur) WGS PVHW010025409.1

CATCACTTTTAATCTTCTGGAACCCGCCCACCACTGCCCAAGTCATGATTGAAGCTCAACCAACCAAAGTTTCTGAGGGGAAGGATGTTCTTCTACTTGTCCACAATTTGCCCCAGAATCCTGCTGCCTACGTCTGGTACAAAGGGCAAATAATGGACTTCTACCAATTCATTATAGCATATACAAGATACCCTGATAGAATTCTATTTGGGCCTGCATACAGTGGACGAGAAACACTATATTCCAATGGATCCCTGCTGATCCAGAATGTCACCAAGCAGGACACAGGATCCTACACCGTAAAAATCATGAAGCGAATTGATGAGACTAAAGGAGTAACTGGACATTTCACCTTATACT

>Pne_PSG18N (Pygathrix nemaeus; Red shanked douc langur) WGS PVHW010032342.1

CATCACTTTTAATCTTCTGGAACCCGCCCACCACTGCCCAAGTCATGATTGAAGCTCAACCAACCAAAGTTTCTGAGGGGAAGGATGTTCTTCTACTTGTCCACAATTTGCCCCGGAATGTTGCTGCCTACGTGTGGTACAAAGGGCAAATAATGGATTTCTACCAATTTATTACAGCATATTCAAGAGACCCTGATAGAATTCTATTTGGGCCTGCATACAGTGGACGAGAAACACTATATTCCAATGGATCCCTGCGGATCCAGAATGTCACCAAGCAGGACACAGGATCCTACACTGTAAAAGTCATGAAGCGAATTGATGAGACTAAAGGAGTAACTGGACATTTCACCTTATACC

>Pne_PSGP1N (Pygathrix nemaeus; Red shanked douc langur) WGS PVHW010021750.1 no ORF

CATCACTTTTAATCTTCTGGAACCCGCCCACCACTGCCCAAGTCATGATTGAAGCCCAGCCAAACAAAGTTTCTGAGGGGAAGCATACTCTTCTACTTGTCCAGAATTAGCCCCAGAATCTTACTGGCTACATCTGGTACAAAGGGCAAATAATGGACCTCCACCAATTCATTACAGCATATACAATAGACACTGAAACAATTATATTTGGGCCTGCATACAGTGGACGAGAAACAGTATATTCCAATGCATCCCTGCTGATCCAGAATGTCACCCAAAATGACACAGGATCCTACACCATACAAATCATAAAGCGAGGTGATAAGATTAAAGGAGTAACTGGACATTTCACCTTATACC

>Pne_PSGP2N (Pygathrix nemaeus; Red shanked douc langur) WGS PVHW010022783.1 no ORF

CATCACTTTAAATCTTCTGGAACTCGCCCACCATGGCCCAAGTCACGACTGAAGCCTAGCCAACCAAAGTTTCTGAGGGGAAGGATGTTCTGCTACTTGTCTACAATTTGCCCCAGAATGTTGCTGCCTACATCTGGTACAAAGAGCAAATAATAGACCTCCACCATTACATTACAGCATATACAATAGACACTGAAATGATTATATTTGGGCCTGCATACAGTGGATGAGAAACTGTATATCCCAACGCATCCCTGTTGATCCAGAATGTCACCCAGAAGGACACAGGATCCTACACCATTCAAATCACACAGCGAGGTGATGGAACTAAAGGAGTAACTGGACATTTCACCTTATACC

>Pne_PSGP3N (Pygathrix nemaeus; Red shanked douc langur) WGS PVHW010028652.1 no ORF

CATTACTTTTAATCTTCTGAAACCTGCGCACCACTGCTCAAGTCATGATTGAAGCCCAGCCAACCAAAGTTTCTGAGGGGAAGGATGTTCTTCTACTTGTCCACAATTTGCCCCAGAATCTTACTGGCTACATCTGGTACAAAGGGCAAATGATGGACCTCCACCATTACATTACAGCATATACAATAGACACTGAAACAACTATATTTGGGCCTGTATACAGTGGATGAGAAACAGTATATTCCAATGCATCCCTGCTGATCCAGAATGTCACCCAGAAGGACACAGGATCCTACACCATAAAAATCACAAAGCGAGGTGATAGGACTGAAGGAGTAACTGGATATTTGACCTTATGCC

>Pne_PSGP4N (Pygathrix nemaeus; Red shanked douc langur) WGS PVHW010054090.1 no ORF

CATCACTTTCAATCTTCTGGAACCTGCCCACCACTGCCCAAGTCACGATTGAAGCTCAGCCAACCAAAGTTTCTGAAGGGAAGGATATTCTTCTACTTGTCCACAATTTGCCCCAGAATCCTGCTGCCTAGTTCTGGTACAAAGGGCAAATAATGGACCTCCACCATTACATTCAGCATATACAATAGACACTGAAAGAGTTATATTCGGGCCTGCATACAGTACACGAGAAACTGTATATTCCAATGCTTTCCTGCTGATCCAGAGTGTCACCTAGAAGGACGCAGGATCCTACACCATAGAAATCATAAAGCAAGGTGATAGGACTGAAGGAGTAACTGGACATTTCACCTTATACC

>Pne_PSGP5N (Pygathrix nemaeus; Red shanked douc langur) WGS PVHW010053896.1 no ORF

CATCACTTTCAATCTTCTGGAACCCGCCCACCACTGCCCAAGTCACGATTGAAGCTCAGCCAACCGAAGTTTCTGAAGGGAAGGATAGTCTTCTATTTGTCCACAATTTGCCCCAGAATCCTGCTGCCTACTTCTGGTACAAAGGGCAAATAATGGACCTCCACCATTACATTCAGCATATACAATAGACACTGAAAGAGTTATATTCGGGCCTGCATACAGTGCAGGAGAAACAGTATATTCCAATGCTTTCCTGCTGATCCAGAGTGTCACCTAGAAGGACGCAGGATCCTACACCGTAGAAATCATAAAGCAAGGTGATAGGACTGAAGGAGTAACTGGACATTTCACCTTATACC

>Ppa_PSG1N (Pan paniscus; bonobo) XM_008955506.1

CATCACTTTTAAACTTCTGGAACCCACCCACCACAGCCCAAGTCACGATTGAAGCTCAGCCACCCAAAGTTTCCAAGGGGAAGGATGTTCTTCTACTTGTCCACAATTTGCCCCAGAATCTTACCGGCTACATCTGGTACAAAGGGCAAATGAGGGACCTCTACCATTACATTACATCATATGTAGTAGATGGTCAAAGAATTATATATGGGCCTGCATATAGTGGACGAGAAACAGTATATTCCAATGCATCCCTGCTGATCCAGAATGTCACCTGGGAGGACGCAGGATCCTACACCTTACACATCATAAAGGGAGGTGATGAGACTAGAGGAGTAACTGGACGTTTCACCTTCACCTTATACC

>Ppa_PSG2N (Pan paniscus; bonobo) WGS AJFE02044804

CATCACTTTTAAACTTCTGGAACTCGCCCACCACTGCCCAAGTTACGATTGAAGCCCGGCCACCAAAAGTTTCCGAGGGGAAGGATGTTCTTCTACTTGTCCACAATTTGCCCCAGAATCTTACTGGCTACATCTGGTACAAAGGGCAAATAAGGGACCTCTACCATTACATTACATCATATGTAGTAGACGGTCAAATAATTATATATGGGCCTGCATATAGTGGACGAGAAACAGTATATTCCAATGCATCCCTGCTGATCCAGAATGTCACCCGGGAGGACGCAGGATCCTACACCTTACACATCATAAAGCGAGGTGATGGGACTAGAGGAATAACTGGAAATTTCACCTTCACCTTATACC

>Ppa_PSG3N (Pan paniscus; bonobo) XM_008964142.2

CATCACTTTTAAACTTCTGGAACCCGCCTACCACTGCCCAAGTCACGATTGAAGCCCAGCCAACCAAAGTTTCCAAGGGGAAGGACGTTATTCTACTTGTCCACAATTTGCCCCAGAATCTTGCTGGCTACATCTGGTACAAAGGGCAAATGACGGACCTCTACCATTACATTACATCATACGTAGTAGATGGTCAAATAATTATATATGGGCCTGCATACAGTGGACGAGAAACAGTATATTCCAATGCATCCCTGCTGATCCAGAATGTCACCCGGGAGGACGCAGGATCCTACACCTTACACATCGTAAAGCGAGGTGATGGGACTAGAGGAATAACTGGACATTTCACCTTCACCTTATACC

>Ppa_PSG4N (Pan paniscus; bonobo) XM_008966588.1

CATCACTTTTAAACTTCTGGAACCTGCCCACAACTGCCCAAGTCACGATTGAAGCCCTGCCACCCAAAGTTTCTGAGGGGAAGGATGTTCTTCTACTTGTCCACAATTTGCCCCAGAATCTTGCTGGCTACATTTGGTACAAAGGGCAAATGACATACCTCTACCATTACATTACATCATATGTAGTAGACAGTCAAAGAATTATATATGGGCCTGCATACAGTGGAAGAGAAACAGTATATTCCAACGCATCCCTGCTGATCCAGAATGTCACCTGGGAGGACGCAGGATCCTACACCTTACACATCATAAAACGAGGTGATGGGACTGGAGGAGTAACTGGACATTTCACCTTCACCTTATACC

>Ppa_PSG5N (Pan paniscus; bonobo) WGS SSBP01000261.1

CATCACTTTTAAACTTCTGGAACCTGCCTACCACTGCCCAAGTCACGATTGAAGCCCTGCCACCCAAAGTGTCTGAGGGGAAGGATGTTCTTCTACTTGTCCACAATTTGCCCCAGAATCTAGCTGGCTACATCTGGTACAAAGGACAACTGATGGACCTCTACCATTACATTACATCATATGTAGTAGACGGTCAAATAAATGTATATGGGCCTGCATACACTGGACGAGAAACAGTATATTCCAATGCATCCCTGCTGATCCAGAATGTCACCCGGGAGGATGCAGGATCCTACACCTTACACATCATAAAGCGAGGTGATAGGACTAGAGGAGTAACTGGATATTTCACCTTCAACTTATACC

>Ppa_PSG6N (Pan paniscus; bonobo) XM_008955507.1;

CATCACTTTTAAACTTCTGGAACCTGCCCACCACTGCCCAAGTAATAATTGAAGCCCAGCCACCCAAAGTTTCCGAGGGGAAGGATGTTCTTCTACTTGTCCACAATTTGCCCCAGAATCTTACTGGCTACATCTGGTACAAAGGGCAAATGAGGGACCTCTACCATTACATTACATCATATGTAGTAGACGGTCAANATATTATATATGGGCCTGCATACAGTGGACGAGAAACAGTATATTCCAATGCATCCCTGCTGATCCAGAATGTCACCCAGGAGGATGCAGGATCCTACACCTTACACATCATAAAGCGAGGCGATGGGACTGGAGGAGTAACTGGACATTTCACCTTCACCTTATACC

>Ppa_PSG7N (Pan paniscus; bonobo) WGS SSBP01000261.1

CATCACTTTTAAACTTCTGGAACCCGCCCACCACAGCCCAAGTCACGATTGAAGCCCAGCCACCAAAAGTTTCCGAGGGGAAGGATGTTCTTCTACTTGTCCACAATTTGCCCCAGAATCTTACCGGCTACATCTGGTACAAAGGGCAAATCAGGGACCTCTACCATTACGTTACATCATATGTAGTAGACGGTCAAATAATTATATATGGGCCCGCATATAGTGGACGAGAAACAGTATATTCCAATGCATCCCTGCTGATCCAGAATGTCACCCGGGAAGATGCAGGATCCTACACCTTACACATCATAAAGCGTGGTGATGGGACTGGAGGAGTAACTGGACATTTCACCTTCACCTTATACC

>Ppa_PSG8N (Pan paniscus; bonobo) XM_008964139.1

CATCACTTTTAAACTTCTGGAACCCACCCACGACTGCCCAAGTCACGATTGAAGCCCAGCCAACCAAAGTTTCCGAGGGGAAGGATGTTCTTCTACTTATCCACAATTTGCCCCAGAATCTTACCGGCTACATCTGGTACAAAGGGCAAATGAGGGACCTCTACCATTACATTACATCATATGTAGTAGATGGTCAAAGAATTATATATGGGCCTGCATACAGTGGACGAGAAACAATATATTCCAATGCATCCCTGCTGATCCAGAATGTCACCCGGGAAGACGCAGGATCCTACACCTTACACATCATAAAGGGAGGTGATGAGACTAGAGGAGTAACTGGACATTTCACCTTCACCTTATACC

>Ppa_PSG9N (Pan paniscus; bonobo) XM_008966587.1

CATCACTTTTAAACTTCTGGAACCTGCCTACCACTGCCGAAGTCACGATTGAAGCCCAGCCACCCAAAGTGTCTGAGGGGAAGGATGTTCTTCTACTTGTCCACAATTTGCCCCAGAATCTTCCTGGCTACTTCTGGTACAAAGGGCAACTTACGGACCTCTACCATTACATTATATCATATATAGTAGATGGTAAAATAATTATATATGGGCCTGCATACAGTGGAAGAGAAACAGTATATTCCAACGCATCCCTGCTGATCCAGAATGTCACCCGGGAGGATGCAGGAACCTACACCTTACACATCATAAAGCGAAGTGATGAGACTAGAGAAGAAATTCGATATTTCACCGTCACCTTATACT

>Ppa_PSG10N (Pan paniscus; bonobo) XM_014342697.1

CATCACTTTTAAACTTCTGGAACCCACCTACCATTGCCCAAGTCACGATTGAAGCCCAGCCACCCAAAGTTTCCGAGGGGAAGGATGTTCTTCTGCTTGTCCACAATTTGCCCCAGAATCTTACTGGTTACATGTGGTACAAAGGGCAAATAAGGGACCTCTACCATTACATTACATCATATGTAGTAGACGGTCAAAGAATTACATATGGGCCTACATACAGTGGACGAGAAACAGTATATTCCAATGCATCCCTGCTGATCCAGAATGTCACCTGGGAGGACGCAGGATCCTACACATTACACATCATACAGCGAGGTGATGGGACTAGAGGAGTAACTGGAAATTTCACCTTCACCTTATACC

>Ppa_PSG11N (Pan paniscus; bonobo) XM_008955500.1

CATTACTTTTAAACTTCTGGAACCTGCCTACCACTGCCCAAGTCATGATTGAAGCCCAGCCACCCAAAGTGTCTGAGGGGAAGGATGTTCTTCTACTTGTCCACAATTTGCCCCAGAATCTTACTGGCTACATCTGGTACAAAGGGCAAATAAGGGACCTCTACCATTACATTACATCATATGTAGTAGACGGTCAAATAATTATATATGGACCGGCATACAGTGGACGAGAAACAGTATATTCCAATGCATCCCTGCGGATCCAGAATGTCACACGGGAGGACGCAGGATCCTACACCTTACACATCATAAAGCGAGGTGATGGGACTAGAGGAATAACTGGAAATTTCACCTTCACCTTATACC

>Ppi_PSG1N (Pithecia pithecia; white-faced saki) WGS PVIP01058652.1

CATCACTTTTAAACTTCTGGAACCCACCCACCACTGCCCAAGTCGTGATTGAAGCCCAGCCACATGTTGTTTCAGAGGGAAAGGATGTTCTTCTACTTGTCCGCAATTTGCCCCAGAATCTTACTGGCTACAGCTGGTACAGAGGGCCAATTATGGACATCCACCATTACGTTACAGCATATTTAATAGAAAAGGAAATAATTATACAAGGGCCTGCATACAGTGGACGAGAAACAATATATTCCAATGCATCTCTGCTGATCCAGAACCTCACCCAGAATGACGCAGGAATCTACACCCTACAAGTCACCAAGCAAGGTGAAAGGAATAAAGGAGTAACTGGACATTTCATCTTACACC

>Ppi_PSG2N (Pithecia pithecia; white-faced saki) WGS PVIP01052437.1

CATCACTTTTAAACTTCTGGAACCCACCCACCACTGCCCAAGTCGTGATTGAAGCCCAGCCACACGTTGTTTCAGAGGGAAAGGATGTTCTTCTACTTGTCCACAATTTGCCCCAGAATCTTACTGGCTACAGCTGGTACAGAGGGCCAATTATGGACGTCCACCATTACGTTACAGCATATTTAATAGAAAAGGAAGTAATTATACAAGGGCCTGCATACAGTGGACGAGAAACAATATATTCCAATGCATCCCTGCTGATCCAGAACGTCACCCAGAATGACGCAGGAATCTACACCCTACATATCACCAAGCAAGGTGAAAAGAATAAAGGAGTAACTGGACATTTCATCTTACACC

>Ppi_PSG3N (Pithecia pithecia; white-faced saki) WGS PVIP01043088.1

CATCACTTTTAAACTTCTGGAACCCACCCACCACTGCCCAAGTCGTGATTGAAGCCCAGCCACACGTTGTTTCAGAGGGGAAGGATGTTATTCTACTTGCCCGCAATTTGCCCCAGAATGTTTTTCACTACAGCTGGTACAGAGGGACAGTTATGGACATCAACCATTACATTACAGCATATTTAATAGGCACTGAAGTAATTATATATGGGCCTGCATACAGTGGACGAGAAACAATATATTCCAATGCATCCCTGCTGATCCAGAACGTCACCCAGAATGACGCAGGAACCTACATCCTACAAGTCACCAAGCAAGGTGAAAGGAGTAAGGGAGTAACTGGACATTTCATCTTACACC

>Ppi_PSG4N (Pithecia pithecia; white-faced saki) WGS PVIP01059527.1

CATCACTTTTAAACTTCTGGAACCCACCCACCACTGCCCAAGTCGTGATTGAAGCCCAGCCACATGTTGTTTCAGAGGGAAAGGATGTTCTTCTACTTGTCCACAATTTGCCCCAGAATCTTACTGGCTACAGCTGGTACAGAGGGCCAATTATGGACGTCCACCATTACGTTACAGCATATTTAATAGAAAAGGAAGTAATTATACAAGGGCCTGCATACAGTGGACGAGAAACAATATATTCCAATGCATCCCTGCTGATCCAGAACGTCACCCAGAATGACGCAGGAATCTACACCCTACATGTCACCAAGCAAGGTGAAAAGAATAAAGGAGTAACTGGATATTTCATCTTACACC

>Pte_PSG1N (Piliocolobus tephrosceles; Ugandan red colobus) WGS PDMG03014413.1

CATCACTTTTAATCTTCTGGAACCCGCCCACCACTGCCCAAGTCACAATTGAAGCCCAGCCAACCACAGTTTCCGAGGGGAAGGATGTTCTGCTACTTGTCCACAATTTGCCCCAGAATGTTACTGGCTACATCTGGTACAAAGGGCAAATAATGGACCTCCACCATTACATTACAGCATATACAATAGACACTGAAACGATTATACTTGGGCCTGCATACAGTGGACGAGAAGCAATATATTCCAATGCATCCCTGCTGATCCAGAATGTCACCCAGAATGACACAGGATCCTACACCATTCAAATCACACAGCGAGGTGATGGGACTAAAGGAGTAACTGGACATTTCACCTTATACC

>Pte_PSG2N (Piliocolobus tephrosceles; Ugandan red colobus) WGS PDMG03018444.1

CATCACTTTTAATCTTCTGGAACCCGCCCATCGCTGCCCAAGTCATGATTGAAGCCCAGCCAACCAAAGTTTCTGAGGGGAAGGATGTTCTGCTACTTGTCCACAATTTGCCCCAGAATGTTACTGGCTACATCTGGTACAAAGGGCAAATAATGGACCTCCACCATTACATTACATCATATGTAATAGACACTGAAATGATTATACTTGGGCCTGCATACAGTGGACGAGAAACTGTATATTCCAATGCATCCCTGCTGATCCAGAATGTCACCCGGAACGACACAGGATCCTACACCATTCAAATGACACAGCGAGGTGATGGGACTAAAGGAGTAACTGGACATTTCACCTTATACC

>Pte_PSG3N (Piliocolobus tephrosceles; Ugandan red colobus) WGS PDMG03000021.1

CATCACTTTTAATCTTCTGGAACCCACACGCCACTGCCCAAGTCACGATTGAAGCCCAGCCAACAAAAGTTTCTGAGGGGAAGGATGTTCTGCTACTTGTCCACAATTTGCCCCAGAATCTTGCTGGATACATCTGGTACAAAGGGCAAATAATGGACCTCCACCATTACCTTACAGCATATGTAATAGACACTGAAATGATTATACTTGGGCCTGCATACAGTGGACGAGAAACAATATATTCCAATGCATCCCTGCTGATCCAGAATGTCACCCAAAATGACACAGGATCCTACACCATACAATTCATAAAGCGAGGTGATAAGACTAAAGGAGTAACTGGACATTTCACCTTATACC

>Pte_PSG4N (Piliocolobus tephrosceles; Ugandan red colobus) WGS PDMG03000021.1

CATCACTTTTAATCTTCTGGAACCCGCCCATTGCTGCCCAAGTCTTGATTGAAGCACAGCCAAACAAAGTTTCTGAGGGGAAGGATGTTCTTCTACTTGTCCACAATTTGCCCCAGAATCTTGCTGCCTACATCTGGTACAAAGGGCAAATAATGGACCTCCACCATTACATTACAGCATATGTAATAGACACTGAAATAATTATATATGGGCCTGCATACAGTGGACGAGAAACAATATATTCCAATGCATCCCTGCTGATCCAGAATGTCACCCGGAACGACACAGGATCCTACACCATACAAACCATAAAGCGAGGTGATAGCACTAAAGGAGTAACTGGACATTTCACCTTATACC

>Pte_PSG5N (Piliocolobus tephrosceles; Ugandan red colobus) WGS PDMG03000021.1

CATCACTTTTAATCTTCTGGAACATGCCCACCACTGCCCAAGTCACGATTGAAGCCCAGCCAACCAAAGTTTCTGAGGGGAAGGATGTTCTGCTACTTGTCCACAACTTGCCCCAGAATCCTATTGGCTACATCTGGTACAAAGGGCAAATAATGGACATCCACCATTACATTACATCATATGTAATAGACGCTGAAACAATTATATCTGGGCCTGCATACAGTGGACGAGAAACAGTATATTCCAATGCATCCCTGCTGATCCAGAATGTCACCCAGAACGACACAGGATCCTACACCATACAAATCATGAAGCTAGGTGATAAGACTGAAGGACTAACTGGACATTTCACCTTATACC

>Pte_PSG6N (Piliocolobus tephrosceles; Ugandan red colobus) WGS PDMG03000021.1

CATCACTTTTACTCTTCTGGAACCCGCCCACTACTGCTCAAGTCACGATTGAAGCCCAGCCAACCAAAGTTTCTGAGGGGAAGGATGTTCTTCTACTTGTCCATAATTTGCCCCAGAATCTTACTGGCTACATCTGGTACAAAGGGCAAATAATGGACTACCACCATTACATTACATCATATGTAATAGACCCTGAAACAATTATATTTGGGCCTGCATACAGTGGACGAGAAACAGTATATTCCAATGCATCCCTGCTGATCGAGAATGTCACCCGGAATGACACAGGATCCTACACCATAAAAATCATAAAGCGAGGTGATAGGACTGAAGGAATAACTGGACATTTCACCTTATACC

>Pte_PSG7N (Piliocolobus tephrosceles; Ugandan red colobus) WGS PDMG03000021.1

CATCACTTTTAATCTTCTGGAACCCGCCCACCACTGCCCAAGTCACGATTGAAGCACAGCCAACCAATGTTTCTGAGGGGAACAATGTTCTTCTACTTGTACACAATTTGCCCCAGAATCCTGCTGCCTACATCTGGTACAAAGGGCAAATAATGGACCTCCACCATTACATTACAGCATATGTAATAGAAACTGAAAGAATTGTATTTGGGCCTGCATATAGTGGACGAGAAACAGTATATTCCAATGCATCCCTGCTGATCCAGAGTCTCAACCAGAAGGACGCAGGATCCTACACCGTAGAAATCATAAAGCGAGGTGATGGGAATGAAGGAGTAACTGGAAATTTCACCTTATACC

>Pte_PSG8N (Piliocolobus tephrosceles; Ugandan red colobus) WGS PDMG03000021.1

CATCACTTTTAATCTTCTGGAACCCGCCCACCACTGCCCAAGTCATGATTGAAACTCAACCAACCAAAGTTTCTGAGGGGAAGGATGTTCTTCTACTTGTCCACAATTTGCCCCCAAATCCTGCTGCCTACGTCTGGTACAAAGGGCAAATAATGGACTTCAACCAATTCATTATAGCATATACAACATACCCTGATAGAATTCTATTTGGGCCTGCAAGCAGTGGACGAGAAACACTATATTCCAATGGATCCCTGGGGATCCAGAATGTCACCAAGCAGGACACAGGATCCTACACCATAAGAGTCATGAAGCGAATGGATGGTACTAAAGGAGTAACTGGACATTTCACCTTATACC

>Pte_PSG9N (Piliocolobus tephrosceles; Ugandan red colobus) WGS PDMG03009696.1

CATCACTTTTAATCTTCTGGAACGGGCCCACCACTGCTCAAGTCACGATTGAAGCTCAGCCAACCAAAGTCTCTGAGGGGAAGGATGTTCTTATACTTGTCCACAATTTGCCCCAGAATGTTGTTGGCTACATCTGGTACAAAGGGCAAATAATGGACCTCCACCATTACATTACAGCATATACAATAGACACTGAAACAATTATACTTGGGTCTGCATACAGTGGACGAGAAACAGTATATTCCAATGCATCCCTGCTGATCCAGAGTGTCACCAAGCAGGACACAGGATCCTACACCATAAAAATCATAAAGCGAGGTGATGGGAATGAAGGAGTAACTGGACATTTCACCTTATACT

>Pte_PSG10N (Piliocolobus tephrosceles; Ugandan red colobus) WGS PDMG03042439.1

CATCACTTTTCATCTTCTGGAACCTGCCTACCATGGCCCAAGTCATGATTGAAGATCAGCCAACCGAAGTTTCTGAGGGGAAGGATGTTCTTCTACTTGTCCACAATTTGCCCCAGAATCCTACTGGCTACATCTGGTACAAAGGGCAAATAACGGATATCCACAATTACATTACATCATATGTAATAGACGCTGAAATGATTATATTTGGGCCTGCATACAGTGGACGAGAAACAGTATATTCCAATGCATCCCTGCTGATCCAGAATGTCACCCAGAAGGACACAGGATCCTACAACATACAAATCACAAAGCGAGGTGATAGCACTAAAGGAGTAACTGAATGTTTCACCTTATACC

>Pte_PSG11N (Piliocolobus tephrosceles; Ugandan red colobus) WGS PDMG03028859.1

CATCACTTTTAATCTTCTGGAACCCACCCACCGCTGCCGAAGTCATGATTGAAGCACAGCCAACCAAAGTTTCCGAGGGGAAGGATGTTCTTCTACTTGTTCATAATTTGCCCCAGAATGTTGCTGCCTACATCTGGTACAAAGGGCAAATAACGGACGTCCACCATTACATTACGGGATATGTAATAGACCCTGAAACAATTATATTTGGGCCTGCATACACTGGACGAGAAAGAGTATATTCCAACGCATCCCTGCTGATTCAGAAAGTCACCCAGAAGGACGCAGGATCCTACACCATAAAAATCACAAAGCGAGGTGATAAGACTAAAGGAGTAACTGGACATTTCACCTTATACT

>Pte_PSGP1N (Piliocolobus tephrosceles; Ugandan red colobus) WGS PDMG03000021.1 no ORF

CATCACTTTTAATCTTCTGGAACCCACCCATCCCTGCGCAAGTCACGATTGAAGCCCAGCCAACAAAAGTTTCCGAGGGGAAGGATGTTCTGCTACTTGTCCACAATTTGCCCCAGAATCTTATTGGATACATCTGGTACAAAGGGCAAATAATGGACCTCCACCATTACATTATGGGATATGTAATAGAGGCTGAAGCAATTATATTTGGGCCTGCATACAGTGGATGAGAAATAGTATATTCCAATGCATCCCTGCTGATCCAGAATGTCACCCAGAATGACACAGGATCCTACACCATACAAATCATAAAGCGAGGTGATAAGACTAAAGGAGTAACTGGACATTTCACCTTATACC

>Pte_PSGP2N (Piliocolobus tephrosceles; Ugandan red colobus) WGS PDMG03000021.1 no ORF

CATCACTTTTAATCTTCAGGAACTCGCCCACCATGGCCCAAGTCACGATTGAAGCCCAGCCAACCAAAGTTTCTGAGGGGAAGGATGCTCTGTTACTTGTCCACAATTTGCCCCAGAATGTTGCTGCCTACATCTGGTACAAAGGGCAAATAATGGACCTCCACCATTACATTACAGCATATACAATAGACACTGAAATGATTATATTTGGGCCTACATACAGTGGATGAGAAACTATATATCCCAATGCATCCCTGTTGATCCAGAATGACACCCAGAATGACACAGGATCCTACACCATTCAAATCACATAGCGAGGTGATGGGACTAAATGAGTAACTGGACACTTCACCTTATACC

>Ptr_PSG1N (Pan troglodytes; chimpanzee) WGS NBAG03000326.1

CATCACTTTTAAACTTCTGGAACCCGCCCACCACAGCCCAAGTCACGATTGAAGCCCAGCCACCCAAAGTTTCCAAGGGGAAGGATGTTCTTCTACTTGTCCACAATTTGCCCCAGAATCTTACTGGCTACATCTGGTACAAAGGGCAAATGAGGGACCTCTACCATTACATTACATCATATGTAGTAGACGGTCAAATAATTATATATGGGCCTGCATATAGTGGACGAGAAACAGTATATTCCAATGCATCCCTGCTGATCCAGAATGTCACCTGGGAGGACGCAGGATCCTACACCTTACACATCATAAAGGGAGGTGATGAGACTAGAGGAGTAACTGGACGTTTCACCTTCACCTTATACC

>Ptr_PSG2N (Pan troglodytes; chimpanzee) WGS NBAG03000560.1

CATCACTTTTAAACTTCTGGAACTCGCCCACCACTGCCCAAGTTACGATTGAAGCCCAGCCACCAAAAGTTTCCGAGGGGAAGGATGTTCTTCTACTTGTCCACAATTTGCCCCAGAATCTTACTGGCTACATCTGGTACAAAGGGCAAATAAGGGACCTCTACCATTACATTACATCATATGTAGTAGACGGTCAAATAATTATATATGGGCCTGCATATAGTGGACGAGAAACAGTATATTCCAATGCATCCCTGCTGATCCAGAATGTCACCCGGGAGGACGCAGGATCCTACACCTTACACATCACAAAGCGAGGTGATGGGACTAGAGGAATAACTGGAAATTTCACCTTCACCTTATACC

>Ptr_PSG3N (Pan troglodytes; chimpanzee) WGS AACZ04019656.1

CATCACTTTTAAACTTCTGGAACCCGCCTACCACTGCCCAAGTCACGATTGAAGCCCAGCCAACCAAAGTTTCCAAGGGGAAGGACGTTCTTCTACTTGTCCACAATTTGCCCCAGAATCTTGCTGGCTACATCTGGTACAAAGGGCAAATGACGGACCTCTACCATTACATTACATCATACGTAGTAGATGGTCAAATAATTATATATGGGCCTGCATACAGTGGACGAGAAACAGTATATTCCAATGCATCCCTGCTGATCCAGAATGTCACCCGGGAGGACGCAGGATCCTACACCTTACACATCGTAAAGCGAGGTGATGGGACTAGAGGAATAACTGGACATTTCACCTTCACCTTATACC

>Ptr_PSG4N (Pan troglodytes; chimpanzee) WGS NBAG03000560.1

CATCACTTTTAAACTTCTGGAACCTGCCCACAACTGCCCAAGTCACGATTGAAGCCCTGCCACCCAAAGTTTCTGAGGGGAAGGATGTTCTTCTACTTGTCCACAATTTGCCCCAGAATCTTGCTGGCTACATTTGGTACAAAGGGCAAATGACATACCTCTACCATTACATTACATCATATGTAGTAGACAGTCAAAGAATTATATATGGGCCTGCATACAGTGGAAGAGAAACAGTATATTCCAACGCATCCCTGCTGATCCAGAATGTCACCTGGGAGGACGCAGGATCCTACACCTTACACATCATAAAACGAGGTGATGGGACTGGAGGAGTAACTGGACATTTCACCTTCACCTTATACC

>Ptr_PSG5N (Pan troglodytes; chimpanzee) WGS AACZ04019636.1

CATCACTTTTAAACTTCTGGAACCTGCCTACCACTGCCCAAGTCACGATTGAAGCCCTGCCACCCAAAGTGTCTGAGGGGAAGGATGTTCTTCTACTTGTCCACAATTTGCCCCAGAATCTAGCTGGCTACATCTGGTACAAAGGACAACTGATGGACCTCTACCATTACATTACATCATATGTAGTAGACGGTCAAATAAATATATATGGGCCTGCATACACTGGACGAGAAACAGTATATTCCAATGCATCCCTGCTGATCCAGAATGTCACCCGGGAGGATGCAGGATCCTACACCTTACACATCATAAAGCGAGGTGATAGGACTAGAGGAGTAACTGGATATTTCACCTTCAACTTATACC

>Ptr_PSG8N (Pan troglodytes; chimpanzee) WGS AACZ04019656.1

CATCACTTTTAAACTTCTGGAACCTGCCCACCACTGCCCAAGTCACGATTGAAGCCCAGCCAACCAAAGTTTCCGAGGGGAAGGATGTTCTTCTACTTATCCACAATTTGCCCCAGAATCTTACCGGCTACATCTGGTACAAAGGGCAAATGAGGGACCTCTACCATTACATTACATCATATGTAGTAGACAGTCAAAGAATTATATATGGGCCTGCATACAGTGGACGAGAAACAATATATTCCAATGCATCCCTGCTGATCCAGAATGTCACCCGGGAAGACGCAGGATCCTACACCTTACACATCATAAAGGGAGGTGATGAGACTAGAGGAGTAACTGGACATTTCACCTTCACCTTATACC

>Ptr_PSG9N (Pan troglodytes; chimpanzee) WGS AACZ04019635.1

CATCACTTTTAAACTTCTGGAACCCGCCCACCACTGCCGAAGTCACGATTGAAGCCCAGCCACCCAAAGTTTCTGAGGGGAAGGATGTTCTTCTACTTGTCCACAATTTGCCCCAGAATCTTCCTGGCTACTTCTGGTACAAAGGGGAAATTACGGACCTCTACCATTACATTATATCATATATAGTAGATGGTAAAATAATTATATATGGGCCTGCATACAGTGGAAGAGAAACAGTATATTCCAACGCATCCCTGCTGATCCAGAATGTCACCCGGGAGGATGCAGGAACCTACACCTTACACATCATAAAGCGAAGTGATGAGACTAGAGAAGAAATTCGATATTTCTCCTTCACCTTATACT

>Ptr_PSG10N (Pan troglodytes; chimpanzee) WGS AADA01200754.1

CATCACTTTTAAACTTCTGGAACCCACCTACCATTGCCCAAGTCACGATTGAAGCCCAGCCACCCAAAGTTTCCGAGGGGAAGGATGTTCTTCTACTTGTCCACAATTTGCCCCAGAATCTTACTGGTTACATGTGGTACAAAGGGCAAATAAGGGACCTCTACCATTACATTACATCATATGTAGTAGACGGTCAAAGAATTACATATGGGCCTACATACAGTGGACGAGAAACAGTATATTCCAATGCATCCCTGCTGATCCAGAATGTCACCCGGGAGGACGCAGGATCCTACACCTTACACATCATACAGCGAGGTGATGGGACTAGAGGAGTAACTGGAAATTTCACCTTCACCTTATACC

>Ptr_PSG11N (Pan troglodytes; chimpanzee) WGS AACZ04019644.1

CATTACTTTTAAACTTCTGGAACCTGCCTACCACTGCCCAAGTCATGATTGAAGCCCAGCCACCCAAAGTGTCTGAGGGGAAGGATGTTCTTCTACTTGTCCACAATTTGCCCCAGAATCTTACTGGCTACATCTGGTACAAAGGGCAAATCAGGGACCTCTACCATTACATTACATCATATGTAGTAGACGGTCAAATAATTATATATGGACCGGCATACAGTGGACGAGAAACAGTATATTCCAATGCATCCCTGCGGATCCAGAATGTCACCCGGGAGGACGCAGGATCCTACACCTTACACATCATAAAGCGAGGTGATGGGACTAGAGGAATAACTGGAAATTTCACCTTCACCTTATACC

>Rbi_PSG1N (Rhinopithecus bieti; black snub-nosed monkey) WGS MCGX01021099.1; XM_017861979.1

CATCACTTTTAATCTTCTGGAACCCGCCCACCACTGCCCAAGTCATGATTGAAGCACAGCCAAACAAAGTTTCTGAGGGGAAGGATGTTCTTCTACTTGTCCACAATTTGCCCCAGAATCTTGCTGCCTACATCTGGTACAAAGGGCAAATAATGGACCTCCACCATTACATTACAGCATATGTAATAGACACTGATACAATTATATTTGGGCCTGCATACAGTGGACGAGAAACAGTATATTCCAATGCATCCCTGCTGATCCAGAATGTCACCCAGAAGGACACAGGATCCTACACCATACAAATCATAAAGCGAGGTGATAGGACTGAAGCAGTAACTGGACATTTCACCTTATACC

>Rbi_PSG2N (Rhinopithecus bieti; black snub-nosed monkey) WGS MCGX01051582.1; XM_017866302.1

CATCACTTTTAATCTTCTGGAACCCACCTACCACGGCCCACGTCATGATTGAAGCTCAGCCAACCGAAGTTTCTGAGGGGAAGGATGTTCTTCTACTTGTCCACAATTTGCCCCAGAATCCTACTGGCTACATCTGGTACAAAGGGCAAATAATGGATATCCACAATTACATTACATCATATGTAATAGACACTGATACAATTATATTTGGGCCTGCATACAGTGGGCGAGAAACAGTATATTCCAATGCATCCCTGCTGATCCAGAATGTCACCCAGAAAGACACAGGATCCTACACCATACAAATCATAAAGCGAGGTGATAGCACTAAAGGAGTAACTGGACATTTCACCTTATACC

>Rbi_PSG3N (Rhinopithecus bieti; black snub-nosed monkey) MCGX01011242.1; XM_017849584.1

CATCACTTTTAATCTTCTGGAACCTGCCCACCACTGCTCAAGTCACGATTGAAGCTCAGCCAACCAAAGTTTCTGAGGGGAAGGATGTTCTTCTACTTGTCCACAATTTGCCCCAGAATCTTACTGGCTACATCTGGTACAAAGGGCAAATAATGGACTACCACCATTACATTACAGCATATGTAATAGACCCTGAAACAATTATATTTGGGCCTGCATACAGTGGACGAGAAACAGTATATTCCAATGCATCCCTGCTGATCCAGAATGTCACCCGGAACGACACAGGATCCTACACCATAAAAATCATAAAGCGAGGTGATAGGACTGAAGGAGTAACTGGACATTTCACCTTATACC

>Rbi_PSG4N (Rhinopithecus bieti; black snub-nosed monkey) WGS MCGX01023171.1; XM_017863242.1

CATCACTTTTAATCTTCTGGAACCCGCCCACCACTGCCCAAGTCACAATTGAAGCTCAGCCAACCAATGTTTCTGAGGGGAACGATGTTCTTCTACTTGTTCACAATTTGCCCCAGAATCCTGCTGCCTACATCTGGTACAAAGGGCAAATAATGGATCTCCACCATTACATTACAGCATATGTAATAGAAACGGAAAGAATTGTATTTGGGCCTGCATACAGTGGACGAGAAACAGTATATTCCAATGCATCCCTGCTGATCCAGAGTGTCAACCAGAAGGACGCAGGATCCTACACTGTAGAAATCATAAAGCGAGGTGATGGGAATGAAGGAGTAACTGGAAATTTCACCTTATACC

>Rbi_PSG5N (Rhinopithecus bieti; black snub-nosed monkey) WGS MCGX01018074.1; XM_017860021.1

CATCACTTTTAATCTTCTGGAACCCACCCACCACTGCGCAAGTCATGATTGAAGCCCAGCCAACAAAAGTTTCTGAGGGGAAGGATGTTCTTCTACTTGTCCACAATTTGCCCCATAATCTTGCTGGATACATCTGGTACAAAGGGAAAATAATGGACCTCTACCATTACATTACAGCATATACAATAGACACTGAAATGATTATATTTGGGCCTGCATACAGTGGACGAGAAACAGTATATTCCAATGCATCCCTGCTGATCCAGAATGTGACCCAGAAGGACACAGGATCCTACACGATACAAATCATAAAGCGAGGTGATAAGACTAAAGGAGTAACTGGACATTTCACGTTATACC

>Rbi_PSG6N (Rhinopithecus bieti; black snub-nosed monkey) WGS MCGX01002644.1 CATCACTTTTAATCTTCTGGAACACGCCCACCACTGCCCAAGTCACGATTGAAGCACAGCCAACCAAAGTATCTGAGGGGAAGGATGTTCTGCTACTTGTTCACAATTTGCCCCAGAATATTGCTGCCTATATCTGGTACAAAGGGCAAATAATGGACCTACGCCATTACATTACAGCATATATAATAGACACTGAAATGATTATACTTGGGCCTGCATACAGTGGACGAGAAACTGTATATTCCAATGCATCCCTGATGATCCAGAATGTCACCCAGAATGACACAGGATCCTACACCATTCAAATCACACAGCGAGGTGATGGAGCTAAAGGAGTAACTGGACATTTCACCTTATACC

>Rbi_PSG7N (Rhinopithecus bieti; black snub-nosed monkey) XM_017849954.1

caTCACTTTTAATCTTCTGGAACGCGCCCACCACTGCTCAAGTCACGACTGAAGCTCAGCCAACCAAAGTCTCTGAGGGGAAGGATGTTCTTCTACTTGTCCACAATTTGCCCCAGAATGTTGTTGGCTACATCTGGTACAAAGGGCAAATAATGGACCTCCGCCATTACATTACAGCATATACAATCGACAATGAAATTATATTTGGGCCTGCATACAGTGGACGAGAAAAAATATATTCCAATGCATCCCTGCTGATCCCGAATGTCACCAAGCAGGACATAGGATCCTACACCATAAAAATCATAAAGCGAGGTGATGAGACTAAAGGAGTAACTGGACATTTCACCTTATACC

>Rbi_PSG8N (Rhinopithecus bieti; black snub-nosed monkey) ; XM_017885826.1; MCGX01005392.1

caTTACTTTTCATCTTCTGGAACCCACCCACCACTGCCCAAGTCACGATTGAAGCTCAGCCAACCAAAGTTTCCGAGGGGAAGGATGTTCTTCTACTTGTCCACAATTTGCCCCAGAATCTTACCGGCTACATCTGGTACAAAGGGCAAATAATGGACCTCCACCAATTCATTACAGCATATACAATAGACACTGAAACAATTATATCTGGGCCTGCATACAGTGGACGAGAAACAGTATATTCCAATGCATCCCTGCTGATCCAGAATGTCACCAAGAATGACACAGGATCCTACACGATACAAATCATAAAGCGAGGTGATAAGATTAAACGAGTAACTGGACATTTCACCTTATACC

>Rbi_PSG9N (Rhinopithecus bieti; black snub-nosed monkey) WGS MCGX01013176.1

CATCACTTTTAATCTTCTGGAACCCGCCCACCGCTGCCCAAGTCATGATTGAAGCACAGCCAAACAAAGTTTCTGAGGGGAAGGATGTTCTTCTACTTGTCCACAATTTGCCCCAGAATGTTGCTGCCTACATCTGGTACAAAGGGCAAATAATCGACTTCCACCATTACATTACGGGATATGTAATGGAGGCTGAAGCAAATATATTTGGGCCTGCACACAGTGGACGAGAAACAGTATATTCCAATGCATCCCTGCTGATTCAGAATGTCACCCAGAAGAACACAGGATCCTACACCATACAAATCATGAAGCTAGGTAATAGGACTGAAGGAGTAACTGGACATTTCACCTTATACC

>Rbi_PSG10N (Rhinopithecus bieti; black snub-nosed monkey) WGS MCGX01027142.1

caTCACTTTTAATCTTCTGGAACCCACCCACCACTGCCCAAGTCACGATTGAAGCCCGGCCAACCAAAGTTTCTGAGGGCAAGGATGTTCTGCTACTTGTCCACAATTTGCCCCAGAATGTTACTGGCTACGTCTGGTACAAAGGGCAAATAATGGACCTCTACCATTACATTACAGCATATATAATAGACACTGAAATGATTATATTTGGGCCTGCATACAGTGGACGAGAAACTGTATATTCCAATGCATCCCTGCTGATCCAGAATGTCACCCAGAATGACACAGGATCCTACACCATTCAAATCACACAGCGAGGTGGTGGGATTAAAGGAGTAACTGGACATTTCACCTTATACC

>Rbi_PSG11N (Rhinopithecus bieti; black snub-nosed monkey) WGS MCGX01028843.1

CATCACTTTTAATCTTCTGGAACCCGCCCACCACTGCCCAAGTCATGATTGAAGCTCAACCAACCAAAGTTTCTGAGGGGAAGGATGTTCTTCTACTTGTCCACAATTTGCCCCGGAATGTTGCTGCCTACGTGTGGTACAAAGGGCAAATAATGGACTTCTACCAATTTATTACAGCATATTCAAGAGACCCTGATAGAATTCTATTTGGGCCTGCATACAGTGGACGAGAAACACTATATTCCAATGGATCCCTGCGGATCCAGAATGTCACCAAGCAGGACACAGGATCCTACACCATAAAAGTCATGAAGCGAATTGATGAGACTAAAGGAGTAACTGGACATTTCACCTTATACC

>Rbi_PSG12N (Rhinopithecus bieti; black snub-nosed monkey) WGS MCGX01077751.1 CATCACTTTTAATCTTCTGGAACCCACCCACCACTGCCCAAGTCATGATTGAAGCTCAACCAACCAAAGTTTCTGAGGGCAAGGATGTTCTTCTACTTGTCCACAATTTGCCCCAGAATCCTGCTGCCTACGTCTGGTACAAAGGGCAAATAATGGACTTCTACCAATTCATTATAGAATATACAAGATACCCTGATAGAATTCTATTTGGGCCTGCATACAGTGGACGAGAAACACTATATTCCAATGGATCCCTGCTGATCCAGAATGTCACCAAGCAGGACACAGGATCCTACACCGTAAAAATCATGAAGCGAATTGATGAGACTAAAGGAGTAACTGGACATTTCACCTTATACC

>Rbi_PSGP1N (Rhinopithecus bieti; black snub-nosed monkey) WGS MCGX01010162; XM_017847386.1

CATTACTTTTAATCTTCTGAAACTGGCCCACCACTGCTCAAGTCACGATTGAAGCTCAGCCAACCAAAGTTTCTGAGGGGAAGGATGTTCTGCTACTTGTCCACAATTTGCCCCAGAATCTTACTGGCTACATCTGGTACAAAGGGCAAATGATGGACCTCCACCATTACATTACAGCATATACAATAGATACTGAAATGATTATACTTGGGCCTGCATACAGTGGACGAGAAACTGTATATTCCAATGCATCCCTGCTGATCCAGAATGTCACCCAGAAGGACACAGGATCCTACACCATAAAAATCACAAAGCGAGGTGATAGGATTGACGGAGTAACTGGACATTTCACCTTatacc

>Rbi_PSGP2N (Rhinopithecus bieti; black snub-nosed monkey) WGS MCGX01023806.1

caTCACTTTTAATCTTCTGGAACCCGCCCACCACTGCCCAAGTCATGATTGAAGCCCAGCCAAACAAAGTTTCTGAGTGGAAGGATACTCTTCTACTTGTCCAGAATTAGCCCCAGAATCTTACTGGCTACATCTGGTACAAAGGGCAAATAATGGACCTCCACCAATTCATTACAGCATATACAATAGACACTGAAACAATTATATTTGGGCCTGCATACAGTGGATGAGAAACACTATATTCCAATGCATCCCTGCTGATCCAGAATGTCACCCAAAATGACACAGGATCCTACACCATACAAATCATGAAGCGAGGTGATGAGATTAAAGGAGTAACTGGACATTTCACCTTACACC

>Rbi_PSGP3N (Rhinopithecus bieti; black snub-nosed monkey) WGS MCGX01078639.1

CATCACTTTGAATCTTCTGGAACCCACCCACCACTGCCCAAGTCACGATTGAAGCTCAGCCAACCAAAGTTTCTGAACGGAAGGATGTTCTTCTACTTATCCACAATTTGCCCCAGAATCCTGCTGCCTACTTCTGGTACAAAGGGCAAATAATGGACCTCCACCATTACATTCAGCATATACAATAGACACTGAAAGAGTTATATTTGGGCCTGCATACAGTGGACGAGAAACAGTATATTCCAACGCTTCCCTGCTGATCCAGAGTGTCACCTAGAAGGACGCAGGATCCTACACCGTAGAAATCATAAAGCGAGGTGATAGGACTGAAGGAGTAACTGGACATTTCACCTTATACC

>Rro_PSG1N (Rhinopithecus roxellana; golden snub-nosed monkey) XM_010353520.1

CATCACTTTTAATCTTCTGGAACCCGCCCACCACTGCCCAAGTCATGATTGAAGCACAGCCAAACAAAGTTTCTGAGGGGAAGGATGTTCTTCTACTTGTCCACAATTTGCCCCAGAATCTTGCTGCCTACATCTGGTACAAAGGGCAAATAATGGACCTCCACCATTACATTACAGCATATGTAATAGACACTGATACAATTATATTTGGGCCTGCATACAGTGGACGAGAAACAGTATATTCCAATGCATCCCTGCTGATCCAGAATGTCACCCAGAAGGACACAGGATCCTACACCATACAAATCATAAAGCGAGGTGATAGGACTGAAGCAGTAACTGGACGTTTCACCTTATACC

>Rro_PSG2N (Rhinopithecus roxellana; golden snub-nosed monkey) XR_749094.1

CATCACTTTTAATCTTCTGGAACCTGCCCACCACTGCTCAAGTCACGATTGAAGCTCAGCCAACCAAAGTTTCTGAGGGGAAGGATGTTCTTCTACTTGTCCACAATTTGCCCCAGAATCTTACTGGCTACATCTGGTACAAAGGGCAAATAATGGACTACCACCATTACATTACAGCATATGTAATAGACCCTGAAACAATTATATTTGGGCCTGCATACAGTGGACGAGAAACAGTATATTCCAATGCATCCCTGCTGATCCAGAATGTCACCCGGAACGACACAGGATCCTACACCATAAAAATCATAAAGCGAGGTGATAGGACTGAAGGAGTAACTGGACATTTCACCTTATACC

>Rro_PSG3N (Rhinopithecus roxellana; golden snub-nosed monkey) XR_748939.1

CATCACTTTTAATCTTCTGGAACCCACCCACCACTGCGCAAGTCATGATTGAAGCCCAGCCAACAAAAGTTTCTGAGGGGAAGGATGTTCTTCTACTTGTCCACAATTTGCCCCAGAATCTTGCTGGATACATCTGGTACAAAGGGAAAATAATGGACCTCTACCATTACATTACAGCATATACAATAGACACTGAAATGATTATATTTGGGCCTGCATACAGTGGACGAGAAACAGTATATTCCAATGCATCCCTGCTGATCCAGAATGTGACCCAAAATGACACAGGATCCTACACCATACAAATCATAAAGCGAGGTGATAAGACTAAAGGAGTAACTGGACATTTCACCTTATACC

>Rro_PSG4N (Rhinopithecus roxellana; golden snub-nosed monkey) XM_010372415.1

TATTACTTTTCATCTTCTGGAACCCACCCACCACTGCCCAAGTCACGATTGAAGCCCGGCCAACCAAAGTTTCCGAGGGGAAGGATGTTCTTCTACTTGTCCATAATTTGCCCCAGAATCTTACTGGCTACATCTGGTACAAAGGGCAAATAATGGACCTCCACCAATTCATTACAGCATATACAATAGACACTGAAACAATTATATCTGGGCCTGCATACAGTGGACGAGAAACAGTATATTCCAATGCATCCCTGCTGATCCAGAATGTCACCAAGAATGACACAGGATCCTACACGATACAAATCATAAAGCGAGGTGATAAGACTAAAGGAGTAACTGGACATTTCACCTTATACC

>Rro_PSG5N (Rhinopithecus roxellana; golden snub-nosed monkey) XR_747618.1

CATCACTTTTAATCTTCTGGAACCCACCCACCACTGCCCAAGTCACGATTGAAGCCCGGCCAACCAAAGTTTCTGAGGGCAAGGATGTTCTGCTACTTGTCCACAATTTGCCCCAGAATGTTACTGGCTACGTCTGGTACAAAGGGCAAATAATGGACCTCTACCATTACATTACAGCATATATAATAGACACTGAAATGATTATATTTGGGCCTGCATACAGTGGACGAGAAACTGTATATTCCAATGCATCCCTGCTGATCCAGAATGTCACCCAGAATGACACAGGATCCTACACCATTCAAATCACACAGCGAGGTGATGGGATTAAAGGAGTAACTGGACATTTCACCTTATACC

>Rro_PSG6N (Rhinopithecus roxellana; golden snub-nosed monkey) XM_010375567.1

CATCACTTTTAATCTTCTGGAACCCGCCCACCACTGCCCAAGTCATGATTGAAGCTCAACCAACCAAAGTTTCTGAGGGGAAGGATGTTCTTCTACTTGTCCACAATTTGCCCCGGAATGTTGCTGCCTACGTGTGGTACAAAGGGCAAATAATGGACTTCTACCAATTTATTACAGCATATTCAAGAGACCCTGATAGAATTCTATTTGGGCCTGCATACAGTGGACGAGAAACACTATATTCCAATGGATCCCTGCGGATCCAGAATGTCACCAAGCAGGACACAGGATCCTACACCATAAAAGTCATGAAGCGAATTGATGAGACTAAAGGAGTAACTGGACATTTCACCTTATACC

>Rro_PSG7N (Rhinopithecus roxellana; golden snub-nosed monkey) XM_010361982.1

CATCACTTTTAATCTTCTGGAACCCGCCCACCGCTGCCCAAGTCATGATTGAAGCACAGCCAAACAAAGTTTCTGAGGGGAAGGATGTTCTTCTACTTGTCCACAATTTGCCCCAGAATGTTGCTGCCTACATCTGGTACAAAGGGCAAATAATCGACTTCCACCATTACATTACGGGATATGTAATGGAGGCTGAAGCAAATATATTTGGGCCTGCACACAGTGGACGAGAAACAGTATATTCCAATGCATCCCTGCTGATTCAGAATGTCACCCAGAAGAACACAGGATCCTACACCATACAAATCATGAAGCTAGGTAATAGGACTGAAGGAGTAACTGGACATTTCACCTTATACC

>Rro_PSG8N (Rhinopithecus roxellana; golden snub-nosed monkey) XR_749338.1

CATCACTTTTAATCTTCTGGAATACGCCCACCACTGCCCAAGTCACGATTGAAGCACAGCCAACCAAAGTATCTGAGGGGAAGGATGTTCTGCTACTTGTCCACAACTTGCCCCAGAATCTTATTGGCTACATCTGGTACAAAGGCCAAGTAACGGACTTCCACCATTACATTACATCATATGTAATAGACGTTGAAACAATTATATTTGGGCCTGCATACAGTGGACGAGAAACAGTATATTCCAATGCATCCCTGCTGATCCAGAATGTCACCCGGAAGGACACAGGATCCTACACCATACAAATCATAAAGCTAGGTGATAGGACTAAAGGAGTAACTGGACATTTCACCTTATACC

>Rro_PSG9N (Rhinopithecus roxellana; golden snub-nosed monkey) XM_010385443.1

CATCACTTTTAATCTTCTGGAACACGCCCACCACTGCCCAAGTCACGATTGAAGCACAGCCAACCAAAGTATCTGAGGGGAAGGATGTTCTGCTACTTGTCCACAACTTGCCCCAGAATCTTATTGGCTACATCTGGTACAAAGGCCAAGTAATGGACTTCCACCATTACATTACATCATATGTAATAGACGCTGAAACAATTATAACTGGGCCTGCATACAGTGGACGAGAAACAGTATATTCCAATGCATCCCTGCTGATCCAAAATGTCACCCGGAAGGACACAGGATCCTACACCATACAAATCATAAAGCTAGGTGATAGGACTAAAGGAGCAACTGGACATTTCACCTTATACT

>Rro_PSG10N (Rhinopithecus roxellana; golden snub-nosed monkey) XM_010381087.1

CATCACTTTTAATCTTCTGGAACCCGCCCACCACTGTCCAAGTCACGATTGAAGCCCAGCCAACCAAAGTTTCTGAGGGGAAGGATGTTCTGCTACTTGTTCACAATTTGCCCCAGAATATTGCTGCCTATATCTGGTACAAAGGGCAAATAATGGACCTACGCCATTACATTACAGCATATATAATAGACACTGAAATGATTATACTTGGGCCTGCATACAGTGGACGAGAAACTGTATATTCCAATGCATCCCTGCTGATCCAGAATGTCACCCAGAATGACACAGGATCCTACACCATTCAAATCACACAGCGAGGTGATGGAGCTAAAGGAGTAACTGGACATTTCACCTTATACC

>Rro_PSG11N (Rhinopithecus roxellana; golden snub-nosed monkey) WGS ABR01013422.1; XM_010359802.1

CATCACTTTTAATCTTCTGGAACCCGCCCATCGCTGCCCAAGTCATGATTGAAGCCCAGCCAAGCAAAGTTTCCAACGGGAAGGATGTTCTTCTGCTTGTTCACAATTTGCCCCAGAATGTTGCTGCCTACATCTGGTACAAAGGGCAAATAAGGGACCTCCACCATTACATTATGGGATATGTAATAGAGGCTGAAGCAATTTTATCTGGGCCTGCATACAGTGGACGAGAAACAGTATATTCCAATGCATCCCTGCTGATTCAGAATGTCACCCAGAAGGACACAGGATCCTACACCGTACAAATCATAAAGCAAGGTGATAAGACTGAAGGAGTAACTGGACATTTCACGTTATACT

>Rro_PSG12N (Rhinopithecus roxellana; golden snub-nosed monkey) XM_010381088.1

CATCACTTTTAATCTTCTGGAACGCGCCCACCACTGCTCAAGTCACGACTGAAGCTCAGCCAACCAAAGTCTCTGAGGGGAAGGATGTTCTTCTACTTGTCCACAATTTGCCCCAGAATGTTGTTGGCTACATCTGGTACAAAGGGCAAATAATGGACCTCCGCCATTACATTACAGCATATACAATCGACAATGAAACAATTATATTTGGGCCTGCATACAGTGGACGAGAAAAAATATATTCCAATGCATCCCTGCTGATCCCAAATGTCACCAAGCAGGACATAGGATCCTACACCATAAAAATCATAAAGCGAGGTGATGAGACTAAAGGAGTAACTGGACATTTCACCTTATACC

>Rro_PSG13N (Rhinopithecus roxellana; golden snub-nosed monkey) XM_010372740.1

CATCACTTTTAATCTTCTGGAACCCACCCACCACTGCCCAAGTCATGATTGAAGCTCAACCAACCAAAGTTTCTGAGGGCAAGGATGTTCTTCTACTTGTCCACAATTTGCCCCAGAATCCTGCTGCCTACGTCTGGTACAAAGGGCAAATAATGGACTTCTACCAATTCATTATAGAATATACAAGATACCCTGATAGAATTCTATTTGGGCCTGCATACAGTGGACGAGAAACACTATATTCCAATGGATCCCTGCTGATCCAGAATGTCACCAAGCAGGACACAGGATCCTACACCGTAAAAATCATGAAGCGAATTGATGAGACTAAAGGAGTAACTGGACATTTCACCTTATACC

>Rro_PSGP1N (Rhinopithecus roxellana; golden snub-nosed monkey)

CATTACTTTTAATCTTCTGAAACCGGCCCACCACTGCTCAAGTCACGATTGAAGCTCAGCCAACCAAAGTTTCTGAGGGGAAGGATGTTCTGCTACTTGTCCACAATTTGCCCCAGAATCTTACTGGCTACATCTGGTACAAAGGGCAAATGATGGACCTCCACCATTACATTACAGCATATACAATTGACACTGAAATGATTATACTTGGGCCTGCATACAGTGGACGAGAAACTGTATATTCCAATGCATCCCTGCTGATCCAGAATGTCACCCAGAAGGACACAGGATCCTACACCATAAAAATCACAAAGCGAGGTGATAGGATTGAAGGAGTAACTGGACATTTCACCTTCTGCC

>Sap_PSG1N (Sapajus apella; tufted capuchin) WGS WRPQ01048603.1

CATCACTTTTAAACTTCTGGAACCCACCCACCACTGCCCAAGTCATGGCTGAAGCCCAGCTACGCATTGTTTCAGAGGGGAAGGATGTTCTTCTACTTGTCCACAATTTACCCCAGAATCTTACTGGCTACAGCTGGTACAGAGGGAAAGTGATGGACATCCACCATTACCTTACACCATATTTAATAGACACTCAAATAACTATAGTTGGGCATGCATACAGTGGACGAGAAACAATATTTTCCAATGCATCCCTGCTGATCGAGAACGTCACCCAGAATGAAGGAGGACCCTACATCCTACAAGTCACCAAGCAAGGTGCTAGGAATGAAGGAGAAACCGGACACTTCACCTTAAAAC

>Sen_PSG1N (Semnopithecus entellus; Hanuman langur) WGS PVII010102250.1

CATCACTTTTAATCTTCCGGAACCTGCCCACCACTGCTCAAGTCACGACTGAAGCTCAGCCAACCAAAGTCTCTGAGGGGAAGGATGTTCTTCTACTTGTCCACAATTTGCCCCAGAATGTTGTTGGCTACATCTGGTACAAAGGGCAAATAATGGACGTCCACCAATTCATTACAGCATATACAATAGACACTGAAACAATTATACTTGGGCCTGTATACAGTGGACGAGAAAAAATATATTCCAACGCATCCCTGCTGATCCAGAATGTCACCCAGAATGACACAGGATCCTACACCATAAAAATCATAAAGCGAGGTGATGAGACTAAAGGAGTAACTGGACATTTCACCTTATACC

>Sen_PSG2N (Semnopithecus entellus; Hanuman langur) WGS PVII010083473.1

CATCACTTTTAATCTTCTGGAACTCACCCACCACTGCCCAAGTCACGATTGAAGCCCAGCCAACCAAAGTTTCTGAGGGGAAGGATGTTCTGCTGCTTGTCCACAATTTGCCCCAGAATGTTATTGGCTACGTCTGGTACAAAGGGCAAATAATGGACCTCTACCATTACATTACAGCATATATAATAGACACTGAAATGATTATATTTGGGCCTGCATACAGTGGACGAGAAACTGTATATTCCAATGCATCCCTGCTGATCCAGAATGTCACCCAGAATGACACAGGATCCTACACCATTCAAATCACACAGCGAGGTGATGGGATTAAAGGAGTAACTGGACATTTCACCTTATACT

>Sen_PSG3N (Semnopithecus entellus; Hanuman langur) WGS PVII010041833.1

CATCACTTTTAATCTTCTGGAACCCACCCACCACTGCCCAAGTCACGATTGAAGCCCAGCCAACCAAAGTTTCTGAGGGGAAGGATGTTCTGCTACTTGTTCACAATTTGCCCCAGAATATTGCTGCCTATATCTGGTACAAAGGGCAAATAATGGACCTCCGCCATTACATTACAGCATATATAATAGACACTGAAATGATTATACTTGGGCCTGCATACAGTGGACGAGAAACTGTATATTCCAATGCATCCCTGCTGATCCAGAATGTCACCCAGAATGACACAGGATCCTACACCATTCAAATCACACAGCGAGGTGATGGGATTAAAGGAGTAACTGGACATTTCACCTTATACC

>Sen_PSG4N (Semnopithecus entellus; Hanuman langur) WGS PVII010027329.1

CATTACTTTTAATCTTCTGGAACCCGCCCACCACTGCTCAAGTCACGATTGAAGCCCAGCCAACCAAAGTTTCTGAGGGGAAGGATGTTCTTCTACTTGTCCACAATTTGCCCCAGAATCTTACTGGCTACATCTGGTACAAAGGGCAAATGATGGACCTCTACCATTACATTACAGCATATACAATAGACACTGAAATAACTATATTTGGGCCTGCATACAGTGGACGAGAAACAGTATATTCCAATGCATCCCTGCTGATCCAGAATGTCACCCAGAAGGACACAGGATCCTACACCATAAAAATCATAAAGCGAGGTGATAGGATTGAAGGAGTAACTGGACATTTCACCTTATACC

>Sen_PSG5N (Semnopithecus entellus; Hanuman langur) WGS PVII010015627.1

CATCACTTTTAATCTTCTGGAACCCACCCACCGCTGCGCAAGTCATGATTGAAGCCCAGCCAACAAAAGTTTCTGAGGGGAAGGATGTTCTGCTACTTGTCCACAATTTGCCCCAGAATCTTGCTGGATATATCTGGTACAAAGGGCAAATAATGGACCTCTACCATTACATTACAGCATATACAATAGACACTGAAATGATTATATTTGGGCCTGCATACAGTGGACGAGAAACAGTATATTCCAATGCATCCCTGCTGATCCAGAATGTCACCCAAAATGACACAGGATCCTACACCATACAAATCATAAAGCGAGGTGATAAGACTAAAGGAGTAACTGGACATTTCACCTTATACC

>Sen_PSG6N (Semnopithecus entellus; Hanuman langur) WGS PVII010057490.1

CATCACTTTTAATCTTCTGGAACCCGCCCACCACTGCCCAAGTCATGATTGAAGCACAGCCAACCAAAGTTTCTGAGGGGAAGGATGTTCTTCTACTTGTCCACAATTTGCCCCAGAATCTTGCTGCCTACATCTGGTACAAAGGGCAAATAATGGACATCCACCATTACATTACAGCATATGTAAAAGACACTGATACAATTATATTTGGGCCTGCATACAGTGGAAGAGAAACAGTATATTCCAATGCATCCCTGCTGATCCAGAATGTCACCCAGAAGGACACAGGATCCTACACCATACAAATCATAAAGCGAGGTGATAGGACTGAAGCAGTAACTGGACATTTCACCTTATACT

>Sen_PSG7N (Semnopithecus entellus; Hanuman langur) WGS PVII010041833.1

CATCACTTTTAATCTTCTGGAACCCACCCACCACTGCCCAAGTCACGATTGAAGCCCAGCCAACCAAAGTTTCTGAGGGGAAGGATGTTCTGCTACTTGTTCACAATTTGCCCCAGAATATTGCTGCCTATATCTGGTACAAAGGGCAAATAATGGACCTCCGCCATTACATTACAGCATATATAATAGACACTGAAATGATTATACTTGGGCCTGCATACAGTGGACGAGAAACTGTATATTCCAATGCATCCCTGCTGATCCAGAATGTCACCCAGAATGACACAGGATCCTACACCATTCAAATCACACAGCGAGGTGATGGGATTAAAGGAGTAACTGGACATTTCACCTTATACC

>Sen_PSG8N (Semnopithecus entellus; Hanuman langur) WGS PVII010129675.1

CATCACTTTTAATCTTCTGGAACCCGCCCACCGCTGCCCAAGTCATGATTGAAGCCCAGCCAACCAAAGTTTCTGAGGGGAAGGATGTTCTTCTACTTGTTCACAATTTGCCCCAGAATGTTGCTGCCTACATCTGGTACAAAGGGCAAATAATGGACGTCCACCATTACATTACGGGATATGTAATACACCCTGAAACAATTATATTTGGGCCTGCATACACTGGACGAGAAAGATTATATTCCAACGCATCCCTGCTGATTCAGAAAGTCACTCAGAAGGACACAGGATCCTACACCATAAAAATCACAAAGCGAGGTGATAAGACTAAACGAGTAACTGGACATTTCACCTTATACT

>Sen_PSG9N (Semnopithecus entellus; Hanuman langur) WGS PVII010140745.1

CATCACTTTTAATCTTCTGGAACACGCCCACCACTGCCCAAGTCACGATTGAAGCACAGCCAACCAAAGTATCTGAGGGGAAGGATGTTCTGCTACTTGTCCACAACTTGCCCCAGAATCTTATTGGCTACATCTGGTACAAAGGCCAAGTAATGGACTTCCACCATTACATTACATCATATGTAATAGATGCTGAAACAGTTATAATTGGGCCTGCATACAGTGGACGAGAAACAGTATATTCCAATGCATCCCTGCTGATCCAGAATGTCACCCGGAAGGACACAGGATCCTACACCATACAAATCATAAAGCTAGGTGATAGGACTAAAGGAGTAACTGGACATTTCACCTTATACT

>Sen_PSG10N (Semnopithecus entellus; Hanuman langur) WGS PVII010093332.1

CATCACTTTTAATCTTCTGGAACACGCCCACCACTGCCCAAGTCACGATTGAAGCACAGCCAACCAAATTATCTGAGGGGAAGGATGTTCTGCTACTTGTCCACAACTTGCCCCAGAATCTTATTGGCTACATCTGGTACAAAGGCCAAGTAATGGACCTCCACCATTACATTACATCATATGTAATAGACGTTGAAACAATTATATTTGGGCCTGCATACAGTGGACGAGAAACAGTATATTCCAATGCATCCCTGCTGATCCAGAATGTCACCTGGAAGGACACAGGATCCTACACCATACAAATCATAAAGCTAGGTGATAGGACTAAAGGAGTAACTGGACATTTCACCTTATACT

>Sen_PSG11N (Semnopithecus entellus; Hanuman langur) WGS PVII010061652.1

CATCACTTTTAATCTTCTGGAACCCGCCCACCACTGCCCAAGTCATGATTGAAGCTCAACCAACCAAAGTTTCTGAGGGGAAGGATGTTCTTCTACTTGTCCACAATTTGCCCCAGAATGTTGCTGCCTACGTGTGGTACCAAGGGCAAATAATGGACTTCTACCAATTTATTACAGCATATTCAAGAGACCCTGATAGAATTCTATTTGGGCCTGCATACAGTGGACGAGAAACAGTATATTCCAATGGATCCCTGCGGATCCAGAATGTCACCAAGGAGGACACAGGATCCTACACCGTAAAAGTCATGAAGCGAATTGATGAGACTAAAGGAGTAACTGGACATTTCACCTTATACC

>Sen_PSG12N (Semnopithecus entellus; Hanuman langur) WGS PVII010107177.1

CATCACTTTTAATCTTCTGGAACCCGCCCACCACTGCCCAAGTCATGACTGAAGCTCAACCAACCAAAGTTTCTGAGGGGAAGGATGTTCTATTACTTGTCCACAATTTGCCCCAGAATCCTGCTGCCTACGTCTGGTACAAAGGGCAAATAATGGACTTCTACCAATTCATTATAGCATATACAAGATACCCTGATAGACTTCTATTTGGGCCTGCATACAGTGGACGAGAAACACTATATTCCAATGGATCCCTGCTGATCCAGAATGTCACCAAGCAGGACACAGGATCCTACACCGTAAAAATCATGAAGCGAATTGATGACACTAAAGGAGTAACTGGACATTTCACCTTATACC

>Sen_PSG13N (Semnopithecus entellus; Hanuman langur) WGS PVII010157427.1

CATCACTTTTATTCTTCTGGAACCCGCCCACCACAGCCCAAGTCATGATTGAAGCACAGCCAACCAAAGTTTCCGACGGGAAGGATGTTCTTCTACTTGTTCACAATTTGCCCCAGAATGTTGCTGCCTACATCTGGTACAAAGGGCAAATCATAGACCTCCACCATTACATTATGGGATATGTAATAGAGGCTGAAGCAATTTTATTTGGGCCTGCATACAGTGGACGAGAAACAGTATATTCCAATGCATCCCTGCTGATCCAGAATGTCACCCAGAAGGACACAGGATCCTACACCATACAAATCATAAAGCGAGGTGATAAGACTAAAGGAGTAACTGGACATTTCACGTTATACT

>Sen_PSG14N (Semnopithecus entellus; Hanuman langur) WGS PVII010040921.1

CATCACTTTTAATCTTCTGGAACCCGCCCACCACTGCTCAAGTCACAATTGAAGCTCAGCCAACCAATGTTTCTGAGGGGAACGATGTTCTTCTACTTGTTCACAATTTGCCCCAGAATCCTGCTGCCTACATCTGGTACAAAGGGCAAATAATGGATCTCCACCATTACATTACAGCATATGTAATAGAAACTGAAAGAATTGTATTTGGGCCTGCATACAGTGGACGAGAAACAGTATATTCCAATGCATCCCTGCTGATCCAGAGTGTCAACCAGAAGGACGCAGTATCCTACACCGTAGAAATCATAAAGCGAGGTGATGGGAATGAAGGAGTAACTGGAAATTTCACCTTATACT

>Sen_PSGP1N (Semnopithecus entellus; Hanuman langur) WGS PVII010005485.1 no ORF

CATCACTTTTAATCTTCCGGAACCCGCCCACCACTGCCAAGTCATGATTGAAGCCCAGCCAACCAAAGTTTCTGAGGGGAAGGATGCTCTTCTACTTGTCCAGAATTAGCCCCAGAATCTTACTGGCTACATCTGGTACAAAGGGCAAATAATGGACCTCCACCAATTCATTACAGCATATACAATAGACACGGATACAATTATATTTGGGCCTGCATACAGTGGACGAGAAACAGTATATTCCAATGCATCCCTGCTGATCCAGAATGTCACCCAAAGTGACACAGGATCCTACACCATACAAATCATAAAGCGAGGTGATAAGATTAAACGAGTAACTGGACATTTCACCTTATACC

>Sen_PSGP2N (Semnopithecus entellus; Hanuman langur) WGS PVII010015303.1 no ORF

CATCACTTTAAATCTTCTGGAACTCACCCACCATGGCCCAAGTCACGTATGAAGCCCAGCCAACCAAAGTTTCTGAGGGGAAGGATGTTCTGCTACTTGTCTACAATTTGCCCCAGAATGTTGCGGCCTACATCTGGTACAAAGGGCAAATAATGGACGTCCACCATTACATTACAGCATATACAATAGACACTGAAATGATTATATTTGGGCCTGCATACAGTGGATGAGAAACTGTATATCCCAATGCATCCCTGTTGATCCAGAATGTCACCCAGAAGGACACAGAATCCTACACCATTCAAATCACACAGCGAGGTGATGGGACTAAAGGAGTAACTGGACATTTCACCTTATACC

>Sim_PSG1N (Saguinus imperator; tamarin) WGS PVHO010374269.1

CATCACTTTTAAACTTCTGGAACCCACCCACCACTGCCCAAGTCATGATTGAAGCCCAGCCACACGTTGTTTCAGAGGGGAAGGATGTTCTTCTGCTTGTCCACAATTTGCCCCAGAATCCTACTGGCTACAGCTGGTACAGAGGGAAAATTAAGGACATCAACCATTACATTACAGCTTATTTAATAGACACTGAGATAACTATATTTGGGCCTGCACACACTGGACGAGAAACAATATATCCCAATGCATCCCTGCTGATTGAGAACGTCACCCAGAATGACGCAGGACTCTACACCCTACAAGTCACCACGCAAGGTGATAGGAATAAAGGAGAAACTGGACATTTCACCTTACACC

>Sim_PSG2N (Saguinus imperator; tamarin) WGS PVHO010111679.1

CATCACTTTTAAACTTCTGGAACCCACCCACCACTGCCCAAGTCATGATTGAAGCCCAGCCACACGTTGTTTCAGAGGGGAAGGATGTTCTTCTGCTTGTCCACAATTTGCCCCAGAATCTTACTGGCTACAGCTGGTACAGAGGGAATATCATGGACATCAAACATTACATTACAGCATTTTTAATAGAACGTCACATAACTATATTTGGGCCTGCACACACTGGACGAGAAACAATATATCCCAATGCATCCCTGCTGATTGAGAACGTCAACCATAATGACGCAGGACTCTACACCCTACAAGTCGCCACGCAAGGTGATAGGAATAAAGGAGAAACTGGACATTTCACCTTACACC

>Tfr_PSG1aN (Trachypithecus francoisi; Francois's langur) WGS VVIV01000998.1

CATTACTTTTAATCTTCTGGAACCCGCCCACCACTGCTCAAGTCACGATTGAAGCCCAGCCAACCAAAGTTTCTGAGGGGAAGGATGTTCTTCTACTTGTCCACAATTTGCCCCAGAATCTTACTGGCTACATCTGGTACAAAGGGCAAATAATGGACCTCTACCATTACATTACAGCATATACAATAGACACTGAAATAATTATATTTGGGCCTGCATACAGTGGACGAGAAACAGTATATTCCAATGCATCCCTGCTGATCCAGAATGTCACCCAGAAGGACACAGGATCCTACACCATAAAAATCATAAAGCGAGGTGATAGGACTGAAGGAGTAACTGGACATTTCACCTTATACC

>Tfr_PSG1bN (Trachypithecus francoisi; Francois's langur) WGS QXJD01001741.1

CATTACTTTTAATCTTCTGGAACCCGCCCACCACTGCTCAAGTCACGATTGAAGCCCAGCCAACCAAAGTTTCTGAGGGGAAGGATGTTCTTCTACTTGTCCACAATTTGCCCCAGAATCTTACTGGCTACATCTGGTACAAAGGGCAAATGATGGACCTCTACCATTACATTACAGCATATACAATAGACACTGAAATAACTATATTTGGGCCTGCATACAGTGGACGAGAAACAGTATATTCCAATGCATCCCTGCTGATCCAGAATGTCACCCAGAAGGACACAGGATCCTACACCATAAAAATCATAAAGCGAGGTGATAGGACTGAAGGAGTAACTGGACATTTCACCTTATACC

>Tfr_PSG2N (Trachypithecus francoisi; Francois's langur) WGS VVIV01000998.

CATCACTTTTAATCTTCTGGAACCCACCCACCACTGCCCAAGTCATGATTGAAGCTCAACCAACCAAAGTTTCTGAGGGGAAGGATGTTCTTCTACTTGTCCACAATTTGCCCCAGAATGTTGCTGCCTACGTGTGGTACCAAGGGCAAATAATGGACTTCTACCAATTTATTACAGCATATTCAAGAGACCCTGATAGAATTCTATTTGGGCCTGCATACAGTGGACGAGAAACTGTATATTCCAATGGATCCCTGCGGATCCAGAATGTCACCAAGCAGGACACGGGATCCTACACCGTAAAAGTCATGAAGCGAATTGATGAGACTAAAGGAGTAACTGGACATTTCACCTTATACC

>Tfr_PSG3N (Trachypithecus francoisi; Francois's langur) WGS QXJD01001741.1

CATCACTTTTAATCTTCTGGAACCCACCCACCACTGTGCAAGTCATGATTGAAGCCCAGCCAACCAAAGTTTCTGAGGGGAAGGATGTTCTGCTACTTGTCCACAATTTGCCCCAGAATCTTGCTGGATACATCTGGTACAAAGGGCAAATAATGGACCTCTACCATTACATTACAGCATATACAATAGACACTGAAATGATTATATTTGGGCCTGCATACAGTGGACGAGAAACAGTATATTCCAATGCATCCCTGCTCATCCAGAATGTCACCCAAAATGACACAGGATCCTACACCATACAAATCATAAAGCGAGGTGATAAGACTAAAGGAGTAACTGGACATTTCACCTTATACC

>Tfr_PSG4N (Trachypithecus francoisi; Francois's langur) WGS QXJD01001741.1

CATCACTTTTAATCTTCTGGAACTCACCCACCACTGCCCAAGTCACGATTGAAGCCCAGCCAACCAAAGTTTCTGAGGGGAAGGATGTTCTGCTGCTTGTCCACAATTTGCCCCAGAATGTTATTGGCTACGTCTGGTACAAAGGGCAAATAATGGACCTCTACCATTACATTACAGCATATATAATAGACACTGAAATGATTATATTTGGGCCTGCATACAGTGGACGAGAAACTGTATATTCCAATGCATCCCTGCTGATCCAGAATGTCACCCAGAATGACACAGGATCCTACACCATTCAAATCACACAGCGAGGTGATGGGATTAAAGGAGTAACTGGACATTTCACCTTATACT

>Tfr_PSG5N (Trachypithecus francoisi; Francois's langur) WGS QXJD01001741.1

CATCACTTTTAATCTTCTGGAACCCACCCACCACTGCCCAAGTCATGATTGAAGCACAGCCAACCAAAGTTTCTGAGGGGAAGGATGTTCTTCTACTTGTCCACAATTTGCCCCAGAATCTTGCTGCCTACATCTGGTACAAAGGGCAAATAATGGACCTCCACCATTACATTACAGCATATGTAAAAGACACTGATACAATTATATTTGGGCCTGCATACAGTGGACGAGAAACAGTATATTCCAATGCATCCCTGCTGATCCAGAATGTCACCCGGAAGGACACAGGATCCTACACCATACAAATCATAAAGCTAGGTGATAGGACCAAAGGAGTAACTGGACATTTCACCTTATACT

>Tfr_PSG6N (Trachypithecus francoisi; Francois's langur) WGS QXJD01001741.1

CATCACTTTTAATCTTCTGGAACCCACCCACCACTGCCCAAGTCACGATTGAAGCCCAGCCAACCAAAGTTTCTGAGGGGAAGGATGTTCTGCTACTTGTTCACAATTTGCCCCAGAATATTGCTGCCTATATCTGGTACAAAGGGCAAATAATGGACCTCCGCCATTACATTACAGCATATATAATAGACACTGAAATGATTATACTTGGGCCTGCATACAGTGGACGACAAACTGTATATTCCAATGCATCCCTGCTGATCCAGAATGTCACCCAGAATGACACAGGATCCTACACCATTCGTATCACACAGCGAGGTGATGGAACTAAAGGAGTAACTGGACATTTCACCTTATACC

>Tfr_PSG7N (Trachypithecus francoisi; Francois's langur) WGS QXJD01001741.1

CATCACTTTTAATCTTCTGGAACACGCCCACCACTGCCCAAGTCACGATTGAAGCACAGCCAACCAAAGTATCTGAGGGGAAGGATGTTCTGCTACTTGTCCACAACTTGCCCCAGAATCTTATTGGCTACATCTGGTACAAAGGCCAAGTAATGGACTTCCACCATTACATTACATCATATGTAATAGATGCTGAAACAGTTATAATTGGGCCTGCATACAGTGGACGAGAAACAGTATATTCCAATGCATCCCTGCTGATCCAGAATGTCACCCGGAAGGACACAGGATCCTACACCATACAAATCATAAAGCTAGGTGATAGGACCAAAGGAGTAACTGGACATTTCACCTTATACT

>Tfr_PSG8N (Trachypithecus francoisi; Francois's langur) WGS QXJD01001741.1

CATCACTTTTACTCTTCTGGAACCCGCCCACCACTGCCCAAGTCACGATTGAAGCCCAGCCAACCAAAGTTTCCGATGGGAAGGATGTTCTTCTACTTGTTCACAATTTGCCCCAGAATGTTGCTGCCTACATCTGGTACAAAGGGCAAATCATAGACCTCCACCATTACATTATGGGATATGTAATAGAGGCTGAAGCAATTTTATTTGGGCCTGCATACAGTGGACGAGAAACAGTATATTCCAATGCATCCCTGCTGATCCAGAATGTCACCCAGAAGGACACAGGATCCTACACCATACAAATCATAAAGCGAGGTGATAAGACTAAAGGAGTAACTGGACATTTCACGTTATACT

>Tfr_PSG9N (Trachypithecus francoisi; Francois's langur) WGS QXJD01001741.1

TATTACTTTTCATCTTCTGGAACCCACCCACCACTGCCCAAGTCACGATTGAAGCTCAGCCAACCAAAGTTTCCGAGGGGAAGGATGTTCTTCTACTTGTCCACGATTTGCCCCAGAATCTTACTGGCTACATCTGGTACAAAGGACAAATAATGGACCTCCACCAATTCATTACAGCATATACAATAGACACTGAAACAATTATCTTTGGGCCTGCATACAGTGGACGAGAAACAGTATATTCCAATGCATCCCTGCTGATCCGGAATGTCACCCAGAATGACACAGGATCCTACATGATACAAATCATAAAGCGAGGTGATAAGATTAAACGAGTAACTGGACATTTCACCTTATACC

>Tfr_PSG10N (Trachypithecus francoisi; Francois's langur) WGS QXJD01001741.1

CATCACTTTTAATCTTCTGGAACGTGCCCACCACTGCTCAAGTCACGACTGAAGCTCAGCCAACCAAAGTCTCTGAGGGGAAGCATGTTCTACTTGTCCACAATTTGCCCCAGAATGTTGTTGGCTACATCTGGTACAAAGGGCAAATAATGGACGTCCACCAATTCATTACAGCATATACAATAGACACTGAAACAATTATATTTGGGCCTGTATATAGTGGACGAGAAAAAATATATTCCAACGCATCCCTGCTGATCCAGAATGTCACCCAGAATGACACAGGATCCTACACCATAAAAATCATAAAGCGAGGTGATGAGACTAAAGAAGTAACTGGACATTTCACCTTATACC

>Tfr_PSG11N (Trachypithecus francoisi; Francois's langur) WGS QXJD01001741.1

CATCATTTTTAATCCTCTGGAACCTGCCTACCATGGCCCACGTCATGATTGAAGCTCAGCCAACCGAAGTTTCTGAGGGGAAGGATGTTCTTCTACTTGTCCACAATTTGCCCCAGAATCCTACTGGCTACATCTGGTACAAAGGGCAAATAACGGATATCCACAATTACATTACATCATATGTAATAGACACTGATACAATTATATTTGGGCCTGCATACAGTGGACGAGAAACAGTATATTCCAATGCATCCCTGCTGATCCAGAATGTCACCCGGAAGGACACAGGATCCTACACCATACAAATCATAAAGCGAGGTGATAGCACTAAAGGAGTAACTGGACATTTCACCTTATACT

>Tfr_PSG12N (Trachypithecus francoisi; Francois's langur) WGS QXJD01001741.1

CATCACTTTTAATCTTCTGGAACCCGCCCACCGCTGCCCAAGTCATGATTGAAGCCCAGCCAACCAAAGTTTCTGAGGGGAAGGATGTTCTTCTACTTGTTCACAATTTGCCCCAGAATGTTGCTGCCTACATCTGGTACAAAGGGCAAATAATGGACGTCCACCATTACGTTACGGGATATGTAATACACCCTGAAACAATTATATTTGGGCCTGCATACACTGGACGAGAAAGATTATATTCCAACGCATCCCTGCTGATTCAGAATGTCACCCAGAAGGACACAGGATCCTACACCATAAAAATCACAAAGCGAGGTGATAAGACTAAACGAGTAACTGGACATTTCACCTTATACT

>Tfr_PSG13N (Trachypithecus francoisi; Francois's langur) WGS QXJD01001741.1

CATCACTTTTAATCTTCTGGAACCCGCCCACCACTGCTCAAGTCACAATTGAAGCTCAGCCAACCAATGTTTCTGAGGGGAACGATGTTCTTCTACTTGTTCACAATTTGCCCCAGAATCCTGCTGCCTACATCTGGTACAAAGGGCATTTAATGGATCTCCACCATTACATTACAGCATATGTAATAGAAACTGAAAGAATTGTATTTGGGCCTGCATACAGTGGACGAGAAACAGTATATTCCAATGCATCCCTGCTGATCCAGAGTGTCAACCAGAAGGACGCAGTATCCTACACCGTAGAAATCATAAAGCAAGGTGATGGGAATGAAGGAGTAACTGGAAATTTCACCTTATACT

>Tfr_PSG14N (Trachypithecus francoisi; Francois's langur) WGS QXJD01001741.1

CATCACTTTTAATCTTCTGGAACCCGCCCACCACTGCCCAAGTCATGATTGAAACTCAACCAACCAAAGTTTCTGAGGGGAAGGATGTTCTTTTACTTGTCCACAATTTGCCCCAGAATCCTGCTGCCTACGTCTGGTACAAAGGGCAAATAATGGACTTCTACCAATTCATTATAGCATATACAAGATACCCTGATAGAATTCTATTTGGGCCTGCATACAGTGGACGAGAAACACTATATTCCAATGGATCCCTGCTGATCCAGAATGTCACCAAGCAGGACACAGGATCCTACACCGTAAAAATCATGAAGCGAATTGATGACACTAAAGGAGTAACTGGACATTTCACCTTATACC

>Tfr_PSG15N (Trachypithecus francoisi; Francois's langur) WGS QXJD01001960.1

CATCACTTTTAATCTTCTGGAACCCGCCCACTGCTGCCCAAGTCATGATTGAAGCCCAGCCAACCAAAGTTTCTGAGGGGAAGAATGTTCTTCTACTTGTCCACAATTTGCCCCAGAATGTTGCTGCCTACATCTGGTACAAAGGGCAAATAATGGACGTCCACCATTACATTACGGGATATGTAATGGAGGCTGAAGCAGTTATATTTGGGCCTGCATACAGTGGACGAGAAACAGTATATTCCAATGCATCCCTGCTGATTCAGAATGTCACCCAGAAGGACACAGGATCCTACACCATACAAATCATAAAGCGAGGTGATAGGATTGAAGGAGTAACTGGACATTTCACCTTATACC

>Tfr_PSGP1N (Trachypithecus francoisi; Francois's langur) WGS VVIV01000998.1 no ORF

CATCACTTTCAATCTTCTGGAACCCGCTCACCACTGCCCAAGTCACGACTGAATCTCAGCCAACCAAAGTTTCTGAAGGGAAGGATATTCTTCTACTTGTCCACAATTTGCCCCAGAATCCTGCTGCCTACTTCTGGTACAAAGGGCAAATAATGGACCTCCACCATTACATTCAGCATATACAACAGACACTGAAAGAGTTATATTTGGGCCTGCATACAGTGCATGAGAAACTGTATATTCCAATGCTTCCCTGCTGATCCAGAGTGTCACCTAGAAGGATGTAGGATCCTACACCATGGAAATCATAAAGCAAGGTGATAGGACTGAAGGAGTAACTGGACATTTCACCTTATACC

>Tfr_PSGP2N (Trachypithecus francoisi; Francois's langur) WGS QXJD01001741.1 no ORF

CATCACTTTTAATCTTCTGGAACCCGCCCACCGCTGCTCAAGTCACGATTGAAGCCCAGCCAACCAAAGTTTCTGAGGGGAAGGATGTTCTTCTACTTGTCCACAATTTGCCCCAGAATTTTACTGGCTACATCTGGTACAAAGGGCAAATAACGGACCTCCACCATTACATTACATCATATGTAATAGACTTGAAACAATTATATTTGGGCCTGCATACAGTGGACGAGAAACAGTATATTCCAATGCATCCCTGCTGATCCAGAATGTCACCTGGAAGGACACAGGATCCTACACCATACAAATCATAAAGCGAGGTGATAGGAGTGAAGGAGTAACTGGACATTACACGTTATACC

>Tfr_PSGP3N (Trachypithecus francoisi; Francois's langur) WGS QXJD01001741.1 no ORF

CATCACTTTTAATCTTCCGGAACCCGCCCACCACTGCCCAAGTCATGATTGAAGCCCAGCCAAACAAAGTTTCTGAGGGGAAGGATGCTCTTCTACTTGTCCAGAATTAGCCCCAGAATCTTACTGGCTACATCTGGTACAAAGGGCAAATAATGGACCTCCACCAATTCATTACAGCATATACAATAGACACTGATACAATTATATTTGGGCCTGCATACAGTGGACAAGAAACAGTATATTCCAATGCATCCCTGCTGATCCAGAATGTCACCCAAAATGACACAGGATCCTACACCATACAAATCATAAAGCGAGGTGATAAGATTAAACGAGTAACTGGACATTTCACCTTATACC

>Tge_PSG1N (Theropithecus gelada; gelada baboon) WGS QGDE01000020.1

CATCACTTTTAATCTTCTGGAACCTGCCCACCACTGCCCAAGTCACGATTGAAGCACAGCCAACCAAAGTTTCTGAGGGGAAGGATGTTCTGCTACTTGTCCACAATTTGCCCCAGAATGTTGCTGGCTACAGCTGGTACAAAGGGCAAATAATGGACCTCCACCATTACATTACAGCATATACAATAGACACTGAAATGATTATAGTTGGGCCTGCATACAGTGGACGAGAAACAGTATATTCCAATGCATCCCTGCTGATCCAGAATGTCACCCAGAAGGACACAGGATCCTACACCATACAAATCATAAAGCGAGGTGATGGGACTAAAAGAGTAACTGGACATTTCACTTTATACC

>Tge_PSG2N (Theropithecus gelada; gelada baboon) WGS QGDE01000020.1

CATCACTTTTAATCTTCTGGAACACGCCCACCACTGCCCAAGTCACGATTGAAGCCCAGCCAACCAAAGTTTCTGAGGGGAAGGATGTTCTGCTACTTGTCCACAATTTGCCCCAGAATCCTACTGGCTACATCTGGTACAAAGGGCAAATAATGGACCTCCACCATCACATTACATCATATGTAGTAGACACTGAAATAATTGTATTTGGGCCTGCATACAGTGGACGAGAAACAGTATATTCCAATGCATCCCTGCTGATCCAGAATGTCACCCAGAAGGACACAGGATCCTACACCATACAAATCATACAGCGAGGTGATACCACTAAAGGAGTAACTGGACATTTCACCTTATACC

>Tge_PSG3N (Theropithecus gelada; gelada baboon) WGS QGDE01000020.1

CATCACTTTTCATCTTCTGGAACCCACCTATCGCGGCCCAAGTCACGATTGAAGCTCAGCCAACCGAAGTTTCTGAGGGGAAGGATGTTCTTCTACTTGTCCACAATTTGCCCCAGAATCCTACTGGCTACATCTGGTACAAAGGGCAAATAACGGATAGCCACAATTACATTACATCATATGTAATAGACACTGAAATGATTATATTTGGGCCTGCATACAGTGGACGAGAAACAGTATATTCCAATGCATCCCTGCTGATCCAGAATGTCACCCGGAAGGACACAGGATCCTACACCATACAAATCATAAAGCGAGGTGATAGCACTAAAGGAGTAACTGGACATTACACCTTATACC

>Tge_PSG4N (Theropithecus gelada; gelada baboon) WGS QGDE01000020.1

CATCACTTTTCATCTTCTGGAACCCACCCACCACTGCCCAAGTCACGATTGAAGCTCAGCCAACCAAAGTTTCCGAGGGGAAGGATGTTCTTCTACTTGTCCAGAATTTGCCCCAGAATCTTACTGGCTACGTCTGGTACAAAGGGCAAAAAACGGACCTCCACCAATTCATTACAGCGTATACGATAGACACTGAAACAATTATATCTGGGCCTGCATACAGTGGACGAGAAACAGTATATTCCAATGCATCCCTGCTGATCCAGAATGTCACCCGGAATGACACAGGATCCTACACCATACAAATCATACAGCGAGGTGATAAGATTAAAAGAATAACTGGACATTTCACCTTATACC

>Tge_PSG5N (Theropithecus gelada; gelada baboon) WGS QGDE01000020.1

CATCACTTTTAATCTTCTGGAACTCGCCTACCACTGCCCAAGTCACAATTGAAGCTCAGCCAACCAATATTTCCGAGGGGAACGATGTTCTTCTACTTGTACACAATTTGCCCCAGAATCCTGCTGCCTACATCTGGTACAAAGGGCAAATAATGGACCTCCACCATTACATTACAGCATATACAATAGACACTGAAAGAATTATATTTGGGCCTGCATACAGTGGACGAGAAAGAGTATATTCCAATGCATCCCTGCTGATCCAGAGTGTGAACCAGAAGGACGCAGGATCCTACACCATAAAAATCATAAAGCGAGGTGACGGGACTGAAGGAGTAACTGGACATTTCACCTTATAtg

>Tge_PSG6N (Theropithecus gelada; gelada baboon) WGS QGDE01000020.1

CATCACTTTTAATCTTCTGGAACCCGCCCACCACTGCTCAAGTCACAATTGAAGCCCAGCCAGCCAAAGTTTCTGAGGGAAAGGATGTTCTTCTACTTGTCCACAATTTGCCCCAGAATCTTGCTGCCTGCATCTGGTACAAAGGGCAAATAATGGACCTCCAACATTACATTACAGCATATGTAATAGATGCTGAAACAATTATATTTGGGCCTGCCTACAGTGGACGAGAAACAGTATATTCCAATGCATCCCTGCTGATCCAGAATGTCACCCAGAAGGACACAGGATCCTACACCATACAAATCATACAGCGAGGTGATAAGACTAAAGGAGTAACTGGACATTTCACCTTATAtg

>Tge_PSG7N (Theropithecus gelada; gelada baboon) WGS QGDE01000020.1

CATCACTTTTAATCTTCTGGAACACACCCACCACTGCCCAAGTCACGATTGAAGCACAGCCAACCAAAGTTTCCGAGGGGAAGGATGTTCTTCTACTTGTCCAGAATTTGCCCCAGAATCTTATTGCCTACATCTGGTACAAAGGGCAAAAAACGGACTTCCGCCATTACATTACATCATATGTAATAGATGCTGAAACAATTATAGTTGGGCCTGCATACAGTGGACGAGAAACAGTATATTCCAATGCATCCCTGCTGATCCAGAATGTCACCCAGAAGGACACAGGATCCTACACCATACAAATGATAAAGCAAGGTGATAAGACTAAAGGAGTAATTGGACATTTCACCTTATACC

>Tge_PSG8N (Theropithecus gelada; gelada baboon) WGS QGDE01000020.1

CATCATTTTTAATCTTGTGGAACCCGCCCACCACTGCCCAAGTCATGATTGAAGCTCAGCCAACCAAAGTTTCTGAGGGGAAGGATGTTCTTCTACCTGTCCGCAATTTGCCCCAGGAAGTTGCTGCCTATATCTGGTACAAAGGGCAAATAATGGACTTCCACCAATTCATTACAGCATATACAATAGACACTGAAAGAATTATATTTGGGCCTGCATACAGTGGACGAGAAACAGTATATTCCAATGGATCCCTGCTGATCCGGAATGTCACCAAGCAGGACACAGGATCCTACACCGTAAAAATCAGGAAGCCAGCTGAGGAGACTAAAGGAGTAACTGTACATTTCACCTTATAtg

>Tge_PSG9N (Theropithecus gelada; gelada baboon) WGS QGDE01000020.1

CATCACTTTTAATCTTCTGGAACGCGCCCACCACTGCTCAAGTCACAATTGAAGCCCAGCCAACCAAAGTTTCCGAGGGGAAGGATGTTCTTCTACTTGTCCACAATTTGCCCCAGAATCTTACCGGCTACATCTGGTACAAAGGGCAAAAAACGGACCTCCACCTTTATGTTACATCATATGTAAAAGACACTGAAACAGTTATAGCTGGGCCTGCATACAGTGGACGAGAAACAGTATATTCCAATGCATCCCTGCTGATCCAGAATGTCACCCAGAAGGACACAGGATCCTACACCATAGAAATCACAAAGCGAGGTGATAGGACTGAAGGAGAAATTGGACATTTCACCTTATAtg

>Tge_PSG10N (Theropithecus gelada; gelada baboon) WGS QGDE01002138.1

TATCACTTTTAATCATCTGGAACCCACCTACCACGGGTCAAGTCACGATTGAAGCTCAGCCAACCGAGGTTTCTGAGGGGAAGGATGTTCTTCTACTTGTCCACAATTTGCCCCAGAATCCTACTGGCTACAGCTGGTACAAAGGGCAAATAACAGACCTCCACCATTACATTACATCATATGTAATAGACACTGAAATGATTGTATTTGGGCCTGCATACAGTGGACGAGAAACAGTATATTCCAATGCATCCCTGCTGATCCAGAATGTCACCCAGAAGGACACAGGATCCTACACCATAGAAATCATACAGCGAGGTGATACCACTAAAGGAGTAACTGGACATTTCACCTTATACG

>Tge_PSG11N (Theropithecus gelada; gelada baboon) WGS QGDE01003938.1

CATCACTTTTAATCTTCTGGAACCCACCCACCACTGCCCAAGTCACAATTGAAGCCCAGCCAGCCAAAGTTTCCGAGGGGAAGGATGTTCTTCTACTTGTCCAGAATTTGCCTGAGAATCTTACTGGCTACGTCTGGTTCAAAGGGCAAATAATGGACTTCCACCAATTCATTACAGCGTATACAATAGACACTGAAACAATTATATTTGGGCCTGCATACAGTGGACGAGAAACAGTATATTCCAATGCATCCCTGCTGATCCAGAATGTCACCCAGAATGACACAGGATCCTACACCATAGAAATTATAAAGCGAGGTGATAAGATTAAAGGAGTAACTGGACATTTCACCTTATACC

>Tge_PSG12N (Theropithecus gelada; gelada baboon) WGS QGDE01002154.1

CATCACTTTTAATCTTCTGGAACCCACCCACCGCTGCTCAAGTCACGATTGAAGCCCAGCCAACCAAAGTTTCCGAGGGGAAGGATGTTCTTCTACTTGTCCACAATTTGCCCCAGAATCTTACTGGCTACATCTGGTACAAAGGCCAAAAAATGGACCACCACCATTACATTACATCATATGTGATAGACACTGAAACAATTATATTTGGGCCTGCATACAGTGAACGAGAAACAGTATATTCCAATGCATCCCTGCTGATCCAGAATGTCACCAAGAATGACACAGGATCCTACACCATACAAATCATAAAGCGAGGTCATAGGACTGAAGGAGTAACTGGACATTACACCTTATCCC

>Tge_PSG13N (Theropithecus gelada; gelada baboon) WGS QGDE01001817.1

CATCACTTTTAATCTTCTGGAACTCGCCCACCACTGCCCAAGTCACAATTGAAGCTCAGCCAACCAATATTTCCGAGGGGAATGATGTTCTTCTACTTGTGCACAATTTACCCAAGAATCCTGCTGCCTACATCTGGTACAAAGGGCAAATATTGGACCTCCACCATTACATTACAGCATATACAGTAGACACTGAAAGAATTATATTTGGGTCTGCATACAGTGGACGAGAAAGAGTATATTCCAATGCATCCCTGCTGATCCAGAGTGTGAACCAGAAGGACGCAGGATCCTACACCGTACAAATCATAAAGCAAGGTGACAGGACTGAAGGAGTAACTGGACATTTCACCTTATATG

>Tge_PSGP1N (Theropithecus gelada; gelada baboon) WGS QGDE01000020.1 no ORF

CATCACTTTTAATCTTCTGGAACCCACCCACCACTGCTCAAGTCACAATTGAAGCCCAGCCAACCAAAGTTTCCGAGGGGAAGGATGTTCTTCTACTTGTCCACAATTTGCCCCAGAATCTTACTGGCTACATCTGATACAAAGGGCAAAAAACGGACCACCACCTTTACATTACATCATATGTAATAGACGCTGAAACAATTATATTTGGGCCTGCATACAGTTGACGAGAAACAGTATATTCCAATGCATCCCTGCTGATCCAGAATGTCACCCGGAAGGACACAGGATCCTACACCATAGAAATCATAAAGCGAGGTGATAGGACTGAAGGAGAAACTGGACATTACACCTTATACC

**Partial primate PSG N exon sequences**

**(number of nnnnn were adjusted based on multi-alignment)**

>Can_PSGP1N (Colobus angolensis palliatus; black and white colobus monkey) WGS JYKR01104454.1

nnnnnnnnnnnnnnnnnnnnnnnnnnnnnnnnnnnnnnnnnnnnnnnnnTGAAGCCCAGCCAACCAAAGTTTCTGAGGGGAAGGATGCTCTGCTACTTGTCCACAATTTGCCCCAGAATGCTGCTGCCTACGTCTGGTACAAAGAGCAAATAATGGACCTCCACCATTACATTACAGCATATACAATAGACACTGAAATGATTATATTTGAGCCTACATACAGTGGATGAGAAAATGTATATCCCAATGCATCCCTGTTGATCCAGAATGTCACCCAGAATGACACAGGATCCTACACCATTCAAATCACACAGCGAGGTGATGGGACTAAATGAGTAACTGGACATTTCACCTTATACC

>Can_PSGP5N (Colobus angolensis palliatus; black and white colobus monkey) WGS JYKR01145170.1

nnnnnnnnnnnnnnnnnnnnnnnnnnnnnnnnnnnnnnnnnnnnnnnnnnnnnnnnnnnnnnnnnnnnnnnnnnnnnnnnnnnnnTGCTCTGCTACTTGTCCACAATTTGCCCCAGAATGCTGCTGCCTACGTCTGGTACAAAGGGCAAATAATGGACCTCCACCATTACATTACAGCATATACAATAGACACTGAAATGATTATATTTGGGCCTACATACAGTGGATGAGAAAATGTATATCCCAATGCATCCCTGTTGATCCAGAATGTCACCCAGAATGACACAGGATCCTACAGCATTCAAATCACACAGCGAGGTGATGGGACTAAATGAGTAACTGGACATTTCACCTTATACC

>Cne_PSG21N (Cercopithecus neglectus; De Brazza's monkey) WGS PVKI011047635.1

nnnnnnnnnnnnnnnnnnnnnnnnnnnnnnnnnnnnnnnnnnnnnnnnnnnnnnnnnnnnnnnnnnnnnnnnnnnnnnnnnnnnnnnnnnnnnnnnnnnnnnnnnnnnnnnnnnnnAATCTTACTGGCTACATCTGGTACAAAGGGCAAATAATGGACCTCCAGCATTACATTACAGCATATACAATAGACACTGAAATGATTATATTTGGGTCTGCATACAGTGGACGAGAAACAGTATATTCCAATGCATCCCTGCTGATCCAGAATGTCACCAAGAATGACACAGGATCCTACACCATTCAAATCACAAAGCAAGGTGATGAGACTAAAGGAGTAACTGGACATTTCACCTTATACC

>Cne_PSG22aN (Cercopithecus neglectus; De Brazza's monkey) WGS PVKI011006061.1

nnnnnnnnnnnnnnnnnnnnnnnnnnnnnnnnnnnnnnnnnnnnnnnnnnnnnnnnnnnnnnnnnnnnnnnnnnnnnnnnnnnnnnnnnnnnnnnnnTGTCCACAATTTGCCCCAGAATCTTGCTGCCTGCATCTGGTACAAAGGGCAAATAATGGACCTCCAACATTACATTACAGCATATGTAATAGATGCTGAAACAATTATATTTGGGCCTGCATACAGTGGACGAGAAAGAGTATATTCCAATGCATCCCTGCTGATCCAGAATGTCACCCAGAAGGACACAGGATCCTACACCATACAAATCATAACGCGAGGTGATAAGACTAAAGGAGTAACTGGACATTTCACCTTATACC

>Cne_PSG22bN (Cercopithecus neglectus; De Brazza's monkey) WGS PVKI010843863.1

nnnnnnnnnnnnnnnnnnnnnnnnnnnnnnnnnnnnnnnnnnnnnnnnnnnnnnnnnnnnnnnnnnnnnnnnnnnnnnnnnnnnnnnnnnnnnnnnnTGTCCACAATTTGCCCCAGAATCTTGCTGCCTGCATCTGGTACAAAGGGCAAATAATGGACCTCCAACATTACATTACAGCATATGTAATAGATGCTGAAACAATTATATTTGGGCCTGCATACAGTGGACGAGAAACAGTATATTCCAATGCATCCCTGCTGATCCAGAATGTCACCCAGAAGGACACAGGATCCTACACCATACAAATCATAAAGCGAGGTGATAAGACTAAAGGAGTAACTGGACATTTCACCTTATACC

>Cne_PSG22cPN (Cercopithecus neglectus; De Brazza's monkey) WGS PVKI011007259.1 completed with PVKI011334196.1

nnnnnnnnnnnnnnnnnnnnnnnnnnnnCACCACTGCCCAAGTTGTGATTGAAGCCCAGCCAACCAAAGTTTCCGAGGGGAAGGATGTTCTGCTACGTGTCCACAATTTGCCCCAGAATCTTGCTGCCTGCATCTGGTACAAAGGGCAAATAATGGACCTCCAACATTACATTACAGCATATGTAATAGATGCTGAAACAATTATATTTGGGCCTGCATACAGTGGATGAGAAACAGTATATTCCAATGCATCCCTGCTGATCCAGAATGTCACCCAGAAGGACACAGGATCCTACACCATACAAATCATAACGCGAGGTGATAAGACTAAAGGAGTAACTGGACATTTCACCTTATACC

>Cne_PSG22dN (Cercopithecus neglectus; De Brazza's monkey) WGS PVKI011374330.1, PVKI010324521.1

nnnnnnnnnnnnnnnnnnnnnnnnnnnnnnnnACTGCCCAAGTTGTGATTGAAGCCCAGCCAACCAAAGTTTCCGAGGGGAAGGATGTTCTGCTACGTGTCCACAATTTGCCCCAGAATCTTGCTGCCTGCATCTGGTACAAAGGGCAAATAATGGACCTCCAACATTACATTACAGCATATGTAATAGATGCTGAAACAATTATATTTGGGCCTGCATACAGTGGACGAGAAAGAGTATATTCCAATGCATCCCTGCTGATCCAGAATGTCACCCAGAAGGACACAGGATCCTACnnnnnnnnnnnnnnnnnnnnnnnnnnnnnnnnnnnnnnnnnnnnnnnnnnnnnnnnnnnnnnnn

>Cne_PSG23N (Cercopithecus neglectus; De Brazza's monkey) WGS PVKI010325439.1

CATCACTTTTAATCTTCTGGAACCTGCCCACTACTGCCCAAGTTGTGATTGAAGCCCAGCCAGCCAAAGTTTCGGAGGGGAAGGATGTTCTTCTACTTGTCCACAATTTGCCCCAGAATCTTGCTGCCTGCATCTGGTACAAAGGGCAAATAATGGACCTCCAACATTACATTACAGCATATGTAATAGATGCTGAAACAATTATATTTGGGCCTGCATACAGTGGAnnnnnnnnnnnnnnnnnnnnnnnnnnnnnnnnnnnnnnnnnnnnnnnnnnnnnnnnnnnnnnnnnnnnnnnnnnnnnnnnnnnnnnnnnnnnnnnnnnnnnnnnnnnnnnnnnnnnnnnnnnnnnnnnnnnnn

>Epa_PSG24N (Erythrocebus patas; red guenon) WGS PVJV010128228.1

CATCACTTTTAATCTTCTGGAACCCGCCCACCACTGCGCAAGTCAGGATTGAAGCTCAGCCAACCAAAGTTTCTGAGGGGAAGGATATTCTTCTACTTGTCCACAATTTGCCCCAGAATGTTGCTGGCTACATCTGGTACAAAGGGCAAATAATGGACCTCCAGTATTACATTACAGCATATGCAATAGACACTGAAATGnnnnnnnnnnnnnnnnnnnnnnnnnnnnnnnnnnnnnnnnnnnnnnnnnnnnnnnnnnnnnnnnnnnnnnnnnnnnnnnnnnnnnnnnnnnnnnnnnnnnnnnnnnnnnnnnnnnnnnnnnnnnnnnnnnnnnnnnnnnnnnnnnnnnnnnnnnnnnnnn

>Hmo_PSG5N (Hylobates moloch; silvery gibbon) XM_032147605.1; WGS WKKJ01000585.1 CATCACTTTTAAACTTCTGGAACCCGCTCACCACTGCCCAAGTCATGATTGAAGCCCAGCCACCCAAAGTTTCCGAGGGGAAGGATGTTCTTCTACTTGTTCACAATTTGCCCCATAATCTTGCCAGCTATAGCTGGCGCAAAGGGCGAACGATGGACCTCCACAATTACATTACATCATATGTAGTAGCCAGTCAAATAATTATATCTGGGCCTGCATACAGTGGACGAGAAACACTATATTCCAATGCATCCCTGCTGATCCAGAATGTCACCCGGGAGGACGnnnnnnnnnnnnnnnnnnnnnnnnnnnnnnnnnnnnnnnnnnnnnnnnnnnnnnnnnnnnnnnnnnnnnnnnnnnnnnnnn

>Mfu_PSG14N (Macaca fuscata fuscata; Japanese macaque) WGS BFBW01079348.1

CATCACTTTTAATCTTCTGGAACTCGCCCACCACTGCCCAAGTCACGATTGAAGCTCAGCCAACCAAAGTTTCTGAGGGGAAGGATGTTCTTCTACTTGTCCACAATTTGCCCACGAACGTTGTTGGCTACATCTGGTACAAAGGGCAAATAATGGACCTCCAGCATTACATTACAGCATATACAACAGACACTGAAATGATTCTATTTGGGCCTGCATACAGTGGACGAGAAACAGTATATTCCAATGCATCCCTGCTGATCCAGAGTGCCACCAAGAATGACACAGGATCCTACACCATACAAATCATAAAGCGAGGTCATAGGACTGAAGGAGTAACTGGACATTnnnnnnnnnnnn

>Mfu_PSG15N (Macaca fuscata fuscata; Japanese macaque) WGS BFBW01048116.1

CATCACTTTTAATCTTCTGGAACCCGCACACCACTGCTCAAGTCACAATTGAAGCCCAGCCAGCCAAAGTTTCTGAGGGGAAGGATGTTCTTCTACTTGTCCACAATTTGCCCCAGAATCTTGCTGCCTGCATCTGGTACAAAGGGCAAATAATGGACCTCCAACATTACATTACAGCATATGTAATAGATGCTGAAACAATTATATTTGGGCCTGCATACAGTGGACGAGAAACAGTATATTCCAATGCATCCCnnnnnnnnnnnnnnnnnnnnnnnnnnnnnnnnnnnnnnnnnnnnnnnnnnnnnnnnnnnnnnnnnnnnnnnnnnnnnnnnnnnnnnnnnnnnnnnnnnnnnnnnn

>Mfu_PSG16N (Macaca fuscata fuscata; Japanese macaque) WGS BFBW01059895.1

CATCACTTTTAATCTTCTGGAACGCGCCCACCACTGCTCAAGTCACAATTGAAGCCCAGCCAACCAAAGTTTCCGAGGGGAAAGATGTTCTTCTACTTGTCCACAATTTGCCCCAGAATCTTACTGGCTACATCTGGTACAAAGGGCAAAAAACGGACTTCCACCTTTACGTTACATCATATGTAAAAGATACTGAAACAGTTATAGCTGGGCCTGCATACAGTGGACGAGAAACAGTATATTCCAATGCATCCCnnnnnnnnnnnnnnnnnnnnnnnnnnnnnnnnnnnnnnnnnnnnnnnnnnnnnnnnnnnnnnnnnnnnnnnnnnnnnnnnnnnnnnnnnnnnnnnnnnnnnnnnn

>Mfu_PSG17N (Macaca fuscata fuscata; Japanese macaque) WGS BFBW01063802.1

CATCACTTTTAATCTTCTGGAACCCGCCCACCACTGCCCAAGTCACGATTGAAGCACAGCCAGCCAAAGTTTCTGAGGGGAAGGATGTTCTTCTACTTGTCCACAATTTGCCCCAGAATCTTACTGGCTACAGCTGGTACAAAGGGCAAATAATAGACCTCCAGCATTACATTACAGCATATACAATAGACACTGAAATGATTGTATTTGGGCCTGCATACAGTGGACGAGAAACAGTATATTCCAATGCATCCCTGCTGATCCAGAATGTCACCAAGAATGnnnnnnnnnnnnnnnnnnnnnnnnnnnnnnnnnnnnnnnnnnnnnnnnnnnnnnnnnnnnnnnnnnnnnnnnnnnnnn

>Msp_PSG6N (Mandrillus sphinx; Mandrill) WGS SRPC01046955.1

CATCACTTTTAATCGTCTGGAACCCGCCCACCACTGCTCAAGTCACAATTGAAGCCCAGCCAGCCAAAGTTTCTGAGGGGAAGGATGTTCTTCTACTTGTCCACAATTTGCCCCAGAATCTTGCTGCCTGCATCTGGTACAAAGGGCAAATAATGGACCTCCAACATTATATTACAGCATATGTAATAGATGCTAAAACAATTATATTTGGGTCTGCATACAGTGGACGAGAAACAGTATATTCCAATGCATCCCTGCTGATCCAGAATGTnnnnnnnnnnnnnnnnnnnnnnnnnnnnnnnnnnnnnnnnnnnnnnnnnnnnnnnnnnnnnnnnnnnnnnnnnnnnnnnnnnnnnnnnn

>Mle_PSGP2N (Mandrillus leucophaeus; drill) WGS JYKQ01030901.1

nnnnnnnnnnnnnnnnnnnnnnnnnnnnnnnnnnnnnnnnnnnnnnnnnnnnnnnnnnnnnnnnnnnnnnnnnnnnnnnnnnnnnnnnnnnnnnnnnnnnnnnnnnnnnnnnnnnnnnnnnnnnnnnnnnnnnnnnnnnCAAAGGGCAAAAAACGGACTTCCGCCATTACATTACATCATATGTAATAGATGCTGAAACAATTATAGTTGGGCCTGCATACAGTGGATGAGAAACAGTATATTCCAATGCATCCCTGCTGATCCAGAATGTCACCCAGAAGGACACAGGATCCTACACCATACAAATGATAAAGCAAGGTGATAAGACTAAAGGAGTAATTGGACATTTCACCTTATACC

>Nla_PSGP1N (Nasalis larvatus; proboscis monkey) WGS JMHX01319548.1

CATCACTTTTAATCTTCTGGAATCCGCCCACCACTGCCCAAGTCATGATTGAAGCACAGCCAAACAAAGTTTCTGAGGGGAAGGATGTTCTTCTACTTGTCCACAATTTGCCCCAGAATCTTGCTGCCTACATCTGGTACAAAGGGCAAATAATGGATCTCCACCATTACATTACAGCATATGTAATAGACACTGATACAATTATATTTGGGCCTGCATACAGTGGACGAGAAACATGTATATTCCAATGCATCCCTGCTGATCCAGAATGTCACCCAGAATGACACAGGATCCTACACCATACAAATCATAAAGCGAGGTGATAGnnnnnnnnnnnnnnnnnnnnnnnnnnnnnnnnnnn

>Nle_PSG10N (Nomascus leucogenys; northern white-cheeked gibbon) WGS ADFV01127705.1

CATCACTTTTAAACTTCTGGAACCCGCCCACCACTGCCCAAGTCACTATTGAAGCCCAGCCACCCAAACTTTCTGAGGGGAAGGACGTTCTTCTACTTGTCCACAATTTGCCCCAGAATCTTACTGGCTACACCTGGTACAAAGGGCAAATGACGGACCTCTACCATTACATTACATCATATGTAGTAGACAATGACATAATTATATCTGGGCCTGCATACACTGGACGAGAAACAGTATATTCCAACGCATCCCTGCTGnnnnnnnnnnnnnnnnnnnnnnnnnnnnnnnnnnnnnnnnnnnnnnnnnnnnnnnnnnnnnnnnnnnnnnnnnnnnnnnnnnnnnnnnnnnnnnnnnnnn

>Nle_PSG11N (Nomascus leucogenys; northern white-cheeked gibbon) WGS ADFV01160544.1

CATCACTTCTAAACTTCTGGAACCCGCCTACCACTGCCCAAGTCATGATTGAAGCCCAGCCACCCAAAGTTTCCGAGGGGAAGGATGTTCTTCTACTTGTCCACAATTTGCCCCATAATCTTGCCAGCTATAGCTGGCACAAAGGGCGAATGATGGACCTCCACAATTACATTACATCATATGTAGTAGCCAGACAAATAATTATATCTGGGCCTGCATACAGTGGACGAGAAAAACTATATTCCAATGCATCCCTGCTGATCCAGAATGTCACCAGGGAGGACGCAGGATCCTACACCTTACACATCAGACAACCAGGTGATGGGATTAGAGGAGCATCTGnnnnnnnnnnnnnnnnnn

>Pdo_PSG4N (Plecturocebus donacophilus; Bolivian titi) WGS PVKP010106024.1

nnnnnnnnnnnnnnnnnnnnnnnnnnnnnnnnnnnnnnnnnnnnnnnnnnnnnnnnnnGCCACACATTGTTTCAGAGGGGAAGGATGTTCTTCTACTTGTCCACAATTTGCCCCAGAATCTTACTGGCTACAGCTGGTACAGAGGGAAAGTGATGGACATCCACCATTACATTACAGCATATTTAATAGAAAAAGAAATAATTATATATGGGCATGCATACAGTGGACGAGAAACAATATATTCCAATGCATCCCTGTTGATCCAGAACGTCACCCTGAATGACACAGGATCCTACACCCTGCAAGTCGTCAATCAAGGTGAAAGGAATAAAGGAGTAACTGCACATTTCACCTTACACC

>Rbi_PSGP4N (Rhinopithecus bieti; black snub-nosed monkey) WGS MCGX01001961

nnnnnnnnnnnnnnnnnnnnnnnnnnnnnnnnnnnnnnnnnnnnnnnnnnnnnnnnnnnnnnnnnnnnnnnnnnnnnnnnnnnnnnnnnnnnnnnnnnnnnnnnnnnnnnnnnnnnnnnnnnnnnnnnnnnntctggtacaaagggcaaataatggacctccaccattacattacagcatatacaatagacactgaaatgattatatttgggcctgcatacagtggatgagaaactgtatatcccaatgcatccctgttgatccagaatgtcacccagaaggacacaggatcctacaccattcaaatcacacagcgaggtgatgggactaaaggagtaactggacatttcaccttatacc
