# Supplementary material for: The immune-modulating pregnancy-specific glycoproteins evolve rapidly and their presence correlates with hemochorial placentation in primates
Source: BMC Genomics. 2021 Feb 18;22:128. doi: 10.1186/s12864-021-07413-8 (PMC7893922; doi:10.1186/s12864-021-07413-8)
Supplement: Supplementary file 6 — Additional file 6: Supplementary Table 1. CEACAM1-like genes in primates. This table lists the common names of primate species, their abbreviation, Latin name, taxonomic classification, genomic data source and the number and types of CEACAM1-related genes and pseudogenes. [file 12864_2021_7413_MOESM6_ESM.docx]

**Supplementary Table 1: Primate CEACAM families**

| **abbre-viation** | **Latin name** | **common name** | **classification** | **genomic data source^a^** | **genome coverage**  **sequencing technology** | **CEACAM1-related genes** | | | | | |
| --- | --- | --- | --- | --- | --- | --- | --- | --- | --- | --- | --- |
|  |  |  |  |  |  | **total^b^** | **pseudogenes with N exons^c^** | **GPI-linked members** | **members with ITAM-like motif** | **members with ITIM/ITSM^d^** | **PSG**  **non-ps/ps** |
| Age | *Ateles geoffroyi* | black-handed spider monkey | New World monkeys | PVHS00000000.1 | 60.0x  Illumina HiSeq | 9^e^ | 0 | 4 | 0 | 1/0 | 3/0 |
| Ana | *Aotus nancymaa^e^* | Ma's night monkey | New World monkeys | Anan_2.0 | 132.4x  PacBio RSII; PacBio Sequel; Illumina | 10 | 0 | 4 | 0 | 1/0 | 5/0 |
| Apa | *Alouatta palliata* | mantled howler monkey | New World monkeys | PVKV000000000.1 | 43.1x  Illumina HiSeq | 13^e^ | 0 | 4 | 0 | 1/0 | 7/0 |
| Cal | *Cebus albifrons* | white-fronted capuchin | New World monkeys | PVKJ000000000.1 | 28.8x  Illumina HiSeq | 7^e^ | 1 | 3 | 0 | 0/1 | 1/0 |
| Can | *Colobus angolensis palliatus* | black and white colobus monkey | Old World monkeys | Cang.pa_1.0  JYKR00000000.1 | 86.8x  Illumina | 30 | 5 | 4 | 2 | 1/0 | 18/5 |
| Cat | *Cercocebus atys* | sooty mangabey | Old World monkeys | Caty_1.0  JZLG00000000.1 | 192.0x  Illumina; PacBio RS | 31 | 2 | 4 | 2 | 1/0 | 22/2 |
| Cca | *Cebus capucinus imitator* | capuchin monkey | New World monkeys | Cebus_imitator-1.0  LVWQ00000000.1 | 81.0x  Illumina | 7^e^ | 0 | 4 | 0 | 0/1 | 1^f^/0 |
| Cja | *Callithrix jacchus* | marmoset | New World monkeys | C_jacchus3.2.1  ACFV00000000.1 | 6.6x  ABI 3730 | 9 | 0 | 4 | 0 | 1/0 | 4/0 |
| Cme | *Cheirogaleus medius* | lesser dwarf lemur | lemurs | PVHR00000000.1  VBSM00000000.1 | 64.9x  Illumina HiSeq  110.0x  Illumina | 9 | 0 | 2 | 2 | 0/1 | 0/0 |
| Cne | *Cercopithecus neglectus* | De Brazza's monkey | Old World monkeys | PVKI010000000.1 | 27.5x  Illumia HiSeq | 33 | 4 | 3 | 2 | 1/0 | 22/5 |
| Csa | *Chlorocebus sabaeus* | green monkey | Old World monkeys | UCSC chlSab2  AQIB00000000.1 | 95.0x  454 Titanium Illumina HiSeq; ABI | 26 | 6 | 4 | 2 | 1/0 | 15/5 |
| Dma | *Daubentonia madagascariensis* | aye-aye | lemurs | AGTM000000000.1AGTM000000000 | 38.0x  Illumina GA IIx | 8 | 2 | 1 | 1 | 0/2 | 0/0 |
| Efl | *Eulemur flavifrons* | blue-eyed black lemur | lemurs | LGHW00000000.1 | 52.0x  Illumia HiSeq | 5 | 0 | 1 | 2 | 0/1 | 0/0 |
| Efu | *Eulemur fulvus* | brown lemur | lemurs | PVJU000000000.1 | 63.5x  Illumia HiSeq | 8 | 0 | 2 | 2 | 0/1 | 0/0 |
| Ema | *Eulemur macaco* | black lemur | lemurs | LGHX00000000.1 | 21.0x  Illumia HiSeq | 5 | 0 | 2 | 2 | 0/1 | 0/0 |
| Epa | *Erythrocebus patas* | red guenon | Old World monkeys | PVJV000000000.1 | 43.7x  Illumia HiSeq | 33 | 1 | 4 | 2 | 1/0 | 24/1 |
| Ggo | *Gorilla gorilla* | gorilla | great apes | gorGor4  CYUI00000000.3 | no information | 21^g^ | 7 | 4 | 2 | 1/0 | 5/2 |
| Gva | *Galeopterus variegatus* | Malayan flying lemur | flying lemurs | UCSC galVar1  JMZW00000000.1 | 55.0x  Illumina | 18 | 4 | 5 | 1 | 0/1 | 0/0 |
| Hmo | *Hylobates moloch* | silvery gibbon | gibbons | WKKJ00000000.2 | 70.0x  Illumina; Oxford Nanopore; PacBio; 10X Chromium | 19^g^ | 4 | 4 | 1 | 1/0 | 8/1 |
| Hsa | *Homo sapiens* | man | great apes | GRCh38.p10 | high quality | 24^g^ | 5 | 4 | 2 | 1/0 | 11/0 |
| Iin | *Indri indri* | Indri | lemurs | RJWJ010000000.1 | 34.4x  Illumina HiSeq | 6 | 1 | 2 | 2 | 0/1 | 0/0 |
| Lca | *Lemur catta* | ring-tailed lemur | lemurs | Pcoq_1.0  PVHV00000000.1 | 56x  Illumina HiSeq | 5 | 0 | 2 | 2 | 0/1 | 0/0 |
| Mco | *Mirza coquereli* | Coquerel's mouse lemur | lemurs | PVHQ00000000.1 | 60.7x  Illumina HiSeq | 8 | 1 | 2 | 3 | 0/1 | 0/0 |
| Mfa | *Macaca fascicularis* | crab-eating macaque | Old World monkeys | Macaca_fascicularis_5.0  AQIA00000000.1 | 68.0x  Illumina HiSeq | 26 | 0 | 2 | 2 | 1/0 | 22/0 |
| Mfu | *Macaca fuscata fuscata* | Japanese macaque | Old World monkeys | BFBW00000000.1 | 42.0x  Illumina HiSeq X | 24 | 0 | 4 | 2 | 1/0 | 17/0 |
| Mgr | *Microcebus griseorufus* | Reddish-gray mouse lemur | lemurs | VSMI00000000.1 | 40.0x  Illumina HiSeq | 8 | 1 | 2 | 4 | 0/1 | 0/0 |
| Mje | *Microcebus jenahi* | Microcebus sp. 3 GT-2019 | lemurs | VSMF01000000.1 | 43.0x  Illumina HiSeq | 12 | 2 | 2 | 6 | 0/1 | 0/0 |
| Mle | *Mandrillus leucophaeus* | drill | Old World monkeys | Mleu.le_1.0  JYKQ00000000.1 | 117.2x  Illumina | 22 | 2 | 4 | 2 | 1/0 | 14/2 |
| Mmi | *Microcebus mittermeieri* | Mittermeier's mouse lemur | lemurs | VSMH01000000.1 | 35.0x  Illumina HiSeq | 16 | 2 | 2 | 4 | 0/1 | 0/0 |
| Mml | *Macaca mulatta* | rhesus macaque | Old World monkeys | Mmul_10  SBKD00000000.1  VSDM010000000.1 | 60.0x  PacBio RSII | 31 | 3 | 4 | 2 | 1/0 | 21/3 |
| Mmr | *Microcebus murinus* | gray mouse lemur | lemurs | Mmur_3.0  ABDC00000000.3 | 221.6x  Illumina; PacBio RS; PacBio RSII | 8 | 1 | 1 | 5 | 0/1 | 0/0 |
| Mne | *Macaca nemestrina* | pig-tailed macaque | Old World monkeys | Mnem_1.0  JZLF00000000.1 | 113.1x  Illumina; PacBio RS; PacBio RSII | 29 | 1 | 4 | 2 | 1/0 | 21/1 |
| Mra | *Microcebus*  *ravelobensis* | Golden-brown mouse lemur | lemurs | VSMG01000000.1 | 26.0x  Illumina HiSeq | 10 | 0 | 3 | 4 | 0/2 | 0/0 |
| Msp | *Mandrillus sphinx* | Mandrill | Old World monkeys | SRPC00000000.1 | 96.0x  Illumina HiSeq | 14 | 2 | 4 | 2 | 1/0 | 6/1 |
| Mta | *Microcebus tavaratra* | Northern rufous mouse lemur | lemurs | VSME01000000.1 | 41.0x  Illumina HiSeq | 16 | 2 | 2 | 6 | 0/1 | 0/0 |
| Mza | *Mirza zaza* | Northern giant mouse lemur | lemurs | VSMD00000000.1 | 44.0x  Illumina HighSeq | 9 | 0 | 2 | 3 | 0/1 | 0/0 |
| Nla | *Nasalis larvatus* | proboscis monkey | Old World monkeys | UCSC nasLar1  JMHX00000000.1 | 290.0x  454; Illumina HiSeq | 12 | 2 | 4 | 2 | 1/0 | 18/7 |
| Nle | *Nomascus leucogenys* | Northern white-cheeked gibbon | gibbons | Nleu3.0  ADFV00000000.1 | 5.6x  Sanger | 17^g^ | 0 | 3 | 1 | 1/0 | 11/0 |
| Oga | *Otolemur garnettii* | bush baby | galagos | OtoGar3  AAQR00000000.3 | 137.0x  Illumina GAIIx | 8 | 1 | 1 | 1 | 0/1 | 0/0 |
| Pab | *Pongo abelii* | Sumatran orangutan | great apes | PPYG2  NDHI00000000.3 | 101.2x  PacBio; Illumina NextSeq 500; BioNano; Saphyr | 23^g^ | 6 | 4 | 2 | 1/0 | 10/1 |
| Pan | *Papio anubis* | olive baboon | Old World monkeys | Panu_3.0  AHZZ00000000.2 | 104.0x  Sanger 3730; 454 FLX; Illumina; PacBio | 28 | 3 | 4 | 2 | 1/0 | 18/3 |
| Pco | *Propithecus coquereli* | Coquerel's sifaka | lemurs | Pcoq_1.0  JZKE00000000.1 | 104.7x  Illumina | 6 | 1 | 2 | 2 | 0/1 | 0/0 |
| Pdo | *Plecturocebus donacophilus* | Bolivian titi | New World monkeys | PVKP000000000.1 | 23.7x  Illumia HiSeq | 10^e^ | 0 | 4 | 0 | 1/0 | 4/0 |
| Pne | *Pygathrix nemaeus* | red shanked douc langur | Old World monkeys | PVHW000000000.1 | 65.5x  Illumia HiSeq | 17 | 1 | 3 | 2 | 1/0 | 10/1 |
| Ppa | *Pan paniscus* | bonobo | great apes | panpan1.1  AJFE00000000.2 | 26.0x  454 GS FLX; 454 GS FLX Titanium | 20 | 4 | 4 | 2 | 1/0 | 9/0 |
| Ppi | *Pithecia pithecia* | white-faced saki | New World monkeys | PVIP00000000.1 | 47.3x  Illumia HiSeq | 10^e^ | 0 | 4 | 0 | 1/0 | 4/0 |
| Psi | *Prolemur simus* | greater bamboo lemur | lemurs | MPIZ00000000.1 | 152.7x  Illumina | 7 | 0 | 2 | 2 | 0/3 | 0/0 |
| Pte | *Piliocolobus tephrosceles* | Ugandan red colobus | Old World monkeys | PDMG00000000.3 | 38.61x  Illumia HiSeq | 18 | 2 | 3 | 1 | 1/0 | 11/2 |
| Ptr | *Pan troglodytes* | chimpanzee | great apes | Pan_tro_3.0  NBAG00000000.3 | 124.0x  PacBio; Illumina NextSeq 500; BioNano; Saphyr | 21 | 4 | 4 | 2 | 1/0 | 10/0 |
| Rbi | *Rhinopithecus bieti* | black snub-nosed monkey | Old World monkeys | MCGX01000000 | 76.6x  Illumia HiSeq | 21 | 4 | 2 | 2 | 1/0 | 12/4 |
| Rro | *Rhinopithecus roxellana* | golden snub-nosed monkey | Old World monkeys | Rrox_v1  JABR00000000.1 | 53.7x  Illumia HiSeq | 20 | 1 | 3 | 2 | 1/0 | 13/1 |
| Sap | *Sapajus apella* | tufted capuchin | New World monkeys | WRPQ01000000 | 34.0x  Illumia HiSeq | 7^e^ | 0 | 4 | 0 | 0/1 | 1/0 |
| Sbo | *Saimiri boliviensis boliviensis* | Bolivian squirrel monkey | New World monkeys | SaiBol1.0 | 80.0x  Illumia HiSeq | 7^e^ | 1 | 4 | 0 | 1/0 | 1^f^/0 |
| Sen | *Semnopithecus entellus* | Hanuman langur | Old World monkeys | PVII000000000.1 | 29.3x  Illumia HiSeq | 22 | 2 | 3 | 2 | 1/0 | 14/2 |
| Sim | *Saguinus imperator* | tamarin | New World monkeys | PVHO000000000.1 | 61.4x  Illumia HiSeq | 7^e^ | 1 | 3 | 0 | 1/0 | 2/0 |
| Tbe | *Tupaia belangeri* | Northern tree shrew | tree shrews | AAPY01000000 | 2.0x | 5 | 1 | 0 | 2 | 0/1 | 0/0 |
| Tch | *Tupaia chinensis* | Chinese tree shrew | tree shrews | ALAR01000000 | 80.0x  Illumina HiSeq 2000 | 6 | 2 | 0 | 2 | 0/1 | 0/0 |
| Tfr | *Trachypithecus francoisi* | Francois's langur | Old World monkeys | VVIV00000000.1 | 1.0x  Illumina | 25 | 4 | 3 | 2 | 1/0 | 15/3 |
| Tge | *Theropithecus gelada* | gelada baboon | Old World monkeys | QGDE00000000.1 | 56.0x  Illumia HiSeq | 21 | 1 | 4 | 2 | 1/0 | 13/1 |
| Tsy | *Tarsius syrichta* | tarsier | tarsiers | ABRT00000000.2  Tarsius_syrichta-2.0.1 | 48.0x  Sanger; 454; Illumina | 15 | 4 | 4 | 5 | 0/1 | 0/0 |
| Tta | *Tupaia tana* | large tree shrew | tree shrews | RJWV010000000 | 25.0x  Illumina HiSeq | 6 | 2 | 0 | 2 | 0/1 | 0/0 |

^a^ Ensembl databases; UCSC, UCSC Genome Browser assembly; GenBank

^b^ CEACAM gene identification in species with unfinished genome sequences are based partly on N domain exon counts. N exon sequences with >99% identity were considered to represent alleles; pseudogenes included; for low coverage genome sequences gene numbers represent a minimal estimate

^c^ Genes were registered as pseudogenes when stop codons or frame shift mutations were detected in N domain exons

^d^ The number of members with ITIM or ITSM motifs at the C-terminal end are separated by slashes

^e^ Including the New World monkey-specific *PSG-like* gene (*PSGL*)

^f^ No N exon found, only IgC-like domain exons, locus with sequencing gaps

^g^ Including the great ape-specific *CEACAM21*

CEACAM, CEA-related cell adhesion molecule; GPI, glycosylphosphatidyl inositol; ITAM, immunoreceptor tyrosine-based activation motif; ITIM, immunoreceptor tyrosine-based inhibition motif; ITSM, immunoreceptor tyrosine-based switch motif; ps, pseudogene, PSG, pregnancy-specific glycoprotein.
